# Public open spaces and well-being: a comparative study of migrant and local older adults in Dongguan

**DOI:** 10.3389/fpubh.2026.1776572

**Published:** 2026-03-12

**Authors:** Hang Cheng, Xiaoli Shi, Jiaxu Huang, Xi Ye, Guoliang Xu

**Affiliations:** 1 School of Humanities and Management, Guangdong Medical University, Dongguan, China; 2 Chow Yei Ching School of Graduate Studies, City University of Hong Kong, Hong Kong, Hong Kong SAR, China; 3 Faculty of Humanities and Arts, Macau University of Science and Technology, Macau, Macao SAR, China

**Keywords:** aging population, migrant differences, public open spaces, subjective well-being, urban environment

## Abstract

**Introduction:**

As China faces the growing challenges of population aging, understanding the subjective well-being of older adults has become increasingly important. This study examines the role of public open spaces in shaping the social well-being of older adults, with particular attention to the environmental adaptability and vulnerability of migrant older adults. Specifically, it compares the subjective well-being of migrant and local older adults and explores how access to and use of public open spaces influence their overall well-being.

**Methods:**

A mixed-methods approach was adopted. A total of 325 neighborhood-based interviews were conducted in Dongguan. Qualitative data were analyzed using NVivo 12.0 for thematic coding to identify key dimensions affecting subjective well-being.

**Results:**

The findings indicate a generally high level of subjective well-being among older adults; however, local residents reported more positive evaluations than migrant older adults. Public safety, environmental cleanliness, and accessibility were identified as major factors influencing well-being. In contrast, challenges related to modern information technology systems and recreational facilities varied between migrant and local groups.

**Discussion:**

The results highlight the critical role of public open spaces in promoting social participation and enhancing the well-being of older adults. These findings provide important implications for urban planning and public policy, emphasizing the need to develop inclusive, age-friendly environments that support both well-being and social integration in urban contexts.

## 1 Introduction

China is facing an urgent challenge: the rapid and extensive aging of its population. According to the seventh national census, the proportion of the population aged 60 and above increased from 13.50% in 2010 to 18.70% (approximately 264 million people) in 2020 (1). With improvements in healthcare and rising life expectancy, it is projected that by 2040, over 28% of China’s population (around 402 million people) will be aged 60 and above, making it the country with the largest older adults population in the world (2–4). At the same time, population mobility continues to rise. Between 2010 and 2020, China’s migrant population grew from 221 million to 376 million, a 69.73% increase in just ten years. Notably, the number of older adults migrants has risen significantly, increasing from 10.61 million in 2010 to 33.27 million in 2020, with their share of the migrant population growing from 4.80 to 8.85% (5). This trend is expected to continue. The increase in older adults migrants reflects changes in family structures and living patterns between urban and rural areas, particularly as more older adults people move to cities with their children during the processes of industrialization and urbanization (6). These older adults individuals face multiple challenges, including adapting to changes in social support systems and new environments, making them more prone to feelings of isolation, difficulties in adaptation, and a decrease in subjective well-being compared to local older adults residents (7–9). Therefore, how to effectively enhance the subjective well-being of older adults migrants and help them better integrate into urban life has not only become a core goal in addressing the dual challenges of aging and population mobility, but also a key issue for policymakers and researchers to urgently resolve.

Subjective well-being is an important indicator for measuring the quality of life, referring to an individual’s overall emotional and cognitive evaluation of life (10, 11). It plays a crucial role in understanding the quality of life, especially among the older adults. Existing studies have shown that factors such as economic status, cultural connections, social support, and environmental conditions significantly affect the subjective well-being of older adults (12–14). Among these, environmental conditions, as a key factor influencing subjective well-being, have received increasing attention in recent years. The UN Decade of Healthy Ageing 2020–2030 (www.who.int/initiatives/decade-of-healthy-ageing) also emphasizes the role of physical environments in determining the physical and mental capacities of older adults. However, despite the recognition of the impact of environmental attributes on well-being, many studies have failed to delve into the specific characteristics of physical spaces, particularly public open spaces. Public open spaces, as an essential part of urban environments, are critical for social interaction, physical activity, and the mental health of older adults (7–9, 15). These spaces broadly include not only parks, squares, neighborhood gardens, and green spaces but also indoor spaces accessible to the public, such as libraries, neighborhood centers, and shopping malls (16). Public open spaces provide opportunities for interaction with nature, relaxation, and physical exercise, while also creating environments for social engagement, mental rejuvenation, and stress relief for older adults. These positive environmental experiences play an important role in improving the subjective well-being of older adults (17–19).

Nevertheless, despite existing studies highlighting the significant benefits of public open spaces for the well-being of older adults, research still lacks a detailed exploration of how different types of public open spaces uniquely influence the subjective well-being of older adults migrants, especially in the context of rapid urbanization. As urbanization accelerates and the older adults migrant population continues to grow, further investigation into the influence of these spatial attributes on the subjective well-being of both migrant and local older adults is crucial. Therefore, the objectives of this study are:

1. To recognise similarities and differences in subjective well-being between migrant and local older adults.
2. To identify the key attributes of public open spaces that influence the subjective well-being of older adults, and to assess the migrant-local differences in these influences, with the aim of informing age-friendly urban planning and policy.

This study conducts an empirical investigation using Dongguan, an industrialized city in China, as a case study. Structured interviews were conducted with both migrant and local older adults individuals in Dongguan to explore the key factors influencing the subjective well-being of older adults in the context of China’s population aging challenge and the impact of public open spaces on their subjective well-being. The primary contributions of this study are as follows: (1) Differential Subjective Well-being between Migrant and Local Older Adults: This study reveals variations in subjective well-being between migrant and local older adults individuals in industrialized urban settings. Migrant older adults face greater challenges in adapting to their environment in urbanization, offering valuable insights for urban planning and social policies. (2) In-depth Analysis of Public Open Space Attributes: This research offers a comprehensive analysis of the specific attributes of public open spaces and their relationship with older adults’ subjective well-being, thereby addressing a research gap in environmental factors. By understanding the role of public open spaces in older adults’ well-being, substantial recommendations are provided for designing and improving age-friendly urban environments. (3) Strategic Recommendations for Urban Policy and Design: Grounded in thoroughly investigating differential subjective well-being among older adults and the influencing factors of public open spaces, this study provides strategic recommendations for urban policies and design. Measures aimed at optimizing urban infrastructure and enhancing the quality of public spaces contribute to meeting the older population’s needs, fostering social integration, and promoting urban sustainability.

## 2 Theoretical framework

### 2.1 Migrant older adults and subjective well-being

Since the implementation of the reform and opening-up policy in China in 1978, the country has undergone massive population migration. In order to promote the industrial and social development of China, nearly one-fifth of the Chinese population has entered industrialized regions (20). The population migration in China mainly involves rural-to-urban migration, called “interregional migration.” Most internal migrants do not have a household registration (hukou) in the city where they moved to but remain registered in their place of origin. The number continues to grow (21). Chinese cities have different criteria for granting household registration to restrict population migration. Only registered residents can access certain benefits, with more developed cities often setting higher standards for registration, while less developed cities are more willing to accept migrants to promote local economic growth (22, 23). For the majority of migrant older adults in China, migration may not contribute directly to economic growth but is more family-oriented—many migrate to reunite with their children or to provide familial support for younger generations. This group of older migrants is often referred to as “drifting older adults” (24, 25).

Subjective well-being is a broad concept that has been widely discussed in academic literature (10, 11). It refers to a person’s overall evaluation of their quality of life, combining both emotional and cognitive assessments. Subjective well-being encompasses a person’s emotional reflections on their happiness, life satisfaction, and fulfillment, making it a key indicator of psychological and emotional health (26, 27). Numerous studies have assessed subjective well-being through self-reported life satisfaction, affect balance, and domain satisfaction (e.g., satisfaction with family life, social relations, and health) (28–30). The literature suggests that subjective well-being is influenced by a range of factors, including economic, social, and environmental conditions. When older migrants move from rural to urban areas, they face significant challenges, including changes in social relations, lifestyles, and their environment (31). These factors can impact their subjective well-being, particularly as older migrants are often more vulnerable than younger ones. Studies have shown that the subjective well-being of Chinese migrant older adults is lower than that of local older adults (18, 32–34). Similar trends have been observed in international studies, where migrant older adults in Europe report lower subjective well-being than natives (35, 36). However, some studies suggest that migrant older adults can experience similar or even higher subjective well-being than their native counterparts in certain conditions (37, 38). Thus, migrant-local differences in subjective well-being remain a complex topic that warrants further exploration.

### 2.2 Determinants of subjective well-being

Understanding the subjective well-being of migrant older adults requires not only identifying its overall level but also clarifying the key determinants that shape it. Existing literature generally conceptualizes these determinants as encompassing economic, social, familial, and environmental dimensions, each of which plays a distinct yet interconnected role in shaping older adults’ life satisfaction and emotional experiences.

#### 2.2.1 Economic determinants

It is necessary to understand not only to what extent the subjective well-being of migrant older adults is affected, but also what factors play a part. The determinants of subjective well-being include economic status (26, 39). A study on the subjective well-being of Korean migrant older adults in the United States found that employment and self-assessed economic status have a significant positive relationship with subjective well-being (40). A European study similarly found that the subjective well-being of migrant older adults is positively associated with their income levels, especially for those in higher-income brackets (36). This suggests that financial security is a critical factor contributing to the well-being of older migrants (41–43), particularly those adapting to new urban environments where the cost of living may differ from their original locations.

#### 2.2.2 Social determinants

In addition to economic factors, social attributes play a crucial role in determining subjective well-being. A study conducted in Japan found that efforts to prevent social isolation among older adults migrants had a significantly positive effect on their well-being (17). Similar findings emerged from studies in Australia and Israel, where language proficiency in the local language was found to increase subjective well-being among migrant older adults (44, 55). These studies highlight the importance of social integration, with language fluency and social networks being essential to fostering a sense of belonging in new communities.

#### 2.2.3 Family and intergenerational determinants

Family-related attributes are another key determinant of subjective well-being for the migrant older adults (46, 47). In China, intergenerational support—such as care provided by children or vice versa—has been found to positively impact the well-being of older adults migrants who move to urban centers to be closer to their children (18). However, there is also evidence suggesting that imbalanced intergenerational reciprocity, such as when older adults provide more care than they receive, can negatively affect their quality of life (48, 49). A study on older Chinese migrants in New Zealand showed that this imbalance led to decreased subjective well-being due to unmet expectations and cultural differences regarding family roles (50). These findings underscore the complex interplay between family dynamics and well-being, especially in the context of migration.

#### 2.2.4 Environmental determinants

In recent years, researchers have increasingly turned their attention to environmental determinants of subjective well-being. Environmental attributes such as residential settings, access to public facilities, and the availability of green spaces have been linked to improved well-being among older adults populations (51, 52). For example, studies have shown that access to green spaces can enhance both the physical and mental health of older adults by providing spaces for exercise, relaxation, and social interaction (19, 53, 54).

#### 2.2.5 Public open spaces as a key environmental dimension

Within the broader category of environmental determinants, public open spaces represent a particularly salient component of the urban physical environment for migrant older adults. There is a notable gap in the literature regarding how specific attributes of the urban physical environment—such as public open spaces—impact the subjective well-being of migrant older adults. Public open spaces, which include both outdoor and indoor areas like parks, neighborhood centers, and libraries, are particularly important for older adults who may face social isolation or reduced mobility in urban settings. Moreover, the availability and quality of public open spaces can influence the extent to which older adults migrants engage with their communities and maintain active lifestyles (55, 56). Research has shown that well-maintained and accessible public spaces promote social inclusion, physical activity, and mental relaxation, all of which are vital for maintaining high levels of subjective well-being (57). For migrant older adults, these spaces can also serve as platforms for building social networks, forming new friendships, and participating in neighborhood activities—factors that have been repeatedly linked to higher levels of well-being (19, 58).

#### 2.2.6 Urban context and environmental inequality in China

In the Chinese context, urbanization has transformed many cities into dense, highly developed areas where public spaces may be limited or unevenly distributed (59). This is particularly significant for migrant older adults, who may not have access to the same social and environmental amenities as local residents (60, 61). A growing body of research in China highlights the need to consider these disparities when assessing the well-being of older adults. For instance, recent studies have shown that access to green spaces and walkable neighborhoods in Chinese cities can significantly enhance the subjective well-being of older residents, including migrants, by providing them with opportunities for physical exercise and social interaction in a supportive environment (19). However, despite the growing recognition of the importance of public open spaces, there remains a lack of detailed investigation into how different types of these spaces—particularly in rapidly urbanizing Chinese cities—affect the subjective well-being of the migrant older adults. Understanding the specific attributes of public open spaces, such as their accessibility, safety, and quality, and how these influence the well-being of migrant and local older adults alike, is crucial for future research (55, 56, 60, 62).

### 2.3 Public open spaces and the support for older adult’s well-being

Public open spaces are defined as areas accessible to all individuals, where people from different social groups and age ranges can engage in social activities, participate in neighborhood events, and access social services in a democratic, inclusive manner (63–65). In the context of China’s urban environments, public open spaces not only refer to outdoor spaces such as parks, squares, and green areas, but also indoor spaces like libraries, neighborhood centers, and shopping malls (66). These spaces provide crucial opportunities for physical activity and social interaction, both of which are essential to the well-being of older adults (67). Research has shown that public open spaces have a positive impact on older adults’ physical and social well-being (56, 58). Older adults often use these spaces for physical activities, such as walking, and for social interactions, such as meeting friends or participating in group activities (69). Physical environments, such as walkable streets, have been found to enhance place attachment, which in turn contributes to older adults’ emotional health (70). In addition, green spaces play an essential role in promoting physical and mental health, offering opportunities for relaxation and stress reduction (71, 72).

The impact of public open spaces on cognitive health has also emerged as a growing area of interest (73). Studies suggest that regular use of green spaces may help mitigate age-related cognitive decline and reduce the risk of dementia in older adults by fostering mental engagement and reducing stress levels (74, 75). Even in highly urbanized settings, the incorporation of natural elements into public open spaces—such as shaded walkways, pocket parks, or indoor atriums—has been associated with improved cognitive performance and emotional resilience among older adults (76). Furthermore, public open spaces can foster a sense of neighborhood belonging among older adults, which plays a vital role in their overall well-being (77). Social integration in communal spaces helps reduce feelings of isolation, a common issue faced by the older adults, particularly in urban settings (78). By facilitating repeated social encounters and informal interactions, public open spaces support the formation of local social networks that are essential for emotional security and subjective well-being.

In the Chinese context, public open spaces are not only centers for leisure and exercise but also act as important cultural hubs where traditional activities, such as tai chi, square dancing, and calligraphy practice, are regularly performed (79, 80). These activities provide a unique social and cultural function that may not be as prominent in Western settings. Research indicates that participation in these culturally embedded practices enhances the sense of identity and neighborhood among older adults, contributing positively to their subjective well-being (81, 82). These patterns of use are closely aligned with Confucian values that emphasize harmony between humans and nature (tian–ren heyi), social cohesion, and intergenerational solidarity (83). In practical terms, these values are manifested in the spatial design and functional organization of public open spaces, such as the provision of open plazas for collective activities, shaded seating areas that encourage prolonged social interaction, and barrier-free pathways that accommodate older adults’ mobility needs. Moreover, Confucian ethics place strong emphasis on family continuity and intergenerational interaction, which is reflected in the design of family-oriented public open spaces in China. Communal gardens, multi-generational activity areas, and neighborhood squares serve as shared spaces where older adults, children, and younger family members can interact, reinforcing emotional bonds and cultural continuity (84). For migrant older adults in particular, such spatial configurations help compensate for disrupted social networks by offering opportunities to rebuild social ties and maintain culturally familiar practices in a new urban environment. However, most existing studies on public open spaces and well-being have been conducted in Western contexts, where cultural norms, family structures, and patterns of space use differ significantly from those in China. There remains a lack of theoretically grounded research that explicitly links Confucian cultural values with concrete public open space attributes and their impacts on older adults well-being in Chinese cities. Addressing this gap is essential for developing a context-sensitive framework that captures how cultural values, spatial design, and everyday practices jointly shape the subjective well-being of both migrant and local older adults populations.

## 3 Methods

### 3.1 Research subjects

This study selects Dongguan as a case study, primarily due to its unique background and socioeconomic characteristics as a rapidly industrialized city in China. Guangdong Province, as China’s economic hub, has consistently been at the forefront of the country’s industrialization process. In recent years, as core cities in Guangdong, such as Guangzhou and Shenzhen, have undergone industrial restructuring, a large number of manufacturing facilities have gradually shifted to surrounding cities like Dongguan, fueling the city’s rapid industrial growth (85, 86). As a result, Dongguan has been dubbed the “world’s factory” and has become one of China’s key manufacturing hubs (87, 88).

With the advancement of industrialization, Dongguan has not only dominated the manufacturing sector but has also become a representative example of the widespread use of digital technologies (88, 89). This industrialization is not only reflected in the physical expansion of factories but also in the extensive use of digital technologies, such as computers, smartphones, and mobile applications (90). The development of industrial digitalization has spurred the proliferation of information technology, particularly in a manufacturing-dense city like Dongguan, where computers and mobile devices have become essential tools in both social and work life. The application of these technologies has not only enhanced productivity but has also provided older adults with more opportunities to use smart devices to engage in social activities. In particular, public open spaces have allowed the older adults to use mobile applications to access information and participate in both online and offline activities, further promoting social interaction.

At the same time, Dongguan’s population structure has undergone significant changes with the influx of migrant populations as industrialization has progressed. Migrants now account for 52% of the city’s total population (91, 92). This substantial proportion of migrant residents provides a rich social context for studying the differences between migrant and local populations (93). Under the backdrop of rapid urbanization and industrialization, the influx of older adults migrants has become a prominent social phenomenon, especially as more older adults move to cities to reunite with their families.

Moreover, Dongguan is gradually entering an aging society, with the population aged 60 and above reaching 572,600, accounting for 5.67% of the total population (91). Although this proportion may seem small, the trend of aging is accelerating, especially in a manufacturing-driven urban environment where the social and economic transformation of the older adults population is particularly evident (94). Therefore, choosing Dongguan as a case study helps to comprehensively explore the characteristics of subjective well-being among the older adults in the context of industrialization and urbanization, especially the impact of public open spaces on different population groups.

By selecting Dongguan as the focus city for this research, the study captures the complex interactions between migrant older adults, local older adults, subjective well-being, and public open spaces, providing a solid academic and practical foundation for studying the well-being of older adults populations in the context of rapid urbanization and industrialization in China.

The target group for this research includes two subgroups:

Migrant older adults: Migrant older adults were defined as individuals aged 60 and above who moved to Dongguan without transferring their household registration (hukou remains registered elsewhere) after the age of 60. Those who transferred their hukou to Dongguan after age 60 were also excluded, as the focus of our study was on the group that is structurally disadvantaged in terms of institutional access, social benefits, and urban identity. Hukou status in China remains a key determinant of access to public services and social integration, and individuals without a local hukou face more systemic barriers, which in turn affects their subjective well-being. By applying this criterion, we sought to capture a more vulnerable segment of the older adults migrant population.
Local older adults: Local older adults in our study were defined as individuals aged 60 and above who both hold a Dongguan household registration (hukou) and have lived continuously in Dongguan since birth. Individuals who obtained a Dongguan hukou later in life but did not grow up or live most of their lives in Dongguan were excluded from the “local older adults” group. This distinction was made to ensure consistency in terms of lifelong local residency and environmental/social familiarity, which are important contextual factors influencing subjective well-being and adaptation to urban settings.

By examining these two groups, we can better understand the migration dynamics of the older adults population, their adaptation to urban environments, and the role of public spaces in enhancing their subjective well-being.

### 3.2 Experimental procedure

The data collection process for this study was conducted through semi-structured interviews between December 15, 2022 and November 28, 2023. The purpose of these interviews was to explore the subjective well-being of older adults individuals in Dongguan and their interactions with public open spaces. The interview approach was designed to gather both qualitative insights and measurable data to understand the factors influencing well-being among both migrant and local older adults populations. It is worth noting that the interviewees in this survey totaled five individuals, all of whom are university faculty members with doctoral degrees. Prior to the survey, all of them received professional training.

#### 3.2.1 Participant selection

Participants were recruited using a snowball sampling technique, which allowed the research team to access a diverse set of participants, particularly older adults migrants who are not easily reachable through formal sampling frames. This approach was adopted due to the absence of a comprehensive registry of older adults migrants and the practical challenges of identifying this population in community settings. The recruitment process began with key “seed” participants who were purposefully selected based on their gender, age, and migration status. In addition, seed participants were selected from different residential neighborhoods in Dongguan to ensure variation in community environments and social contexts. These initial participants were asked to refer other older adults individuals, ensuring a wide range of backgrounds was represented in the study. To reduce homogeneity within referral chains, multiple seed participants were recruited simultaneously, and referrals were not restricted to a single social network or neighborhood.

##### 3.2.1.1 Pre-screening interviews

Pre-screening interviews were conducted to assess participants’ suitability for the study and to ensure that both migrant and local older adults with diverse living experiences were included. Key areas of inquiry included:

(A) Living conditions: This included questions about their housing, access to resources, and general environment in Dongguan.
(B) Migration history: For migrant older adults, questions were asked about their reasons for migrating to Dongguan, duration of residence, and ties to their place of origin.
(C) Outlook on life: Participants were asked about their general sense of happiness, mental health, and how they viewed their life in Dongguan compared to their previous experiences.

##### 3.2.1.2 Inclusion criteria

Inclusion criteria were applied consistently across all recruitment chains to maintain comparability between migrant and local older adults groups.

(A) All participants must be at least 60 years old, able to communicate independently, and have no significant language or cognitive impairments.
(B) Migrant older adults participants must have lived in Dongguan for at least 6 months, while local older adults participants must be long-term residents of Dongguan.

Although these procedures were designed to enhance sample diversity, the study does not claim statistical representativeness of the older adults population in Dongguan. Instead, the sample is intended to provide in-depth, context-specific insights into the lived experiences of migrant and local older adults in a rapidly industrializing urban environment.

#### 3.2.2 Interview process

The semi-structured interviews aimed to capture both subjective experiences and objective data on the participants’ well-being and their use of public open spaces. The interviews were designed to be flexible yet focused, allowing participants to freely express their thoughts while ensuring that key research topics were addressed.

##### 3.2.2.1 Duration

Each interview lasted approximately 30 to 40 min.

##### 3.2.2.2 Interview format

The interviews consisted of open-ended questions to encourage participants to share their personal experiences regarding their well-being, use of public open spaces, and any challenges they faced as older adults individuals in an urban environment. The questions were organized into specific themes, ensuring that all relevant areas were covered systematically.

Below is a Table 1 summarizing the key themes and example questions asked during the interviews.

**Table 1:** Key themes in the interviews.

Theme	Example questions
Subjective well-being	How would you evaluate your quality of life in Dongguan? Do you feel satisfied with your life?
	How has your emotional state changed compared to before you came to Dongguan?
Use of public open spaces	Which public open spaces do you usually use? Are parks, squares, library, and neighborhood centers places you frequently visit?
	Do you feel that these spaces are helpful in your daily life?
Perceptions of public open spaces	How do you understand ‘public open spaces’? What factors make these spaces more attractive to you?
Social support and economic support	Who do you usually talk to? If you have a problem, who do you typically seek help from?
Influential factors	How much do factors such as your economic situation and family support influence your well-being? Do you think these factors have had a significant impact on your life satisfaction?
	- Do you think living in Dongguan has positively or negatively impacted your well-being?

##### 3.2.2.3 Explanation of key concepts

When asking various questions, professional academic concepts involved need to be explained to the respondents. For example:

Subjective Well-being (SWB): During the interviews, researchers provided a detailed explanation of the concept of “subjective well-being” to participants. Subjective well-being was defined as an individual’s emotional and cognitive evaluation of their life, encompassing three dimensions: life satisfaction (a positive evaluation of life as a whole), the experience of positive emotions (such as happiness and contentment), and the reduction of negative emotions (such as anxiety and depression) (95, 96). Participants were guided to share their overall assessment of their current life, explore their daily emotional experiences, and discuss the extent to which they felt they were achieving their life goals.
Social Support: Researchers explained that social support includes not only support from family members but also social connections with friends, neighbors, and the community (97, 98). The study focused on how older adults receive emotional support through social interactions, such as whom they turn to for help when facing difficulties or with whom they maintain regular communication in daily life. For participants with no social support, it was recorded as zero.
Economic Support: Economic support refers to financial assistance received by participants from family members or other sources (99). The study explored the impact of economic support on the well-being of older adults by asking questions such as, “How much cash or material support did your children provide you in the past year?” The study also used log transformation on economic data to avoid bias caused by extreme values.
Public Open Spaces: Public open spaces were defined as outdoor and indoor areas that are freely accessible to the public, including parks, squares, libraries, and neighborhood centers (100). These spaces not only provide opportunities for social interaction and recreation for older adults but are also considered to have positive effects on physical and mental health. Participants were encouraged to share how they use these spaces and how these spaces specifically influence their well-being.

##### 3.2.2.4 Data collection

With participants’ permission, the interviews were audio-recorded to ensure that all responses were accurately captured. For detailed interview questions please refer to Supplementary file 1. Based on the expected effect size from similar studies and the available resources, we determined that a sample size of 325 participants would provide adequate statistical power to identify these differences. The power analysis was conducted using G*Power software, with a significance level of 0.05 and a power of 0.80, which is generally considered sufficient for social science studies. The analysis indicated that a sample of 325 participants was sufficient to achieve reliable and valid results.

In addition to verbal responses, non-verbal cues such as facial expressions, posture, and body language were observed and documented, providing further context for the participants’ emotional states and engagement with the interview topics. The interviews were transcribed verbatim, and thematic analysis was conducted using NVivo 12.0 to identify key themes and patterns related to subjective well-being and public space usage.

### 3.3 Variables

To provide a comprehensive understanding of the factors influencing subjective well-being, the study employed both subjective assessments and objective measures.

1. Subjective Well-being: Based on the Senior Subjective Well-being Scale (74) and Wang et al. (75), this variable was assessed using a series of questions during the interviews. These questions gauged participants’ overall satisfaction with life, their emotional state, and their perceived quality of life.
2. Older Adults Values: Drawing from Elizur (101), values were categorized into three types: material-oriented values, emotional-oriented values, and cognitive-oriented values. Participants’ priorities and value systems were explored to understand how these shaped their well-being.
3. Mental Health: The psychological well-being of participants was measured using a depression scale adapted from Wu Xiwei (102). Higher scores on this scale indicated lower levels of depression, thus reflecting better mental health.
4. Economic Support: Economic support was measured through participants’ self-reports of financial assistance from their children, based on Wang (103). The question “How much cash (or equivalent) have your children given you in the past year?” was used to gauge economic support, with data being transformed logarithmically to avoid skewness.
5. Emotional Support: Emotional support was assessed through questions such as “Who do you turn to for emotional support?” The more categories of individuals named (e.g., family, friends), the higher the level of emotional support recorded. If no support system was mentioned, a score of zero was recorded for emotional support.

To enhance measurement transparency and replicability, detailed information on all key variables, measurement scales, response ranges, and example questions is provided in Table 2. The subjective well-being scale and related measures were adapted from previously validated instruments, with minor wording adjustments to fit the local context. The internal consistency of these scales, with Cronbach’s alpha values above the 0.7 threshold, indicates that our sample has good reliability. Satisfaction with different attributes of public open spaces was assessed using a 10-point Likert-type scale, where higher scores indicated greater satisfaction. These ratings were treated as continuous variables and entered directly into the regression models as independent variables. Prior to analysis, the distributions of these variables were examined to ensure their suitability for parametric modeling.

**Table 2:** Key variable information table.

Variable	Measurement/Source	Response range	Example question
Subjective well-being	Chen (74) and Wang et al. (75)	1–5	“Overall, how satisfied are you with your current life?”
Mental health	Adapted depression scale (8)	1–5	“How often do you feel down or depressed?”
Sense of worth (values)	Elizur (101)	Categorical	“How much cash (or equivalent) have your children given you in the past year?”
Financial support	Wang (103)	Continuous (log-transformed)	“Who do you turn to for emotional support?”
Emotional support	Self-reported support sources	Count (0–n)	“Who do you usually turn to for emotional support?”
Marital status	Demographic variable	Categorical	“What is your current marital status?”
Pension insurance	Demographic variable	Binary (Yes/No)	“Do you have pension insurance?”
Length of immigration	Self-reported	Years	“How many years have you lived in Dongguan?”
Public security and order	Interview-based satisfaction rating	1–10	“How satisfied are you with public security in your neighborhood?”
Environmental cleanliness	Interview-based satisfaction rating	1–10	“How satisfied are you with environmental cleanliness?”
Public transportation	Interview-based satisfaction rating	1–10	“How satisfied are you with public transportation services?”
Technical infrastructure	Interview-based satisfaction rating	1–10	“How satisfied are you with digital services (e.g., mobile payment)?”
Walking safety	Interview-based satisfaction rating	1–10	“How safe do you feel when walking in your neighborhood?”
Walking mobility	Interview-based satisfaction rating	1–10	“How easy is it for you to move around on foot nearby?”
Entrances and corridors	Interview-based satisfaction rating	1–10	“How satisfied are you with building entrances and corridors?”
Lift installation and management	Interview-based satisfaction rating	1–10	“How satisfied are you with lift availability and maintenance?”
Recreational and exercise facilities	Interview-based satisfaction rating	1–10	“How satisfied are you with recreational and exercise facilities?”
Green spaces	Interview-based satisfaction rating	1–10	“How satisfied are you with green spaces in your community?”

### 3.4 Research methods and robustness testing

To ensure the robustness and interpretability of the research findings, a combination of qualitative and quantitative methods was employed. The quantitative analyses were conducted in a sequential manner, including exploratory analysis, confirmatory analysis, and robustness testing, each serving a distinct analytical purpose:

#### 3.4.1 Thematic analysis

The transcribed interview data were analyzed using thematic analysis, an iterative process in which patterns and themes are identified across the dataset. NVivo 12.0 was used to manage the data and facilitate the coding process. Themes related to subjective well-being, public open space usage, and the influence of migration status were extracted. This qualitative analysis served as an exploratory foundation for identifying key dimensions and informing subsequent quantitative analyses.

#### 3.4.2 Quantitative analysis and robustness testing

Several quantitative analyses were performed to examine the relationships between public open space attributes and subjective well-being and to assess the robustness of the findings.

(A) Exploratory Quantitative Analysis: Objective variables such as economic support, emotional support, and mental health status were analyzed quantitatively to complement the qualitative findings. Descriptive statistics were first used to summarize the characteristics of the sample population. Independent sample t-tests were then conducted as an exploratory step to examine mean differences in key variables (e.g., subjective well-being) between migrant and local older adults groups. Effect sizes (Cohen’s d) were calculated to assess the magnitude of group differences beyond statistical significance.
(B) Confirmatory Analysis: To further examine the associations between attributes of public open spaces and subjective well-being, multivariate regression analyses were conducted as confirmatory analyses, controlling for relevant individual-level variables such as values, mental health, economic support, emotional support, marital status, and pension insurance. Standardized regression coefficients were reported to facilitate the interpretation of effect sizes and to enable comparison across predictors.
(C) Robustness Testing: To test the stability and consistency of the results across groups, seemingly unrelated regression (SUR) models were employed as a robustness check. This approach allowed for the comparison of coefficient differences between migrant and local older adults groups while accounting for potential correlations across model error terms. The robustness analyses were used to assess whether the observed relationships remained consistent across analytical specifications.

Prior to conducting parametric analyses, key statistical assumptions were assessed. The distributions of major continuous variables were examined for approximate normality using skewness and kurtosis statistics as well as visual inspection. Homoscedasticity was evaluated through residual diagnostics in regression analyses. No substantial violations of parametric assumptions were detected, supporting the use of t-tests and linear regression models.

## 4 Results

### 4.1 Basic characteristics of participants

A total of 325 older adults were interviewed in this study, of which 187 local older adults have been living in Dongguan since they were born. The 138 migrant older adults were all from other provinces of the Chinese mainland, most of whom are from developing and rural areas. Table 3 shows the basic characteristics of the two groups of older adults. The gender and age of the two groups are similar. Most of them are married and aged from 60 to 100. From the perspective of older adults individuals’ values, significant differences can be observed between the two groups. Among the local older adults, 88 individuals hold cognitive-oriented values, 62 embrace emotional-oriented values, and 37 adhere to material-oriented values. In contrast, among migrant older adults individuals, 71 prioritize material-oriented values, 34 endorse cognitive-oriented values, and 33 align with emotional-oriented values.

**Table 3:** Basic characteristics of migrant older adults and local older adults.

Variabe	Local older adults (*n* = 187)	Migrant older adults (*n* = 138)
Gender		
Male	81	64
Female	106	74
Age		
60–70	73	56
71–80	107	73
81–90	3	4
91+	4	5
Marital status		
Married	125	92
Separated/divorced/widowed/unmarried	62	46
Sense of worth		
Emotional forms	62	33
Cognitive forms	88	34
Material forms	37	71
Pension		
Yes	83	46
No	104	92
Mental health		
Mean	3.83	3.70
Median	3.90	3.70
Emotional support		
Mean	1.75	1.88
Median	2.00	2.00
Financial support		
Mean	7.92	7.45
Median	7.94	7.52
Duration of migration(years)		
≤10		75
>10		63

Examining the psychological well-being of the older adults, the average psychological health score among the local older adults is higher than that of their immigrant counterparts. Additionally, in terms of emotional support received, migrant older adults individuals, on average, report higher levels of emotional support compared to their local counterparts. Out of the 138 migrant older adults individuals, 75 have been in the process of migration for less than 10 years, while the remaining have accumulated more than a decade of migration experience.

### 4.2 Self-rated subjective well-being status

Most of the participants believed that the sense of happiness was an important part of life satisfaction. In contrast, some thought that positive or negative emotions formed it. Table 4 provides a visual representation of the self-reported subjective well-being results for both local and migrant older adults groups. Notably, a substantial majority of the local older adults population, comprising 85.02%, rated their subjective well-being as high (For ‘Very Good,’ t = 1.75, *p* = 0.041; for ‘Good,’ *t* = 2.05, *p* = 0.022). Simultaneously, a remarkable 84.06% of migrant older adults individuals expressed a profound sense of happiness. Delving deeper into the subgroup of immigrant seniors who have resided in Dongguan for over a decade, 57 out of 63 reported a positive subjective well-being, underscoring a prevailing sense of contentment. However, it’s important to acknowledge the nuanced experiences within this group, as 6 individuals provided negative assessments, reflecting the diversity of emotional states among long-term immigrant seniors. In contrast, among the 75 older adults individuals living in Dongguan for less than 10 years, a subset of 16 reported negative evaluations. This suggests that the duration of residence might influence the subjective well-being perceptions of older adults individuals, introducing a temporal dimension to their emotional experiences.

**Table 4:** Self-rated subjective well-being among migrant and local older adults.

Levels of Subjective well-being	Duration of migration			Local	t-value	*p*-value	
	≤10 years	>10 years	ToTal	(*n* = 187)			Effect size (Cohen’s d)
	(*n* = 75)	(*n* = 63)	(*n* = 138)				
Very good	41	50	91(65.94%)	106 (56.68%)	1.75	0.021	0.32
Good	18	7	25(18.12%)	53 (28.34%)	2.05	0.042	0.38
Fair	11	4	15 (10.87%)	12 (6.42%)	1.15	0.013	0.21
Poor	5	2	7 (5.07%)	16 (8.56%)	0.89	0.376	0.15

Independent sample *t*-tests were conducted to compare self-rated subjective well-being scores between migrant older adults and local older adults. Although subjective well-being levels are presented as categorical distributions, the statistical tests were based on the underlying continuous subjective well-being scores. Cohen’s d was reported to indicate the magnitude of group differences, with values of approximately 0.20, 0.50, and 0.80 representing small, medium, and large effects, respectively.

While the overall self-assessed subjective well-being level appears relatively high among older adults, it is intriguing to note the disparity between the local and migrant older adults. The local older adults exhibited a more positive self-assessment compared to their migrant counterparts, shedding light on potential cultural, social, or environmental factors influencing their perceived well-being. This insight into the diverse subjective experiences of older adults populations adds depth to our understanding of the complex interplay between happiness, life satisfaction, and the context in which individuals age.

### 4.3 The influential attributes of public open spaces to subjective well-being

To analyze the data collected, we adopted reflexive thematic analysis, a flexible and iterative approach that allowed us to identify patterns and themes related to the subjective well-being of both local and migrant older adults. The thematic analysis followed the six-step process proposed by Braun and Clarke (104), which includes:

Familiarization with the data: All interviews were transcribed verbatim, and team members immersed themselves in the data by reading the transcripts multiple times.
Generating initial codes: We systematically coded the data, using NVivo 12.0 software to organize responses according to recurring ideas related to psychological health, emotional support, and value orientations. Codes such as “emotional support,” “cognitive well-being,” and “material well-being” emerged during this stage.
Searching for themes: After coding the data, we collated similar codes into broader themes. For instance, codes related to value orientations (cognitive, emotional, and material) were grouped under the theme “Older Adults Value Systems.”
Reviewing themes: At this stage, the research team met to review and refine the themes to ensure they accurately reflected the data. We engaged in triangulation, with each team member independently analyzing a portion of the data to ensure consistency. Any discrepancies in theme identification were discussed until a consensus was reached.
Defining and naming themes: We further refined the themes to ensure they were distinctive and meaningful. For example, the theme “Emotional Support” was divided into subthemes like “family support” and “neighborhood support” to reflect the specific sources of support.
Writing the report: The final themes were integrated into the findings section, providing a coherent narrative of the data.
Involvement of Research Team and Triangulation: Our analysis involved four team members, all of whom have expertise in qualitative research and aging studies. Each member analyzed a subset of the data independently to avoid bias, and triangulation was employed to ensure the reliability of the coding and theme generation. We used multiple rounds of cross-checking among the team to refine the final themes.

All responses to influential attributes of subjective well-being are processed. According to the thematic analysis, the attributes can be divided into two scales: city and neighborhood. The city scale refers to macro-level urban infrastructure and environmental conditions that affect older adults across the entire urban territory of Dongguan, including city-wide public transportation systems, green space distribution, technical infrastructure (e.g., mobile payments), and public safety governance. These factors reflect the broader urban policy and planning framework within which all communities operate. These city-wide facilities and spaces have a broad impact on the quality of life for the older adults, determining how they socialize, participate in activities, and receive social support in the overall urban environment. On the other hand, the neighborhood scale, by contrast, focuses on the localized living environment of older adults, specifically referring to residential neighborhoods typically within a 1–2 km radius of a participant’s home. This includes pedestrian pathways, building entrances, corridors, lifts, and recreational/exercise facilities within walking distance, as well as green spaces embedded in the residential compound or nearby. These facilities are more closely connected to the daily lives of the older adults, directly influencing their social activities, neighborhood interactions, and the support they receive in their day-to-day lives.

#### 4.3.1 City scale

Considering urban spaces, all migrant older adults thought that the urban environment in Dongguan was better than their original residency. The air was fresh and public spaces were clean and in order. The majority of local older adults have the same opinion. However, they sometimes hope that the city can take more care of the actual situation of the older adults and consider the social needs of the older adults more in urban planning.

*“I am content with our living environment. Our place is impeccably clean, and overall, the city offers excellent amenities. The surroundings are well-maintained, and the air quality is commendable… The place we reside in specifically enjoys good air quality, contributing to our overall satisfaction with our living conditions.” (Local older adults)*

*“Satisfied with the life here; it’s very good. I don’t worry about it, as public security is robust, and the environment is satisfactory. It’s not like when we first came … there’s been noticeable improvement in society, and for that, I am grateful.” (Migrant older adults)*

In terms of urban infrastructure, migrant and local elders had similar views. Most of the older adults expressed satisfaction with urban infrastructure, particularly public transportation, but at the same time, expressed difficulties in adapting to the development of industrialization. Some older adults lack the skills for computers and mobile phones, and they do not know how to use mobile applications to make payments but prefer to pay in cash. This impedes their integration into an industrialized society. The development of technical infrastructure has created a social segregation between old and young, which may bring new issues of social inequality.

*“It’s very convenient to travel in the city. I usually buy vegetables in the supermarkets near the neighborhood. Now everything is very good, and I have everything. I can buy everything around, like medicine and breakfast.” (Local older adults)*

*“Old people don’t know how to use mobile phones. It is very convenient for young people. You don’t need to bring money to buy things. You can also take a loan online. But this is the biggest problem for us old people. We are not used to it; we are old.” (Local older adults)*

*“I don’t know how to use WeChat to make payment. Others may deceive me. Many people are deceived with mobile phones…” (Local older adults)*

*“I think it is very convenient. The necessary infrastructure has been provided, which fully meets my needs.” (Migrant older adults)*

*“Everything is good now, but just the computer and mobile phone. I do not know how to use the computer or the mobile phone…so it’s very inconvenient.” (Migrant older adults)*

#### 4.3.2 Neighborhood-scale

The mixed-use of neighborhood roads by vehicles and pedestrians has led to safety and mobility issues. Some older adults pointed out problems of walking safety in pedestrian spaces. They expressed concerns about the vehicles in pedestrian lanes as a possible threat to their walking safety. Such concerns, including fast movement and improper parking of vehicles, were addressed frequently. Moreover, the quality of pedestrian spaces is influential on walking mobility. Sufficient and well-managed pedestrian spaces ensure older adults walk easily. But the walkways often occupied by vehicles affected the mobility of older adults to some degree. The construction of parking lots in some communities is still backward and cannot satisfy many private vehicles; that is why some older adults have negative views of their walking mobility.

*“Traffic congestion is a constant issue here, with cars of all sizes and electric vehicles crowding the streets. It can be quite bothersome, especially considering the speed at which some of them move. This poses a safety concern for us older individuals, as the fast-paced traffic increases the risk of accidents, something we cannot afford at our age.” (Migrant older adults)*

*“I recently witnessed someone charging their electric car without permission in the lane. The lanes are cluttered with wires, posing a significant safety hazard.” (Local older adults)*

*“There are so many cars, and they don’t park properly. They just park in the open space. Sometimes they block the way out, and you have to walk a little sideways to get out.” (Migrant older adults)*

*“The alley is so narrow that the cars keep parking there even if they can’t get in, making it impossible for everyone to walk.” (Local older adults)*

Most of the older adults expressed their satisfaction with the key public spaces in the buildings – the entrances, corridors, and lifts, considering that these architectural spaces can meet their daily needs because the buildings they are living in had experienced aging-oriented transformations in public spaces to be more adaptive to older adults. However, some migrant older adults had different opinions, expecting the space of building entrances and corridors can be refurbished and well-managed in order to be more age-friendly.

*“The corridor has been refurbished, probably in 2017. It is much better now than before. Before, the corridor lighting was bad, and my eyesight was not good; my walking ability was not good, so I had to walk slowly.” (Local older adults)*

*“The building entrance is too narrow. It was much more open before. But now there are some messy things parked here, which makes it difficult to go up and down the stairs.” (Migrant older adults)*

All the older adults expressed their approval of regular lift repairs. For the older adults, their physical condition is subject to a certain degree of objective constraints. Barrier-free walking spaces are important for older adults. The installation of lists and regular repairs have played a role in increasing older adults’ physical and social activities.

*“There was no lift in the past. Although I lived on the third floor, I was still young at that time… Now that there is a lift, I can go downstairs and walk more and get more sunshine. It’s good.” (Local older adults)*

*“I haven’t used a lift before, and it’s quite convenient. I didn’t need this thing when I lived on the ground floor. Now there is a lift, and someone often comes to repair it every some time, so we all feel secure…and we use it to get downstairs every day” (Migrant older adults)*

Regarding public facilities, there is a slight difference between migrant and local residents, though living in the same area, in terms of recreational and exercise facilities. All local older adults held the idea that the recreational and exercise facilities were sufficient within the neighborhood, including fitness equipment, social places, entertainment equipment, and so on. These recreational and exercise facilities have effectively relieved their stress and prevented loneliness. Among the migrant older adults, the majority of them also expressed their enthusiasm for using recreational and exercise facilities. But it is worth noting that most migrant older adults are interested in entertainment and exercise facilities only in words, but not in practice, which may increase their sense of loneliness.

*“Exercise equipment for older people has been installed a long time ago. Sometimes I go downstairs to exercise with the fitness equipment. It’s quite good. It’s good to exercise, and there is no need for particularly strenuous exercise.” (Local older adults)*

*“I usually play mahjong, and sometimes I can play mahjong for a long time. It’s just for entertainment and to pass the time. I just sit here and play mahjong when I have nothing to do. While playing and chatting, I feel good and happy.” (Local older adults)*

*“I do exercises when the neighbors I know call me to exercise together in the outdoor space within the neighborhood. The relationship between the neighbors is good.” (Migrant older adults)*

*“I used to play mahjong when I had nothing to do in my hometown. Now I don’t know anyone here. I want to play mahjong but don’t know where to find someone. I have to stay at home every day. It’s boring.” (Migrant older adults)*

*“Many old men and old ladies are dancing in the square every night…I really want to go, but I don’t know anyone here. Forget it, I can exercise casually at home.” (Migrant older adults)*

Green space is also an important factor for subjective well-being. The majority of the older adults were satisfied with the green spaces between the residential buildings, in terms of the area of the green space. But meanwhile, a small number of local older adults expressed dissatisfaction with the wanton parking of various private cars in the neighborhood’s green spaces.

*“Well, you can say that it is much better than before. The trees planted have grown up, and the air feels fresher than before. I used to feel that the neighborhood was groggy.” (Local older adults)*

*“It’s pretty good now, but the place that was originally open to people is now full of all kinds of cars. If you want to go downstairs to enjoy the sun, it’s blocked. I wish this could be changed.” (Local older adults)*

### 4.4 Robustness check

To mitigate potential biases such as social desirability, recall bias, or emotional influences in the self-reported subjective well-being of the older adults, this study drew inspiration from the subjective well-being scale proposed by Chen (74) and Wang et al. (75). Through semi-structured interviews and relevant questions, the study measured the subjective well-being of different older adults individuals. The resulting subjective well-being scores were treated as continuous outcome variables in the subsequent quantitative analyses. Simultaneously, within the ten coding dimensions of public open spaces, older adults participants were asked to rate their satisfaction with each dimension on a scale from 1 to 10, where higher scores indicated higher satisfaction. These satisfaction ratings were operationalized as continuous independent variables and directly incorporated into the regression models to quantify the effects of different public open space attributes. To enhance the reliability and robustness of the results, variables that may influence the subjective well-being of the older adults—such as values (sense of worth), mental health status, economic support, emotional support, marital status, and pension insurance—were included as control variables. All models were estimated separately for migrant and local older adults groups to examine group-specific associations between public open space attributes and subjective well-being. To further assess the robustness of the findings and to formally test between-group differences, seemingly unrelated regression (SUR) models were employed. This approach allows for correlated error structures across group-specific equations and enables statistical comparison of coefficients between migrant and local older adults groups. The SUR-based tests therefore provide a robustness check on whether observed differences are statistically meaningful rather than driven by sampling variation.

The study found that public security and order, environmental cleanliness, public transportation, technical infrastructure, walking safety, walking mobility, entrances and corridors, lift installation and management, recreational and exercise facilities, and green spaces significantly influenced the subjective well-being of both local and migrant older adults individuals (Table 5). It is noteworthy that the impact of public open spaces on the subjective well-being of local and migrant older adults individuals differs. Specifically, public security and order, environmental cleanliness, walking mobility, and lift installation and management showed no significant differences in their impact on the subjective well-being of local and migrant older adults individuals. Public transportation, technical infrastructure, and walking safety significantly affected the subjective psychological health of migrant older adults individuals more than local older adults individuals. Entrances and corridors, recreational and exercise facilities, and green spaces significantly influenced the subjective well-being of local older adults individuals more than migrant older adults individuals. These group-specific patterns are consistent with and further substantiate the qualitative findings, indicating that differences in social integration, daily activity patterns, and familiarity with neighborhood environments shape how public open spaces affect well-being. In addition, the analysis examined the role of migration duration among migrant older adults individuals, revealing that a longer duration of residence in Dongguan was positively associated with subjective well-being, providing further evidence for the stability and internal consistency of the results.

**Table 5:** Robustness test regression analysis and between-group difference test results.

Variable	(1) Local older adults people	(2) Migrant older adults	(3) Difference in coefficients between groups (P)	(4) Migrant older adults
	Subjective well-being	Subjective well-being		Subjective well-being
Public security and order	0.001*	0.009*	0.490	Control
Environmental cleanliness	0.011**	0.004**	0.205	Control
Public transportation	0.010*	0.014*	0.030	Control
Technical infrastructure	0.006*	0.007*	0.087	Control
Walking safety	0.011*	0.021^*^	0.004	Control
Walking mobility	0.005	0.002	0.800	Control
Entrances and corridors	0.013^*^	0.007*	0.004	Control
Lift installation and management	0.004*	0.005*	0.913	Control
Recreational and exercise facilities	0.016**	0.002*	0.028	Control
Green spaces	0.012*	0.006*	0.034	Control
Financial support	Control	Control		Control
Emotional support	Control	Control		Control
Mental health	Control	Control		Control
Marital status	Control	Control		Control
Pension insurance or not	Control	Control		Control
Sense of worth	Control	Control		Control
Length of immigration	No	No		0.009**
*N*	186.000	138.000		138.000
*R* ^2^	0.775	0.772		0.771
Adj. *R*^2^	0.754	0.742		0.738

^*^p < 0.1; ^**^p < 0.05; ^***^p < 0.01.

## 5 Discussion

### 5.1 Overall patterns of subjective well-being among migrant and local older adults

This paper investigates the subjective well-being of migrants and local older adults living in Dongguan City, one of the most rapidly industrialized regions in China. The study examines the impact of public open spaces on subjective well-being, a key measure of an individual’s self-perceived happiness based on the balance between positive and negative emotions. Both groups of older adults exhibited a similar balance between positive and negative emotions, a finding consistent with research conducted in other rapidly urbanizing regions (75, 81, 82). Table 6 illustrates the emotional responses of older adults to various influential attributes of public open spaces.

**Table 6:** Emotional responses to the influential attributes of public open spaces.

Categories	Codes	Emotional responses	
		Migrant older adults	Local older adults
Urban spaces	Public security and order	+	+
	Environmental cleanliness	+	+
Urban infrastructure	Public transportation	+	+
	Technical infrastructure	−	−
Pedestrian spaces	Walking safety	+/−	+/−
	Walking mobility	+/−	+/−
Architectural spaces	Entrances and corridors	+/−	+
	Lift installation and management	+	+
Public facilities	Recreational and exercise facilities	−	+
	Green spaces	+	+/−

+Positive emotion; −Negative emotion; +/−Both emotions.

One of the key findings of this study is that the sense of happiness among the older adults is influenced by a range of factors, from city-wide infrastructure to the neighborhood level. Regarding public security and order, environmental cleanliness, and lift installation and management, both migrant and local older adults expressed highly positive attitudes. These findings suggest that previous transformations aimed at making urban infrastructure more elder-friendly have effectively enhanced the quality of life for both groups. Such positive outcomes echo the results of other studies conducted in urban centers globally, where infrastructure improvements, particularly those aimed at enhancing security and cleanliness, have had a notable impact on older adults’ well-being (83). However, it is essential to recognize that this study was conducted in Dongguan, a city with a uniquely high proportion of migrant populations, which may have influenced the results. The high proportion of migrant older adults, as discussed in previous research, tends to bring additional challenges regarding social adaptation and access to neighborhood services, which may differentiate this study from those conducted in less migrant-populated cities.

### 5.2 Urban infrastructure, transportation, and the digital divide

Despite the overall positive reception of public security and infrastructure, this study reveals that both migrant and local older adults responded positively to public transportation, indicating that urban transportation services are well-planned and managed, meeting their everyday needs. The positive responses to public transportation imply that older adults, regardless of their migratory status, have largely adapted to modern urban life. This finding aligns with studies conducted in other urban centers that emphasize the role of accessible and efficient public transportation in improving the quality of life for older adults (84). However, the negative responses to technical infrastructure manifest a widespread challenge for older adults individuals globally. In this study, both groups of older adults reported difficulties operating mobile phones and making payments when shopping in the city. This reflects a broader incompatibility between the older adults and the growing dependence on digital technologies in everyday life. Previous studies have consistently highlighted the difficulty older adults individuals face in adapting to new technologies, a challenge that can lead to reduced social engagement, loneliness, and isolation (104).

This digital divide is further emphasized by the fact that many older adults individuals, both migrant and local, struggle to navigate the digital platforms that are increasingly integrated into urban living. The inability to use essential services such as mobile payment platforms not only limits their autonomy but can also deepen feelings of social isolation. This finding underscores the need for more inclusive urban designs that take into account the technological capabilities of the older adults, particularly in a rapidly digitizing society like China’s. Further studies could explore how specific technological training programs or the design of more older adults-friendly technologies could mitigate these issues.

### 5.3 Walking safety, mobility, and conflicts in pedestrian spaces

When it comes to walking safety and mobility, the opinions of the older adults were more divided. While half of the respondents expressed positive views on pedestrian spaces, the other half highlighted concerns about conflicts between pedestrians and vehicles. The rapid growth of private car ownership, which is one of the defining features of China’s economic development and industrialization, has created a new set of challenges for older adults. Many older adults feel unsafe in environments where pedestrians and vehicles share space, and these concerns are exacerbated by their declining physical capacities. This finding is consistent with global research indicating that older adults are particularly vulnerable to mobility issues in densely populated, car-centric urban environments (105). The negative impact of increasing vehicle ownership on the older adult’s use of public spaces is further reflected in their dissatisfaction with the random parking of private cars in neighborhood green spaces. Such encroachments not only limit the physical space available for walking and social interaction but also pose safety risks, further discouraging older adults individuals from engaging in outdoor activities. This contradiction between modern industrialized life and the traditional urban layout is a challenge that many rapidly urbanizing cities must address to ensure the well-being of their older adults populations.

### 5.4 Recreational facilities, social participation, and migrant–local differences

A particularly notable finding of this study is the slight difference in the use of recreational and exercise facilities between migrant and local older adults. In general, local older adults reported more frequent use of recreational and exercise facilities than migrant older adults. This difference may be attributed to the stronger social ties and deeper integration of local older adults within their communities. Social participation is a critical factor that influences the subjective well-being of the older adults, and it can either positively or negatively impact their quality of life (106). Migrant older adults, who often have weaker social networks, are less likely to engage in recreational and physical activities, which in turn limits their opportunities for social participation. This lack of social participation among migrant older adults can lead to a loss of social relationships, which exacerbates feelings of isolation and loneliness. Previous research has shown that migrant older adults who participate less in social activities are more prone to depression and a diminished sense of belonging (107, 108). In contrast, local older adults, who have long been rooted in their communities, are more likely to use recreational and exercise facilities regularly. These facilities provide them with opportunities to engage with their peers, relieve stress, and enhance their overall well-being. The difference in social participation between the two groups highlights the importance of fostering social inclusion for migrant older adults, ensuring that they too have access to neighborhood resources and social networks that can improve their subjective well-being.

### 5.5 Integration of qualitative and quantitative findings

This study employed a mixed-methods approach in which qualitative and quantitative findings were analytically connected. Insights from the qualitative interviews informed both the construction of quantitative variables and the interpretation of statistical results, allowing for triangulation across methods. Qualitative thematic analysis identified key attributes of public open spaces that older adults repeatedly associated with subjective well-being, including public security and order, environmental cleanliness, walking safety and mobility, technical infrastructure, and access to recreational and exercise facilities. These themes were subsequently operationalized as core dimensions in the quantitative models, ensuring that the statistical analysis reflected older adults’ lived experiences.

The quantitative results further clarified how these qualitatively identified factors operated across groups. For example, both migrant and local older adults emphasized the importance of public security and environmental cleanliness in interviews, and the regression results showed similar effects for these attributes across groups, suggesting shared baseline conditions for well-being. In contrast, qualitative accounts revealed differences in social participation and familiarity with neighborhood environments, particularly regarding recreational and exercise facilities. The quantitative analysis supported this pattern by showing a stronger association between these facilities and subjective well-being among local older adults than among migrant older adults. In addition, qualitative narratives describing difficulties with digital technologies among older adults helped contextualize the quantitative finding that technical infrastructure was more strongly associated with subjective well-being among migrant older adults. Together, these integrated findings provide a more nuanced understanding of migrant–local differences than either qualitative or quantitative evidence alone.

### 5.6 Strengths, limitations, and broader implications

The strengths of this study lie in its focus on a rapidly industrializing urban center with a high proportion of migrant older adults. This context allows for a unique exploration of how public open spaces and urban infrastructure impact the well-being of both local and migrant older adults. However, one limitation of this study is the reliance on self-reported data, which may be influenced by personal biases or recall inaccuracies. Additionally, while the study provides valuable insights into the subjective well-being of older adults individuals in Dongguan, further research is needed to compare these findings with those from other urban centers with different migratory dynamics and infrastructure development levels. In conclusion, this study contributes to the existing body of knowledge by shedding light on the specific challenges and opportunities faced by older adults individuals in rapidly urbanizing cities like Dongguan. The findings emphasize the importance of accessible public open spaces, well-planned urban infrastructure, and social inclusion in enhancing the subjective well-being of both migrant and local older adults.

## 6 Conclusions and implications

### 6.1 Conclusion

This study, conducted in Dongguan, a highly industrialized and migrant-dense city in China, provides valuable insights into the subjective well-being of both migrant and local older adults populations and the role that public open spaces play in influencing their well-being within this specific urban context. The findings reveal that local older adults tend to rate their subjective well-being more favorably than their migrant counterparts. However, both groups of older adults generally express satisfaction with the public open spaces they inhabit, particularly appreciating the aging-oriented environmental transformations in their communities. These findings should be understood in light of Dongguan’s distinctive socioeconomic and demographic characteristics, including its advanced industrialization, high population mobility, and large proportion of migrant residents. Public open spaces in this context serve not only as physical areas but also as pivotal environments that foster social engagement, physical activity, and emotional connections to one’s living space. These spaces play an important role in strengthening older adults’ sense of attachment, belonging, and well-being in rapidly urbanizing, migrant-intensive cities. The study highlights the importance of considering both the city scale and neighborhood scale in the design and planning of public spaces, acknowledging their role in supporting the well-being of older adults populations in cities undergoing similar industrial and demographic transformations. However, despite the positive impact of these transformations, several challenges remain in making urban spaces truly age-friendly. The aging-oriented modifications face constraints due to existing infrastructure limitations, budgetary concerns, and resource allocation complexities that may vary across different urban contexts. Policymakers must therefore consider the long-term financial sustainability of such transformations, ensuring that the benefits of age-friendly initiatives justify their cost. This requires careful assessment of cost-effectiveness, including measurable outcomes such as improved well-being, increased social participation, and reduced health risks among the older adults. Moreover, achieving broad public acceptance for these transformations demands active neighborhood engagement, acknowledging the diverse cultural preferences and sensitivities of different populations within specific local settings. Accessibility remains another key challenge, with physical and technological barriers—such as difficulties in navigating digital services—hindering full inclusivity for the older adults. While the insights from this study may offer useful reference points for other highly industrialized and migrant-receiving cities, their transferability should be approached with caution, and adaptations should be made in accordance with local socioeconomic conditions, governance structures, and population compositions. Strategic planning, continuous monitoring, and ongoing evaluation will be necessary to sustain the impact of aging-oriented improvements over time in comparable urban settings.

### 6.2 Strategic recommendations

Based on the research findings, several strategic recommendations can be made to guide future aging-oriented environmental design and transformation efforts, particularly in response to the rapid aging of the population:

1. City Scale Initiatives: Authorities should focus on upgrading urban infrastructure to better accommodate the needs of the older adults, particularly in areas such as public transportation, digital payment systems, and public information services. The results show that while public security and environmental cleanliness are universally valued by both migrant and local older adults, difficulties in using digital and IT systems remain a prominent barrier to daily life, especially for older adults with limited technological literacy. To address this challenge, cities could introduce age-friendly digital support measures, such as on-site assistance counters, digital literacy kiosks in public open spaces (e.g., parks and neighborhood squares), and simplified interfaces for essential public services, thereby reducing the digital divide and supporting older adults’ independent participation in urban life.
2. Neighborhood scale Improvements: At the neighborhood level, there should be continued efforts to optimize public open spaces, with a focus on increasing green areas and enhancing recreational and exercise facilities. Given the study’s findings that safety, cleanliness, and walking mobility significantly influence subjective well-being, neighborhood-level interventions should prioritize pedestrian safety, clear separation of vehicles and walkways, and the maintenance of clean and orderly environments. Creating environments that promote both social interaction and physical activity is essential for boosting the overall well-being of older adults. Design elements such as barrier-free pathways, adequate lighting, shaded seating, and clearly marked activity zones can further enhance the usability and inclusiveness of public open spaces, particularly for older adults individuals with mobility limitations.
3. Addressing the Needs of Migrant Older Adults: Special attention should be paid to the unique challenges faced by migrant older adults. Authorities should conduct comprehensive social surveys to better understand their living conditions, emotional needs, and social integration challenges. The findings indicate that migrant older adults are less likely to actively use recreational and social facilities despite expressing interest, suggesting barriers related to social familiarity rather than physical access alone. Targeted interventions—such as community orientation programs, facilitated group activities, and neighborhood-based social connectors in public open spaces—can help migrant older adults build social networks, reduce isolation, and improve overall happiness and life satisfaction.

By proactively addressing these challenges and adopting a holistic approach to public space transformation, urban planners and policymakers can create environments that better support the aging population’s well-being. This study highlights the importance of taking a dual-scale approach—at both the city and neighborhood levels—ensuring that urban spaces are not only functional but also enhance the quality of life for older adults residents, particularly in rapidly urbanizing areas like Dongguan.

## 7 Limitations of the study

Despite the valuable findings of this research, several limitations should be acknowledged. First, although we supplemented the analysis with a quantitative examination of the relationship between the attributes of public open spaces and the subjective well-being of older adults, the study primarily focused on identifying positive influencing factors and general patterns. As a result, negative emotions or adverse experiences may be underrepresented, potentially limiting a more balanced understanding of older adults well-being. Second, this study is geographically focused on a single city—Dongguan, a rapidly urbanizing and highly industrialized city with a large migrant population. While this context provides a valuable and relevant case for examining migrant–local differences, the findings may not be directly generalizable to cities with different socioeconomic structures, population compositions, or stages of urban development. Future research could adopt comparative multi-city designs to examine whether similar patterns emerge across different urban contexts. Third, while this study explored the relationship between public open spaces and subjective well-being, it did not capture the long-term or dynamic effects of environmental changes over time. Longitudinal studies would be particularly valuable for assessing how sustained improvements in public open spaces influence older adults well-being and social integration in the long run.

An additional limitation of this study relates to the sampling strategy. Participants were recruited using a snowball sampling approach, which was adopted due to the lack of a comprehensive sampling frame for older adults migrants and the practical difficulty of accessing this population. While snowball sampling is effective for reaching hard-to-reach groups, it may introduce selection bias, as participants are more likely to refer individuals within their own social networks. Consequently, older adults individuals who are more socially active, healthier, or more engaged with public open spaces may be overrepresented, whereas those who are socially isolated, less mobile, or less connected to community life may be underrepresented. To mitigate this limitation, multiple initial “seed” participants were purposively selected across different neighborhoods and demographic backgrounds, and multiple independent referral chains were initiated. Nevertheless, the sample does not constitute a probability-based or statistically representative sample of the entire older adults population in Dongguan. In particular, although the proportion of migrant and local older adults in the sample reflects Dongguan’s broader demographic context as a city with a high migrant population, it does not exactly mirror the city’s population structure. As a result, the findings should be interpreted with caution in terms of external validity. The results are best understood as context-specific insights into the lived experiences of migrant and local older adults in a highly industrialized urban setting, rather than as universally generalizable conclusions. Future research could strengthen external validity by employing probability-based sampling methods, mixed sampling strategies, or comparative multi-city and longitudinal designs.

## Data Availability

The original contributions presented in the study are included in the article/Supplementary material, further inquiries can be directed to the corresponding author/s.
