# Supplementary material for: Public open spaces and well-being: a comparative study of migrant and local older adults in Dongguan
Source: Front Public Health. 2026 Mar 12;14:1776572. doi: 10.3389/fpubh.2026.1776572 (PMC13020563; doi:10.3389/fpubh.2026.1776572)
Supplement: Supplementary file 1 [file Data_Sheet_1.docx]

Interview records

Q: How old are you aged now?

A: I am 67 years old this year.

Q: How are you doing?

A: At the age of 60, my blood pressure was high and I took a lot of antihypertensive drugs.

Q: How many sons do you have?

A: Three sons. Three fields a person, ah, that field is very few, my husband brought out Dongguan here a lot of land to Dongguan ah, farming ah, less high high male fish grain ah grain ah, so. I have very little farming here.

Q: How long have you been here since then?

Answer: ah, there have been more than 40 years, more than 40 years ago, divide the land to the household came. Because the three fields of my land at home are the fields of four people. You don't even cook enough food. That three meals rotten and no money to earn, farming harvest that hundreds of jin, the rice is not enough to eat ran out.

Q: That means you and your husband came here 40 years ago. Why are you getting a Yangjiang hukou? Is your hukou from Yangjiang or here?
 Answer: my registered permanent residence is still in my hometown not to come over, do not come over.

Q: But if you said it here for 40 years, must your children have been born here?

Answer: ah, I still live here ah, buy the land is a little building ah,

Ask: oh, oneself can still buy, you are not a local person can also buy a building ah.
 Answer: before have land to sell ah, now you have money all useless, have no land to sell. Well, my three sons all came to Dongguan and were born.softly chant,

Q: Your three sons.

A: Well.

Ask: ah, that you this is still very fierce, good fierce ah, very early come over, before many years ago to come to farm here to have no money to earn.
 A: Well. Well. I just came here to help interplant rice seedlings. Three yuan a day, my two in-laws took a son, the son saw my two in-laws to plant rice seedlings. At 6:00, I took the son back to cook. Three dollars a day for now.

Q: the former local worker, do you think you are satisfied with your own living situation? How do you think you're doing it. Do you feel still happy?

A: Own. Also on the general, also one.

Q: Do you think you have a family relationship with your family, those with the children, their relationship with the husband.
 Answer: well, ok, then my three sons are also very good to me, oh, well, my husband is also very good to me, and a little body uncomfortable ah.

Q: Well, how do you look at it. I take you to see again, that you are because also very filial piety? Because I do not read, and not their own where all have a husband to take, I am very stupid. Is I before ah originally small sister at that time to practice, practice got my I married, to give birth to a child, there is no vitality to practice, no? The old man helped me take up, I was a child, all by myself, I brought up,

Q: Do you are still satisfied with the whole living environment? What do you think of the living environment? Are you still satisfied with the environment you live in?

A: Well, I live in a house that I built by myself. Well. I feel the surrounding environment, my own building, the building.

Q: But you think you're with neighbors or with you, you're satisfied with your whole environment. Is you next to the house you live in, including this what a lot of very polluted ah, the air is good? Air air can not cough up.
 Answer: The air is not good high or low. I'm just that and I'll say you go to that hundred stops there. Oh, tall and cold.

Q: Oh. Well. Understand, understand. Do you usually walk around with people like you, chat, walk and dance.

A: I don't play cards.
 Q: What entertainment do you usually have?

Answer: very good that that aunt, she asks me I all can't what? I'm just get close to talk, play, joke. Ah, so oh play with the phone, open a video, talk about that happy joke.softly chant. Their own entertainment or compare that is right? Well. Is there anything to play with your neighbors or with your fellow countrymen. Ah, someone ah, feed I call me to play oh, the neighbor relationship is ok ah.

Q: Ok, then you think you are most satisfied with what you are most satisfied with in any aspect of your life. The most satisfied with is which part is the most satisfied with the most happy?

Answer: I am um son grew up to be satisfied, son, son grew up, the wife married back to grow all have, I am satisfied, satisfied with happy ah, I am my granddaughter big um 21 years old, that granddaughter's granddaughter is 21 years old. Well, wow, I graduated from college.

Ask: college graduation, you look you still very young, very healthy, you say you are too young, immediately all the fourth generation, very happy die. That is to say, these children in the family grow up, grow up, you see them grow up you are the most happy, he is the most happy beginning.

Answer: I for my life is for two in-laws, well, no old man to help, I well his father and help not to help me, ah, and no family public and no family husband to help, my two in-laws ah climb up. Well, now I have bought the land and made a building to live in, ah, the son is going to marry a wife, the daughter is married out again, also passed by. I now have nothing do not that what do not rely on him, I have been happy for a long time. Well, well, happy, well, have a happy day. Good, good, day by day, day by day, people will get old. Well, I won't be able to get up by then. I'm not very healthy.

Q: Well, overall, you think you're still happy right now.

A: The happy feeling of happiness still looks very happy. Well, now the two little daughters-in-law have a son, well, well, one is just three years old, and the youngest is three years old. Well, he and his daughter-in-law do not work, and don't need me to take ah, I will work much harder than me. Ah, mom, you used to do so hard ah, I now I don't work, I take my son ah, waiting for you to enjoy a little bit. So, he said so, I will be happy, happy, happy, at that time is not a big market, and to take grandchildren, and love to cook. So I am now two in-laws to eat, I am not as good as that daughter-in-law son and son daughter-in-law is not usually not to eat together ah. Sometimes, sometimes people come back, Gigimu, they eat. My son comes back from buying food, for example, my mother, where I come here to eat. Well, take you to dinner. This business ah, the grandson's birthday ah, ah, is the birthday of our two in-laws, we will buy food back to cook rice to eat.

Q: How are you happier, that is, if there is anything in the community that you can do for you? Well, just think about the government, and the government will do something for you. Do something for the elderly. Make you all even happier.

Answer: Very satisfied, have no opinion.

Q: How old are you aged now?

A: 81 years old.

Q: Are you a local person?

Answer: My hometown is in Chaoshan. I visited Shenzhen 6 years ago, and now I live in Dongguan. He had three sons and a daughter, and the sons did business, so they moved over with him.

Q: You look so healthy.

A: Yes, I always get sick,

Q: But you look very energetic. What are the main diseases?

A: This heart disease. Heart at first the body is still very good, 69 years old that year car accident ah, originally I was often often have exercise ah, this his foot was broken, just stay at home for a year. I started having a bad heart at the end of my 70s. Well, the heart is not good. I keep taking that heart medicine. Well. Just eat about four or five years, four or five years later began that stomach is bad, gastric ulcer, slowly chronic gastritis erosion. After this heart also this to this foot influence, began the heart is not good. The heart is not good to take medicine for a long time, the stomach is bad.be not allowed. Now you can't even take that cerebrovascular, nor is the arteriosclerosis.

Ask: oh, this way, that this is really multiple serious diseases, that you these diseases will usually affect your life a lot, is not you usually affect your own life, or mood ah, family relations, old body health can body body is not healthy trouble. Well, then you think you now the whole of your own life, because these diseases will be your life is generally satisfied?

A: Generally speaking, it is still ok, right?

Q: that you and the children their family relationship, and neighbor relationship these all very good?

A: Generally,

Q: Not so much, right? How you have your children. How many children are there?

A: Three boys and a girl.

Q: Oh, four children. Are you a local person or a nonlocal person?

Answer: I chaoshan come over, how long did you come over? I started working out in Shenzhen when I was 50.
 Q: I came here in 1997. I have been here for a long time. There are still more than ten days, more than 20 years away.

Q: Well. Oh, if you come here, will you come here for your own business, or.

A: My son did a little business.

Q: So you came with your son, so you came with you?

A: Well.

Q: Oh, so, that's like most of the family population is here,

A: Yes, son, what daughter-in-law, grandchildren, and these are all here.

Q: What do you think is the happiest thing in your life now is what you are most satisfied with. What are you most satisfied with in your whole state of life?

Answer: satisfaction is this has children to have a family, have grandson. That grandson even graduated from college and came out to work.

Q: Generally speaking, I am quite satisfied with my own feelings and my life. Still satisfied? Or not how satisfied?

A: Satisfied.
 Q: Do you think you are living with your children or not right now?

A: Live together.

Q: That means that you think you live now and you say you live with your children, right?

A: Yes.

Q: If you live together, do you think it is quite different to live together with the young people? Or do you prefer to live alone or prefer to live with your children to answer: ah. No, no, then you are all the young people are very filial piety.

Q: Oh, that's good, because many young people are not used to living with the old people. Generally speaking, you feel very comfortable living with them, don't they?

Answer: That is still very filial piety.

Q: Are you usually sick, they will accompany you to see a doctor. Will come with you, won't you? For example, what you usually take medicine, they will help you to pay attention to ah or how

A: Yes, yes.

Q: Ok, that means you think you mean you are generally satisfied, but do you really think about it? Well, from the community, or the government, or from the community or other aspects, you can make your life a little better and happier. Do you have any good advice? What can we do? From the community, ah, or from this medical staff, ah, or from the government, ah, what do you think we can do better. It can improve your satisfaction, improve your satisfaction, improve your life happiness, then you are happier.

Answer: that generally basically in that community now, that service also generally still can ah.

Q: Generally speaking, do you think this kind of service in their community is ok in all aspects. Does the government have any care or special care for your elderly people?

A: Yes, yes, yes, yes,

Q: What are the benefits.

A: For example, it was in the countryside, ah ah, I live at the age of MAO Zedong era, it was very hard at that time. Yes, yes, that substance is very good, and you will get bored. Yes, that's different now. Since I have been in the countryside since I was 65 years ago, then the government has taken care of the old man. At first, it is 50 yuan, 50 yuan a month after 55 yuan, slowly lift, slowly lift, until now there are more than 200 points.

Q: Every month, right?

A: Yes

Q: every month you although you this number in the outside this situation is that proportion is very small, right? But you have so much money so much old people calculate that amount is very powerful. Then you quite understand the government.

A: Well, yes.

Q: Do they give you, for example, the usual care is if the community pays you if the government gives you some other benefits. Ask you how well you are. That means to care about you or to check for you. You're not having anything else.

Answer: it is a family like me, if I also had to apply at that time, is my daughter my son-in-law she is also a civil servant.

She works in the internal department. Well, if I were ordinary and I wouldn't want that much, I would apply to the disabled government, but I was just getting right, I didn't do that. Well, even a country is not very easy either. You see, the international situation is not good, and this country costs a lot of money.

A lot of money, if you otherwise, if the international situation is good, we can improve a little, but no way, the world is like this.

Ask: ah, big ye, you are what work do? I think you understand the government that way,

Answer: I farm by land, farm by oneself.

Q: Yeah, what did you don't do when you came here in' 97?

Answer: After doing a small business, take grandchildren.

Q: Then you just said that you just said that they are still good. But do you feel that from your own point of view, do you as an old person, do you think they can do a little better? To make you even happier? Is there any advice from here?

A: That's not so demanding. Ok, Ok, thank you for working hard

Q: How old are you now this year?

Answer: 66.

Q: Oh, you can't see it at all, very clear.

A: Ah, yes, yes.

Q: Are you this native person?

A: No,

Q: Is this one from Erlin village? Is it?

Answer: It is Hunan Province.

Q: Where do you live now?

Answer: I now live in that student electronics factory is too far away, others do health, do cleaner.

Q: Oh, understand, understand, understand. Where is your niece right now?

A: My niece and my two sons both work in the mother's factory here.

Q: Oh. Well, how many nieces do you have?

A: Only two ah, two children.

Q: Oh, good, good, good good. Well, do you think your children are filial now? It's like filial piety, right?

A: Yes.life. I'm just very satisfied. It's all relatively big.

Q: What do you think of the living environment around you now? How are you living here?

A: Ok,

Q: The air is very good in all aspects, right?

A: It's pretty good,

Q: It's all better with the neighborhood,

A: Good, fine.

Q: Well, what do you usually like to do? Is what hobby is not ah, or what you play cards usually play cards.

A: I don't like this. Sometimes I just want to play the flute.

Q: ah, good, good, this is good ah, this is very edify sentiment ah,

A: Yes.

Q: What do you think are the happiest things in the world now? Let you be satisfied with it.

A: I'm just what I just said right now,

Q: Your children are all around you, right?

A: Yes, yes. But it's not like one factory, I'm another hygiene, they work in another factory,

Q: At least you always see each other.

A: Yes,

Q: That's pretty happy, and then that's the way you think you're happy right right.

A: Have lived a relatively happy life.

Q: Ok, ok, what is your body?

A: The body is just this. Oh little brother he feels cold, just a cold all year round

Q: I'm usually in good health, right?

A: Usually it is very good,

Q: What do you think is the most important thing in your life that affects your happiness and unhappiness?

Answer: ah, is usually all aspects are very satisfied, all very satisfied.

Q: What do you think is the community, or our hospital, or what things need to be improved? It can help you improve your satisfaction and happiness.

A: Oh, we all have to talk about this alone, and we're not that high level, that's what you say feels good now. Now you see all parties face these old people, whether middle-aged and old people are very good,

Q: What do you think is inconvenient? For example, does the hospital have any need to take an exclusive channel for the elderly ah these or the number of your physical examination should be more?

A: Oh, yes, this is these now this era of development is so fast, is these for intelligent things for us to do. The lack of guidance staff in the hospital is to guide you to some intelligent these introduction.

Q: Understand, thank you very much, hello, good, good, good

A: Thank you

Q: How old are you, please?

A: 38 years old

Q: What ways do you think the hospital should take to increase the happiness of the elderly?

Answer: the hospital guidance personnel is insufficient, insufficient parking space, the lack of special access for the elderly.

Q: How old are you this year?

A: 44

Q: What ways do you think are possible to improve the happiness of the elderly?

Answer: organize old people physical examination activity more, good family atmosphere is very important, old people have a hobby also very important, otherwise energy is put on children body, easy spirit is empty. In addition, in terms of national medical security, the problem of remote medical treatment needs to be paid attention to. My parents, for example, have a low proportion of remote medical expenses.

Q: How old are you aged now?
 A: I am 68 years old this year.

Q: How are you doing?
 A: I started having diabetes at the age of 60, and I have been taking medicine to control it.

Q: How many sons do you have?
 A: Two sons. Our family used to live in the countryside, but later we moved to Dongguan to farm. Although the land was not much, it was better than my hometown.

Q: How long have you been here since then?
 Answer: have 35 years, after dividing the field to the household came. The family land is not enough to eat, to Dongguan farming life is better.

Q: That means like you and your husband came here early. Why are you getting a Yangjiang hukou?
 A: Our hukou is still in Yangjiang, not moved when we came to Dongguan.

Q: Are all of your children being born here?
 A: Yes, the two sons were both born in Dongguan.

Q: What do you think of your life right now? Are you satisfied?
 A: Life is ok, my sons are very good to me, and my husband also cares about me. Although the body is some uncomfortable, but the overall satisfaction.

Q: What is the living environment? Are you satisfied?
 A: We live in the houses we built. The environment is good and the neighbors are good.

Q: Do you usually have any recreational activities?
 A: Usually will chat with the neighbors, occasionally walk, do not play cards, also do not dance.

Q: What is the most satisfying thing in your life?
 A: The most satisfied thing is that my sons have grown up, got married and employed, and have their grandchildren. I am very happy to see them grow up healthily.

Q: Do you think what else that the community or the government can do to make you happier?
 A: I am quite satisfied by now. There are no special requirements.

Q: How old are you aged now?
 A: I'm 70 years old.

Q: How are you doing?
 Answer: the body is ok, it is the knee is not good, often ache.

Q: How many sons do you have?
 A: Four sons. We used to farm in our hometown, and then we moved to Dongguan to look for opportunities.

Q: How long have you been here since then?
 A: It's been about 45 years ago, and I moved here to find a better life.

Ask: that your registered permanent residence still in old home?
 Answer: right, registered permanent residence is in hometown all the time, did not move over.

Q: Are all of your children being born here?
 A: Yes, all four sons were born and raised in Dongguan.

Q: Are you satisfied with your current life situation?
 Answer: still calculate satisfied, the children are very filial piety, life also does not worry about food and clothing.

Q: What is the living environment?
 A: The environment is ok, I live in self-built houses, and the neighbors are very friendly.

Q: Do you usually have any recreational activities?
 Answer: usually chat with friends, watch TV, do not like to go out for activities.

Q: What is the happiest part in your life?
 A: I was satisfied to see all my sons get married and all my grandchildren grow up healthy.

Q: Do you think what the community or the government can do to make you happier?
 A: The government has done something very well. I don't have any special requirements.

Q: How old are you aged now?
 A: I is 72 years old.

Q: How are you doing?
 A: The body is general, with hypertension and arthritis.

Q: How many sons do you have?
 A: Two sons. Our family could not go on living in my hometown, so we moved to Dongguan to look for opportunities.

Q: How long have you been here since then?
 A: For 40 years, I moved here for a better life.

Ask: that your registered permanent residence still in old home?
 Answer: right, registered permanent residence is in hometown all the time, after coming Dongguan also did not move.

Q: Are all of your children being born here?
 A: Yes, both sons were born in Dongguan.

Q: Are you satisfied with your current life situation?
 A: Life is ok, the children are very filial piety, life is ok.

Q: What is the living environment?
 A: The environment is good, live in the house built, neighbors are very good.

Q: Do you usually have any recreational activities?
 A: I usually chat with my neighbors, and sometimes I watch my grandchildren play without playing cards or dancing.

Q: What is the happiest part in your life?
 A: The happiest thing is to see my sons get married and their grandchildren grow up healthily.

Q: Do you think what the community or the government can do to make you happier?
 A: I am quite satisfied now. There are no special requirements.

Q: How old are you aged now?
 A: I am 69 years old.

Q: How are you doing?
 Answer: the body is ok, it is occasionally some small problems.

Q: How many sons do you have?
 A: Three sons. We used to be in our hometown, and then we moved to Dongguan to farm.

Q: How long have you been here since then?
 A: For 38 years, I moved here after dividing the land to the household.

Ask: that your registered permanent residence still in old home?
 Answer: right, the registered permanent residence has been in the hometown.

Q: Are all of your children being born here?
 A: Yes, all three sons were born in Dongguan.

Q: Are you satisfied with your current life situation?
 Answer: Life is ok, the sons are very filial piety, the life is ok.

Q: What is the living environment?
 Answer: the environment is ok, live in is the house that oneself builds, the surrounding neighbors are very good.

Q: Do you usually have any recreational activities?
 A: Usually chat with neighbors, watch TV, don't like to go out for activities.

Q: What is the happiest part in your life?
 A: The happiest thing is to see my sons get married and and their grandchildren grow up healthily.

Q: Do you think what the community or the government can do to make you happier?
 A: The government has done it very well. I don't have any special requirements.

Q: How old are you aged now?
 A: I is 71 years old.

Q: How are you doing?
 Answer: the body is ok, it is some senile disease.

Q: How many sons do you have?
 A: Two sons. We could not live in our hometown before, so we moved to Dongguan to look for opportunities.

Q: How long have you been here since then?
 A: For 43 years, I moved in for a better life.

Ask: that your registered permanent residence still in old home?
 Answer: right, the registered permanent residence has been in the hometown.

Q: Are all of your children being born here?
 A: Yes, both sons were born in Dongguan.

Q: Are you satisfied with your current life situation?
 Answer: Life is ok, the children are very filial piety, the life is ok.

Q: What is the living environment?
 A: The environment is good, live in the house built, neighbors are very good.

Q: Do you usually have any recreational activities?
 A: I usually chat with my neighbors, and sometimes I watch my grandchildren play without playing cards or dancing.

Q: What is the happiest part in your life?
 A: The happiest thing is to see my sons get married and and their grandchildren grow up healthily.

Q: Do you think what the community or the government can do to make you happier?
 A: I am quite satisfied now. There are no special requirements.

Q: How old are you aged now?
 A: 81 years old.
 Q: Are you a local person?
 Answer: My hometown is in Chaoshan. I visited Shenzhen 6 years ago, and now I live in Dongguan. He had three sons and a daughter, and the sons did business, so they moved over with him.
 Q: You look so healthy.
 A: Yes, I always get sick,
Q: But you look very energetic. What are the main diseases?
 A: This heart disease. Heart at first the body is still very good, 69 years old that year car accident ah, originally I was often often have exercise ah, this his foot was broken, just stay at home for a year. I started having a bad heart at the end of my 70s. Well, the heart is not good. I keep taking that heart medicine. Well. Just eat about four or five years, four or five years later began that stomach is bad, gastric ulcer, slowly chronic gastritis erosion. After this heart also this to this foot influence, began the heart is not good. The heart is not good to take medicine for a long time, the stomach is bad.be not allowed. Now you can't even take that cerebrovascular, nor is the arteriosclerosis.
 Ask: oh, this way, that this is really multiple serious diseases, that you these diseases will usually affect your life a lot, is not you usually affect your own life, or mood ah, family relations, old body health can body body is not healthy trouble. Well, then you think you now the whole of your own life, because these diseases will be your life is generally satisfied?
 A: Generally speaking, it is still ok, right?
 Q: that you and the children their family relationship, and neighbor relationship these all very good?
 A: Generally,
Q: Not so much, right? How you have your children. How many children are there?
 A: Three boys and a girl.
 Q: Oh, four children. Are you a local person or a nonlocal person?
 Answer: I chaoshan come over, how long did you come over? I started working out in Shenzhen when I was 50.
 Q: I came here in 1997. I have been here for a long time. There are still more than ten days, more than 20 years away.
 Q: Well. Oh, if you come here, will you come here for your own business, or.
 A: My son did a little business.
 Q: So you came here with your son, so you came along with you?
 A: Well.
 Q: Oh, so, that's like most of the family population is here,
A: Yes, son, what daughter-in-law, grandchildren, and these are all here.
 Q: What do you think is the happiest thing in your life now is what you are most satisfied with. What are you most satisfied with in your whole state of life?
 Answer: satisfaction is this has children to have a family, have grandson. That grandson even graduated from college and came out to work.

 Q: Generally speaking, I am quite satisfied with my own feelings and my life. Still satisfied? Or not how satisfied?
 A: Satisfied.
 Q: Do you think you are living with your children or not right now?
 A: Live together.
 Q: That means that you think you live now and you say you live with your children, right?
 A: Yes.
 Q: If you live together, do you think it is quite different to live together with the young people? Or do you prefer to live alone or prefer to live with your children to answer: ah. No, no, then you are all the young people are very filial piety.

 Q: Oh, that's good, because many young people are not used to living with the old people. Generally speaking, you feel very comfortable living with them, don't they?
 Answer: That is still very filial piety.
 Q: Are you usually sick, they will accompany you to see a doctor. Will come with you, won't you? For example, what you usually take medicine, they will help you to pay attention to ah or how
A: Yes, yes.
 Q: Ok, that means you think you mean you are generally satisfied, but do you really think about it? Well, from the community, or the government, or from the community or other aspects, you can make your life a little better and happier. Do you have any good advice? What can we do? From the community, ah, or from this medical staff, ah, or from the government, ah, what do you think we can do better. It can improve your satisfaction, improve your satisfaction, improve your life happiness, then you are happier.

 Answer: that generally basically in that community now, that service also generally still can ah.
 Q: Generally speaking, do you think this kind of service in their community is ok in all aspects. Does the government have any care or special care for your elderly people?
 A: Yes, yes, yes, yes,
Q: What are the benefits.

 A: For example, it was in the countryside, ah ah, I live at the age of MAO Zedong era, it was very hard at that time. Yes, yes, that substance is very good, and you will get bored. Yes, that's different now. Since I have been in the countryside since I was 65 years ago, then the government has taken care of the old man. At first, it is 50 yuan, 50 yuan a month after 55 yuan, slowly lift, slowly lift, until now there are more than 200 points.
 Q: Every month, right?
 A: Yes
Q: every month you although you this number in the outside this situation is that proportion is very small, right? But you have so much money so much old people calculate that amount is very powerful. Then you quite understand the government.
 A: Well, yes.
 Q: Do they give you, for example, the usual care is if the community pays you if the government gives you some other benefits. Ask you how well you are. That means to care about you or to check for you. You're not having anything else.

 Answer: it is a family like me, if I also had to apply at that time, is my daughter my son-in-law she is also a civil servant.

 She works in the internal department. Well, if I were ordinary and I wouldn't want that much, I would apply to the disabled government, but I was just getting right, I didn't do that. Well, even a country is not very easy either. You see, the international situation is not good, and this country costs a lot of money.
 A lot of money, if you otherwise, if the international situation is good, we can improve a little, but no way, the world is like this.
 Ask: ah, big ye, you are what work do? I think you understand the government that way,
Answer: I farm by land, farm by oneself.
 Q: Yeah, what did you don't do when you came here in' 97?

 Answer: After doing a small business, take grandchildren.
 Q: Then you just said that you just said that they are still good. But do you feel that from your own point of view, do you as an old person, do you think they can do a little better? To make you even happier? Is there any advice from here?

 A: That's not so demanding. Ok, Ok, thank you for working hard

Q: How old are you aged now?
 A: 82 years old.
 Q: Are you a local person?
 Answer: My hometown is in Chaoshan. I visited Shenzhen 7 years ago, and now I live in Dongguan. He had three sons and a daughter, and the sons did business, so they moved over with him.
 Q: You look so healthy.
 A: Yes, I always get sick,
Q: But you look very energetic. What are the main diseases?
 A: This heart disease. Heart at first the body is still very good, 70 years old that year car accident, originally I was often often have exercise, this his foot broke, just stay at home for a year. I started having a bad heart at the end of age 71. Well, the heart is not good. I keep taking that heart medicine. Well. Just eat about four or five years, four or five years later began that stomach is bad, gastric ulcer, slowly chronic gastritis erosion. After this heart also this to this foot influence, began the heart is not good. The heart is not good to take medicine for a long time, the stomach is bad.be not allowed. Now you can't even take that cerebrovascular, nor is the arteriosclerosis.
 Ask: oh, this way, that this is really multiple serious diseases, that you these diseases will usually affect your life a lot, is not you usually affect your own life, or mood ah, family relations, old body health can body body is not healthy trouble. Well, then you think you now the whole of your own life, because these diseases will be your life is generally satisfied?
 A: Generally speaking, it is still ok, right?
 Q: that you and the children their family relationship, and neighbor relationship these all very good?
 A: Generally,
Q: Not so much, right? How you have your children. How many children are there?
 A: Three boys and a girl.
 Q: Oh, four children. Are you a local person or a nonlocal person?
 Answer: I chaoshan come over, how long did you come over? I started working out in Shenzhen when I was 50.
 Q: I came here in 1997. I have been here for a long time. There are still more than ten days, more than 20 years away.
 Q: Well. Oh, if you come here, will you come here for your own business, or.
 A: My son did a little business.
 Q: So you came here with your son, so you came along with you?
 A: Well.
 Q: Oh, so, that's like most of the family population is here,
A: Yes, son, what daughter-in-law, grandchildren, and these are all here.
 Q: What do you think is the happiest thing in your life now is what you are most satisfied with. What are you most satisfied with in your whole state of life?
 Answer: satisfaction is this has children to have a family, have grandson. That grandson even graduated from college and came out to work.

 Q: Generally speaking, I am quite satisfied with my own feelings and my life. Still satisfied? Or not how satisfied?
 A: Satisfied.
 Q: Do you think you are living with your children or not right now?
 A: Live together.
 Q: That means that you think you live now and you say you live with your children, right?
 A: Yes.
 Q: If you live together, do you think it is quite different to live together with the young people? Or do you prefer to live alone or prefer to live with your children to answer: ah. No, no, then you are all the young people are very filial piety.

 Q: Oh, that's good, because many young people are not used to living with the old people. Generally speaking, you feel very comfortable living with them, don't they?
 Answer: That is still very filial piety.
 Q: Are you usually sick, they will accompany you to see a doctor. Will come with you, won't you? For example, what you usually take medicine, they will help you to pay attention to ah or how
A: Yes, yes.
 Q: Ok, that means you think you mean you are generally satisfied, but do you really think about it? Well, from the community, or the government, or from the community or other aspects, you can make your life a little better and happier. Do you have any good advice? What can we do? From the community, ah, or from this medical staff, ah, or from the government, ah, what do you think we can do better. It can improve your satisfaction, improve your satisfaction, improve your life happiness, then you are happier.

 Answer: that generally basically in that community now, that service also generally still can ah.
 Q: Generally speaking, do you think this kind of service in their community is ok in all aspects. Does the government have any care or special care for your elderly people?
 A: Yes, yes, yes, yes,
Q: What are the benefits.

 A: For example, it was in the countryside, ah ah, I live at the age of MAO Zedong era, it was very hard at that time. Yes, yes, that substance is very good, and you will get bored. Yes, that's different now. Since I have been in the countryside since I was 65 years ago, then the government has taken care of the old man. At first, it is 50 yuan, 50 yuan a month after 55 yuan, slowly lift, slowly lift, until now there are more than 200 points.
 Q: Every month, right?
 A: Yes
Q: every month you although you this number in the outside this situation is that proportion is very small, right? But you have so much money so much old people calculate that amount is very powerful. Then you quite understand the government.
 A: Well, yes.
 Q: Do they give you, for example, the usual care is if the community pays you if the government gives you some other benefits. Ask you how well you are. That means to care about you or to check for you. You're not having anything else.

 Answer: it is a family like me, if I also had to apply at that time, is my daughter my son-in-law she is also a civil servant.

 She works in the internal department. Well, if I were ordinary and I wouldn't want that much, I would apply to the disabled government, but I was just getting right, I didn't do that. Well, even a country is not very easy either. You see, the international situation is not good, and this country costs a lot of money.
 A lot of money, if you otherwise, if the international situation is good, we can improve a little, but no way, the world is like this.
 Ask: ah, big ye, you are what work do? I think you understand the government that way,
Answer: I farm by land, farm by oneself.
 Q: Yeah, what did you don't do when you came here in' 97?

 Answer: After doing a small business, take grandchildren.
 Q: Then you just said that you just said that they are still good. But do you feel that from your own point of view, do you as an old person, do you think they can do a little better? To make you even happier? Is there any advice from here?

 A: That's not so demanding. Ok, Ok, thank you for working hard

Q: How old are you aged now?
 A: 83 years old.
 Q: Are you a local person?
 A: My hometown is in Chaoshan. I visited Shenzhen 8 years ago, and now I live in Dongguan. He had three sons and a daughter, and the sons did business, so they moved over with him.
 Q: You look so healthy.
 A: Yes, I always get sick,
Q: But you look very energetic. What are the main diseases?
 A: This heart disease. Heart at first the body is still very good, 71 years old that year car accident ah, originally I was often often have exercise ah, this his foot broke, just stay at home for a year. He started having a bad heart at the end of age 72. Well, the heart is not good. I keep taking that heart medicine. Well. Just eat about four or five years, four or five years later began that stomach is bad, gastric ulcer, slowly chronic gastritis erosion. After this heart also this to this foot influence, began the heart is not good. The heart is not good to take medicine for a long time, the stomach is bad.be not allowed. Now you can't even take that cerebrovascular, nor is the arteriosclerosis.
 Ask: oh, this way, that this is really multiple serious diseases, that you these diseases will usually affect your life a lot, is not you usually affect your own life, or mood ah, family relations, old body health can body body is not healthy trouble. Well, then you think you now the whole of your own life, because these diseases will be your life is generally satisfied?
 A: Generally speaking, it is still ok, right?
 Q: that you and the children their family relationship, and neighbor relationship these all very good?
 A: Generally,
Q: Not so much, right? How you have your children. How many children are there?
 A: Three boys and a girl.
 Q: Oh, four children. Are you a local person or a nonlocal person?
 Answer: I chaoshan come over, how long did you come over? I started working out in Shenzhen when I was 50.
 Q: I came here in 1997. I have been here for a long time. There are still more than ten days, more than 20 years away.
 Q: Well. Oh, if you come here, will you come here for your own business, or.
 A: My son did a little business.
 Q: So you came here with your son, so you came along with you?
 A: Well.
 Q: Oh, so, that's like most of the family population is here,
A: Yes, son, what daughter-in-law, grandchildren, and these are all here.
 Q: What do you think is the happiest thing in your life now is what you are most satisfied with. What are you most satisfied with in your whole state of life?
 Answer: satisfaction is this has children to have a family, have grandson. That grandson even graduated from college and came out to work.

 Q: Generally speaking, I am quite satisfied with my own feelings and my life. Still satisfied? Or not how satisfied?
 A: Satisfied.
 Q: Do you think you are living with your children or not right now?
 A: Live together.
 Q: That means that you think you live now and you say you live with your children, right?
 A: Yes.
 Q: If you live together, do you think it is quite different to live together with the young people? Or do you prefer to live alone or prefer to live with your children to answer: ah. No, no, then you are all the young people are very filial piety.

 Q: Oh, that's good, because many young people are not used to living with the old people. Generally speaking, you feel very comfortable living with them, don't they?
 Answer: That is still very filial piety.
 Q: Are you usually sick, they will accompany you to see a doctor. Will come with you, won't you? For example, what you usually take medicine, they will help you to pay attention to ah or how
A: Yes, yes.
 Q: Ok, that means you think you mean you are generally satisfied, but do you really think about it? Well, from the community, or the government, or from the community or other aspects, you can make your life a little better and happier. Do you have any good advice? What can we do? From the community, ah, or from this medical staff, ah, or from the government, ah, what do you think we can do better. It can improve your satisfaction, improve your satisfaction, improve your life happiness, then you are happier.

 Answer: that generally basically in that community now, that service also generally still can ah.
 Q: Generally speaking, do you think this kind of service in their community is ok in all aspects. Does the government have any care or special care for your elderly people?
 A: Yes, yes, yes, yes,
Q: What are the benefits.

 A: For example, it was in the countryside, ah ah, I live at the age of MAO Zedong era, it was very hard at that time. Yes, yes, that substance is very good, and you will get bored. Yes, that's different now. Since I have been in the countryside since I was 65 years ago, then the government has taken care of the old man. At first, it is 50 yuan, 50 yuan a month after 55 yuan, slowly lift, slowly lift, until now there are more than 200 points.
 Q: Every month, right?
 A: Yes
Q: every month you although you this number in the outside this situation is that proportion is very small, right? But you have so much money so much old people calculate that amount is very powerful. Then you quite understand the government.
 A: Well, yes.
 Q: Do they give you, for example, the usual care is if the community pays you if the government gives you some other benefits. Ask you how well you are. That means to care about you or to check for you. You're not having anything else.

 Answer: it is a family like me, if I also had to apply at that time, is my daughter my son-in-law she is also a civil servant.

 She works in the internal department. Well, if I were ordinary and I wouldn't want that much, I would apply to the disabled government, but I was just getting right, I didn't do that. Well, even a country is not very easy either. You see, the international situation is not good, and this country costs a lot of money.
 A lot of money, if you otherwise, if the international situation is good, we can improve a little, but no way, the world is like this.
 Ask: ah, big ye, you are what work do? I think you understand the government that way,
Answer: I farm by land, farm by oneself.
 Q: Yeah, what did you don't do when you came here in' 97?

 Answer: After doing a small business, take grandchildren.
 Q: Then you just said that you just said that they are still good. But do you feel that from your own point of view, do you as an old person, do you think they can do a little better? To make you even happier? Is there any advice from here?

 A: That's not so demanding. Ok, Ok, thank you for working hard

Q: How old are you aged now?
 A: 84 years old.
 Q: Are you a local person?
 A: My hometown is in Chaoshan. I visited Shenzhen 9 years ago, and now I live in Dongguan. He had three sons and a daughter, and the sons did business, so they moved over with him.
 Q: You look so healthy.
 A: Yes, I always get sick,
Q: But you look very energetic. What are the main diseases?
 A: This heart disease. Heart at first the body is very good, 72 years old that year car accident, originally I was often often have exercise, this his foot broken, just stay at home for a year. He started having a bad heart at the end of age 73. Well, the heart is not good. I keep taking that heart medicine. Well. Just eat about four or five years, four or five years later began that stomach is bad, gastric ulcer, slowly chronic gastritis erosion. After this heart also this to this foot influence, began the heart is not good. The heart is not good to take medicine for a long time, the stomach is bad.be not allowed. Now you can't even take that cerebrovascular, nor is the arteriosclerosis.
 Ask: oh, this way, that this is really multiple serious diseases, that you these diseases will usually affect your life a lot, is not you usually affect your own life, or mood ah, family relations, old body health can body body is not healthy trouble. Well, then you think you now the whole of your own life, because these diseases will be your life is generally satisfied?
 A: Generally speaking, it is still ok, right?
 Q: that you and the children their family relationship, and neighbor relationship these all very good?
 A: Generally,
Q: Not so much, right? How you have your children. How many children are there?
 A: Three boys and a girl.
 Q: Oh, four children. Are you a local person or a nonlocal person?
 Answer: I chaoshan come over, how long did you come over? I started working out in Shenzhen when I was 50.
 Q: I came here in 1997. I have been here for a long time. There are still more than ten days, more than 20 years away.
 Q: Well. Oh, if you come here, will you come here for your own business, or.
 A: My son did a little business.
 Q: So you came here with your son, so you came along with you?
 A: Well.
 Q: Oh, so, that's like most of the family population is here,
A: Yes, son, what daughter-in-law, grandchildren, and these are all here.
 Q: What do you think is the happiest thing in your life now is what you are most satisfied with. What are you most satisfied with in your whole state of life?
 Answer: satisfaction is this has children to have a family, have grandson. That grandson even graduated from college and came out to work.

 Q: Generally speaking, I am quite satisfied with my own feelings and my life. Still satisfied? Or not how satisfied?
 A: Satisfied.
 Q: Do you think you are living with your children or not right now?
 A: Live together.
 Q: That means that you think you live now and you say you live with your children, right?
 A: Yes.
 Q: If you live together, do you think it is quite different to live together with the young people? Or do you prefer to live alone or prefer to live with your children to answer: ah. No, no, then you are all the young people are very filial piety.

 Q: Oh, that's good, because many young people are not used to living with the old people. Generally speaking, you feel very comfortable living with them, don't they?
 Answer: That is still very filial piety.
 Q: Are you usually sick, they will accompany you to see a doctor. Will come with you, won't you? For example, what you usually take medicine, they will help you to pay attention to ah or how
A: Yes, yes.
 Q: Ok, that means you think you mean you are generally satisfied, but do you really think about it? Well, from the community, or the government, or from the community or other aspects, you can make your life a little better and happier. Do you have any good advice? What can we do? From the community, ah, or from this medical staff, ah, or from the government, ah, what do you think we can do better. It can improve your satisfaction, improve your satisfaction, improve your life happiness, then you are happier.

 Answer: that generally basically in that community now, that service also generally still can ah.
 Q: Generally speaking, do you think this kind of service in their community is ok in all aspects. Does the government have any care or special care for your elderly people?
 A: Yes, yes, yes, yes,
Q: What are the benefits.

 A: For example, it was in the countryside, ah ah, I live at the age of MAO Zedong era, it was very hard at that time. Yes, yes, that substance is very good, and you will get bored. Yes, that's different now. Since I have been in the countryside since I was 65 years ago, then the government has taken care of the old man. At first, it is 50 yuan, 50 yuan a month after 55 yuan, slowly lift, slowly lift, until now there are more than 200 points.
 Q: Every month, right?
 A: Yes
Q: every month you although you this number in the outside this situation is that proportion is very small, right? But you have so much money so much old people calculate that amount is very powerful. Then you quite understand the government.
 A: Well, yes.
 Q: Do they give you, for example, the usual care is if the community pays you if the government gives you some other benefits. Ask you how well you are. That means to care about you or to check for you. You're not having anything else.

 Answer: it is a family like me, if I also had to apply at that time, is my daughter my son-in-law she is also a civil servant.

 She works in the internal department. Well, if I were ordinary and I wouldn't want that much, I would apply to the disabled government, but I was just getting right, I didn't do that. Well, even a country is not very easy either. You see, the international situation is not good, and this country costs a lot of money.
 A lot of money, if you otherwise, if the international situation is good, we can improve a little, but no way, the world is like this.
 Ask: ah, big ye, you are what work do? I think you understand the government that way,
Answer: I farm by land, farm by oneself.
 Q: Yeah, what did you don't do when you came here in' 97?

 Answer: After doing a small business, take grandchildren.
 Q: Then you just said that you just said that they are still good. But do you feel that from your own point of view, do you as an old person, do you think they can do a little better? To make you even happier? Is there any advice from here?

 A: That's not so demanding. Ok, Ok, thank you for working hard

Q: How old are you aged now?
 A: 85 years old.
 Q: Are you a local person?
 A: My hometown is in Chaoshan. I came to Shenzhen 10 years ago, and now I live in Dongguan. He had three sons and a daughter, and the sons did business, so they moved over with him.
 Q: You look so healthy.
 A: Yes, I always get sick,
Q: But you look very energetic. What are the main diseases?
 A: This heart disease. Heart at first the body is still very good, 73 years old that year car accident, originally I was often often have exercise, this his foot was broken, just stay at home for a year. He started having a bad heart at the end of age 74. Well, the heart is not good. I keep taking that heart medicine. Well. Just eat about four or five years, four or five years later began that stomach is bad, gastric ulcer, slowly chronic gastritis erosion. After this heart also this to this foot influence, began the heart is not good. The heart is not good to take medicine for a long time, the stomach is bad.be not allowed. Now you can't even take that cerebrovascular, nor is the arteriosclerosis.
 Ask: oh, this way, that this is really multiple serious diseases, that you these diseases will usually affect your life a lot, is not you usually affect your own life, or mood ah, family relations, old body health can body body is not healthy trouble. Well, then you think you now the whole of your own life, because these diseases will be your life is generally satisfied?
 A: Generally speaking, it is still ok, right?
 Q: that you and the children their family relationship, and neighbor relationship these all very good?
 A: Generally,
Q: Not so much, right? How you have your children. How many children are there?
 A: Three boys and a girl.
 Q: Oh, four children. Are you a local person or a nonlocal person?
 Answer: I chaoshan come over, how long did you come over? I started working out in Shenzhen when I was 50.
 Q: I came here in 1997. I have been here for a long time. There are still more than ten days, more than 20 years away.
 Q: Well. Oh, if you come here, will you come here for your own business, or.
 A: My son did a little business.
 Q: So you came here with your son, so you came along with you?
 A: Well.
 Q: Oh, so, that's like most of the family population is here,
A: Yes, son, what daughter-in-law, grandchildren, and these are all here.
 Q: What do you think is the happiest thing in your life now is what you are most satisfied with. What are you most satisfied with in your whole state of life?
 Answer: satisfaction is this has children to have a family, have grandson. That grandson even graduated from college and came out to work.

 Q: Generally speaking, I am quite satisfied with my own feelings and my life. Still satisfied? Or not how satisfied?
 A: Satisfied.
 Q: Do you think you are living with your children or not right now?
 A: Live together.
 Q: That means that you think you live now and you say you live with your children, right?
 A: Yes.
 Q: If you live together, do you think it is quite different to live together with the young people? Or do you prefer to live alone or prefer to live with your children to answer: ah. No, no, then you are all the young people are very filial piety.

 Q: Oh, that's good, because many young people are not used to living with the old people. Generally speaking, you feel very comfortable living with them, don't they?
 Answer: That is still very filial piety.
 Q: Are you usually sick, they will accompany you to see a doctor. Will come with you, won't you? For example, what you usually take medicine, they will help you to pay attention to ah or how
A: Yes, yes.
 Q: Ok, that means you think you mean you are generally satisfied, but do you really think about it? Well, from the community, or the government, or from the community or other aspects, you can make your life a little better and happier. Do you have any good advice? What can we do? From the community, ah, or from this medical staff, ah, or from the government, ah, what do you think we can do better. It can improve your satisfaction, improve your satisfaction, improve your life happiness, then you are happier.

 Answer: that generally basically in that community now, that service also generally still can ah.
 Q: Generally speaking, do you think this kind of service in their community is ok in all aspects. Does the government have any care or special care for your elderly people?
 A: Yes, yes, yes, yes,
Q: What are the benefits.

 A: For example, it was in the countryside, ah ah, I live at the age of MAO Zedong era, it was very hard at that time. Yes, yes, that substance is very good, and you will get bored. Yes, that's different now. Since I have been in the countryside since I was 65 years ago, then the government has taken care of the old man. At first, it is 50 yuan, 50 yuan a month after 55 yuan, slowly lift, slowly lift, until now there are more than 200 points.
 Q: Every month, right?
 A: Yes
Q: every month you although you this number in the outside this situation is that proportion is very small, right? But you have so much money so much old people calculate that amount is very powerful. Then you quite understand the government.
 A: Well, yes.
 Q: Do they give you, for example, the usual care is if the community pays you if the government gives you some other benefits. Ask you how well you are. That means to care about you or to check for you. You're not having anything else.

 Answer: it is a family like me, if I also had to apply at that time, is my daughter my son-in-law she is also a civil servant.

 She works in the internal department. Well, if I were ordinary and I wouldn't want that much, I would apply to the disabled government, but I was just getting right, I didn't do that. Well, even a country is not very easy either. You see, the international situation is not good, and this country costs a lot of money.
 A lot of money, if you otherwise, if the international situation is good, we can improve a little, but no way, the world is like this.
 Ask: ah, big ye, you are what work do? I think you understand the government that way,
Answer: I farm by land, farm by oneself.
 Q: Yeah, what did you don't do when you came here in' 97?

 Answer: After doing a small business, take grandchildren.
 Q: Then you just said that you just said that they are still good. But do you feel that from your own point of view, do you as an old person, do you think they can do a little better? To make you even happier? Is there any advice from here?

 A: That's not so demanding. Ok, Ok, thank you for working hard

Q: How old are you aged now?
 A: 86 years old.
 Q: Are you a local person?
 Answer: My hometown is in Chaoshan. I visited Shenzhen 6 years ago, and now I live in Dongguan. He had three sons and a daughter, and the sons did business, so they moved over with him.
 Q: You look so healthy.
 A: Yes, I always get sick,
Q: But you look very energetic. What are the main diseases?
 A: This heart disease. Heart at first the body is still very good, 69 years old that year car accident ah, originally I was often often have exercise ah, this his foot was broken, just stay at home for a year. I started having a bad heart at the end of my 70s. Well, the heart is not good. I keep taking that heart medicine. Well. Just eat about four or five years, four or five years later began that stomach is bad, gastric ulcer, slowly chronic gastritis erosion. After this heart also this to this foot influence, began the heart is not good. The heart is not good to take medicine for a long time, the stomach is bad.be not allowed. Now you can't even take that cerebrovascular, nor is the arteriosclerosis.
 Ask: oh, this way, that this is really multiple serious diseases, that you these diseases will usually affect your life a lot, is not you usually affect your own life, or mood ah, family relations, old body health can body body is not healthy trouble. Well, then you think you now the whole of your own life, because these diseases will be your life is generally satisfied?
 A: Generally speaking, it is still ok, right?
 Q: that you and the children their family relationship, and neighbor relationship these all very good?
 A: Generally,
Q: Not so much, right? How you have your children. How many children are there?
 A: Three boys and a girl.
 Q: Oh, four children. Are you a local person or a nonlocal person?
 Answer: I chaoshan come over, how long did you come over? I started working out in Shenzhen when I was 50.
 Q: I came here in 1997. I have been here for a long time. There are still more than ten days, more than 20 years away.
 Q: Well. Oh, if you come here, will you come here for your own business, or.
 A: My son did a little business.
 Q: So you came here with your son, so you came along with you?
 A: Well.
 Q: Oh, so, that's like most of the family population is here,
A: Yes, son, what daughter-in-law, grandchildren, and these are all here.
 Q: What do you think is the happiest thing in your life now is what you are most satisfied with. What are you most satisfied with in your whole state of life?
 Answer: satisfaction is this has children to have a family, have grandson. That grandson even graduated from college and came out to work.

 Q: Generally speaking, I am quite satisfied with my own feelings and my life. Still satisfied? Or not how satisfied?
 A: Satisfied.
 Q: Do you think you are living with your children or not right now?
 A: Live together.
 Q: That means that you think you live now and you say you live with your children, right?
 A: Yes.
 Q: If you live together, do you think it is quite different to live together with the young people? Or do you prefer to live alone or prefer to live with your children to answer: ah. No, no, then you are all the young people are very filial piety.

 Q: Oh, that's good, because many young people are not used to living with the old people. Generally speaking, you feel very comfortable living with them, don't they?
 Answer: That is still very filial piety.
 Q: Are you usually sick, they will accompany you to see a doctor. Will come with you, won't you? For example, what you usually take medicine, they will help you to pay attention to ah or how
A: Yes, yes.
 Q: Ok, that means you think you mean you are generally satisfied, but do you really think about it? Well, from the community, or the government, or from the community or other aspects, you can make your life a little better and happier. Do you have any good advice? What can we do? From the community, ah, or from this medical staff, ah, or from the government, ah, what do you think we can do better. It can improve your satisfaction, improve your satisfaction, improve your life happiness, then you are happier.

 Answer: that generally basically in that community now, that service also generally still can ah.
 Q: Generally speaking, do you think this kind of service in their community is ok in all aspects. Does the government have any care or special care for your elderly people?
 A: Yes, yes, yes, yes,
Q: What are the benefits.

 A: For example, it was in the countryside, ah ah, I live at the age of MAO Zedong era, it was very hard at that time. Yes, yes, that substance is very good, and you will get bored. Yes, that's different now. Since I have been in the countryside since I was 65 years ago, then the government has taken care of the old man. At first, it is 50 yuan, 50 yuan a month after 55 yuan, slowly lift, slowly lift, until now there are more than 200 points.
 Q: Every month, right?
 A: Yes
Q: every month you although you this number in the outside this situation is that proportion is very small, right? But you have so much money so much old people calculate that amount is very powerful. Then you quite understand the government.
 A: Well, yes.
 Q: Do they give you, for example, the usual care is if the community pays you if the government gives you some other benefits. Ask you how well you are. That means to care about you or to check for you. You're not having anything else.

 Answer: it is a family like me, if I also had to apply at that time, is my daughter my son-in-law she is also a civil servant.

 She works in the internal department. Well, if I were ordinary and I wouldn't want that much, I would apply to the disabled government, but I was just getting right, I didn't do that. Well, even a country is not very easy either. You see, the international situation is not good, and this country costs a lot of money.
 A lot of money, if you otherwise, if the international situation is good, we can improve a little, but no way, the world is like this.
 Ask: ah, big ye, you are what work do? I think you understand the government that way,
Answer: I farm by land, farm by oneself.
 Q: Yeah, what did you don't do when you came here in' 97?

 Answer: After doing a small business, take grandchildren.
 Q: Then you just said that you just said that they are still good. But do you feel that from your own point of view, do you as an old person, do you think they can do a little better? To make you even happier? Is there any advice from here?

 A: That's not so demanding. Ok, Ok, thank you for working hard

Q: How old are you aged now?
 A: 87 years old.
 Q: Are you a local person?
 Answer: My hometown is in Chaoshan. I visited Shenzhen 7 years ago, and now I live in Dongguan. He had three sons and a daughter, and the sons did business, so they moved over with him.
 Q: You look so healthy.
 A: Yes, I always get sick,
Q: But you look very energetic. What are the main diseases?
 A: This heart disease. Heart at first the body is still very good, 70 years old that year car accident, originally I was often often have exercise, this his foot broke, just stay at home for a year. I started having a bad heart at the end of age 71. Well, the heart is not good. I keep taking that heart medicine. Well. Just eat about four or five years, four or five years later began that stomach is bad, gastric ulcer, slowly chronic gastritis erosion. After this heart also this to this foot influence, began the heart is not good. The heart is not good to take medicine for a long time, the stomach is bad.be not allowed. Now you can't even take that cerebrovascular, nor is the arteriosclerosis.
 Ask: oh, this way, that this is really multiple serious diseases, that you these diseases will usually affect your life a lot, is not you usually affect your own life, or mood ah, family relations, old body health can body body is not healthy trouble. Well, then you think you now the whole of your own life, because these diseases will be your life is generally satisfied?
 A: Generally speaking, it is still ok, right?
 Q: that you and the children their family relationship, and neighbor relationship these all very good?
 A: Generally,
Q: Not so much, right? How you have your children. How many children are there?
 A: Three boys and a girl.
 Q: Oh, four children. Are you a local person or a nonlocal person?
 Answer: I chaoshan come over, how long did you come over? I started working out in Shenzhen when I was 50.
 Q: I came here in 1997. I have been here for a long time. There are still more than ten days, more than 20 years away.
 Q: Well. Oh, if you come here, will you come here for your own business, or.
 A: My son did a little business.
 Q: So you came here with your son, so you came along with you?
 A: Well.
 Q: Oh, so, that's like most of the family population is here,
A: Yes, son, what daughter-in-law, grandchildren, and these are all here.
 Q: What do you think is the happiest thing in your life now is what you are most satisfied with. What are you most satisfied with in your whole state of life?
 Answer: satisfaction is this has children to have a family, have grandson. That grandson even graduated from college and came out to work.

 Q: Generally speaking, I am quite satisfied with my own feelings and my life. Still satisfied? Or not how satisfied?
 A: Satisfied.
 Q: Do you think you are living with your children or not right now?
 A: Live together.
 Q: That means that you think you live now and you say you live with your children, right?
 A: Yes.
 Q: If you live together, do you think it is quite different to live together with the young people? Or do you prefer to live alone or prefer to live with your children to answer: ah. No, no, then you are all the young people are very filial piety.

 Q: Oh, that's good, because many young people are not used to living with the old people. Generally speaking, you feel very comfortable living with them, don't they?
 Answer: That is still very filial piety.
 Q: Are you usually sick, they will accompany you to see a doctor. Will come with you, won't you? For example, what you usually take medicine, they will help you to pay attention to ah or how
A: Yes, yes.
 Q: Ok, that means you think you mean you are generally satisfied, but do you really think about it? Well, from the community, or the government, or from the community or other aspects, you can make your life a little better and happier. Do you have any good advice? What can we do? From the community, ah, or from this medical staff, ah, or from the government, ah, what do you think we can do better. It can improve your satisfaction, improve your satisfaction, improve your life happiness, then you are happier.

 Answer: that generally basically in that community now, that service also generally still can ah.
 Q: Generally speaking, do you think this kind of service in their community is ok in all aspects. Does the government have any care or special care for your elderly people?
 A: Yes, yes, yes, yes,
Q: What are the benefits.

 A: For example, it was in the countryside, ah ah, I live at the age of MAO Zedong era, it was very hard at that time. Yes, yes, that substance is very good, and you will get bored. Yes, that's different now. Since I have been in the countryside since I was 65 years ago, then the government has taken care of the old man. At first, it is 50 yuan, 50 yuan a month after 55 yuan, slowly lift, slowly lift, until now there are more than 200 points.
 Q: Every month, right?
 A: Yes
Q: every month you although you this number in the outside this situation is that proportion is very small, right? But you have so much money so much old people calculate that amount is very powerful. Then you quite understand the government.
 A: Well, yes.
 Q: Do they give you, for example, the usual care is if the community pays you if the government gives you some other benefits. Ask you how well you are. That means to care about you or to check for you. You're not having anything else.

 Answer: it is a family like me, if I also had to apply at that time, is my daughter my son-in-law she is also a civil servant.

 She works in the internal department. Well, if I were ordinary and I wouldn't want that much, I would apply to the disabled government, but I was just getting right, I didn't do that. Well, even a country is not very easy either. You see, the international situation is not good, and this country costs a lot of money.
 A lot of money, if you otherwise, if the international situation is good, we can improve a little, but no way, the world is like this.
 Ask: ah, big ye, you are what work do? I think you understand the government that way,
Answer: I farm by land, farm by oneself.
 Q: Yeah, what did you don't do when you came here in' 97?

 Answer: After doing a small business, take grandchildren.
 Q: Then you just said that you just said that they are still good. But do you feel that from your own point of view, do you as an old person, do you think they can do a little better? To make you even happier? Is there any advice from here?

 A: That's not so demanding. Ok, Ok, thank you for working hard

Q: How old are you aged now?
 A: 88 years old.
 Q: Are you a local person?
 A: My hometown is in Chaoshan. I visited Shenzhen 8 years ago, and now I live in Dongguan. He had three sons and a daughter, and the sons did business, so they moved over with him.
 Q: You look so healthy.
 A: Yes, I always get sick,
Q: But you look very energetic. What are the main diseases?
 A: This heart disease. Heart at first the body is still very good, 71 years old that year car accident ah, originally I was often often have exercise ah, this his foot broke, just stay at home for a year. He started having a bad heart at the end of age 72. Well, the heart is not good. I keep taking that heart medicine. Well. Just eat about four or five years, four or five years later began that stomach is bad, gastric ulcer, slowly chronic gastritis erosion. After this heart also this to this foot influence, began the heart is not good. The heart is not good to take medicine for a long time, the stomach is bad.be not allowed. Now you can't even take that cerebrovascular, nor is the arteriosclerosis.
 Ask: oh, this way, that this is really multiple serious diseases, that you these diseases will usually affect your life a lot, is not you usually affect your own life, or mood ah, family relations, old body health can body body is not healthy trouble. Well, then you think you now the whole of your own life, because these diseases will be your life is generally satisfied?
 A: Generally speaking, it is still ok, right?
 Q: that you and the children their family relationship, and neighbor relationship these all very good?
 A: Generally,
Q: Not so much, right? How you have your children. How many children are there?
 A: Three boys and a girl.
 Q: Oh, four children. Are you a local person or a nonlocal person?
 Answer: I chaoshan come over, how long did you come over? I started working out in Shenzhen when I was 50.
 Q: I came here in 1997. I have been here for a long time. There are still more than ten days, more than 20 years away.
 Q: Well. Oh, if you come here, will you come here for your own business, or.
 A: My son did a little business.
 Q: So you came here with your son, so you came along with you?
 A: Well.
 Q: Oh, so, that's like most of the family population is here,
A: Yes, son, what daughter-in-law, grandchildren, and these are all here.
 Q: What do you think is the happiest thing in your life now is what you are most satisfied with. What are you most satisfied with in your whole state of life?
 Answer: satisfaction is this has children to have a family, have grandson. That grandson even graduated from college and came out to work.

 Q: Generally speaking, I am quite satisfied with my own feelings and my life. Still satisfied? Or not how satisfied?
 A: Satisfied.
 Q: Do you think you are living with your children or not right now?
 A: Live together.
 Q: That means that you think you live now and you say you live with your children, right?
 A: Yes.
 Q: If you live together, do you think it is quite different to live together with the young people? Or do you prefer to live alone or prefer to live with your children to answer: ah. No, no, then you are all the young people are very filial piety.

 Q: Oh, that's good, because many young people are not used to living with the old people. Generally speaking, you feel very comfortable living with them, don't they?
 Answer: That is still very filial piety.
 Q: Are you usually sick, they will accompany you to see a doctor. Will come with you, won't you? For example, what you usually take medicine, they will help you to pay attention to ah or how
A: Yes, yes.
 Q: Ok, that means you think you mean you are generally satisfied, but do you really think about it? Well, from the community, or the government, or from the community or other aspects, you can make your life a little better and happier. Do you have any good advice? What can we do? From the community, ah, or from this medical staff, ah, or from the government, ah, what do you think we can do better. It can improve your satisfaction, improve your satisfaction, improve your life happiness, then you are happier.

 Answer: that generally basically in that community now, that service also generally still can ah.
 Q: Generally speaking, do you think this kind of service in their community is ok in all aspects. Does the government have any care or special care for your elderly people?
 A: Yes, yes, yes, yes,
Q: What are the benefits.

 A: For example, it was in the countryside, ah ah, I live at the age of MAO Zedong era, it was very hard at that time. Yes, yes, that substance is very good, and you will get bored. Yes, that's different now. Since I have been in the countryside since I was 65 years ago, then the government has taken care of the old man. At first, it is 50 yuan, 50 yuan a month after 55 yuan, slowly lift, slowly lift, until now there are more than 200 points.
 Q: Every month, right?
 A: Yes
Q: every month you although you this number in the outside this situation is that proportion is very small, right? But you have so much money so much old people calculate that amount is very powerful. Then you quite understand the government.
 A: Well, yes.
 Q: Do they give you, for example, the usual care is if the community pays you if the government gives you some other benefits. Ask you how well you are. That means to care about you or to check for you. You're not having anything else.

 Answer: it is a family like me, if I also had to apply at that time, is my daughter my son-in-law she is also a civil servant.

 She works in the internal department. Well, if I were ordinary and I wouldn't want that much, I would apply to the disabled government, but I was just getting right, I didn't do that. Well, even a country is not very easy either. You see, the international situation is not good, and this country costs a lot of money.
 A lot of money, if you otherwise, if the international situation is good, we can improve a little, but no way, the world is like this.
 Ask: ah, big ye, you are what work do? I think you understand the government that way,
Answer: I farm by land, farm by oneself.
 Q: Yeah, what did you don't do when you came here in' 97?

 Answer: After doing a small business, take grandchildren.
 Q: Then you just said that you just said that they are still good. But do you feel that from your own point of view, do you as an old person, do you think they can do a little better? To make you even happier? Is there any advice from here?

 A: That's not so demanding. Ok, Ok, thank you for working hard

Q: How old are you aged now?
 A: 89 years old.
 Q: Are you a local person?
 A: My hometown is in Chaoshan. I visited Shenzhen 9 years ago, and now I live in Dongguan. He had three sons and a daughter, and the sons did business, so they moved over with him.
 Q: You look so healthy.
 A: Yes, I always get sick,
Q: But you look very energetic. What are the main diseases?
 A: This heart disease. Heart at first the body is very good, 72 years old that year car accident, originally I was often often have exercise, this his foot broken, just stay at home for a year. He started having a bad heart at the end of age 73. Well, the heart is not good. I keep taking that heart medicine. Well. Just eat about four or five years, four or five years later began that stomach is bad, gastric ulcer, slowly chronic gastritis erosion. After this heart also this to this foot influence, began the heart is not good. The heart is not good to take medicine for a long time, the stomach is bad.be not allowed. Now you can't even take that cerebrovascular, nor is the arteriosclerosis.
 Ask: oh, this way, that this is really multiple serious diseases, that you these diseases will usually affect your life a lot, is not you usually affect your own life, or mood ah, family relations, old body health can body body is not healthy trouble. Well, then you think you now the whole of your own life, because these diseases will be your life is generally satisfied?
 A: Generally speaking, it is still ok, right?
 Q: that you and the children their family relationship, and neighbor relationship these all very good?
 A: Generally,
Q: Not so much, right? How you have your children. How many children are there?
 A: Three boys and a girl.
 Q: Oh, four children. Are you a local person or a nonlocal person?
 Answer: I chaoshan come over, how long did you come over? I started working out in Shenzhen when I was 50.
 Q: I came here in 1997. I have been here for a long time. There are still more than ten days, more than 20 years away.
 Q: Well. Oh, if you come here, will you come here for your own business, or.
 A: My son did a little business.
 Q: So you came here with your son, so you came along with you?
 A: Well.
 Q: Oh, so, that's like most of the family population is here,
A: Yes, son, what daughter-in-law, grandchildren, and these are all here.
 Q: What do you think is the happiest thing in your life now is what you are most satisfied with. What are you most satisfied with in your whole state of life?
 Answer: satisfaction is this has children to have a family, have grandson. That grandson even graduated from college and came out to work.

 Q: Generally speaking, I am quite satisfied with my own feelings and my life. Still satisfied? Or not how satisfied?
 A: Satisfied.
 Q: Do you think you are living with your children or not right now?
 A: Live together.
 Q: That means that you think you live now and you say you live with your children, right?
 A: Yes.
 Q: If you live together, do you think it is quite different to live together with the young people? Or do you prefer to live alone or prefer to live with your children to answer: ah. No, no, then you are all the young people are very filial piety.

 Q: Oh, that's good, because many young people are not used to living with the old people. Generally speaking, you feel very comfortable living with them, don't they?
 Answer: That is still very filial piety.
 Q: Are you usually sick, they will accompany you to see a doctor. Will come with you, won't you? For example, what you usually take medicine, they will help you to pay attention to ah or how
A: Yes, yes.
 Q: Ok, that means you think you mean you are generally satisfied, but do you really think about it? Well, from the community, or the government, or from the community or other aspects, you can make your life a little better and happier. Do you have any good advice? What can we do? From the community, ah, or from this medical staff, ah, or from the government, ah, what do you think we can do better. It can improve your satisfaction, improve your satisfaction, improve your life happiness, then you are happier.

 Answer: that generally basically in that community now, that service also generally still can ah.
 Q: Generally speaking, do you think this kind of service in their community is ok in all aspects. Does the government have any care or special care for your elderly people?
 A: Yes, yes, yes, yes,
Q: What are the benefits.

 A: For example, it was in the countryside, ah ah, I live at the age of MAO Zedong era, it was very hard at that time. Yes, yes, that substance is very good, and you will get bored. Yes, that's different now. Since I have been in the countryside since I was 65 years ago, then the government has taken care of the old man. At first, it is 50 yuan, 50 yuan a month after 55 yuan, slowly lift, slowly lift, until now there are more than 200 points.
 Q: Every month, right?
 A: Yes
Q: every month you although you this number in the outside this situation is that proportion is very small, right? But you have so much money so much old people calculate that amount is very powerful. Then you quite understand the government.
 A: Well, yes.
 Q: Do they give you, for example, the usual care is if the community pays you if the government gives you some other benefits. Ask you how well you are. That means to care about you or to check for you. You're not having anything else.

 Answer: it is a family like me, if I also had to apply at that time, is my daughter my son-in-law she is also a civil servant.

 She works in the internal department. Well, if I were ordinary and I wouldn't want that much, I would apply to the disabled government, but I was just getting right, I didn't do that. Well, even a country is not very easy either. You see, the international situation is not good, and this country costs a lot of money.
 A lot of money, if you otherwise, if the international situation is good, we can improve a little, but no way, the world is like this.
 Ask: ah, big ye, you are what work do? I think you understand the government that way,
Answer: I farm by land, farm by oneself.
 Q: Yeah, what did you don't do when you came here in' 97?

 Answer: After doing a small business, take grandchildren.
 Q: Then you just said that you just said that they are still good. But do you feel that from your own point of view, do you as an old person, do you think they can do a little better? To make you even happier? Is there any advice from here?

 A: That's not so demanding. Ok, Ok, thank you for working hard

Q: How old are you aged now?
 A: 90 years old.
 Q: Are you a local person?
 A: My hometown is in Chaoshan. I came to Shenzhen 10 years ago, and now I live in Dongguan. He had three sons and a daughter, and the sons did business, so they moved over with him.
 Q: You look so healthy.
 A: Yes, I always get sick,
Q: But you look very energetic. What are the main diseases?
 A: This heart disease. Heart at first the body is still very good, 73 years old that year car accident, originally I was often often have exercise, this his foot was broken, just stay at home for a year. He started having a bad heart at the end of age 74. Well, the heart is not good. I keep taking that heart medicine. Well. Just eat about four or five years, four or five years later began that stomach is bad, gastric ulcer, slowly chronic gastritis erosion. After this heart also this to this foot influence, began the heart is not good. The heart is not good to take medicine for a long time, the stomach is bad.be not allowed. Now you can't even take that cerebrovascular, nor is the arteriosclerosis.
 Ask: oh, this way, that this is really multiple serious diseases, that you these diseases will usually affect your life a lot, is not you usually affect your own life, or mood ah, family relations, old body health can body body is not healthy trouble. Well, then you think you now the whole of your own life, because these diseases will be your life is generally satisfied?
 A: Generally speaking, it is still ok, right?
 Q: that you and the children their family relationship, and neighbor relationship these all very good?
 A: Generally,
Q: Not so much, right? How you have your children. How many children are there?
 A: Three boys and a girl.
 Q: Oh, four children. Are you a local person or a nonlocal person?
 Answer: I chaoshan come over, how long did you come over? I started working out in Shenzhen when I was 50.
 Q: I came here in 1997. I have been here for a long time. There are still more than ten days, more than 20 years away.
 Q: Well. Oh, if you come here, will you come here for your own business, or.
 A: My son did a little business.
 Q: So you came here with your son, so you came along with you?
 A: Well.
 Q: Oh, so, that's like most of the family population is here,
A: Yes, son, what daughter-in-law, grandchildren, and these are all here.
 Q: What do you think is the happiest thing in your life now is what you are most satisfied with. What are you most satisfied with in your whole state of life?
 Answer: satisfaction is this has children to have a family, have grandson. That grandson even graduated from college and came out to work.

 Q: Generally speaking, I am quite satisfied with my own feelings and my life. Still satisfied? Or not how satisfied?
 A: Satisfied.
 Q: Do you think you are living with your children or not right now?
 A: Live together.
 Q: That means that you think you live now and you say you live with your children, right?
 A: Yes.
 Q: If you live together, do you think it is quite different to live together with the young people? Or do you prefer to live alone or prefer to live with your children to answer: ah. No, no, then you are all the young people are very filial piety.

 Q: Oh, that's good, because many young people are not used to living with the old people. Generally speaking, you feel very comfortable living with them, don't they?
 Answer: That is still very filial piety.
 Q: Are you usually sick, they will accompany you to see a doctor. Will come with you, won't you? For example, what you usually take medicine, they will help you to pay attention to ah or how
A: Yes, yes.
 Q: Ok, that means you think you mean you are generally satisfied, but do you really think about it? Well, from the community, or the government, or from the community or other aspects, you can make your life a little better and happier. Do you have any good advice? What can we do? From the community, ah, or from this medical staff, ah, or from the government, ah, what do you think we can do better. It can improve your satisfaction, improve your satisfaction, improve your life happiness, then you are happier.

 Answer: that generally basically in that community now, that service also generally still can ah.
 Q: Generally speaking, do you think this kind of service in their community is ok in all aspects. Does the government have any care or special care for your elderly people?
 A: Yes, yes, yes, yes,
Q: What are the benefits.

 A: For example, it was in the countryside, ah ah, I live at the age of MAO Zedong era, it was very hard at that time. Yes, yes, that substance is very good, and you will get bored. Yes, that's different now. Since I have been in the countryside since I was 65 years ago, then the government has taken care of the old man. At first, it is 50 yuan, 50 yuan a month after 55 yuan, slowly lift, slowly lift, until now there are more than 200 points.
 Q: Every month, right?
 A: Yes
Q: every month you although you this number in the outside this situation is that proportion is very small, right? But you have so much money so much old people calculate that amount is very powerful. Then you quite understand the government.
 A: Well, yes.
 Q: Do they give you, for example, the usual care is if the community pays you if the government gives you some other benefits. Ask you how well you are. That means to care about you or to check for you. You're not having anything else.

 Answer: it is a family like me, if I also had to apply at that time, is my daughter my son-in-law she is also a civil servant.

 She works in the internal department. Well, if I were ordinary and I wouldn't want that much, I would apply to the disabled government, but I was just getting right, I didn't do that. Well, even a country is not very easy either. You see, the international situation is not good, and this country costs a lot of money.
 A lot of money, if you otherwise, if the international situation is good, we can improve a little, but no way, the world is like this.
 Ask: ah, big ye, you are what work do? I think you understand the government that way,
Answer: I farm by land, farm by oneself.
 Q: Yeah, what did you don't do when you came here in' 97?

 Answer: After doing a small business, take grandchildren.
 Q: Then you just said that you just said that they are still good. But do you feel that from your own point of view, do you as an old person, do you think they can do a little better? To make you even happier? Is there any advice from here?

 A: That's not so demanding. Ok, Ok, thank you for working hard

Q: How old are you aged now?
 A: 81 years old.
 Q: Are you a local person?
 Answer: My hometown is in Chaoshan. I visited Shenzhen 6 years ago, and now I live in Dongguan. He had three sons and a daughter, and the sons did business, so they moved over with him.
 Q: You look so healthy.
 A: Yes, I always get sick,
Q: But you look very energetic. What are the main diseases?
 A: This heart disease. Heart at first the body is still very good, 69 years old that year car accident ah, originally I was often often have exercise ah, this his foot was broken, just stay at home for a year. I started having a bad heart at the end of my 70s. Well, the heart is not good. I keep taking that heart medicine. Well. Just eat about four or five years, four or five years later began that stomach is bad, gastric ulcer, slowly chronic gastritis erosion. After this heart also this to this foot influence, began the heart is not good. The heart is not good to take medicine for a long time, the stomach is bad.be not allowed. Now you can't even take that cerebrovascular, nor is the arteriosclerosis.
 Ask: oh, this way, that this is really multiple serious diseases, that you these diseases will usually affect your life a lot, is not you usually affect your own life, or mood ah, family relations, old body health can body body is not healthy trouble. Well, then you think you now the whole of your own life, because these diseases will be your life is generally satisfied?
 A: Generally speaking, it is still ok, right?
 Q: that you and the children their family relationship, and neighbor relationship these all very good?
 A: Generally,
Q: Not so much, right? How you have your children. How many children are there?
 A: Three boys and a girl.
 Q: Oh, four children. Are you a local person or a nonlocal person?
 Answer: I chaoshan come over, how long did you come over? I started working out in Shenzhen when I was 50.
 Q: I came here in 1997. I have been here for a long time. There are still more than ten days, more than 20 years away.
 Q: Well. Oh, if you come here, will you come here for your own business, or.
 A: My son did a little business.
 Q: So you came here with your son, so you came along with you?
 A: Well.
 Q: Oh, so, that's like most of the family population is here,
A: Yes, son, what daughter-in-law, grandchildren, and these are all here.
 Q: What do you think is the happiest thing in your life now is what you are most satisfied with. What are you most satisfied with in your whole state of life?
 Answer: satisfaction is this has children to have a family, have grandson. That grandson even graduated from college and came out to work.

 Q: Generally speaking, I am quite satisfied with my own feelings and my life. Still satisfied? Or not how satisfied?
 A: Satisfied.
 Q: Do you think you are living with your children or not right now?
 A: Live together.
 Q: That means that you think you live now and you say you live with your children, right?
 A: Yes.
 Q: If you live together, do you think it is quite different to live together with the young people? Or do you prefer to live alone or prefer to live with your children to answer: ah. No, no, then you are all the young people are very filial piety.

 Q: Oh, that's good, because many young people are not used to living with the old people. Generally speaking, you feel very comfortable living with them, don't they?
 Answer: That is still very filial piety.
 Q: Are you usually sick, they will accompany you to see a doctor. Will come with you, won't you? For example, what you usually take medicine, they will help you to pay attention to ah or how
A: Yes, yes.
 Q: Ok, that means you think you mean you are generally satisfied, but do you really think about it? Well, from the community, or the government, or from the community or other aspects, you can make your life a little better and happier. Do you have any good advice? What can we do? From the community, ah, or from this medical staff, ah, or from the government, ah, what do you think we can do better. It can improve your satisfaction, improve your satisfaction, improve your life happiness, then you are happier.

 Answer: that generally basically in that community now, that service also generally still can ah.
 Q: Generally speaking, do you think this kind of service in their community is ok in all aspects. Does the government have any care or special care for your elderly people?
 A: Yes, yes, yes, yes,
Q: What are the benefits.

 A: For example, it was in the countryside, ah ah, I live at the age of MAO Zedong era, it was very hard at that time. Yes, yes, that substance is very good, and you will get bored. Yes, that's different now. Since I have been in the countryside since I was 65 years ago, then the government has taken care of the old man. At first, it is 50 yuan, 50 yuan a month after 55 yuan, slowly lift, slowly lift, until now there are more than 200 points.
 Q: Every month, right?
 A: Yes
Q: every month you although you this number in the outside this situation is that proportion is very small, right? But you have so much money so much old people calculate that amount is very powerful. Then you quite understand the government.
 A: Well, yes.
 Q: Do they give you, for example, the usual care is if the community pays you if the government gives you some other benefits. Ask you how well you are. That means to care about you or to check for you. You're not having anything else.

 Answer: it is a family like me, if I also had to apply at that time, is my daughter my son-in-law she is also a civil servant.

 She works in the internal department. Well, if I were ordinary and I wouldn't want that much, I would apply to the disabled government, but I was just getting right, I didn't do that. Well, even a country is not very easy either. You see, the international situation is not good, and this country costs a lot of money.
 A lot of money, if you otherwise, if the international situation is good, we can improve a little, but no way, the world is like this.
 Ask: ah, big ye, you are what work do? I think you understand the government that way,
Answer: I farm by land, farm by oneself.
 Q: Yeah, what did you don't do when you came here in' 97?

 Answer: After doing a small business, take grandchildren.
 Q: Then you just said that you just said that they are still good. But do you feel that from your own point of view, do you as an old person, do you think they can do a little better? To make you even happier? Is there any advice from here?

 A: That's not so demanding. Ok, Ok, thank you for working hard

Q: How old are you aged now?
 A: 82 years old.
 Q: Are you a local person?
 Answer: My hometown is in Chaoshan. I visited Shenzhen 7 years ago, and now I live in Dongguan. He had three sons and a daughter, and the sons did business, so they moved over with him.
 Q: You look so healthy.
 A: Yes, I always get sick,
Q: But you look very energetic. What are the main diseases?
 A: This heart disease. Heart at first the body is still very good, 70 years old that year car accident, originally I was often often have exercise, this his foot broke, just stay at home for a year. I started having a bad heart at the end of age 71. Well, the heart is not good. I keep taking that heart medicine. Well. Just eat about four or five years, four or five years later began that stomach is bad, gastric ulcer, slowly chronic gastritis erosion. After this heart also this to this foot influence, began the heart is not good. The heart is not good to take medicine for a long time, the stomach is bad.be not allowed. Now you can't even take that cerebrovascular, nor is the arteriosclerosis.
 Ask: oh, this way, that this is really multiple serious diseases, that you these diseases will usually affect your life a lot, is not you usually affect your own life, or mood ah, family relations, old body health can body body is not healthy trouble. Well, then you think you now the whole of your own life, because these diseases will be your life is generally satisfied?
 A: Generally speaking, it is still ok, right?
 Q: that you and the children their family relationship, and neighbor relationship these all very good?
 A: Generally,
Q: Not so much, right? How you have your children. How many children are there?
 A: Three boys and a girl.
 Q: Oh, four children. Are you a local person or a nonlocal person?
 Answer: I chaoshan come over, how long did you come over? I started working out in Shenzhen when I was 50.
 Q: I came here in 1997. I have been here for a long time. There are still more than ten days, more than 20 years away.
 Q: Well. Oh, if you come here, will you come here for your own business, or.
 A: My son did a little business.
 Q: So you came here with your son, so you came along with you?
 A: Well.
 Q: Oh, so, that's like most of the family population is here,
A: Yes, son, what daughter-in-law, grandchildren, and these are all here.
 Q: What do you think is the happiest thing in your life now is what you are most satisfied with. What are you most satisfied with in your whole state of life?
 Answer: satisfaction is this has children to have a family, have grandson. That grandson even graduated from college and came out to work.

 Q: Generally speaking, I am quite satisfied with my own feelings and my life. Still satisfied? Or not how satisfied?
 A: Satisfied.
 Q: Do you think you are living with your children or not right now?
 A: Live together.
 Q: That means that you think you live now and you say you live with your children, right?
 A: Yes.
 Q: If you live together, do you think it is quite different to live together with the young people? Or do you prefer to live alone or prefer to live with your children to answer: ah. No, no, then you are all the young people are very filial piety.

 Q: Oh, that's good, because many young people are not used to living with the old people. Generally speaking, you feel very comfortable living with them, don't they?
 Answer: That is still very filial piety.
 Q: Are you usually sick, they will accompany you to see a doctor. Will come with you, won't you? For example, what you usually take medicine, they will help you to pay attention to ah or how
A: Yes, yes.
 Q: Ok, that means you think you mean you are generally satisfied, but do you really think about it? Well, from the community, or the government, or from the community or other aspects, you can make your life a little better and happier. Do you have any good advice? What can we do? From the community, ah, or from this medical staff, ah, or from the government, ah, what do you think we can do better. It can improve your satisfaction, improve your satisfaction, improve your life happiness, then you are happier.

 Answer: that generally basically in that community now, that service also generally still can ah.
 Q: Generally speaking, do you think this kind of service in their community is ok in all aspects. Does the government have any care or special care for your elderly people?
 A: Yes, yes, yes, yes,
Q: What are the benefits.

 A: For example, it was in the countryside, ah ah, I live at the age of MAO Zedong era, it was very hard at that time. Yes, yes, that substance is very good, and you will get bored. Yes, that's different now. Since I have been in the countryside since I was 65 years ago, then the government has taken care of the old man. At first, it is 50 yuan, 50 yuan a month after 55 yuan, slowly lift, slowly lift, until now there are more than 200 points.
 Q: Every month, right?
 A: Yes
Q: every month you although you this number in the outside this situation is that proportion is very small, right? But you have so much money so much old people calculate that amount is very powerful. Then you quite understand the government.
 A: Well, yes.
 Q: Do they give you, for example, the usual care is if the community pays you if the government gives you some other benefits. Ask you how well you are. That means to care about you or to check for you. You're not having anything else.

 Answer: it is a family like me, if I also had to apply at that time, is my daughter my son-in-law she is also a civil servant.

 She works in the internal department. Well, if I were ordinary and I wouldn't want that much, I would apply to the disabled government, but I was just getting right, I didn't do that. Well, even a country is not very easy either. You see, the international situation is not good, and this country costs a lot of money.
 A lot of money, if you otherwise, if the international situation is good, we can improve a little, but no way, the world is like this.
 Ask: ah, big ye, you are what work do? I think you understand the government that way,
Answer: I farm by land, farm by oneself.
 Q: Yeah, what did you don't do when you came here in' 97?

 Answer: After doing a small business, take grandchildren.
 Q: Then you just said that you just said that they are still good. But do you feel that from your own point of view, do you as an old person, do you think they can do a little better? To make you even happier? Is there any advice from here?

 A: That's not so demanding. Ok, Ok, thank you for working hard

Q: How old are you aged now?
 A: 83 years old.
 Q: Are you a local person?
 A: My hometown is in Chaoshan. I visited Shenzhen 8 years ago, and now I live in Dongguan. He had three sons and a daughter, and the sons did business, so they moved over with him.
 Q: You look so healthy.
 A: Yes, I always get sick,
Q: But you look very energetic. What are the main diseases?
 A: This heart disease. Heart at first the body is still very good, 71 years old that year car accident ah, originally I was often often have exercise ah, this his foot broke, just stay at home for a year. He started having a bad heart at the end of age 72. Well, the heart is not good. I keep taking that heart medicine. Well. Just eat about four or five years, four or five years later began that stomach is bad, gastric ulcer, slowly chronic gastritis erosion. After this heart also this to this foot influence, began the heart is not good. The heart is not good to take medicine for a long time, the stomach is bad.be not allowed. Now you can't even take that cerebrovascular, nor is the arteriosclerosis.
 Ask: oh, this way, that this is really multiple serious diseases, that you these diseases will usually affect your life a lot, is not you usually affect your own life, or mood ah, family relations, old body health can body body is not healthy trouble. Well, then you think you now the whole of your own life, because these diseases will be your life is generally satisfied?
 A: Generally speaking, it is still ok, right?
 Q: that you and the children their family relationship, and neighbor relationship these all very good?
 A: Generally,
Q: Not so much, right? How you have your children. How many children are there?
 A: Three boys and a girl.
 Q: Oh, four children. Are you a local person or a nonlocal person?
 Answer: I chaoshan come over, how long did you come over? I started working out in Shenzhen when I was 50.
 Q: I came here in 1997. I have been here for a long time. There are still more than ten days, more than 20 years away.
 Q: Well. Oh, if you come here, will you come here for your own business, or.
 A: My son did a little business.
 Q: So you came here with your son, so you came along with you?
 A: Well.
 Q: Oh, so, that's like most of the family population is here,
A: Yes, son, what daughter-in-law, grandchildren, and these are all here.
 Q: What do you think is the happiest thing in your life now is what you are most satisfied with. What are you most satisfied with in your whole state of life?
 Answer: satisfaction is this has children to have a family, have grandson. That grandson even graduated from college and came out to work.

 Q: Generally speaking, I am quite satisfied with my own feelings and my life. Still satisfied? Or not how satisfied?
 A: Satisfied.
 Q: Do you think you are living with your children or not right now?
 A: Live together.
 Q: That means that you think you live now and you say you live with your children, right?
 A: Yes.
 Q: If you live together, do you think it is quite different to live together with the young people? Or do you prefer to live alone or prefer to live with your children to answer: ah. No, no, then you are all the young people are very filial piety.

 Q: Oh, that's good, because many young people are not used to living with the old people. Generally speaking, you feel very comfortable living with them, don't they?
 Answer: That is still very filial piety.
 Q: Are you usually sick, they will accompany you to see a doctor. Will come with you, won't you? For example, what you usually take medicine, they will help you to pay attention to ah or how
A: Yes, yes.
 Q: Ok, that means you think you mean you are generally satisfied, but do you really think about it? Well, from the community, or the government, or from the community or other aspects, you can make your life a little better and happier. Do you have any good advice? What can we do? From the community, ah, or from this medical staff, ah, or from the government, ah, what do you think we can do better. It can improve your satisfaction, improve your satisfaction, improve your life happiness, then you are happier.

 Answer: that generally basically in that community now, that service also generally still can ah.
 Q: Generally speaking, do you think this kind of service in their community is ok in all aspects. Does the government have any care or special care for your elderly people?
 A: Yes, yes, yes, yes,
Q: What are the benefits.

 A: For example, it was in the countryside, ah ah, I live at the age of MAO Zedong era, it was very hard at that time. Yes, yes, that substance is very good, and you will get bored. Yes, that's different now. Since I have been in the countryside since I was 65 years ago, then the government has taken care of the old man. At first, it is 50 yuan, 50 yuan a month after 55 yuan, slowly lift, slowly lift, until now there are more than 200 points.
 Q: Every month, right?
 A: Yes
Q: every month you although you this number in the outside this situation is that proportion is very small, right? But you have so much money so much old people calculate that amount is very powerful. Then you quite understand the government.
 A: Well, yes.
 Q: Do they give you, for example, the usual care is if the community pays you if the government gives you some other benefits. Ask you how well you are. That means to care about you or to check for you. You're not having anything else.

 Answer: it is a family like me, if I also had to apply at that time, is my daughter my son-in-law she is also a civil servant.

 She works in the internal department. Well, if I were ordinary and I wouldn't want that much, I would apply to the disabled government, but I was just getting right, I didn't do that. Well, even a country is not very easy either. You see, the international situation is not good, and this country costs a lot of money.
 A lot of money, if you otherwise, if the international situation is good, we can improve a little, but no way, the world is like this.
 Ask: ah, big ye, you are what work do? I think you understand the government that way,
Answer: I farm by land, farm by oneself.
 Q: Yeah, what did you don't do when you came here in' 97?

 Answer: After doing a small business, take grandchildren.
 Q: Then you just said that you just said that they are still good. But do you feel that from your own point of view, do you as an old person, do you think they can do a little better? To make you even happier? Is there any advice from here?

 A: That's not so demanding. Ok, Ok, thank you for working hard

Q: How old are you aged now?
 A: 84 years old.
 Q: Are you a local person?
 A: My hometown is in Chaoshan. I visited Shenzhen 9 years ago, and now I live in Dongguan. He had three sons and a daughter, and the sons did business, so they moved over with him.
 Q: You look so healthy.
 A: Yes, I always get sick,
Q: But you look very energetic. What are the main diseases?
 A: This heart disease. Heart at first the body is very good, 72 years old that year car accident, originally I was often often have exercise, this his foot broken, just stay at home for a year. He started having a bad heart at the end of age 73. Well, the heart is not good. I keep taking that heart medicine. Well. Just eat about four or five years, four or five years later began that stomach is bad, gastric ulcer, slowly chronic gastritis erosion. After this heart also this to this foot influence, began the heart is not good. The heart is not good to take medicine for a long time, the stomach is bad.be not allowed. Now you can't even take that cerebrovascular, nor is the arteriosclerosis.
 Ask: oh, this way, that this is really multiple serious diseases, that you these diseases will usually affect your life a lot, is not you usually affect your own life, or mood ah, family relations, old body health can body body is not healthy trouble. Well, then you think you now the whole of your own life, because these diseases will be your life is generally satisfied?
 A: Generally speaking, it is still ok, right?
 Q: that you and the children their family relationship, and neighbor relationship these all very good?
 A: Generally,
Q: Not so much, right? How you have your children. How many children are there?
 A: Three boys and a girl.
 Q: Oh, four children. Are you a local person or a nonlocal person?
 Answer: I chaoshan come over, how long did you come over? I started working out in Shenzhen when I was 50.
 Q: I came here in 1997. I have been here for a long time. There are still more than ten days, more than 20 years away.
 Q: Well. Oh, if you come here, will you come here for your own business, or.
 A: My son did a little business.
 Q: So you came here with your son, so you came along with you?
 A: Well.
 Q: Oh, so, that's like most of the family population is here,
A: Yes, son, what daughter-in-law, grandchildren, and these are all here.
 Q: What do you think is the happiest thing in your life now is what you are most satisfied with. What are you most satisfied with in your whole state of life?
 Answer: satisfaction is this has children to have a family, have grandson. That grandson even graduated from college and came out to work.

 Q: Generally speaking, I am quite satisfied with my own feelings and my life. Still satisfied? Or not how satisfied?
 A: Satisfied.
 Q: Do you think you are living with your children or not right now?
 A: Live together.
 Q: That means that you think you live now and you say you live with your children, right?
 A: Yes.
 Q: If you live together, do you think it is quite different to live together with the young people? Or do you prefer to live alone or prefer to live with your children to answer: ah. No, no, then you are all the young people are very filial piety.

 Q: Oh, that's good, because many young people are not used to living with the old people. Generally speaking, you feel very comfortable living with them, don't they?
 Answer: That is still very filial piety.
 Q: Are you usually sick, they will accompany you to see a doctor. Will come with you, won't you? For example, what you usually take medicine, they will help you to pay attention to ah or how
A: Yes, yes.
 Q: Ok, that means you think you mean you are generally satisfied, but do you really think about it? Well, from the community, or the government, or from the community or other aspects, you can make your life a little better and happier. Do you have any good advice? What can we do? From the community, ah, or from this medical staff, ah, or from the government, ah, what do you think we can do better. It can improve your satisfaction, improve your satisfaction, improve your life happiness, then you are happier.

 Answer: that generally basically in that community now, that service also generally still can ah.
 Q: Generally speaking, do you think this kind of service in their community is ok in all aspects. Does the government have any care or special care for your elderly people?
 A: Yes, yes, yes, yes,
Q: What are the benefits.

 A: For example, it was in the countryside, ah ah, I live at the age of MAO Zedong era, it was very hard at that time. Yes, yes, that substance is very good, and you will get bored. Yes, that's different now. Since I have been in the countryside since I was 65 years ago, then the government has taken care of the old man. At first, it is 50 yuan, 50 yuan a month after 55 yuan, slowly lift, slowly lift, until now there are more than 200 points.
 Q: Every month, right?
 A: Yes
Q: every month you although you this number in the outside this situation is that proportion is very small, right? But you have so much money so much old people calculate that amount is very powerful. Then you quite understand the government.
 A: Well, yes.
 Q: Do they give you, for example, the usual care is if the community pays you if the government gives you some other benefits. Ask you how well you are. That means to care about you or to check for you. You're not having anything else.

 Answer: it is a family like me, if I also had to apply at that time, is my daughter my son-in-law she is also a civil servant.

 She works in the internal department. Well, if I were ordinary and I wouldn't want that much, I would apply to the disabled government, but I was just getting right, I didn't do that. Well, even a country is not very easy either. You see, the international situation is not good, and this country costs a lot of money.
 A lot of money, if you otherwise, if the international situation is good, we can improve a little, but no way, the world is like this.
 Ask: ah, big ye, you are what work do? I think you understand the government that way,
Answer: I farm by land, farm by oneself.
 Q: Yeah, what did you don't do when you came here in' 97?

 Answer: After doing a small business, take grandchildren.
 Q: Then you just said that you just said that they are still good. But do you feel that from your own point of view, do you as an old person, do you think they can do a little better? To make you even happier? Is there any advice from here?

 A: That's not so demanding. Ok, Ok, thank you for working hard

Q: How old are you aged now?
 A: 85 years old.
 Q: Are you a local person?
 A: My hometown is in Chaoshan. I came to Shenzhen 10 years ago, and now I live in Dongguan. He had three sons and a daughter, and the sons did business, so they moved over with him.
 Q: You look so healthy.
 A: Yes, I always get sick,
Q: But you look very energetic. What are the main diseases?
 A: This heart disease. Heart at first the body is still very good, 73 years old that year car accident, originally I was often often have exercise, this his foot was broken, just stay at home for a year. He started having a bad heart at the end of age 74. Well, the heart is not good. I keep taking that heart medicine. Well. Just eat about four or five years, four or five years later began that stomach is bad, gastric ulcer, slowly chronic gastritis erosion. After this heart also this to this foot influence, began the heart is not good. The heart is not good to take medicine for a long time, the stomach is bad.be not allowed. Now you can't even take that cerebrovascular, nor is the arteriosclerosis.
 Ask: oh, this way, that this is really multiple serious diseases, that you these diseases will usually affect your life a lot, is not you usually affect your own life, or mood ah, family relations, old body health can body body is not healthy trouble. Well, then you think you now the whole of your own life, because these diseases will be your life is generally satisfied?
 A: Generally speaking, it is still ok, right?
 Q: that you and the children their family relationship, and neighbor relationship these all very good?
 A: Generally,
Q: Not so much, right? How you have your children. How many children are there?
 A: Three boys and a girl.
 Q: Oh, four children. Are you a local person or a nonlocal person?
 Answer: I chaoshan come over, how long did you come over? I started working out in Shenzhen when I was 50.
 Q: I came here in 1997. I have been here for a long time. There are still more than ten days, more than 20 years away.
 Q: Well. Oh, if you come here, will you come here for your own business, or.
 A: My son did a little business.
 Q: So you came here with your son, so you came along with you?
 A: Well.
 Q: Oh, so, that's like most of the family population is here,
A: Yes, son, what daughter-in-law, grandchildren, and these are all here.
 Q: What do you think is the happiest thing in your life now is what you are most satisfied with. What are you most satisfied with in your whole state of life?
 Answer: satisfaction is this has children to have a family, have grandson. That grandson even graduated from college and came out to work.

 Q: Generally speaking, I am quite satisfied with my own feelings and my life. Still satisfied? Or not how satisfied?
 A: Satisfied.
 Q: Do you think you are living with your children or not right now?
 A: Live together.
 Q: That means that you think you live now and you say you live with your children, right?
 A: Yes.
 Q: If you live together, do you think it is quite different to live together with the young people? Or do you prefer to live alone or prefer to live with your children to answer: ah. No, no, then you are all the young people are very filial piety.

 Q: Oh, that's good, because many young people are not used to living with the old people. Generally speaking, you feel very comfortable living with them, don't they?
 Answer: That is still very filial piety.
 Q: Are you usually sick, they will accompany you to see a doctor. Will come with you, won't you? For example, what you usually take medicine, they will help you to pay attention to ah or how
A: Yes, yes.
 Q: Ok, that means you think you mean you are generally satisfied, but do you really think about it? Well, from the community, or the government, or from the community or other aspects, you can make your life a little better and happier. Do you have any good advice? What can we do? From the community, ah, or from this medical staff, ah, or from the government, ah, what do you think we can do better. It can improve your satisfaction, improve your satisfaction, improve your life happiness, then you are happier.

 Answer: that generally basically in that community now, that service also generally still can ah.
 Q: Generally speaking, do you think this kind of service in their community is ok in all aspects. Does the government have any care or special care for your elderly people?
 A: Yes, yes, yes, yes,
Q: What are the benefits.

 A: For example, it was in the countryside, ah ah, I live at the age of MAO Zedong era, it was very hard at that time. Yes, yes, that substance is very good, and you will get bored. Yes, that's different now. Since I have been in the countryside since I was 65 years ago, then the government has taken care of the old man. At first, it is 50 yuan, 50 yuan a month after 55 yuan, slowly lift, slowly lift, until now there are more than 200 points.
 Q: Every month, right?
 A: Yes
Q: every month you although you this number in the outside this situation is that proportion is very small, right? But you have so much money so much old people calculate that amount is very powerful. Then you quite understand the government.
 A: Well, yes.
 Q: Do they give you, for example, the usual care is if the community pays you if the government gives you some other benefits. Ask you how well you are. That means to care about you or to check for you. You're not having anything else.

 Answer: it is a family like me, if I also had to apply at that time, is my daughter my son-in-law she is also a civil servant.

 She works in the internal department. Well, if I were ordinary and I wouldn't want that much, I would apply to the disabled government, but I was just getting right, I didn't do that. Well, even a country is not very easy either. You see, the international situation is not good, and this country costs a lot of money.
 A lot of money, if you otherwise, if the international situation is good, we can improve a little, but no way, the world is like this.
 Ask: ah, big ye, you are what work do? I think you understand the government that way,
Answer: I farm by land, farm by oneself.
 Q: Yeah, what did you don't do when you came here in' 97?

 Answer: After doing a small business, take grandchildren.
 Q: Then you just said that you just said that they are still good. But do you feel that from your own point of view, do you as an old person, do you think they can do a little better? To make you even happier? Is there any advice from here?

 A: That's not so demanding. Ok, Ok, thank you for working hard

Q: How old are you aged now?
 A: 86 years old.
 Q: Are you a local person?
 Answer: My hometown is in Chaoshan. I visited Shenzhen 6 years ago, and now I live in Dongguan. He had three sons and a daughter, and the sons did business, so they moved over with him.
 Q: You look so healthy.
 A: Yes, I always get sick,
Q: But you look very energetic. What are the main diseases?
 A: This heart disease. Heart at first the body is still very good, 69 years old that year car accident ah, originally I was often often have exercise ah, this his foot was broken, just stay at home for a year. I started having a bad heart at the end of my 70s. Well, the heart is not good. I keep taking that heart medicine. Well. Just eat about four or five years, four or five years later began that stomach is bad, gastric ulcer, slowly chronic gastritis erosion. After this heart also this to this foot influence, began the heart is not good. The heart is not good to take medicine for a long time, the stomach is bad.be not allowed. Now you can't even take that cerebrovascular, nor is the arteriosclerosis.
 Ask: oh, this way, that this is really multiple serious diseases, that you these diseases will usually affect your life a lot, is not you usually affect your own life, or mood ah, family relations, old body health can body body is not healthy trouble. Well, then you think you now the whole of your own life, because these diseases will be your life is generally satisfied?
 A: Generally speaking, it is still ok, right?
 Q: that you and the children their family relationship, and neighbor relationship these all very good?
 A: Generally,
Q: Not so much, right? How you have your children. How many children are there?
 A: Three boys and a girl.
 Q: Oh, four children. Are you a local person or a nonlocal person?
 Answer: I chaoshan come over, how long did you come over? I started working out in Shenzhen when I was 50.
 Q: I came here in 1997. I have been here for a long time. There are still more than ten days, more than 20 years away.
 Q: Well. Oh, if you come here, will you come here for your own business, or.
 A: My son did a little business.
 Q: So you came here with your son, so you came along with you?
 A: Well.
 Q: Oh, so, that's like most of the family population is here,
A: Yes, son, what daughter-in-law, grandchildren, and these are all here.
 Q: What do you think is the happiest thing in your life now is what you are most satisfied with. What are you most satisfied with in your whole state of life?
 Answer: satisfaction is this has children to have a family, have grandson. That grandson even graduated from college and came out to work.

 Q: Generally speaking, I am quite satisfied with my own feelings and my life. Still satisfied? Or not how satisfied?
 A: Satisfied.
 Q: Do you think you are living with your children or not right now?
 A: Live together.
 Q: That means that you think you live now and you say you live with your children, right?
 A: Yes.
 Q: If you live together, do you think it is quite different to live together with the young people? Or do you prefer to live alone or prefer to live with your children to answer: ah. No, no, then you are all the young people are very filial piety.

 Q: Oh, that's good, because many young people are not used to living with the old people. Generally speaking, you feel very comfortable living with them, don't they?
 Answer: That is still very filial piety.
 Q: Are you usually sick, they will accompany you to see a doctor. Will come with you, won't you? For example, what you usually take medicine, they will help you to pay attention to ah or how
A: Yes, yes.
 Q: Ok, that means you think you mean you are generally satisfied, but do you really think about it? Well, from the community, or the government, or from the community or other aspects, you can make your life a little better and happier. Do you have any good advice? What can we do? From the community, ah, or from this medical staff, ah, or from the government, ah, what do you think we can do better. It can improve your satisfaction, improve your satisfaction, improve your life happiness, then you are happier.

 Answer: that generally basically in that community now, that service also generally still can ah.
 Q: Generally speaking, do you think this kind of service in their community is ok in all aspects. Does the government have any care or special care for your elderly people?
 A: Yes, yes, yes, yes,
Q: What are the benefits.

 A: For example, it was in the countryside, ah ah, I live at the age of MAO Zedong era, it was very hard at that time. Yes, yes, that substance is very good, and you will get bored. Yes, that's different now. Since I have been in the countryside since I was 65 years ago, then the government has taken care of the old man. At first, it is 50 yuan, 50 yuan a month after 55 yuan, slowly lift, slowly lift, until now there are more than 200 points.
 Q: Every month, right?
 A: Yes
Q: every month you although you this number in the outside this situation is that proportion is very small, right? But you have so much money so much old people calculate that amount is very powerful. Then you quite understand the government.
 A: Well, yes.
 Q: Do they give you, for example, the usual care is if the community pays you if the government gives you some other benefits. Ask you how well you are. That means to care about you or to check for you. You're not having anything else.

 Answer: it is a family like me, if I also had to apply at that time, is my daughter my son-in-law she is also a civil servant.

 She works in the internal department. Well, if I were ordinary and I wouldn't want that much, I would apply to the disabled government, but I was just getting right, I didn't do that. Well, even a country is not very easy either. You see, the international situation is not good, and this country costs a lot of money.
 A lot of money, if you otherwise, if the international situation is good, we can improve a little, but no way, the world is like this.
 Ask: ah, big ye, you are what work do? I think you understand the government that way,
Answer: I farm by land, farm by oneself.
 Q: Yeah, what did you don't do when you came here in' 97?

 Answer: After doing a small business, take grandchildren.
 Q: Then you just said that you just said that they are still good. But do you feel that from your own point of view, do you as an old person, do you think they can do a little better? To make you even happier? Is there any advice from here?

 A: That's not so demanding. Ok, Ok, thank you for working hard

Q: How old are you aged now?
 A: 87 years old.
 Q: Are you a local person?
 Answer: My hometown is in Chaoshan. I visited Shenzhen 7 years ago, and now I live in Dongguan. He had three sons and a daughter, and the sons did business, so they moved over with him.
 Q: You look so healthy.
 A: Yes, I always get sick,
Q: But you look very energetic. What are the main diseases?
 A: This heart disease. Heart at first the body is still very good, 70 years old that year car accident, originally I was often often have exercise, this his foot broke, just stay at home for a year. I started having a bad heart at the end of age 71. Well, the heart is not good. I keep taking that heart medicine. Well. Just eat about four or five years, four or five years later began that stomach is bad, gastric ulcer, slowly chronic gastritis erosion. After this heart also this to this foot influence, began the heart is not good. The heart is not good to take medicine for a long time, the stomach is bad.be not allowed. Now you can't even take that cerebrovascular, nor is the arteriosclerosis.
 Ask: oh, this way, that this is really multiple serious diseases, that you these diseases will usually affect your life a lot, is not you usually affect your own life, or mood ah, family relations, old body health can body body is not healthy trouble. Well, then you think you now the whole of your own life, because these diseases will be your life is generally satisfied?
 A: Generally speaking, it is still ok, right?
 Q: that you and the children their family relationship, and neighbor relationship these all very good?
 A: Generally,
Q: Not so much, right? How you have your children. How many children are there?
 A: Three boys and a girl.
 Q: Oh, four children. Are you a local person or a nonlocal person?
 Answer: I chaoshan come over, how long did you come over? I started working out in Shenzhen when I was 50.
 Q: I came here in 1997. I have been here for a long time. There are still more than ten days, more than 20 years away.
 Q: Well. Oh, if you come here, will you come here for your own business, or.
 A: My son did a little business.
 Q: So you came here with your son, so you came along with you?
 A: Well.
 Q: Oh, so, that's like most of the family population is here,
A: Yes, son, what daughter-in-law, grandchildren, and these are all here.
 Q: What do you think is the happiest thing in your life now is what you are most satisfied with. What are you most satisfied with in your whole state of life?
 Answer: satisfaction is this has children to have a family, have grandson. That grandson even graduated from college and came out to work.

 Q: Generally speaking, I am quite satisfied with my own feelings and my life. Still satisfied? Or not how satisfied?
 A: Satisfied.
 Q: Do you think you are living with your children or not right now?
 A: Live together.
 Q: That means that you think you live now and you say you live with your children, right?
 A: Yes.
 Q: If you live together, do you think it is quite different to live together with the young people? Or do you prefer to live alone or prefer to live with your children to answer: ah. No, no, then you are all the young people are very filial piety.

 Q: Oh, that's good, because many young people are not used to living with the old people. Generally speaking, you feel very comfortable living with them, don't they?
 Answer: That is still very filial piety.
 Q: Are you usually sick, they will accompany you to see a doctor. Will come with you, won't you? For example, what you usually take medicine, they will help you to pay attention to ah or how
A: Yes, yes.
 Q: Ok, that means you think you mean you are generally satisfied, but do you really think about it? Well, from the community, or the government, or from the community or other aspects, you can make your life a little better and happier. Do you have any good advice? What can we do? From the community, ah, or from this medical staff, ah, or from the government, ah, what do you think we can do better. It can improve your satisfaction, improve your satisfaction, improve your life happiness, then you are happier.

 Answer: that generally basically in that community now, that service also generally still can ah.
 Q: Generally speaking, do you think this kind of service in their community is ok in all aspects. Does the government have any care or special care for your elderly people?
 A: Yes, yes, yes, yes,
Q: What are the benefits.

 A: For example, it was in the countryside, ah ah, I live at the age of MAO Zedong era, it was very hard at that time. Yes, yes, that substance is very good, and you will get bored. Yes, that's different now. Since I have been in the countryside since I was 65 years ago, then the government has taken care of the old man. At first, it is 50 yuan, 50 yuan a month after 55 yuan, slowly lift, slowly lift, until now there are more than 200 points.
 Q: Every month, right?
 A: Yes
Q: every month you although you this number in the outside this situation is that proportion is very small, right? But you have so much money so much old people calculate that amount is very powerful. Then you quite understand the government.
 A: Well, yes.
 Q: Do they give you, for example, the usual care is if the community pays you if the government gives you some other benefits. Ask you how well you are. That means to care about you or to check for you. You're not having anything else.

 Answer: it is a family like me, if I also had to apply at that time, is my daughter my son-in-law she is also a civil servant.

 She works in the internal department. Well, if I were ordinary and I wouldn't want that much, I would apply to the disabled government, but I was just getting right, I didn't do that. Well, even a country is not very easy either. You see, the international situation is not good, and this country costs a lot of money.
 A lot of money, if you otherwise, if the international situation is good, we can improve a little, but no way, the world is like this.
 Ask: ah, big ye, you are what work do? I think you understand the government that way,
Answer: I farm by land, farm by oneself.
 Q: Yeah, what did you don't do when you came here in' 97?

 Answer: After doing a small business, take grandchildren.
 Q: Then you just said that you just said that they are still good. But do you feel that from your own point of view, do you as an old person, do you think they can do a little better? To make you even happier? Is there any advice from here?

 A: That's not so demanding. Ok, Ok, thank you for working hard

Q: How old are you aged now?
 A: 88 years old.
 Q: Are you a local person?
 A: My hometown is in Chaoshan. I visited Shenzhen 8 years ago, and now I live in Dongguan. He had three sons and a daughter, and the sons did business, so they moved over with him.
 Q: You look so healthy.
 A: Yes, I always get sick,
Q: But you look very energetic. What are the main diseases?
 A: This heart disease. Heart at first the body is still very good, 71 years old that year car accident ah, originally I was often often have exercise ah, this his foot broke, just stay at home for a year. He started having a bad heart at the end of age 72. Well, the heart is not good. I keep taking that heart medicine. Well. Just eat about four or five years, four or five years later began that stomach is bad, gastric ulcer, slowly chronic gastritis erosion. After this heart also this to this foot influence, began the heart is not good. The heart is not good to take medicine for a long time, the stomach is bad.be not allowed. Now you can't even take that cerebrovascular, nor is the arteriosclerosis.
 Ask: oh, this way, that this is really multiple serious diseases, that you these diseases will usually affect your life a lot, is not you usually affect your own life, or mood ah, family relations, old body health can body body is not healthy trouble. Well, then you think you now the whole of your own life, because these diseases will be your life is generally satisfied?
 A: Generally speaking, it is still ok, right?
 Q: that you and the children their family relationship, and neighbor relationship these all very good?
 A: Generally,
Q: Not so much, right? How you have your children. How many children are there?
 A: Three boys and a girl.
 Q: Oh, four children. Are you a local person or a nonlocal person?
 Answer: I chaoshan come over, how long did you come over? I started working out in Shenzhen when I was 50.
 Q: I came here in 1997. I have been here for a long time. There are still more than ten days, more than 20 years away.
 Q: Well. Oh, if you come here, will you come here for your own business, or.
 A: My son did a little business.
 Q: So you came here with your son, so you came along with you?
 A: Well.
 Q: Oh, so, that's like most of the family population is here,
A: Yes, son, what daughter-in-law, grandchildren, and these are all here.
 Q: What do you think is the happiest thing in your life now is what you are most satisfied with. What are you most satisfied with in your whole state of life?
 Answer: satisfaction is this has children to have a family, have grandson. That grandson even graduated from college and came out to work.

 Q: Generally speaking, I am quite satisfied with my own feelings and my life. Still satisfied? Or not how satisfied?
 A: Satisfied.
 Q: Do you think you are living with your children or not right now?
 A: Live together.
 Q: That means that you think you live now and you say you live with your children, right?
 A: Yes.
 Q: If you live together, do you think it is quite different to live together with the young people? Or do you prefer to live alone or prefer to live with your children to answer: ah. No, no, then you are all the young people are very filial piety.

 Q: Oh, that's good, because many young people are not used to living with the old people. Generally speaking, you feel very comfortable living with them, don't they?
 Answer: That is still very filial piety.
 Q: Are you usually sick, they will accompany you to see a doctor. Will come with you, won't you? For example, what you usually take medicine, they will help you to pay attention to ah or how
A: Yes, yes.
 Q: Ok, that means you think you mean you are generally satisfied, but do you really think about it? Well, from the community, or the government, or from the community or other aspects, you can make your life a little better and happier. Do you have any good advice? What can we do? From the community, ah, or from this medical staff, ah, or from the government, ah, what do you think we can do better. It can improve your satisfaction, improve your satisfaction, improve your life happiness, then you are happier.

 Answer: that generally basically in that community now, that service also generally still can ah.
 Q: Generally speaking, do you think this kind of service in their community is ok in all aspects. Does the government have any care or special care for your elderly people?
 A: Yes, yes, yes, yes,
Q: What are the benefits.

 A: For example, it was in the countryside, ah ah, I live at the age of MAO Zedong era, it was very hard at that time. Yes, yes, that substance is very good, and you will get bored. Yes, that's different now. Since I have been in the countryside since I was 65 years ago, then the government has taken care of the old man. At first, it is 50 yuan, 50 yuan a month after 55 yuan, slowly lift, slowly lift, until now there are more than 200 points.
 Q: Every month, right?
 A: Yes
Q: every month you although you this number in the outside this situation is that proportion is very small, right? But you have so much money so much old people calculate that amount is very powerful. Then you quite understand the government.
 A: Well, yes.
 Q: Do they give you, for example, the usual care is if the community pays you if the government gives you some other benefits. Ask you how well you are. That means to care about you or to check for you. You're not having anything else.

 Answer: it is a family like me, if I also had to apply at that time, is my daughter my son-in-law she is also a civil servant.

 She works in the internal department. Well, if I were ordinary and I wouldn't want that much, I would apply to the disabled government, but I was just getting right, I didn't do that. Well, even a country is not very easy either. You see, the international situation is not good, and this country costs a lot of money.
 A lot of money, if you otherwise, if the international situation is good, we can improve a little, but no way, the world is like this.
 Ask: ah, big ye, you are what work do? I think you understand the government that way,
Answer: I farm by land, farm by oneself.
 Q: Yeah, what did you don't do when you came here in' 97?

 Answer: After doing a small business, take grandchildren.
 Q: Then you just said that you just said that they are still good. But do you feel that from your own point of view, do you as an old person, do you think they can do a little better? To make you even happier? Is there any advice from here?

 A: That's not so demanding. Ok, Ok, thank you for working hard

Q: How old are you aged now?
 A: 89 years old.
 Q: Are you a local person?
 A: My hometown is in Chaoshan. I visited Shenzhen 9 years ago, and now I live in Dongguan. He had three sons and a daughter, and the sons did business, so they moved over with him.
 Q: You look so healthy.
 A: Yes, I always get sick,
Q: But you look very energetic. What are the main diseases?
 A: This heart disease. Heart at first the body is very good, 72 years old that year car accident, originally I was often often have exercise, this his foot broken, just stay at home for a year. He started having a bad heart at the end of age 73. Well, the heart is not good. I keep taking that heart medicine. Well. Just eat about four or five years, four or five years later began that stomach is bad, gastric ulcer, slowly chronic gastritis erosion. After this heart also this to this foot influence, began the heart is not good. The heart is not good to take medicine for a long time, the stomach is bad.be not allowed. Now you can't even take that cerebrovascular, nor is the arteriosclerosis.
 Ask: oh, this way, that this is really multiple serious diseases, that you these diseases will usually affect your life a lot, is not you usually affect your own life, or mood ah, family relations, old body health can body body is not healthy trouble. Well, then you think you now the whole of your own life, because these diseases will be your life is generally satisfied?
 A: Generally speaking, it is still ok, right?
 Q: that you and the children their family relationship, and neighbor relationship these all very good?
 A: Generally,
Q: Not so much, right? How you have your children. How many children are there?
 A: Three boys and a girl.
 Q: Oh, four children. Are you a local person or a nonlocal person?
 Answer: I chaoshan come over, how long did you come over? I started working out in Shenzhen when I was 50.
 Q: I came here in 1997. I have been here for a long time. There are still more than ten days, more than 20 years away.
 Q: Well. Oh, if you come here, will you come here for your own business, or.
 A: My son did a little business.
 Q: So you came here with your son, so you came along with you?
 A: Well.
 Q: Oh, so, that's like most of the family population is here,
A: Yes, son, what daughter-in-law, grandchildren, and these are all here.
 Q: What do you think is the happiest thing in your life now is what you are most satisfied with. What are you most satisfied with in your whole state of life?
 Answer: satisfaction is this has children to have a family, have grandson. That grandson even graduated from college and came out to work.

 Q: Generally speaking, I am quite satisfied with my own feelings and my life. Still satisfied? Or not how satisfied?
 A: Satisfied.
 Q: Do you think you are living with your children or not right now?
 A: Live together.
 Q: That means that you think you live now and you say you live with your children, right?
 A: Yes.
 Q: If you live together, do you think it is quite different to live together with the young people? Or do you prefer to live alone or prefer to live with your children to answer: ah. No, no, then you are all the young people are very filial piety.

 Q: Oh, that's good, because many young people are not used to living with the old people. Generally speaking, you feel very comfortable living with them, don't they?
 Answer: That is still very filial piety.
 Q: Are you usually sick, they will accompany you to see a doctor. Will come with you, won't you? For example, what you usually take medicine, they will help you to pay attention to ah or how
A: Yes, yes.
 Q: Ok, that means you think you mean you are generally satisfied, but do you really think about it? Well, from the community, or the government, or from the community or other aspects, you can make your life a little better and happier. Do you have any good advice? What can we do? From the community, ah, or from this medical staff, ah, or from the government, ah, what do you think we can do better. It can improve your satisfaction, improve your satisfaction, improve your life happiness, then you are happier.

 Answer: that generally basically in that community now, that service also generally still can ah.
 Q: Generally speaking, do you think this kind of service in their community is ok in all aspects. Does the government have any care or special care for your elderly people?
 A: Yes, yes, yes, yes,
Q: What are the benefits.

 A: For example, it was in the countryside, ah ah, I live at the age of MAO Zedong era, it was very hard at that time. Yes, yes, that substance is very good, and you will get bored. Yes, that's different now. Since I have been in the countryside since I was 65 years ago, then the government has taken care of the old man. At first, it is 50 yuan, 50 yuan a month after 55 yuan, slowly lift, slowly lift, until now there are more than 200 points.
 Q: Every month, right?
 A: Yes
Q: every month you although you this number in the outside this situation is that proportion is very small, right? But you have so much money so much old people calculate that amount is very powerful. Then you quite understand the government.
 A: Well, yes.
 Q: Do they give you, for example, the usual care is if the community pays you if the government gives you some other benefits. Ask you how well you are. That means to care about you or to check for you. You're not having anything else.

 Answer: it is a family like me, if I also had to apply at that time, is my daughter my son-in-law she is also a civil servant.

 She works in the internal department. Well, if I were ordinary and I wouldn't want that much, I would apply to the disabled government, but I was just getting right, I didn't do that. Well, even a country is not very easy either. You see, the international situation is not good, and this country costs a lot of money.
 A lot of money, if you otherwise, if the international situation is good, we can improve a little, but no way, the world is like this.
 Ask: ah, big ye, you are what work do? I think you understand the government that way,
Answer: I farm by land, farm by oneself.
 Q: Yeah, what did you don't do when you came here in' 97?

 Answer: After doing a small business, take grandchildren.
 Q: Then you just said that you just said that they are still good. But do you feel that from your own point of view, do you as an old person, do you think they can do a little better? To make you even happier? Is there any advice from here?

 A: That's not so demanding. Ok, Ok, thank you for working hard

Q: How old are you aged now?
 A: 90 years old.
 Q: Are you a local person?
 A: My hometown is in Chaoshan. I came to Shenzhen 10 years ago, and now I live in Dongguan. He had three sons and a daughter, and the sons did business, so they moved over with him.
 Q: You look so healthy.
 A: Yes, I always get sick,
Q: But you look very energetic. What are the main diseases?
 A: This heart disease. Heart at first the body is still very good, 73 years old that year car accident, originally I was often often have exercise, this his foot was broken, just stay at home for a year. He started having a bad heart at the end of age 74. Well, the heart is not good. I keep taking that heart medicine. Well. Just eat about four or five years, four or five years later began that stomach is bad, gastric ulcer, slowly chronic gastritis erosion. After this heart also this to this foot influence, began the heart is not good. The heart is not good to take medicine for a long time, the stomach is bad.be not allowed. Now you can't even take that cerebrovascular, nor is the arteriosclerosis.
 Ask: oh, this way, that this is really multiple serious diseases, that you these diseases will usually affect your life a lot, is not you usually affect your own life, or mood ah, family relations, old body health can body body is not healthy trouble. Well, then you think you now the whole of your own life, because these diseases will be your life is generally satisfied?
 A: Generally speaking, it is still ok, right?
 Q: that you and the children their family relationship, and neighbor relationship these all very good?
 A: Generally,
Q: Not so much, right? How you have your children. How many children are there?
 A: Three boys and a girl.
 Q: Oh, four children. Are you a local person or a nonlocal person?
 Answer: I chaoshan come over, how long did you come over? I started working out in Shenzhen when I was 50.
 Q: I came here in 1997. I have been here for a long time. There are still more than ten days, more than 20 years away.
 Q: Well. Oh, if you come here, will you come here for your own business, or.
 A: My son did a little business.
 Q: So you came here with your son, so you came along with you?
 A: Well.
 Q: Oh, so, that's like most of the family population is here,
A: Yes, son, what daughter-in-law, grandchildren, and these are all here.
 Q: What do you think is the happiest thing in your life now is what you are most satisfied with. What are you most satisfied with in your whole state of life?
 Answer: satisfaction is this has children to have a family, have grandson. That grandson even graduated from college and came out to work.

 Q: Generally speaking, I am quite satisfied with my own feelings and my life. Still satisfied? Or not how satisfied?
 A: Satisfied.
 Q: Do you think you are living with your children or not right now?
 A: Live together.
 Q: That means that you think you live now and you say you live with your children, right?
 A: Yes.
 Q: If you live together, do you think it is quite different to live together with the young people? Or do you prefer to live alone or prefer to live with your children to answer: ah. No, no, then you are all the young people are very filial piety.

 Q: Oh, that's good, because many young people are not used to living with the old people. Generally speaking, you feel very comfortable living with them, don't they?
 Answer: That is still very filial piety.
 Q: Are you usually sick, they will accompany you to see a doctor. Will come with you, won't you? For example, what you usually take medicine, they will help you to pay attention to ah or how
A: Yes, yes.
 Q: Ok, that means you think you mean you are generally satisfied, but do you really think about it? Well, from the community, or the government, or from the community or other aspects, you can make your life a little better and happier. Do you have any good advice? What can we do? From the community, ah, or from this medical staff, ah, or from the government, ah, what do you think we can do better. It can improve your satisfaction, improve your satisfaction, improve your life happiness, then you are happier.

 Answer: that generally basically in that community now, that service also generally still can ah.
 Q: Generally speaking, do you think this kind of service in their community is ok in all aspects. Does the government have any care or special care for your elderly people?
 A: Yes, yes, yes, yes,
Q: What are the benefits.

 A: For example, it was in the countryside, ah ah, I live at the age of MAO Zedong era, it was very hard at that time. Yes, yes, that substance is very good, and you will get bored. Yes, that's different now. Since I have been in the countryside since I was 65 years ago, then the government has taken care of the old man. At first, it is 50 yuan, 50 yuan a month after 55 yuan, slowly lift, slowly lift, until now there are more than 200 points.
 Q: Every month, right?
 A: Yes
Q: every month you although you this number in the outside this situation is that proportion is very small, right? But you have so much money so much old people calculate that amount is very powerful. Then you quite understand the government.
 A: Well, yes.
 Q: Do they give you, for example, the usual care is if the community pays you if the government gives you some other benefits. Ask you how well you are. That means to care about you or to check for you. You're not having anything else.

 Answer: it is a family like me, if I also had to apply at that time, is my daughter my son-in-law she is also a civil servant.

 She works in the internal department. Well, if I were ordinary and I wouldn't want that much, I would apply to the disabled government, but I was just getting right, I didn't do that. Well, even a country is not very easy either. You see, the international situation is not good, and this country costs a lot of money.
 A lot of money, if you otherwise, if the international situation is good, we can improve a little, but no way, the world is like this.
 Ask: ah, big ye, you are what work do? I think you understand the government that way,
Answer: I farm by land, farm by oneself.
 Q: Yeah, what did you don't do when you came here in' 97?

 Answer: After doing a small business, take grandchildren.
 Q: Then you just said that you just said that they are still good. But do you feel that from your own point of view, do you as an old person, do you think they can do a little better? To make you even happier? Is there any advice from here?

 A: That's not so demanding. Ok, Ok, thank you for working hard

Q: How old are you aged now?
 A: 81 years old.
 Q: Are you a local person?
 Answer: My hometown is in Chaoshan. I visited Shenzhen 6 years ago, and now I live in Dongguan. He had three sons and a daughter, and the sons did business, so they moved over with him.
 Q: You look so healthy.
 A: Yes, I always get sick,
Q: But you look very energetic. What are the main diseases?
 A: This heart disease. Heart at first the body is still very good, 69 years old that year car accident ah, originally I was often often have exercise ah, this his foot was broken, just stay at home for a year. I started having a bad heart at the end of my 70s. Well, the heart is not good. I keep taking that heart medicine. Well. Just eat about four or five years, four or five years later began that stomach is bad, gastric ulcer, slowly chronic gastritis erosion. After this heart also this to this foot influence, began the heart is not good. The heart is not good to take medicine for a long time, the stomach is bad.be not allowed. Now you can't even take that cerebrovascular, nor is the arteriosclerosis.
 Ask: oh, this way, that this is really multiple serious diseases, that you these diseases will usually affect your life a lot, is not you usually affect your own life, or mood ah, family relations, old body health can body body is not healthy trouble. Well, then you think you now the whole of your own life, because these diseases will be your life is generally satisfied?
 A: Generally speaking, it is still ok, right?
 Q: that you and the children their family relationship, and neighbor relationship these all very good?
 A: Generally,
Q: Not so much, right? How you have your children. How many children are there?
 A: Three boys and a girl.
 Q: Oh, four children. Are you a local person or a nonlocal person?
 Answer: I chaoshan come over, how long did you come over? I started working out in Shenzhen when I was 50.
 Q: I came here in 1997. I have been here for a long time. There are still more than ten days, more than 20 years away.
 Q: Well. Oh, if you come here, will you come here for your own business, or.
 A: My son did a little business.
 Q: So you came here with your son, so you came along with you?
 A: Well.
 Q: Oh, so, that's like most of the family population is here,
A: Yes, son, what daughter-in-law, grandchildren, and these are all here.
 Q: What do you think is the happiest thing in your life now is what you are most satisfied with. What are you most satisfied with in your whole state of life?
 Answer: satisfaction is this has children to have a family, have grandson. That grandson even graduated from college and came out to work.

 Q: Generally speaking, I am quite satisfied with my own feelings and my life. Still satisfied? Or not how satisfied?
 A: Satisfied.
 Q: Do you think you are living with your children or not right now?
 A: Live together.
 Q: That means that you think you live now and you say you live with your children, right?
 A: Yes.
 Q: If you live together, do you think it is quite different to live together with the young people? Or do you prefer to live alone or prefer to live with your children to answer: ah. No, no, then you are all the young people are very filial piety.

 Q: Oh, that's good, because many young people are not used to living with the old people. Generally speaking, you feel very comfortable living with them, don't they?
 Answer: That is still very filial piety.
 Q: Are you usually sick, they will accompany you to see a doctor. Will come with you, won't you? For example, what you usually take medicine, they will help you to pay attention to ah or how
A: Yes, yes.
 Q: Ok, that means you think you mean you are generally satisfied, but do you really think about it? Well, from the community, or the government, or from the community or other aspects, you can make your life a little better and happier. Do you have any good advice? What can we do? From the community, ah, or from this medical staff, ah, or from the government, ah, what do you think we can do better. It can improve your satisfaction, improve your satisfaction, improve your life happiness, then you are happier.

 Answer: that generally basically in that community now, that service also generally still can ah.
 Q: Generally speaking, do you think this kind of service in their community is ok in all aspects. Does the government have any care or special care for your elderly people?
 A: Yes, yes, yes, yes,
Q: What are the benefits.

 A: For example, it was in the countryside, ah ah, I live at the age of MAO Zedong era, it was very hard at that time. Yes, yes, that substance is very good, and you will get bored. Yes, that's different now. Since I have been in the countryside since I was 65 years ago, then the government has taken care of the old man. At first, it is 50 yuan, 50 yuan a month after 55 yuan, slowly lift, slowly lift, until now there are more than 200 points.
 Q: Every month, right?
 A: Yes
Q: every month you although you this number in the outside this situation is that proportion is very small, right? But you have so much money so much old people calculate that amount is very powerful. Then you quite understand the government.
 A: Well, yes.
 Q: Do they give you, for example, the usual care is if the community pays you if the government gives you some other benefits. Ask you how well you are. That means to care about you or to check for you. You're not having anything else.

 Answer: it is a family like me, if I also had to apply at that time, is my daughter my son-in-law she is also a civil servant.

 She works in the internal department. Well, if I were ordinary and I wouldn't want that much, I would apply to the disabled government, but I was just getting right, I didn't do that. Well, even a country is not very easy either. You see, the international situation is not good, and this country costs a lot of money.
 A lot of money, if you otherwise, if the international situation is good, we can improve a little, but no way, the world is like this.
 Ask: ah, big ye, you are what work do? I think you understand the government that way,
Answer: I farm by land, farm by oneself.
 Q: Yeah, what did you don't do when you came here in' 97?

 Answer: After doing a small business, take grandchildren.
 Q: Then you just said that you just said that they are still good. But do you feel that from your own point of view, do you as an old person, do you think they can do a little better? To make you even happier? Is there any advice from here?

 A: That's not so demanding. Ok, Ok, thank you for working hard

Q: How old are you aged now?
 A: 82 years old.
 Q: Are you a local person?
 Answer: My hometown is in Chaoshan. I visited Shenzhen 7 years ago, and now I live in Dongguan. He had three sons and a daughter, and the sons did business, so they moved over with him.
 Q: You look so healthy.
 A: Yes, I always get sick,
Q: But you look very energetic. What are the main diseases?
 A: This heart disease. Heart at first the body is still very good, 70 years old that year car accident, originally I was often often have exercise, this his foot broke, just stay at home for a year. I started having a bad heart at the end of age 71. Well, the heart is not good. I keep taking that heart medicine. Well. Just eat about four or five years, four or five years later began that stomach is bad, gastric ulcer, slowly chronic gastritis erosion. After this heart also this to this foot influence, began the heart is not good. The heart is not good to take medicine for a long time, the stomach is bad.be not allowed. Now you can't even take that cerebrovascular, nor is the arteriosclerosis.
 Ask: oh, this way, that this is really multiple serious diseases, that you these diseases will usually affect your life a lot, is not you usually affect your own life, or mood ah, family relations, old body health can body body is not healthy trouble. Well, then you think you now the whole of your own life, because these diseases will be your life is generally satisfied?
 A: Generally speaking, it is still ok, right?
 Q: that you and the children their family relationship, and neighbor relationship these all very good?
 A: Generally,
Q: Not so much, right? How you have your children. How many children are there?
 A: Three boys and a girl.
 Q: Oh, four children. Are you a local person or a nonlocal person?
 Answer: I chaoshan come over, how long did you come over? I started working out in Shenzhen when I was 50.
 Q: I came here in 1997. I have been here for a long time. There are still more than ten days, more than 20 years away.
 Q: Well. Oh, if you come here, will you come here for your own business, or.
 A: My son did a little business.
 Q: So you came here with your son, so you came along with you?
 A: Well.
 Q: Oh, so, that's like most of the family population is here,
A: Yes, son, what daughter-in-law, grandchildren, and these are all here.
 Q: What do you think is the happiest thing in your life now is what you are most satisfied with. What are you most satisfied with in your whole state of life?
 Answer: satisfaction is this has children to have a family, have grandson. That grandson even graduated from college and came out to work.

 Q: Generally speaking, I am quite satisfied with my own feelings and my life. Still satisfied? Or not how satisfied?
 A: Satisfied.
 Q: Do you think you are living with your children or not right now?
 A: Live together.
 Q: That means that you think you live now and you say you live with your children, right?
 A: Yes.
 Q: If you live together, do you think it is quite different to live together with the young people? Or do you prefer to live alone or prefer to live with your children to answer: ah. No, no, then you are all the young people are very filial piety.

 Q: Oh, that's good, because many young people are not used to living with the old people. Generally speaking, you feel very comfortable living with them, don't they?
 Answer: That is still very filial piety.
 Q: Are you usually sick, they will accompany you to see a doctor. Will come with you, won't you? For example, what you usually take medicine, they will help you to pay attention to ah or how
A: Yes, yes.
 Q: Ok, that means you think you mean you are generally satisfied, but do you really think about it? Well, from the community, or the government, or from the community or other aspects, you can make your life a little better and happier. Do you have any good advice? What can we do? From the community, ah, or from this medical staff, ah, or from the government, ah, what do you think we can do better. It can improve your satisfaction, improve your satisfaction, improve your life happiness, then you are happier.

 Answer: that generally basically in that community now, that service also generally still can ah.
 Q: Generally speaking, do you think this kind of service in their community is ok in all aspects. Does the government have any care or special care for your elderly people?
 A: Yes, yes, yes, yes,
Q: What are the benefits.

 A: For example, it was in the countryside, ah ah, I live at the age of MAO Zedong era, it was very hard at that time. Yes, yes, that substance is very good, and you will get bored. Yes, that's different now. Since I have been in the countryside since I was 65 years ago, then the government has taken care of the old man. At first, it is 50 yuan, 50 yuan a month after 55 yuan, slowly lift, slowly lift, until now there are more than 200 points.
 Q: Every month, right?
 A: Yes
Q: every month you although you this number in the outside this situation is that proportion is very small, right? But you have so much money so much old people calculate that amount is very powerful. Then you quite understand the government.
 A: Well, yes.
 Q: Do they give you, for example, the usual care is if the community pays you if the government gives you some other benefits. Ask you how well you are. That means to care about you or to check for you. You're not having anything else.

 Answer: it is a family like me, if I also had to apply at that time, is my daughter my son-in-law she is also a civil servant.

 She works in the internal department. Well, if I were ordinary and I wouldn't want that much, I would apply to the disabled government, but I was just getting right, I didn't do that. Well, even a country is not very easy either. You see, the international situation is not good, and this country costs a lot of money.
 A lot of money, if you otherwise, if the international situation is good, we can improve a little, but no way, the world is like this.
 Ask: ah, big ye, you are what work do? I think you understand the government that way,
Answer: I farm by land, farm by oneself.
 Q: Yeah, what did you don't do when you came here in' 97?

 Answer: After doing a small business, take grandchildren.
 Q: Then you just said that you just said that they are still good. But do you feel that from your own point of view, do you as an old person, do you think they can do a little better? To make you even happier? Is there any advice from here?

 A: That's not so demanding. Ok, Ok, thank you for working hard

Q: How old are you aged now?
 A: 83 years old.
 Q: Are you a local person?
 A: My hometown is in Chaoshan. I visited Shenzhen 8 years ago, and now I live in Dongguan. He had three sons and a daughter, and the sons did business, so they moved over with him.
 Q: You look so healthy.
 A: Yes, I always get sick,
Q: But you look very energetic. What are the main diseases?
 A: This heart disease. Heart at first the body is still very good, 71 years old that year car accident ah, originally I was often often have exercise ah, this his foot broke, just stay at home for a year. He started having a bad heart at the end of age 72. Well, the heart is not good. I keep taking that heart medicine. Well. Just eat about four or five years, four or five years later began that stomach is bad, gastric ulcer, slowly chronic gastritis erosion. After this heart also this to this foot influence, began the heart is not good. The heart is not good to take medicine for a long time, the stomach is bad.be not allowed. Now you can't even take that cerebrovascular, nor is the arteriosclerosis.
 Ask: oh, this way, that this is really multiple serious diseases, that you these diseases will usually affect your life a lot, is not you usually affect your own life, or mood ah, family relations, old body health can body body is not healthy trouble. Well, then you think you now the whole of your own life, because these diseases will be your life is generally satisfied?
 A: Generally speaking, it is still ok, right?
 Q: that you and the children their family relationship, and neighbor relationship these all very good?
 A: Generally,
Q: Not so much, right? How you have your children. How many children are there?
 A: Three boys and a girl.
 Q: Oh, four children. Are you a local person or a nonlocal person?
 Answer: I chaoshan come over, how long did you come over? I started working out in Shenzhen when I was 50.
 Q: I came here in 1997. I have been here for a long time. There are still more than ten days, more than 20 years away.
 Q: Well. Oh, if you come here, will you come here for your own business, or.
 A: My son did a little business.
 Q: So you came here with your son, so you came along with you?
 A: Well.
 Q: Oh, so, that's like most of the family population is here,
A: Yes, son, what daughter-in-law, grandchildren, and these are all here.
 Q: What do you think is the happiest thing in your life now is what you are most satisfied with. What are you most satisfied with in your whole state of life?
 Answer: satisfaction is this has children to have a family, have grandson. That grandson even graduated from college and came out to work.

 Q: Generally speaking, I am quite satisfied with my own feelings and my life. Still satisfied? Or not how satisfied?
 A: Satisfied.
 Q: Do you think you are living with your children or not right now?
 A: Live together.
 Q: That means that you think you live now and you say you live with your children, right?
 A: Yes.
 Q: If you live together, do you think it is quite different to live together with the young people? Or do you prefer to live alone or prefer to live with your children to answer: ah. No, no, then you are all the young people are very filial piety.

 Q: Oh, that's good, because many young people are not used to living with the old people. Generally speaking, you feel very comfortable living with them, don't they?
 Answer: That is still very filial piety.
 Q: Are you usually sick, they will accompany you to see a doctor. Will come with you, won't you? For example, what you usually take medicine, they will help you to pay attention to ah or how
A: Yes, yes.
 Q: Ok, that means you think you mean you are generally satisfied, but do you really think about it? Well, from the community, or the government, or from the community or other aspects, you can make your life a little better and happier. Do you have any good advice? What can we do? From the community, ah, or from this medical staff, ah, or from the government, ah, what do you think we can do better. It can improve your satisfaction, improve your satisfaction, improve your life happiness, then you are happier.

 Answer: that generally basically in that community now, that service also generally still can ah.
 Q: Generally speaking, do you think this kind of service in their community is ok in all aspects. Does the government have any care or special care for your elderly people?
 A: Yes, yes, yes, yes,
Q: What are the benefits.

 A: For example, it was in the countryside, ah ah, I live at the age of MAO Zedong era, it was very hard at that time. Yes, yes, that substance is very good, and you will get bored. Yes, that's different now. Since I have been in the countryside since I was 65 years ago, then the government has taken care of the old man. At first, it is 50 yuan, 50 yuan a month after 55 yuan, slowly lift, slowly lift, until now there are more than 200 points.
 Q: Every month, right?
 A: Yes
Q: every month you although you this number in the outside this situation is that proportion is very small, right? But you have so much money so much old people calculate that amount is very powerful. Then you quite understand the government.
 A: Well, yes.
 Q: Do they give you, for example, the usual care is if the community pays you if the government gives you some other benefits. Ask you how well you are. That means to care about you or to check for you. You're not having anything else.

 Answer: it is a family like me, if I also had to apply at that time, is my daughter my son-in-law she is also a civil servant.

 She works in the internal department. Well, if I were ordinary and I wouldn't want that much, I would apply to the disabled government, but I was just getting right, I didn't do that. Well, even a country is not very easy either. You see, the international situation is not good, and this country costs a lot of money.
 A lot of money, if you otherwise, if the international situation is good, we can improve a little, but no way, the world is like this.
 Ask: ah, big ye, you are what work do? I think you understand the government that way,
Answer: I farm by land, farm by oneself.
 Q: Yeah, what did you don't do when you came here in' 97?

 Answer: After doing a small business, take grandchildren.
 Q: Then you just said that you just said that they are still good. But do you feel that from your own point of view, do you as an old person, do you think they can do a little better? To make you even happier? Is there any advice from here?

 A: That's not so demanding. Ok, Ok, thank you for working hard

Q: How old are you aged now?
 A: 84 years old.
 Q: Are you a local person?
 A: My hometown is in Chaoshan. I visited Shenzhen 9 years ago, and now I live in Dongguan. He had three sons and a daughter, and the sons did business, so they moved over with him.
 Q: You look so healthy.
 A: Yes, I always get sick,
Q: But you look very energetic. What are the main diseases?
 A: This heart disease. Heart at first the body is very good, 72 years old that year car accident, originally I was often often have exercise, this his foot broken, just stay at home for a year. He started having a bad heart at the end of age 73. Well, the heart is not good. I keep taking that heart medicine. Well. Just eat about four or five years, four or five years later began that stomach is bad, gastric ulcer, slowly chronic gastritis erosion. After this heart also this to this foot influence, began the heart is not good. The heart is not good to take medicine for a long time, the stomach is bad.be not allowed. Now you can't even take that cerebrovascular, nor is the arteriosclerosis.
 Ask: oh, this way, that this is really multiple serious diseases, that you these diseases will usually affect your life a lot, is not you usually affect your own life, or mood ah, family relations, old body health can body body is not healthy trouble. Well, then you think you now the whole of your own life, because these diseases will be your life is generally satisfied?
 A: Generally speaking, it is still ok, right?
 Q: that you and the children their family relationship, and neighbor relationship these all very good?
 A: Generally,
Q: Not so much, right? How you have your children. How many children are there?
 A: Three boys and a girl.
 Q: Oh, four children. Are you a local person or a nonlocal person?
 Answer: I chaoshan come over, how long did you come over? I started working out in Shenzhen when I was 50.
 Q: I came here in 1997. I have been here for a long time. There are still more than ten days, more than 20 years away.
 Q: Well. Oh, if you come here, will you come here for your own business, or.
 A: My son did a little business.
 Q: So you came here with your son, so you came along with you?
 A: Well.
 Q: Oh, so, that's like most of the family population is here,
A: Yes, son, what daughter-in-law, grandchildren, and these are all here.
 Q: What do you think is the happiest thing in your life now is what you are most satisfied with. What are you most satisfied with in your whole state of life?
 Answer: satisfaction is this has children to have a family, have grandson. That grandson even graduated from college and came out to work.

 Q: Generally speaking, I am quite satisfied with my own feelings and my life. Still satisfied? Or not how satisfied?
 A: Satisfied.
 Q: Do you think you are living with your children or not right now?
 A: Live together.
 Q: That means that you think you live now and you say you live with your children, right?
 A: Yes.
 Q: If you live together, do you think it is quite different to live together with the young people? Or do you prefer to live alone or prefer to live with your children to answer: ah. No, no, then you are all the young people are very filial piety.

 Q: Oh, that's good, because many young people are not used to living with the old people. Generally speaking, you feel very comfortable living with them, don't they?
 Answer: That is still very filial piety.
 Q: Are you usually sick, they will accompany you to see a doctor. Will come with you, won't you? For example, what you usually take medicine, they will help you to pay attention to ah or how
A: Yes, yes.
 Q: Ok, that means you think you mean you are generally satisfied, but do you really think about it? Well, from the community, or the government, or from the community or other aspects, you can make your life a little better and happier. Do you have any good advice? What can we do? From the community, ah, or from this medical staff, ah, or from the government, ah, what do you think we can do better. It can improve your satisfaction, improve your satisfaction, improve your life happiness, then you are happier.

 Answer: that generally basically in that community now, that service also generally still can ah.
 Q: Generally speaking, do you think this kind of service in their community is ok in all aspects. Does the government have any care or special care for your elderly people?
 A: Yes, yes, yes, yes,
Q: What are the benefits.

 A: For example, it was in the countryside, ah ah, I live at the age of MAO Zedong era, it was very hard at that time. Yes, yes, that substance is very good, and you will get bored. Yes, that's different now. Since I have been in the countryside since I was 65 years ago, then the government has taken care of the old man. At first, it is 50 yuan, 50 yuan a month after 55 yuan, slowly lift, slowly lift, until now there are more than 200 points.
 Q: Every month, right?
 A: Yes
Q: every month you although you this number in the outside this situation is that proportion is very small, right? But you have so much money so much old people calculate that amount is very powerful. Then you quite understand the government.
 A: Well, yes.
 Q: Do they give you, for example, the usual care is if the community pays you if the government gives you some other benefits. Ask you how well you are. That means to care about you or to check for you. You're not having anything else.

 Answer: it is a family like me, if I also had to apply at that time, is my daughter my son-in-law she is also a civil servant.

 She works in the internal department. Well, if I were ordinary and I wouldn't want that much, I would apply to the disabled government, but I was just getting right, I didn't do that. Well, even a country is not very easy either. You see, the international situation is not good, and this country costs a lot of money.
 A lot of money, if you otherwise, if the international situation is good, we can improve a little, but no way, the world is like this.
 Ask: ah, big ye, you are what work do? I think you understand the government that way,
Answer: I farm by land, farm by oneself.
 Q: Yeah, what did you don't do when you came here in' 97?

 Answer: After doing a small business, take grandchildren.
 Q: Then you just said that you just said that they are still good. But do you feel that from your own point of view, do you as an old person, do you think they can do a little better? To make you even happier? Is there any advice from here?

 A: That's not so demanding. Ok, Ok, thank you for working hard

Q: How old are you aged now?
 A: 85 years old.
 Q: Are you a local person?
 A: My hometown is in Chaoshan. I came to Shenzhen 10 years ago, and now I live in Dongguan. He had three sons and a daughter, and the sons did business, so they moved over with him.
 Q: You look so healthy.
 A: Yes, I always get sick,
Q: But you look very energetic. What are the main diseases?
 A: This heart disease. Heart at first the body is still very good, 73 years old that year car accident, originally I was often often have exercise, this his foot was broken, just stay at home for a year. He started having a bad heart at the end of age 74. Well, the heart is not good. I keep taking that heart medicine. Well. Just eat about four or five years, four or five years later began that stomach is bad, gastric ulcer, slowly chronic gastritis erosion. After this heart also this to this foot influence, began the heart is not good. The heart is not good to take medicine for a long time, the stomach is bad.be not allowed. Now you can't even take that cerebrovascular, nor is the arteriosclerosis.
 Ask: oh, this way, that this is really multiple serious diseases, that you these diseases will usually affect your life a lot, is not you usually affect your own life, or mood ah, family relations, old body health can body body is not healthy trouble. Well, then you think you now the whole of your own life, because these diseases will be your life is generally satisfied?
 A: Generally speaking, it is still ok, right?
 Q: that you and the children their family relationship, and neighbor relationship these all very good?
 A: Generally,
Q: Not so much, right? How you have your children. How many children are there?
 A: Three boys and a girl.
 Q: Oh, four children. Are you a local person or a nonlocal person?
 Answer: I chaoshan come over, how long did you come over? I started working out in Shenzhen when I was 50.
 Q: I came here in 1997. I have been here for a long time. There are still more than ten days, more than 20 years away.
 Q: Well. Oh, if you come here, will you come here for your own business, or.
 A: My son did a little business.
 Q: So you came here with your son, so you came along with you?
 A: Well.
 Q: Oh, so, that's like most of the family population is here,
A: Yes, son, what daughter-in-law, grandchildren, and these are all here.
 Q: What do you think is the happiest thing in your life now is what you are most satisfied with. What are you most satisfied with in your whole state of life?
 Answer: satisfaction is this has children to have a family, have grandson. That grandson even graduated from college and came out to work.

 Q: Generally speaking, I am quite satisfied with my own feelings and my life. Still satisfied? Or not how satisfied?
 A: Satisfied.
 Q: Do you think you are living with your children or not right now?
 A: Live together.
 Q: That means that you think you live now and you say you live with your children, right?
 A: Yes.
 Q: If you live together, do you think it is quite different to live together with the young people? Or do you prefer to live alone or prefer to live with your children to answer: ah. No, no, then you are all the young people are very filial piety.

 Q: Oh, that's good, because many young people are not used to living with the old people. Generally speaking, you feel very comfortable living with them, don't they?
 Answer: That is still very filial piety.
 Q: Are you usually sick, they will accompany you to see a doctor. Will come with you, won't you? For example, what you usually take medicine, they will help you to pay attention to ah or how
A: Yes, yes.
 Q: Ok, that means you think you mean you are generally satisfied, but do you really think about it? Well, from the community, or the government, or from the community or other aspects, you can make your life a little better and happier. Do you have any good advice? What can we do? From the community, ah, or from this medical staff, ah, or from the government, ah, what do you think we can do better. It can improve your satisfaction, improve your satisfaction, improve your life happiness, then you are happier.

 Answer: that generally basically in that community now, that service also generally still can ah.
 Q: Generally speaking, do you think this kind of service in their community is ok in all aspects. Does the government have any care or special care for your elderly people?
 A: Yes, yes, yes, yes,
Q: What are the benefits.

 A: For example, it was in the countryside, ah ah, I live at the age of MAO Zedong era, it was very hard at that time. Yes, yes, that substance is very good, and you will get bored. Yes, that's different now. Since I have been in the countryside since I was 65 years ago, then the government has taken care of the old man. At first, it is 50 yuan, 50 yuan a month after 55 yuan, slowly lift, slowly lift, until now there are more than 200 points.
 Q: Every month, right?
 A: Yes
Q: every month you although you this number in the outside this situation is that proportion is very small, right? But you have so much money so much old people calculate that amount is very powerful. Then you quite understand the government.
 A: Well, yes.
 Q: Do they give you, for example, the usual care is if the community pays you if the government gives you some other benefits. Ask you how well you are. That means to care about you or to check for you. You're not having anything else.

 Answer: it is a family like me, if I also had to apply at that time, is my daughter my son-in-law she is also a civil servant.

 She works in the internal department. Well, if I were ordinary and I wouldn't want that much, I would apply to the disabled government, but I was just getting right, I didn't do that. Well, even a country is not very easy either. You see, the international situation is not good, and this country costs a lot of money.
 A lot of money, if you otherwise, if the international situation is good, we can improve a little, but no way, the world is like this.
 Ask: ah, big ye, you are what work do? I think you understand the government that way,
Answer: I farm by land, farm by oneself.
 Q: Yeah, what did you don't do when you came here in' 97?

 Answer: After doing a small business, take grandchildren.
 Q: Then you just said that you just said that they are still good. But do you feel that from your own point of view, do you as an old person, do you think they can do a little better? To make you even happier? Is there any advice from here?

 A: That's not so demanding. Ok, Ok, thank you for working hard

Q: How old are you aged now?
 A: 86 years old.
 Q: Are you a local person?
 Answer: My hometown is in Chaoshan. I visited Shenzhen 6 years ago, and now I live in Dongguan. He had three sons and a daughter, and the sons did business, so they moved over with him.
 Q: You look so healthy.
 A: Yes, I always get sick,
Q: But you look very energetic. What are the main diseases?
 A: This heart disease. Heart at first the body is still very good, 69 years old that year car accident ah, originally I was often often have exercise ah, this his foot was broken, just stay at home for a year. I started having a bad heart at the end of my 70s. Well, the heart is not good. I keep taking that heart medicine. Well. Just eat about four or five years, four or five years later began that stomach is bad, gastric ulcer, slowly chronic gastritis erosion. After this heart also this to this foot influence, began the heart is not good. The heart is not good to take medicine for a long time, the stomach is bad.be not allowed. Now you can't even take that cerebrovascular, nor is the arteriosclerosis.
 Ask: oh, this way, that this is really multiple serious diseases, that you these diseases will usually affect your life a lot, is not you usually affect your own life, or mood ah, family relations, old body health can body body is not healthy trouble. Well, then you think you now the whole of your own life, because these diseases will be your life is generally satisfied?
 A: Generally speaking, it is still ok, right?
 Q: that you and the children their family relationship, and neighbor relationship these all very good?
 A: Generally,
Q: Not so much, right? How you have your children. How many children are there?
 A: Three boys and a girl.
 Q: Oh, four children. Are you a local person or a nonlocal person?
 Answer: I chaoshan come over, how long did you come over? I started working out in Shenzhen when I was 50.
 Q: I came here in 1997. I have been here for a long time. There are still more than ten days, more than 20 years away.
 Q: Well. Oh, if you come here, will you come here for your own business, or.
 A: My son did a little business.
 Q: So you came here with your son, so you came along with you?
 A: Well.
 Q: Oh, so, that's like most of the family population is here,
A: Yes, son, what daughter-in-law, grandchildren, and these are all here.
 Q: What do you think is the happiest thing in your life now is what you are most satisfied with. What are you most satisfied with in your whole state of life?
 Answer: satisfaction is this has children to have a family, have grandson. That grandson even graduated from college and came out to work.

 Q: Generally speaking, I am quite satisfied with my own feelings and my life. Still satisfied? Or not how satisfied?
 A: Satisfied.
 Q: Do you think you are living with your children or not right now?
 A: Live together.
 Q: That means that you think you live now and you say you live with your children, right?
 A: Yes.
 Q: If you live together, do you think it is quite different to live together with the young people? Or do you prefer to live alone or prefer to live with your children to answer: ah. No, no, then you are all the young people are very filial piety.

 Q: Oh, that's good, because many young people are not used to living with the old people. Generally speaking, you feel very comfortable living with them, don't they?
 Answer: That is still very filial piety.
 Q: Are you usually sick, they will accompany you to see a doctor. Will come with you, won't you? For example, what you usually take medicine, they will help you to pay attention to ah or how
A: Yes, yes.
 Q: Ok, that means you think you mean you are generally satisfied, but do you really think about it? Well, from the community, or the government, or from the community or other aspects, you can make your life a little better and happier. Do you have any good advice? What can we do? From the community, ah, or from this medical staff, ah, or from the government, ah, what do you think we can do better. It can improve your satisfaction, improve your satisfaction, improve your life happiness, then you are happier.

 Answer: that generally basically in that community now, that service also generally still can ah.
 Q: Generally speaking, do you think this kind of service in their community is ok in all aspects. Does the government have any care or special care for your elderly people?
 A: Yes, yes, yes, yes,
Q: What are the benefits.

 A: For example, it was in the countryside, ah ah, I live at the age of MAO Zedong era, it was very hard at that time. Yes, yes, that substance is very good, and you will get bored. Yes, that's different now. Since I have been in the countryside since I was 65 years ago, then the government has taken care of the old man. At first, it is 50 yuan, 50 yuan a month after 55 yuan, slowly lift, slowly lift, until now there are more than 200 points.
 Q: Every month, right?
 A: Yes
Q: every month you although you this number in the outside this situation is that proportion is very small, right? But you have so much money so much old people calculate that amount is very powerful. Then you quite understand the government.
 A: Well, yes.
 Q: Do they give you, for example, the usual care is if the community pays you if the government gives you some other benefits. Ask you how well you are. That means to care about you or to check for you. You're not having anything else.

 Answer: it is a family like me, if I also had to apply at that time, is my daughter my son-in-law she is also a civil servant.

 She works in the internal department. Well, if I were ordinary and I wouldn't want that much, I would apply to the disabled government, but I was just getting right, I didn't do that. Well, even a country is not very easy either. You see, the international situation is not good, and this country costs a lot of money.
 A lot of money, if you otherwise, if the international situation is good, we can improve a little, but no way, the world is like this.
 Ask: ah, big ye, you are what work do? I think you understand the government that way,
Answer: I farm by land, farm by oneself.
 Q: Yeah, what did you don't do when you came here in' 97?

 Answer: After doing a small business, take grandchildren.
 Q: Then you just said that you just said that they are still good. But do you feel that from your own point of view, do you as an old person, do you think they can do a little better? To make you even happier? Is there any advice from here?

 A: That's not so demanding. Ok, Ok, thank you for working hard

Q: How old are you aged now?
 A: 87 years old.
 Q: Are you a local person?
 Answer: My hometown is in Chaoshan. I visited Shenzhen 7 years ago, and now I live in Dongguan. He had three sons and a daughter, and the sons did business, so they moved over with him.
 Q: You look so healthy.
 A: Yes, I always get sick,
Q: But you look very energetic. What are the main diseases?
 A: This heart disease. Heart at first the body is still very good, 70 years old that year car accident, originally I was often often have exercise, this his foot broke, just stay at home for a year. I started having a bad heart at the end of age 71. Well, the heart is not good. I keep taking that heart medicine. Well. Just eat about four or five years, four or five years later began that stomach is bad, gastric ulcer, slowly chronic gastritis erosion. After this heart also this to this foot influence, began the heart is not good. The heart is not good to take medicine for a long time, the stomach is bad.be not allowed. Now you can't even take that cerebrovascular, nor is the arteriosclerosis.
 Ask: oh, this way, that this is really multiple serious diseases, that you these diseases will usually affect your life a lot, is not you usually affect your own life, or mood ah, family relations, old body health can body body is not healthy trouble. Well, then you think you now the whole of your own life, because these diseases will be your life is generally satisfied?
 A: Generally speaking, it is still ok, right?
 Q: that you and the children their family relationship, and neighbor relationship these all very good?
 A: Generally,
Q: Not so much, right? How you have your children. How many children are there?
 A: Three boys and a girl.
 Q: Oh, four children. Are you a local person or a nonlocal person?
 Answer: I chaoshan come over, how long did you come over? I started working out in Shenzhen when I was 50.
 Q: I came here in 1997. I have been here for a long time. There are still more than ten days, more than 20 years away.
 Q: Well. Oh, if you come here, will you come here for your own business, or.
 A: My son did a little business.
 Q: So you came here with your son, so you came along with you?
 A: Well.
 Q: Oh, so, that's like most of the family population is here,
A: Yes, son, what daughter-in-law, grandchildren, and these are all here.
 Q: What do you think is the happiest thing in your life now is what you are most satisfied with. What are you most satisfied with in your whole state of life?
 Answer: satisfaction is this has children to have a family, have grandson. That grandson even graduated from college and came out to work.

 Q: Generally speaking, I am quite satisfied with my own feelings and my life. Still satisfied? Or not how satisfied?
 A: Satisfied.
 Q: Do you think you are living with your children or not right now?
 A: Live together.
 Q: That means that you think you live now and you say you live with your children, right?
 A: Yes.
 Q: If you live together, do you think it is quite different to live together with the young people? Or do you prefer to live alone or prefer to live with your children to answer: ah. No, no, then you are all the young people are very filial piety.

 Q: Oh, that's good, because many young people are not used to living with the old people. Generally speaking, you feel very comfortable living with them, don't they?
 Answer: That is still very filial piety.
 Q: Are you usually sick, they will accompany you to see a doctor. Will come with you, won't you? For example, what you usually take medicine, they will help you to pay attention to ah or how
A: Yes, yes.
 Q: Ok, that means you think you mean you are generally satisfied, but do you really think about it? Well, from the community, or the government, or from the community or other aspects, you can make your life a little better and happier. Do you have any good advice? What can we do? From the community, ah, or from this medical staff, ah, or from the government, ah, what do you think we can do better. It can improve your satisfaction, improve your satisfaction, improve your life happiness, then you are happier.

 Answer: that generally basically in that community now, that service also generally still can ah.
 Q: Generally speaking, do you think this kind of service in their community is ok in all aspects. Does the government have any care or special care for your elderly people?
 A: Yes, yes, yes, yes,
Q: What are the benefits.

 A: For example, it was in the countryside, ah ah, I live at the age of MAO Zedong era, it was very hard at that time. Yes, yes, that substance is very good, and you will get bored. Yes, that's different now. Since I have been in the countryside since I was 65 years ago, then the government has taken care of the old man. At first, it is 50 yuan, 50 yuan a month after 55 yuan, slowly lift, slowly lift, until now there are more than 200 points.
 Q: Every month, right?
 A: Yes
Q: every month you although you this number in the outside this situation is that proportion is very small, right? But you have so much money so much old people calculate that amount is very powerful. Then you quite understand the government.
 A: Well, yes.
 Q: Do they give you, for example, the usual care is if the community pays you if the government gives you some other benefits. Ask you how well you are. That means to care about you or to check for you. You're not having anything else.

 Answer: it is a family like me, if I also had to apply at that time, is my daughter my son-in-law she is also a civil servant.

 She works in the internal department. Well, if I were ordinary and I wouldn't want that much, I would apply to the disabled government, but I was just getting right, I didn't do that. Well, even a country is not very easy either. You see, the international situation is not good, and this country costs a lot of money.
 A lot of money, if you otherwise, if the international situation is good, we can improve a little, but no way, the world is like this.
 Ask: ah, big ye, you are what work do? I think you understand the government that way,
Answer: I farm by land, farm by oneself.
 Q: Yeah, what did you don't do when you came here in' 97?

 Answer: After doing a small business, take grandchildren.
 Q: Then you just said that you just said that they are still good. But do you feel that from your own point of view, do you as an old person, do you think they can do a little better? To make you even happier? Is there any advice from here?

 A: That's not so demanding. Ok, Ok, thank you for working hard

Q: How old are you aged now?
 A: 88 years old.
 Q: Are you a local person?
 A: My hometown is in Chaoshan. I visited Shenzhen 8 years ago, and now I live in Dongguan. He had three sons and a daughter, and the sons did business, so they moved over with him.
 Q: You look so healthy.
 A: Yes, I always get sick,
Q: But you look very energetic. What are the main diseases?
 A: This heart disease. Heart at first the body is still very good, 71 years old that year car accident ah, originally I was often often have exercise ah, this his foot broke, just stay at home for a year. He started having a bad heart at the end of age 72. Well, the heart is not good. I keep taking that heart medicine. Well. Just eat about four or five years, four or five years later began that stomach is bad, gastric ulcer, slowly chronic gastritis erosion. After this heart also this to this foot influence, began the heart is not good. The heart is not good to take medicine for a long time, the stomach is bad.be not allowed. Now you can't even take that cerebrovascular, nor is the arteriosclerosis.
 Ask: oh, this way, that this is really multiple serious diseases, that you these diseases will usually affect your life a lot, is not you usually affect your own life, or mood ah, family relations, old body health can body body is not healthy trouble. Well, then you think you now the whole of your own life, because these diseases will be your life is generally satisfied?
 A: Generally speaking, it is still ok, right?
 Q: that you and the children their family relationship, and neighbor relationship these all very good?
 A: Generally,
Q: Not so much, right? How you have your children. How many children are there?
 A: Three boys and a girl.
 Q: Oh, four children. Are you a local person or a nonlocal person?
 Answer: I chaoshan come over, how long did you come over? I started working out in Shenzhen when I was 50.
 Q: I came here in 1997. I have been here for a long time. There are still more than ten days, more than 20 years away.
 Q: Well. Oh, if you come here, will you come here for your own business, or.
 A: My son did a little business.
 Q: So you came here with your son, so you came along with you?
 A: Well.
 Q: Oh, so, that's like most of the family population is here,
A: Yes, son, what daughter-in-law, grandchildren, and these are all here.
 Q: What do you think is the happiest thing in your life now is what you are most satisfied with. What are you most satisfied with in your whole state of life?
 Answer: satisfaction is this has children to have a family, have grandson. That grandson even graduated from college and came out to work.

 Q: Generally speaking, I am quite satisfied with my own feelings and my life. Still satisfied? Or not how satisfied?
 A: Satisfied.
 Q: Do you think you are living with your children or not right now?
 A: Live together.
 Q: That means that you think you live now and you say you live with your children, right?
 A: Yes.
 Q: If you live together, do you think it is quite different to live together with the young people? Or do you prefer to live alone or prefer to live with your children to answer: ah. No, no, then you are all the young people are very filial piety.

 Q: Oh, that's good, because many young people are not used to living with the old people. Generally speaking, you feel very comfortable living with them, don't they?
 Answer: That is still very filial piety.
 Q: Are you usually sick, they will accompany you to see a doctor. Will come with you, won't you? For example, what you usually take medicine, they will help you to pay attention to ah or how
A: Yes, yes.
 Q: Ok, that means you think you mean you are generally satisfied, but do you really think about it? Well, from the community, or the government, or from the community or other aspects, you can make your life a little better and happier. Do you have any good advice? What can we do? From the community, ah, or from this medical staff, ah, or from the government, ah, what do you think we can do better. It can improve your satisfaction, improve your satisfaction, improve your life happiness, then you are happier.

 Answer: that generally basically in that community now, that service also generally still can ah.
 Q: Generally speaking, do you think this kind of service in their community is ok in all aspects. Does the government have any care or special care for your elderly people?
 A: Yes, yes, yes, yes,
Q: What are the benefits.

 A: For example, it was in the countryside, ah ah, I live at the age of MAO Zedong era, it was very hard at that time. Yes, yes, that substance is very good, and you will get bored. Yes, that's different now. Since I have been in the countryside since I was 65 years ago, then the government has taken care of the old man. At first, it is 50 yuan, 50 yuan a month after 55 yuan, slowly lift, slowly lift, until now there are more than 200 points.
 Q: Every month, right?
 A: Yes
Q: every month you although you this number in the outside this situation is that proportion is very small, right? But you have so much money so much old people calculate that amount is very powerful. Then you quite understand the government.
 A: Well, yes.
 Q: Do they give you, for example, the usual care is if the community pays you if the government gives you some other benefits. Ask you how well you are. That means to care about you or to check for you. You're not having anything else.

 Answer: it is a family like me, if I also had to apply at that time, is my daughter my son-in-law she is also a civil servant.

 She works in the internal department. Well, if I were ordinary and I wouldn't want that much, I would apply to the disabled government, but I was just getting right, I didn't do that. Well, even a country is not very easy either. You see, the international situation is not good, and this country costs a lot of money.
 A lot of money, if you otherwise, if the international situation is good, we can improve a little, but no way, the world is like this.
 Ask: ah, big ye, you are what work do? I think you understand the government that way,
Answer: I farm by land, farm by oneself.
 Q: Yeah, what did you don't do when you came here in' 97?

 Answer: After doing a small business, take grandchildren.
 Q: Then you just said that you just said that they are still good. But do you feel that from your own point of view, do you as an old person, do you think they can do a little better? To make you even happier? Is there any advice from here?

 A: That's not so demanding. Ok, Ok, thank you for working hard

Q: How old are you aged now?
 A: 89 years old.
 Q: Are you a local person?
 A: My hometown is in Chaoshan. I visited Shenzhen 9 years ago, and now I live in Dongguan. He had three sons and a daughter, and the sons did business, so they moved over with him.
 Q: You look so healthy.
 A: Yes, I always get sick,
Q: But you look very energetic. What are the main diseases?
 A: This heart disease. Heart at first the body is very good, 72 years old that year car accident, originally I was often often have exercise, this his foot broken, just stay at home for a year. He started having a bad heart at the end of age 73. Well, the heart is not good. I keep taking that heart medicine. Well. Just eat about four or five years, four or five years later began that stomach is bad, gastric ulcer, slowly chronic gastritis erosion. After this heart also this to this foot influence, began the heart is not good. The heart is not good to take medicine for a long time, the stomach is bad.be not allowed. Now you can't even take that cerebrovascular, nor is the arteriosclerosis.
 Ask: oh, this way, that this is really multiple serious diseases, that you these diseases will usually affect your life a lot, is not you usually affect your own life, or mood ah, family relations, old body health can body body is not healthy trouble. Well, then you think you now the whole of your own life, because these diseases will be your life is generally satisfied?
 A: Generally speaking, it is still ok, right?
 Q: that you and the children their family relationship, and neighbor relationship these all very good?
 A: Generally,
Q: Not so much, right? How you have your children. How many children are there?
 A: Three boys and a girl.
 Q: Oh, four children. Are you a local person or a nonlocal person?
 Answer: I chaoshan come over, how long did you come over? I started working out in Shenzhen when I was 50.
 Q: I came here in 1997. I have been here for a long time. There are still more than ten days, more than 20 years away.
 Q: Well. Oh, if you come here, will you come here for your own business, or.
 A: My son did a little business.
 Q: So you came here with your son, so you came along with you?
 A: Well.
 Q: Oh, so, that's like most of the family population is here,
A: Yes, son, what daughter-in-law, grandchildren, and these are all here.
 Q: What do you think is the happiest thing in your life now is what you are most satisfied with. What are you most satisfied with in your whole state of life?
 Answer: satisfaction is this has children to have a family, have grandson. That grandson even graduated from college and came out to work.

 Q: Generally speaking, I am quite satisfied with my own feelings and my life. Still satisfied? Or not how satisfied?
 A: Satisfied.
 Q: Do you think you are living with your children or not right now?
 A: Live together.
 Q: That means that you think you live now and you say you live with your children, right?
 A: Yes.
 Q: If you live together, do you think it is quite different to live together with the young people? Or do you prefer to live alone or prefer to live with your children to answer: ah. No, no, then you are all the young people are very filial piety.

 Q: Oh, that's good, because many young people are not used to living with the old people. Generally speaking, you feel very comfortable living with them, don't they?
 Answer: That is still very filial piety.
 Q: Are you usually sick, they will accompany you to see a doctor. Will come with you, won't you? For example, what you usually take medicine, they will help you to pay attention to ah or how
A: Yes, yes.
 Q: Ok, that means you think you mean you are generally satisfied, but do you really think about it? Well, from the community, or the government, or from the community or other aspects, you can make your life a little better and happier. Do you have any good advice? What can we do? From the community, ah, or from this medical staff, ah, or from the government, ah, what do you think we can do better. It can improve your satisfaction, improve your satisfaction, improve your life happiness, then you are happier.

 Answer: that generally basically in that community now, that service also generally still can ah.
 Q: Generally speaking, do you think this kind of service in their community is ok in all aspects. Does the government have any care or special care for your elderly people?
 A: Yes, yes, yes, yes,
Q: What are the benefits.

 A: For example, it was in the countryside, ah ah, I live at the age of MAO Zedong era, it was very hard at that time. Yes, yes, that substance is very good, and you will get bored. Yes, that's different now. Since I have been in the countryside since I was 65 years ago, then the government has taken care of the old man. At first, it is 50 yuan, 50 yuan a month after 55 yuan, slowly lift, slowly lift, until now there are more than 200 points.
 Q: Every month, right?
 A: Yes
Q: every month you although you this number in the outside this situation is that proportion is very small, right? But you have so much money so much old people calculate that amount is very powerful. Then you quite understand the government.
 A: Well, yes.
 Q: Do they give you, for example, the usual care is if the community pays you if the government gives you some other benefits. Ask you how well you are. That means to care about you or to check for you. You're not having anything else.

 Answer: it is a family like me, if I also had to apply at that time, is my daughter my son-in-law she is also a civil servant.

 She works in the internal department. Well, if I were ordinary and I wouldn't want that much, I would apply to the disabled government, but I was just getting right, I didn't do that. Well, even a country is not very easy either. You see, the international situation is not good, and this country costs a lot of money.
 A lot of money, if you otherwise, if the international situation is good, we can improve a little, but no way, the world is like this.
 Ask: ah, big ye, you are what work do? I think you understand the government that way,
Answer: I farm by land, farm by oneself.
 Q: Yeah, what did you don't do when you came here in' 97?

 Answer: After doing a small business, take grandchildren.
 Q: Then you just said that you just said that they are still good. But do you feel that from your own point of view, do you as an old person, do you think they can do a little better? To make you even happier? Is there any advice from here?

 A: That's not so demanding. Ok, Ok, thank you for working hard

Q: How old are you aged now?
 A: 90 years old.
 Q: Are you a local person?
 A: My hometown is in Chaoshan. I came to Shenzhen 10 years ago, and now I live in Dongguan. He had three sons and a daughter, and the sons did business, so they moved over with him.
 Q: You look so healthy.
 A: Yes, I always get sick,
Q: But you look very energetic. What are the main diseases?
 A: This heart disease. Heart at first the body is still very good, 73 years old that year car accident, originally I was often often have exercise, this his foot was broken, just stay at home for a year. He started having a bad heart at the end of age 74. Well, the heart is not good. I keep taking that heart medicine. Well. Just eat about four or five years, four or five years later began that stomach is bad, gastric ulcer, slowly chronic gastritis erosion. After this heart also this to this foot influence, began the heart is not good. The heart is not good to take medicine for a long time, the stomach is bad.be not allowed. Now you can't even take that cerebrovascular, nor is the arteriosclerosis.
 Ask: oh, this way, that this is really multiple serious diseases, that you these diseases will usually affect your life a lot, is not you usually affect your own life, or mood ah, family relations, old body health can body body is not healthy trouble. Well, then you think you now the whole of your own life, because these diseases will be your life is generally satisfied?
 A: Generally speaking, it is still ok, right?
 Q: that you and the children their family relationship, and neighbor relationship these all very good?
 A: Generally,
Q: Not so much, right? How you have your children. How many children are there?
 A: Three boys and a girl.
 Q: Oh, four children. Are you a local person or a nonlocal person?
 Answer: I chaoshan come over, how long did you come over? I started working out in Shenzhen when I was 50.
 Q: I came here in 1997. I have been here for a long time. There are still more than ten days, more than 20 years away.
 Q: Well. Oh, if you come here, will you come here for your own business, or.
 A: My son did a little business.
 Q: So you came here with your son, so you came along with you?
 A: Well.
 Q: Oh, so, that's like most of the family population is here,
A: Yes, son, what daughter-in-law, grandchildren, and these are all here.
 Q: What do you think is the happiest thing in your life now is what you are most satisfied with. What are you most satisfied with in your whole state of life?
 Answer: satisfaction is this has children to have a family, have grandson. That grandson even graduated from college and came out to work.

 Q: Generally speaking, I am quite satisfied with my own feelings and my life. Still satisfied? Or not how satisfied?
 A: Satisfied.
 Q: Do you think you are living with your children or not right now?
 A: Live together.
 Q: That means that you think you live now and you say you live with your children, right?
 A: Yes.
 Q: If you live together, do you think it is quite different to live together with the young people? Or do you prefer to live alone or prefer to live with your children to answer: ah. No, no, then you are all the young people are very filial piety.

 Q: Oh, that's good, because many young people are not used to living with the old people. Generally speaking, you feel very comfortable living with them, don't they?
 Answer: That is still very filial piety.
 Q: Are you usually sick, they will accompany you to see a doctor. Will come with you, won't you? For example, what you usually take medicine, they will help you to pay attention to ah or how
A: Yes, yes.
 Q: Ok, that means you think you mean you are generally satisfied, but do you really think about it? Well, from the community, or the government, or from the community or other aspects, you can make your life a little better and happier. Do you have any good advice? What can we do? From the community, ah, or from this medical staff, ah, or from the government, ah, what do you think we can do better. It can improve your satisfaction, improve your satisfaction, improve your life happiness, then you are happier.

 Answer: that generally basically in that community now, that service also generally still can ah.
 Q: Generally speaking, do you think this kind of service in their community is ok in all aspects. Does the government have any care or special care for your elderly people?
 A: Yes, yes, yes, yes,
Q: What are the benefits.

 A: For example, it was in the countryside, ah ah, I live at the age of MAO Zedong era, it was very hard at that time. Yes, yes, that substance is very good, and you will get bored. Yes, that's different now. Since I have been in the countryside since I was 65 years ago, then the government has taken care of the old man. At first, it is 50 yuan, 50 yuan a month after 55 yuan, slowly lift, slowly lift, until now there are more than 200 points.
 Q: Every month, right?
 A: Yes
Q: every month you although you this number in the outside this situation is that proportion is very small, right? But you have so much money so much old people calculate that amount is very powerful. Then you quite understand the government.
 A: Well, yes.
 Q: Do they give you, for example, the usual care is if the community pays you if the government gives you some other benefits. Ask you how well you are. That means to care about you or to check for you. You're not having anything else.

 Answer: it is a family like me, if I also had to apply at that time, is my daughter my son-in-law she is also a civil servant.

 She works in the internal department. Well, if I were ordinary and I wouldn't want that much, I would apply to the disabled government, but I was just getting right, I didn't do that. Well, even a country is not very easy either. You see, the international situation is not good, and this country costs a lot of money.
 A lot of money, if you otherwise, if the international situation is good, we can improve a little, but no way, the world is like this.
 Ask: ah, big ye, you are what work do? I think you understand the government that way,
Answer: I farm by land, farm by oneself.
 Q: Yeah, what did you don't do when you came here in' 97?

 Answer: After doing a small business, take grandchildren.
 Q: Then you just said that you just said that they are still good. But do you feel that from your own point of view, do you as an old person, do you think they can do a little better? To make you even happier? Is there any advice from here?

 A: That's not so demanding. Ok, Ok, thank you for working hard

Q: How old are you aged now?
 A: 81 years old.
 Q: Are you a local person?
 Answer: My hometown is in Chaoshan. I visited Shenzhen 6 years ago, and now I live in Dongguan. He had three sons and a daughter, and the sons did business, so they moved over with him.
 Q: You look so healthy.
 A: Yes, I always get sick,
Q: But you look very energetic. What are the main diseases?
 A: This heart disease. Heart at first the body is still very good, 69 years old that year car accident ah, originally I was often often have exercise ah, this his foot was broken, just stay at home for a year. I started having a bad heart at the end of my 70s. Well, the heart is not good. I keep taking that heart medicine. Well. Just eat about four or five years, four or five years later began that stomach is bad, gastric ulcer, slowly chronic gastritis erosion. After this heart also this to this foot influence, began the heart is not good. The heart is not good to take medicine for a long time, the stomach is bad.be not allowed. Now you can't even take that cerebrovascular, nor is the arteriosclerosis.
 Ask: oh, this way, that this is really multiple serious diseases, that you these diseases will usually affect your life a lot, is not you usually affect your own life, or mood ah, family relations, old body health can body body is not healthy trouble. Well, then you think you now the whole of your own life, because these diseases will be your life is generally satisfied?
 A: Generally speaking, it is still ok, right?
 Q: that you and the children their family relationship, and neighbor relationship these all very good?
 A: Generally,
Q: Not so much, right? How you have your children. How many children are there?
 A: Three boys and a girl.
 Q: Oh, four children. Are you a local person or a nonlocal person?
 Answer: I chaoshan come over, how long did you come over? I started working out in Shenzhen when I was 50.
 Q: I came here in 1997. I have been here for a long time. There are still more than ten days, more than 20 years away.
 Q: Well. Oh, if you come here, will you come here for your own business, or.
 A: My son did a little business.
 Q: So you came here with your son, so you came along with you?
 A: Well.
 Q: Oh, so, that's like most of the family population is here,
A: Yes, son, what daughter-in-law, grandchildren, and these are all here.
 Q: What do you think is the happiest thing in your life now is what you are most satisfied with. What are you most satisfied with in your whole state of life?
 Answer: satisfaction is this has children to have a family, have grandson. That grandson even graduated from college and came out to work.

 Q: Generally speaking, I am quite satisfied with my own feelings and my life. Still satisfied? Or not how satisfied?
 A: Satisfied.
 Q: Do you think you are living with your children or not right now?
 A: Live together.
 Q: That means that you think you live now and you say you live with your children, right?
 A: Yes.
 Q: If you live together, do you think it is quite different to live together with the young people? Or do you prefer to live alone or prefer to live with your children to answer: ah. No, no, then you are all the young people are very filial piety.

 Q: Oh, that's good, because many young people are not used to living with the old people. Generally speaking, you feel very comfortable living with them, don't they?
 Answer: That is still very filial piety.
 Q: Are you usually sick, they will accompany you to see a doctor. Will come with you, won't you? For example, what you usually take medicine, they will help you to pay attention to ah or how
A: Yes, yes.
 Q: Ok, that means you think you mean you are generally satisfied, but do you really think about it? Well, from the community, or the government, or from the community or other aspects, you can make your life a little better and happier. Do you have any good advice? What can we do? From the community, ah, or from this medical staff, ah, or from the government, ah, what do you think we can do better. It can improve your satisfaction, improve your satisfaction, improve your life happiness, then you are happier.

 Answer: that generally basically in that community now, that service also generally still can ah.
 Q: Generally speaking, do you think this kind of service in their community is ok in all aspects. Does the government have any care or special care for your elderly people?
 A: Yes, yes, yes, yes,
Q: What are the benefits.

 A: For example, it was in the countryside, ah ah, I live at the age of MAO Zedong era, it was very hard at that time. Yes, yes, that substance is very good, and you will get bored. Yes, that's different now. Since I have been in the countryside since I was 65 years ago, then the government has taken care of the old man. At first, it is 50 yuan, 50 yuan a month after 55 yuan, slowly lift, slowly lift, until now there are more than 200 points.
 Q: Every month, right?
 A: Yes
Q: every month you although you this number in the outside this situation is that proportion is very small, right? But you have so much money so much old people calculate that amount is very powerful. Then you quite understand the government.
 A: Well, yes.
 Q: Do they give you, for example, the usual care is if the community pays you if the government gives you some other benefits. Ask you how well you are. That means to care about you or to check for you. You're not having anything else.

 Answer: it is a family like me, if I also had to apply at that time, is my daughter my son-in-law she is also a civil servant.

 She works in the internal department. Well, if I were ordinary and I wouldn't want that much, I would apply to the disabled government, but I was just getting right, I didn't do that. Well, even a country is not very easy either. You see, the international situation is not good, and this country costs a lot of money.
 A lot of money, if you otherwise, if the international situation is good, we can improve a little, but no way, the world is like this.
 Ask: ah, big ye, you are what work do? I think you understand the government that way,
Answer: I farm by land, farm by oneself.
 Q: Yeah, what did you don't do when you came here in' 97?

 Answer: After doing a small business, take grandchildren.
 Q: Then you just said that you just said that they are still good. But do you feel that from your own point of view, do you as an old person, do you think they can do a little better? To make you even happier? Is there any advice from here?

 A: That's not so demanding. Ok, Ok, thank you for working hard

Q: How old are you aged now?
 A: 82 years old.
 Q: Are you a local person?
 Answer: My hometown is in Chaoshan. I visited Shenzhen 7 years ago, and now I live in Dongguan. He had three sons and a daughter, and the sons did business, so they moved over with him.
 Q: You look so healthy.
 A: Yes, I always get sick,
Q: But you look very energetic. What are the main diseases?
 A: This heart disease. Heart at first the body is still very good, 70 years old that year car accident, originally I was often often have exercise, this his foot broke, just stay at home for a year. I started having a bad heart at the end of age 71. Well, the heart is not good. I keep taking that heart medicine. Well. Just eat about four or five years, four or five years later began that stomach is bad, gastric ulcer, slowly chronic gastritis erosion. After this heart also this to this foot influence, began the heart is not good. The heart is not good to take medicine for a long time, the stomach is bad.be not allowed. Now you can't even take that cerebrovascular, nor is the arteriosclerosis.
 Ask: oh, this way, that this is really multiple serious diseases, that you these diseases will usually affect your life a lot, is not you usually affect your own life, or mood ah, family relations, old body health can body body is not healthy trouble. Well, then you think you now the whole of your own life, because these diseases will be your life is generally satisfied?
 A: Generally speaking, it is still ok, right?
 Q: that you and the children their family relationship, and neighbor relationship these all very good?
 A: Generally,
Q: Not so much, right? How you have your children. How many children are there?
 A: Three boys and a girl.
 Q: Oh, four children. Are you a local person or a nonlocal person?
 Answer: I chaoshan come over, how long did you come over? I started working out in Shenzhen when I was 50.
 Q: I came here in 1997. I have been here for a long time. There are still more than ten days, more than 20 years away.
 Q: Well. Oh, if you come here, will you come here for your own business, or.
 A: My son did a little business.
 Q: So you came here with your son, so you came along with you?
 A: Well.
 Q: Oh, so, that's like most of the family population is here,
A: Yes, son, what daughter-in-law, grandchildren, and these are all here.
 Q: What do you think is the happiest thing in your life now is what you are most satisfied with. What are you most satisfied with in your whole state of life?
 Answer: satisfaction is this has children to have a family, have grandson. That grandson even graduated from college and came out to work.

 Q: Generally speaking, I am quite satisfied with my own feelings and my life. Still satisfied? Or not how satisfied?
 A: Satisfied.
 Q: Do you think you are living with your children or not right now?
 A: Live together.
 Q: That means that you think you live now and you say you live with your children, right?
 A: Yes.
 Q: If you live together, do you think it is quite different to live together with the young people? Or do you prefer to live alone or prefer to live with your children to answer: ah. No, no, then you are all the young people are very filial piety.

 Q: Oh, that's good, because many young people are not used to living with the old people. Generally speaking, you feel very comfortable living with them, don't they?
 Answer: That is still very filial piety.
 Q: Are you usually sick, they will accompany you to see a doctor. Will come with you, won't you? For example, what you usually take medicine, they will help you to pay attention to ah or how
A: Yes, yes.
 Q: Ok, that means you think you mean you are generally satisfied, but do you really think about it? Well, from the community, or the government, or from the community or other aspects, you can make your life a little better and happier. Do you have any good advice? What can we do? From the community, ah, or from this medical staff, ah, or from the government, ah, what do you think we can do better. It can improve your satisfaction, improve your satisfaction, improve your life happiness, then you are happier.

 Answer: that generally basically in that community now, that service also generally still can ah.
 Q: Generally speaking, do you think this kind of service in their community is ok in all aspects. Does the government have any care or special care for your elderly people?
 A: Yes, yes, yes, yes,
Q: What are the benefits.

 A: For example, it was in the countryside, ah ah, I live at the age of MAO Zedong era, it was very hard at that time. Yes, yes, that substance is very good, and you will get bored. Yes, that's different now. Since I have been in the countryside since I was 65 years ago, then the government has taken care of the old man. At first, it is 50 yuan, 50 yuan a month after 55 yuan, slowly lift, slowly lift, until now there are more than 200 points.
 Q: Every month, right?
 A: Yes
Q: every month you although you this number in the outside this situation is that proportion is very small, right? But you have so much money so much old people calculate that amount is very powerful. Then you quite understand the government.
 A: Well, yes.
 Q: Do they give you, for example, the usual care is if the community pays you if the government gives you some other benefits. Ask you how well you are. That means to care about you or to check for you. You're not having anything else.

 Answer: it is a family like me, if I also had to apply at that time, is my daughter my son-in-law she is also a civil servant.

 She works in the internal department. Well, if I were ordinary and I wouldn't want that much, I would apply to the disabled government, but I was just getting right, I didn't do that. Well, even a country is not very easy either. You see, the international situation is not good, and this country costs a lot of money.
 A lot of money, if you otherwise, if the international situation is good, we can improve a little, but no way, the world is like this.
 Ask: ah, big ye, you are what work do? I think you understand the government that way,
Answer: I farm by land, farm by oneself.
 Q: Yeah, what did you don't do when you came here in' 97?

 Answer: After doing a small business, take grandchildren.
 Q: Then you just said that you just said that they are still good. But do you feel that from your own point of view, do you as an old person, do you think they can do a little better? To make you even happier? Is there any advice from here?

 A: That's not so demanding. Ok, Ok, thank you for working hard

Q: How old are you aged now?
 A: 83 years old.
 Q: Are you a local person?
 A: My hometown is in Chaoshan. I visited Shenzhen 8 years ago, and now I live in Dongguan. He had three sons and a daughter, and the sons did business, so they moved over with him.
 Q: You look so healthy.
 A: Yes, I always get sick,
Q: But you look very energetic. What are the main diseases?
 A: This heart disease. Heart at first the body is still very good, 71 years old that year car accident ah, originally I was often often have exercise ah, this his foot broke, just stay at home for a year. He started having a bad heart at the end of age 72. Well, the heart is not good. I keep taking that heart medicine. Well. Just eat about four or five years, four or five years later began that stomach is bad, gastric ulcer, slowly chronic gastritis erosion. After this heart also this to this foot influence, began the heart is not good. The heart is not good to take medicine for a long time, the stomach is bad.be not allowed. Now you can't even take that cerebrovascular, nor is the arteriosclerosis.
 Ask: oh, this way, that this is really multiple serious diseases, that you these diseases will usually affect your life a lot, is not you usually affect your own life, or mood ah, family relations, old body health can body body is not healthy trouble. Well, then you think you now the whole of your own life, because these diseases will be your life is generally satisfied?
 A: Generally speaking, it is still ok, right?
 Q: that you and the children their family relationship, and neighbor relationship these all very good?
 A: Generally,
Q: Not so much, right? How you have your children. How many children are there?
 A: Three boys and a girl.
 Q: Oh, four children. Are you a local person or a nonlocal person?
 Answer: I chaoshan come over, how long did you come over? I started working out in Shenzhen when I was 50.
 Q: I came here in 1997. I have been here for a long time. There are still more than ten days, more than 20 years away.
 Q: Well. Oh, if you come here, will you come here for your own business, or.
 A: My son did a little business.
 Q: So you came here with your son, so you came along with you?
 A: Well.
 Q: Oh, so, that's like most of the family population is here,
A: Yes, son, what daughter-in-law, grandchildren, and these are all here.
 Q: What do you think is the happiest thing in your life now is what you are most satisfied with. What are you most satisfied with in your whole state of life?
 Answer: satisfaction is this has children to have a family, have grandson. That grandson even graduated from college and came out to work.

 Q: Generally speaking, I am quite satisfied with my own feelings and my life. Still satisfied? Or not how satisfied?
 A: Satisfied.
 Q: Do you think you are living with your children or not right now?
 A: Live together.
 Q: That means that you think you live now and you say you live with your children, right?
 A: Yes.
 Q: If you live together, do you think it is quite different to live together with the young people? Or do you prefer to live alone or prefer to live with your children to answer: ah. No, no, then you are all the young people are very filial piety.

 Q: Oh, that's good, because many young people are not used to living with the old people. Generally speaking, you feel very comfortable living with them, don't they?
 Answer: That is still very filial piety.
 Q: Are you usually sick, they will accompany you to see a doctor. Will come with you, won't you? For example, what you usually take medicine, they will help you to pay attention to ah or how
A: Yes, yes.
 Q: Ok, that means you think you mean you are generally satisfied, but do you really think about it? Well, from the community, or the government, or from the community or other aspects, you can make your life a little better and happier. Do you have any good advice? What can we do? From the community, ah, or from this medical staff, ah, or from the government, ah, what do you think we can do better. It can improve your satisfaction, improve your satisfaction, improve your life happiness, then you are happier.

 Answer: that generally basically in that community now, that service also generally still can ah.
 Q: Generally speaking, do you think this kind of service in their community is ok in all aspects. Does the government have any care or special care for your elderly people?
 A: Yes, yes, yes, yes,
Q: What are the benefits.

 A: For example, it was in the countryside, ah ah, I live at the age of MAO Zedong era, it was very hard at that time. Yes, yes, that substance is very good, and you will get bored. Yes, that's different now. Since I have been in the countryside since I was 65 years ago, then the government has taken care of the old man. At first, it is 50 yuan, 50 yuan a month after 55 yuan, slowly lift, slowly lift, until now there are more than 200 points.
 Q: Every month, right?
 A: Yes
Q: every month you although you this number in the outside this situation is that proportion is very small, right? But you have so much money so much old people calculate that amount is very powerful. Then you quite understand the government.
 A: Well, yes.
 Q: Do they give you, for example, the usual care is if the community pays you if the government gives you some other benefits. Ask you how well you are. That means to care about you or to check for you. You're not having anything else.

 Answer: it is a family like me, if I also had to apply at that time, is my daughter my son-in-law she is also a civil servant.

 She works in the internal department. Well, if I were ordinary and I wouldn't want that much, I would apply to the disabled government, but I was just getting right, I didn't do that. Well, even a country is not very easy either. You see, the international situation is not good, and this country costs a lot of money.
 A lot of money, if you otherwise, if the international situation is good, we can improve a little, but no way, the world is like this.
 Ask: ah, big ye, you are what work do? I think you understand the government that way,
Answer: I farm by land, farm by oneself.
 Q: Yeah, what did you don't do when you came here in' 97?

 Answer: After doing a small business, take grandchildren.
 Q: Then you just said that you just said that they are still good. But do you feel that from your own point of view, do you as an old person, do you think they can do a little better? To make you even happier? Is there any advice from here?

 A: That's not so demanding. Ok, Ok, thank you for working hard

Q: How old are you aged now?
 A: 84 years old.
 Q: Are you a local person?
 A: My hometown is in Chaoshan. I visited Shenzhen 9 years ago, and now I live in Dongguan. He had three sons and a daughter, and the sons did business, so they moved over with him.
 Q: You look so healthy.
 A: Yes, I always get sick,
Q: But you look very energetic. What are the main diseases?
 A: This heart disease. Heart at first the body is very good, 72 years old that year car accident, originally I was often often have exercise, this his foot broken, just stay at home for a year. He started having a bad heart at the end of age 73. Well, the heart is not good. I keep taking that heart medicine. Well. Just eat about four or five years, four or five years later began that stomach is bad, gastric ulcer, slowly chronic gastritis erosion. After this heart also this to this foot influence, began the heart is not good. The heart is not good to take medicine for a long time, the stomach is bad.be not allowed. Now you can't even take that cerebrovascular, nor is the arteriosclerosis.
 Ask: oh, this way, that this is really multiple serious diseases, that you these diseases will usually affect your life a lot, is not you usually affect your own life, or mood ah, family relations, old body health can body body is not healthy trouble. Well, then you think you now the whole of your own life, because these diseases will be your life is generally satisfied?
 A: Generally speaking, it is still ok, right?
 Q: that you and the children their family relationship, and neighbor relationship these all very good?
 A: Generally,
Q: Not so much, right? How you have your children. How many children are there?
 A: Three boys and a girl.
 Q: Oh, four children. Are you a local person or a nonlocal person?
 Answer: I chaoshan come over, how long did you come over? I started working out in Shenzhen when I was 50.
 Q: I came here in 1997. I have been here for a long time. There are still more than ten days, more than 20 years away.
 Q: Well. Oh, if you come here, will you come here for your own business, or.
 A: My son did a little business.
 Q: So you came here with your son, so you came along with you?
 A: Well.
 Q: Oh, so, that's like most of the family population is here,
A: Yes, son, what daughter-in-law, grandchildren, and these are all here.
 Q: What do you think is the happiest thing in your life now is what you are most satisfied with. What are you most satisfied with in your whole state of life?
 Answer: satisfaction is this has children to have a family, have grandson. That grandson even graduated from college and came out to work.

 Q: Generally speaking, I am quite satisfied with my own feelings and my life. Still satisfied? Or not how satisfied?
 A: Satisfied.
 Q: Do you think you are living with your children or not right now?
 A: Live together.
 Q: That means that you think you live now and you say you live with your children, right?
 A: Yes.
 Q: If you live together, do you think it is quite different to live together with the young people? Or do you prefer to live alone or prefer to live with your children to answer: ah. No, no, then you are all the young people are very filial piety.

 Q: Oh, that's good, because many young people are not used to living with the old people. Generally speaking, you feel very comfortable living with them, don't they?
 Answer: That is still very filial piety.
 Q: Are you usually sick, they will accompany you to see a doctor. Will come with you, won't you? For example, what you usually take medicine, they will help you to pay attention to ah or how
A: Yes, yes.
 Q: Ok, that means you think you mean you are generally satisfied, but do you really think about it? Well, from the community, or the government, or from the community or other aspects, you can make your life a little better and happier. Do you have any good advice? What can we do? From the community, ah, or from this medical staff, ah, or from the government, ah, what do you think we can do better. It can improve your satisfaction, improve your satisfaction, improve your life happiness, then you are happier.

 Answer: that generally basically in that community now, that service also generally still can ah.
 Q: Generally speaking, do you think this kind of service in their community is ok in all aspects. Does the government have any care or special care for your elderly people?
 A: Yes, yes, yes, yes,
Q: What are the benefits.

 A: For example, it was in the countryside, ah ah, I live at the age of MAO Zedong era, it was very hard at that time. Yes, yes, that substance is very good, and you will get bored. Yes, that's different now. Since I have been in the countryside since I was 65 years ago, then the government has taken care of the old man. At first, it is 50 yuan, 50 yuan a month after 55 yuan, slowly lift, slowly lift, until now there are more than 200 points.
 Q: Every month, right?
 A: Yes
Q: every month you although you this number in the outside this situation is that proportion is very small, right? But you have so much money so much old people calculate that amount is very powerful. Then you quite understand the government.
 A: Well, yes.
 Q: Do they give you, for example, the usual care is if the community pays you if the government gives you some other benefits. Ask you how well you are. That means to care about you or to check for you. You're not having anything else.

 Answer: it is a family like me, if I also had to apply at that time, is my daughter my son-in-law she is also a civil servant.

 She works in the internal department. Well, if I were ordinary and I wouldn't want that much, I would apply to the disabled government, but I was just getting right, I didn't do that. Well, even a country is not very easy either. You see, the international situation is not good, and this country costs a lot of money.
 A lot of money, if you otherwise, if the international situation is good, we can improve a little, but no way, the world is like this.
 Ask: ah, big ye, you are what work do? I think you understand the government that way,
Answer: I farm by land, farm by oneself.
 Q: Yeah, what did you don't do when you came here in' 97?

 Answer: After doing a small business, take grandchildren.
 Q: Then you just said that you just said that they are still good. But do you feel that from your own point of view, do you as an old person, do you think they can do a little better? To make you even happier? Is there any advice from here?

 A: That's not so demanding. Ok, Ok, thank you for working hard

Q: How old are you aged now?
 A: 85 years old.
 Q: Are you a local person?
 A: My hometown is in Chaoshan. I came to Shenzhen 10 years ago, and now I live in Dongguan. He had three sons and a daughter, and the sons did business, so they moved over with him.
 Q: You look so healthy.
 A: Yes, I always get sick,
Q: But you look very energetic. What are the main diseases?
 A: This heart disease. Heart at first the body is still very good, 73 years old that year car accident, originally I was often often have exercise, this his foot was broken, just stay at home for a year. He started having a bad heart at the end of age 74. Well, the heart is not good. I keep taking that heart medicine. Well. Just eat about four or five years, four or five years later began that stomach is bad, gastric ulcer, slowly chronic gastritis erosion. After this heart also this to this foot influence, began the heart is not good. The heart is not good to take medicine for a long time, the stomach is bad.be not allowed. Now you can't even take that cerebrovascular, nor is the arteriosclerosis.
 Ask: oh, this way, that this is really multiple serious diseases, that you these diseases will usually affect your life a lot, is not you usually affect your own life, or mood ah, family relations, old body health can body body is not healthy trouble. Well, then you think you now the whole of your own life, because these diseases will be your life is generally satisfied?
 A: Generally speaking, it is still ok, right?
 Q: that you and the children their family relationship, and neighbor relationship these all very good?
 A: Generally,
Q: Not so much, right? How you have your children. How many children are there?
 A: Three boys and a girl.
 Q: Oh, four children. Are you a local person or a nonlocal person?
 Answer: I chaoshan come over, how long did you come over? I started working out in Shenzhen when I was 50.
 Q: I came here in 1997. I have been here for a long time. There are still more than ten days, more than 20 years away.
 Q: Well. Oh, if you come here, will you come here for your own business, or.
 A: My son did a little business.
 Q: So you came here with your son, so you came along with you?
 A: Well.
 Q: Oh, so, that's like most of the family population is here,
A: Yes, son, what daughter-in-law, grandchildren, and these are all here.
 Q: What do you think is the happiest thing in your life now is what you are most satisfied with. What are you most satisfied with in your whole state of life?
 Answer: satisfaction is this has children to have a family, have grandson. That grandson even graduated from college and came out to work.

 Q: Generally speaking, I am quite satisfied with my own feelings and my life. Still satisfied? Or not how satisfied?
 A: Satisfied.
 Q: Do you think you are living with your children or not right now?
 A: Live together.
 Q: That means that you think you live now and you say you live with your children, right?
 A: Yes.
 Q: If you live together, do you think it is quite different to live together with the young people? Or do you prefer to live alone or prefer to live with your children to answer: ah. No, no, then you are all the young people are very filial piety.

 Q: Oh, that's good, because many young people are not used to living with the old people. Generally speaking, you feel very comfortable living with them, don't they?
 Answer: That is still very filial piety.
 Q: Are you usually sick, they will accompany you to see a doctor. Will come with you, won't you? For example, what you usually take medicine, they will help you to pay attention to ah or how
A: Yes, yes.
 Q: Ok, that means you think you mean you are generally satisfied, but do you really think about it? Well, from the community, or the government, or from the community or other aspects, you can make your life a little better and happier. Do you have any good advice? What can we do? From the community, ah, or from this medical staff, ah, or from the government, ah, what do you think we can do better. It can improve your satisfaction, improve your satisfaction, improve your life happiness, then you are happier.

 Answer: that generally basically in that community now, that service also generally still can ah.
 Q: Generally speaking, do you think this kind of service in their community is ok in all aspects. Does the government have any care or special care for your elderly people?
 A: Yes, yes, yes, yes,
Q: What are the benefits.

 A: For example, it was in the countryside, ah ah, I live at the age of MAO Zedong era, it was very hard at that time. Yes, yes, that substance is very good, and you will get bored. Yes, that's different now. Since I have been in the countryside since I was 65 years ago, then the government has taken care of the old man. At first, it is 50 yuan, 50 yuan a month after 55 yuan, slowly lift, slowly lift, until now there are more than 200 points.
 Q: Every month, right?
 A: Yes
Q: every month you although you this number in the outside this situation is that proportion is very small, right? But you have so much money so much old people calculate that amount is very powerful. Then you quite understand the government.
 A: Well, yes.
 Q: Do they give you, for example, the usual care is if the community pays you if the government gives you some other benefits. Ask you how well you are. That means to care about you or to check for you. You're not having anything else.

 Answer: it is a family like me, if I also had to apply at that time, is my daughter my son-in-law she is also a civil servant.

 She works in the internal department. Well, if I were ordinary and I wouldn't want that much, I would apply to the disabled government, but I was just getting right, I didn't do that. Well, even a country is not very easy either. You see, the international situation is not good, and this country costs a lot of money.
 A lot of money, if you otherwise, if the international situation is good, we can improve a little, but no way, the world is like this.
 Ask: ah, big ye, you are what work do? I think you understand the government that way,
Answer: I farm by land, farm by oneself.
 Q: Yeah, what did you don't do when you came here in' 97?

 Answer: After doing a small business, take grandchildren.
 Q: Then you just said that you just said that they are still good. But do you feel that from your own point of view, do you as an old person, do you think they can do a little better? To make you even happier? Is there any advice from here?

 A: That's not so demanding. Ok, Ok, thank you for working hard

Q: How old are you aged now?
 A: 86 years old.
 Q: Are you a local person?
 Answer: My hometown is in Chaoshan. I visited Shenzhen 6 years ago, and now I live in Dongguan. He had three sons and a daughter, and the sons did business, so they moved over with him.
 Q: You look so healthy.
 A: Yes, I always get sick,
Q: But you look very energetic. What are the main diseases?
 A: This heart disease. Heart at first the body is still very good, 69 years old that year car accident ah, originally I was often often have exercise ah, this his foot was broken, just stay at home for a year. I started having a bad heart at the end of my 70s. Well, the heart is not good. I keep taking that heart medicine. Well. Just eat about four or five years, four or five years later began that stomach is bad, gastric ulcer, slowly chronic gastritis erosion. After this heart also this to this foot influence, began the heart is not good. The heart is not good to take medicine for a long time, the stomach is bad.be not allowed. Now you can't even take that cerebrovascular, nor is the arteriosclerosis.
 Ask: oh, this way, that this is really multiple serious diseases, that you these diseases will usually affect your life a lot, is not you usually affect your own life, or mood ah, family relations, old body health can body body is not healthy trouble. Well, then you think you now the whole of your own life, because these diseases will be your life is generally satisfied?
 A: Generally speaking, it is still ok, right?
 Q: that you and the children their family relationship, and neighbor relationship these all very good?
 A: Generally,
Q: Not so much, right? How you have your children. How many children are there?
 A: Three boys and a girl.
 Q: Oh, four children. Are you a local person or a nonlocal person?
 Answer: I chaoshan come over, how long did you come over? I started working out in Shenzhen when I was 50.
 Q: I came here in 1997. I have been here for a long time. There are still more than ten days, more than 20 years away.
 Q: Well. Oh, if you come here, will you come here for your own business, or.
 A: My son did a little business.
 Q: So you came here with your son, so you came along with you?
 A: Well.
 Q: Oh, so, that's like most of the family population is here,
A: Yes, son, what daughter-in-law, grandchildren, and these are all here.
 Q: What do you think is the happiest thing in your life now is what you are most satisfied with. What are you most satisfied with in your whole state of life?
 Answer: satisfaction is this has children to have a family, have grandson. That grandson even graduated from college and came out to work.

 Q: Generally speaking, I am quite satisfied with my own feelings and my life. Still satisfied? Or not how satisfied?
 A: Satisfied.
 Q: Do you think you are living with your children or not right now?
 A: Live together.
 Q: That means that you think you live now and you say you live with your children, right?
 A: Yes.
 Q: If you live together, do you think it is quite different to live together with the young people? Or do you prefer to live alone or prefer to live with your children to answer: ah. No, no, then you are all the young people are very filial piety.

 Q: Oh, that's good, because many young people are not used to living with the old people. Generally speaking, you feel very comfortable living with them, don't they?
 Answer: That is still very filial piety.
 Q: Are you usually sick, they will accompany you to see a doctor. Will come with you, won't you? For example, what you usually take medicine, they will help you to pay attention to ah or how
A: Yes, yes.
 Q: Ok, that means you think you mean you are generally satisfied, but do you really think about it? Well, from the community, or the government, or from the community or other aspects, you can make your life a little better and happier. Do you have any good advice? What can we do? From the community, ah, or from this medical staff, ah, or from the government, ah, what do you think we can do better. It can improve your satisfaction, improve your satisfaction, improve your life happiness, then you are happier.

 Answer: that generally basically in that community now, that service also generally still can ah.
 Q: Generally speaking, do you think this kind of service in their community is ok in all aspects. Does the government have any care or special care for your elderly people?
 A: Yes, yes, yes, yes,
Q: What are the benefits.

 A: For example, it was in the countryside, ah ah, I live at the age of MAO Zedong era, it was very hard at that time. Yes, yes, that substance is very good, and you will get bored. Yes, that's different now. Since I have been in the countryside since I was 65 years ago, then the government has taken care of the old man. At first, it is 50 yuan, 50 yuan a month after 55 yuan, slowly lift, slowly lift, until now there are more than 200 points.
 Q: Every month, right?
 A: Yes
Q: every month you although you this number in the outside this situation is that proportion is very small, right? But you have so much money so much old people calculate that amount is very powerful. Then you quite understand the government.
 A: Well, yes.
 Q: Do they give you, for example, the usual care is if the community pays you if the government gives you some other benefits. Ask you how well you are. That means to care about you or to check for you. You're not having anything else.

 Answer: it is a family like me, if I also had to apply at that time, is my daughter my son-in-law she is also a civil servant.

 She works in the internal department. Well, if I were ordinary and I wouldn't want that much, I would apply to the disabled government, but I was just getting right, I didn't do that. Well, even a country is not very easy either. You see, the international situation is not good, and this country costs a lot of money.
 A lot of money, if you otherwise, if the international situation is good, we can improve a little, but no way, the world is like this.
 Ask: ah, big ye, you are what work do? I think you understand the government that way,
Answer: I farm by land, farm by oneself.
 Q: Yeah, what did you don't do when you came here in' 97?

 Answer: After doing a small business, take grandchildren.
 Q: Then you just said that you just said that they are still good. But do you feel that from your own point of view, do you as an old person, do you think they can do a little better? To make you even happier? Is there any advice from here?

 A: That's not so demanding. Ok, Ok, thank you for working hard

Q: How old are you aged now?
 A: 87 years old.
 Q: Are you a local person?
 Answer: My hometown is in Chaoshan. I visited Shenzhen 7 years ago, and now I live in Dongguan. He had three sons and a daughter, and the sons did business, so they moved over with him.
 Q: You look so healthy.
 A: Yes, I always get sick,
Q: But you look very energetic. What are the main diseases?
 A: This heart disease. Heart at first the body is still very good, 70 years old that year car accident, originally I was often often have exercise, this his foot broke, just stay at home for a year. I started having a bad heart at the end of age 71. Well, the heart is not good. I keep taking that heart medicine. Well. Just eat about four or five years, four or five years later began that stomach is bad, gastric ulcer, slowly chronic gastritis erosion. After this heart also this to this foot influence, began the heart is not good. The heart is not good to take medicine for a long time, the stomach is bad.be not allowed. Now you can't even take that cerebrovascular, nor is the arteriosclerosis.
 Ask: oh, this way, that this is really multiple serious diseases, that you these diseases will usually affect your life a lot, is not you usually affect your own life, or mood ah, family relations, old body health can body body is not healthy trouble. Well, then you think you now the whole of your own life, because these diseases will be your life is generally satisfied?
 A: Generally speaking, it is still ok, right?
 Q: that you and the children their family relationship, and neighbor relationship these all very good?
 A: Generally,
Q: Not so much, right? How you have your children. How many children are there?
 A: Three boys and a girl.
 Q: Oh, four children. Are you a local person or a nonlocal person?
 Answer: I chaoshan come over, how long did you come over? I started working out in Shenzhen when I was 50.
 Q: I came here in 1997. I have been here for a long time. There are still more than ten days, more than 20 years away.
 Q: Well. Oh, if you come here, will you come here for your own business, or.
 A: My son did a little business.
 Q: So you came here with your son, so you came along with you?
 A: Well.
 Q: Oh, so, that's like most of the family population is here,
A: Yes, son, what daughter-in-law, grandchildren, and these are all here.
 Q: What do you think is the happiest thing in your life now is what you are most satisfied with. What are you most satisfied with in your whole state of life?
 Answer: satisfaction is this has children to have a family, have grandson. That grandson even graduated from college and came out to work.

 Q: Generally speaking, I am quite satisfied with my own feelings and my life. Still satisfied? Or not how satisfied?
 A: Satisfied.
 Q: Do you think you are living with your children or not right now?
 A: Live together.
 Q: That means that you think you live now and you say you live with your children, right?
 A: Yes.
 Q: If you live together, do you think it is quite different to live together with the young people? Or do you prefer to live alone or prefer to live with your children to answer: ah. No, no, then you are all the young people are very filial piety.

 Q: Oh, that's good, because many young people are not used to living with the old people. Generally speaking, you feel very comfortable living with them, don't they?
 Answer: That is still very filial piety.
 Q: Are you usually sick, they will accompany you to see a doctor. Will come with you, won't you? For example, what you usually take medicine, they will help you to pay attention to ah or how
A: Yes, yes.
 Q: Ok, that means you think you mean you are generally satisfied, but do you really think about it? Well, from the community, or the government, or from the community or other aspects, you can make your life a little better and happier. Do you have any good advice? What can we do? From the community, ah, or from this medical staff, ah, or from the government, ah, what do you think we can do better. It can improve your satisfaction, improve your satisfaction, improve your life happiness, then you are happier.

 Answer: that generally basically in that community now, that service also generally still can ah.
 Q: Generally speaking, do you think this kind of service in their community is ok in all aspects. Does the government have any care or special care for your elderly people?
 A: Yes, yes, yes, yes,
Q: What are the benefits.

 A: For example, it was in the countryside, ah ah, I live at the age of MAO Zedong era, it was very hard at that time. Yes, yes, that substance is very good, and you will get bored. Yes, that's different now. Since I have been in the countryside since I was 65 years ago, then the government has taken care of the old man. At first, it is 50 yuan, 50 yuan a month after 55 yuan, slowly lift, slowly lift, until now there are more than 200 points.
 Q: Every month, right?
 A: Yes
Q: every month you although you this number in the outside this situation is that proportion is very small, right? But you have so much money so much old people calculate that amount is very powerful. Then you quite understand the government.
 A: Well, yes.
 Q: Do they give you, for example, the usual care is if the community pays you if the government gives you some other benefits. Ask you how well you are. That means to care about you or to check for you. You're not having anything else.

 Answer: it is a family like me, if I also had to apply at that time, is my daughter my son-in-law she is also a civil servant.

 She works in the internal department. Well, if I were ordinary and I wouldn't want that much, I would apply to the disabled government, but I was just getting right, I didn't do that. Well, even a country is not very easy either. You see, the international situation is not good, and this country costs a lot of money.
 A lot of money, if you otherwise, if the international situation is good, we can improve a little, but no way, the world is like this.
 Ask: ah, big ye, you are what work do? I think you understand the government that way,
Answer: I farm by land, farm by oneself.
 Q: Yeah, what did you don't do when you came here in' 97?

 Answer: After doing a small business, take grandchildren.
 Q: Then you just said that you just said that they are still good. But do you feel that from your own point of view, do you as an old person, do you think they can do a little better? To make you even happier? Is there any advice from here?

 A: That's not so demanding. Ok, Ok, thank you for working hard

Q: How old are you aged now?
 A: 88 years old.
 Q: Are you a local person?
 A: My hometown is in Chaoshan. I visited Shenzhen 8 years ago, and now I live in Dongguan. He had three sons and a daughter, and the sons did business, so they moved over with him.
 Q: You look so healthy.
 A: Yes, I always get sick,
Q: But you look very energetic. What are the main diseases?
 A: This heart disease. Heart at first the body is still very good, 71 years old that year car accident ah, originally I was often often have exercise ah, this his foot broke, just stay at home for a year. He started having a bad heart at the end of age 72. Well, the heart is not good. I keep taking that heart medicine. Well. Just eat about four or five years, four or five years later began that stomach is bad, gastric ulcer, slowly chronic gastritis erosion. After this heart also this to this foot influence, began the heart is not good. The heart is not good to take medicine for a long time, the stomach is bad.be not allowed. Now you can't even take that cerebrovascular, nor is the arteriosclerosis.
 Ask: oh, this way, that this is really multiple serious diseases, that you these diseases will usually affect your life a lot, is not you usually affect your own life, or mood ah, family relations, old body health can body body is not healthy trouble. Well, then you think you now the whole of your own life, because these diseases will be your life is generally satisfied?
 A: Generally speaking, it is still ok, right?
 Q: that you and the children their family relationship, and neighbor relationship these all very good?
 A: Generally,
Q: Not so much, right? How you have your children. How many children are there?
 A: Three boys and a girl.
 Q: Oh, four children. Are you a local person or a nonlocal person?
 Answer: I chaoshan come over, how long did you come over? I started working out in Shenzhen when I was 50.
 Q: I came here in 1997. I have been here for a long time. There are still more than ten days, more than 20 years away.
 Q: Well. Oh, if you come here, will you come here for your own business, or.
 A: My son did a little business.
 Q: So you came here with your son, so you came along with you?
 A: Well.
 Q: Oh, so, that's like most of the family population is here,
A: Yes, son, what daughter-in-law, grandchildren, and these are all here.
 Q: What do you think is the happiest thing in your life now is what you are most satisfied with. What are you most satisfied with in your whole state of life?
 Answer: satisfaction is this has children to have a family, have grandson. That grandson even graduated from college and came out to work.

 Q: Generally speaking, I am quite satisfied with my own feelings and my life. Still satisfied? Or not how satisfied?
 A: Satisfied.
 Q: Do you think you are living with your children or not right now?
 A: Live together.
 Q: That means that you think you live now and you say you live with your children, right?
 A: Yes.
 Q: If you live together, do you think it is quite different to live together with the young people? Or do you prefer to live alone or prefer to live with your children to answer: ah. No, no, then you are all the young people are very filial piety.

 Q: Oh, that's good, because many young people are not used to living with the old people. Generally speaking, you feel very comfortable living with them, don't they?
 Answer: That is still very filial piety.
 Q: Are you usually sick, they will accompany you to see a doctor. Will come with you, won't you? For example, what you usually take medicine, they will help you to pay attention to ah or how
A: Yes, yes.
 Q: Ok, that means you think you mean you are generally satisfied, but do you really think about it? Well, from the community, or the government, or from the community or other aspects, you can make your life a little better and happier. Do you have any good advice? What can we do? From the community, ah, or from this medical staff, ah, or from the government, ah, what do you think we can do better. It can improve your satisfaction, improve your satisfaction, improve your life happiness, then you are happier.

 Answer: that generally basically in that community now, that service also generally still can ah.
 Q: Generally speaking, do you think this kind of service in their community is ok in all aspects. Does the government have any care or special care for your elderly people?
 A: Yes, yes, yes, yes,
Q: What are the benefits.

 A: For example, it was in the countryside, ah ah, I live at the age of MAO Zedong era, it was very hard at that time. Yes, yes, that substance is very good, and you will get bored. Yes, that's different now. Since I have been in the countryside since I was 65 years ago, then the government has taken care of the old man. At first, it is 50 yuan, 50 yuan a month after 55 yuan, slowly lift, slowly lift, until now there are more than 200 points.
 Q: Every month, right?
 A: Yes
Q: every month you although you this number in the outside this situation is that proportion is very small, right? But you have so much money so much old people calculate that amount is very powerful. Then you quite understand the government.
 A: Well, yes.
 Q: Do they give you, for example, the usual care is if the community pays you if the government gives you some other benefits. Ask you how well you are. That means to care about you or to check for you. You're not having anything else.

 Answer: it is a family like me, if I also had to apply at that time, is my daughter my son-in-law she is also a civil servant.

 She works in the internal department. Well, if I were ordinary and I wouldn't want that much, I would apply to the disabled government, but I was just getting right, I didn't do that. Well, even a country is not very easy either. You see, the international situation is not good, and this country costs a lot of money.
 A lot of money, if you otherwise, if the international situation is good, we can improve a little, but no way, the world is like this.
 Ask: ah, big ye, you are what work do? I think you understand the government that way,
Answer: I farm by land, farm by oneself.
 Q: Yeah, what did you don't do when you came here in' 97?

 Answer: After doing a small business, take grandchildren.
 Q: Then you just said that you just said that they are still good. But do you feel that from your own point of view, do you as an old person, do you think they can do a little better? To make you even happier? Is there any advice from here?

 A: That's not so demanding. Ok, Ok, thank you for working hard

Q: How old are you aged now?
 A: 89 years old.
 Q: Are you a local person?
 A: My hometown is in Chaoshan. I visited Shenzhen 9 years ago, and now I live in Dongguan. He had three sons and a daughter, and the sons did business, so they moved over with him.
 Q: You look so healthy.
 A: Yes, I always get sick,
Q: But you look very energetic. What are the main diseases?
 A: This heart disease. Heart at first the body is very good, 72 years old that year car accident, originally I was often often have exercise, this his foot broken, just stay at home for a year. He started having a bad heart at the end of age 73. Well, the heart is not good. I keep taking that heart medicine. Well. Just eat about four or five years, four or five years later began that stomach is bad, gastric ulcer, slowly chronic gastritis erosion. After this heart also this to this foot influence, began the heart is not good. The heart is not good to take medicine for a long time, the stomach is bad.be not allowed. Now you can't even take that cerebrovascular, nor is the arteriosclerosis.
 Ask: oh, this way, that this is really multiple serious diseases, that you these diseases will usually affect your life a lot, is not you usually affect your own life, or mood ah, family relations, old body health can body body is not healthy trouble. Well, then you think you now the whole of your own life, because these diseases will be your life is generally satisfied?
 A: Generally speaking, it is still ok, right?
 Q: that you and the children their family relationship, and neighbor relationship these all very good?
 A: Generally,
Q: Not so much, right? How you have your children. How many children are there?
 A: Three boys and a girl.
 Q: Oh, four children. Are you a local person or a nonlocal person?
 Answer: I chaoshan come over, how long did you come over? I started working out in Shenzhen when I was 50.
 Q: I came here in 1997. I have been here for a long time. There are still more than ten days, more than 20 years away.
 Q: Well. Oh, if you come here, will you come here for your own business, or.
 A: My son did a little business.
 Q: So you came here with your son, so you came along with you?
 A: Well.
 Q: Oh, so, that's like most of the family population is here,
A: Yes, son, what daughter-in-law, grandchildren, and these are all here.
 Q: What do you think is the happiest thing in your life now is what you are most satisfied with. What are you most satisfied with in your whole state of life?
 Answer: satisfaction is this has children to have a family, have grandson. That grandson even graduated from college and came out to work.

 Q: Generally speaking, I am quite satisfied with my own feelings and my life. Still satisfied? Or not how satisfied?
 A: Satisfied.
 Q: Do you think you are living with your children or not right now?
 A: Live together.
 Q: That means that you think you live now and you say you live with your children, right?
 A: Yes.
 Q: If you live together, do you think it is quite different to live together with the young people? Or do you prefer to live alone or prefer to live with your children to answer: ah. No, no, then you are all the young people are very filial piety.

 Q: Oh, that's good, because many young people are not used to living with the old people. Generally speaking, you feel very comfortable living with them, don't they?
 Answer: That is still very filial piety.
 Q: Are you usually sick, they will accompany you to see a doctor. Will come with you, won't you? For example, what you usually take medicine, they will help you to pay attention to ah or how
A: Yes, yes.
 Q: Ok, that means you think you mean you are generally satisfied, but do you really think about it? Well, from the community, or the government, or from the community or other aspects, you can make your life a little better and happier. Do you have any good advice? What can we do? From the community, ah, or from this medical staff, ah, or from the government, ah, what do you think we can do better. It can improve your satisfaction, improve your satisfaction, improve your life happiness, then you are happier.

 Answer: that generally basically in that community now, that service also generally still can ah.
 Q: Generally speaking, do you think this kind of service in their community is ok in all aspects. Does the government have any care or special care for your elderly people?
 A: Yes, yes, yes, yes,
Q: What are the benefits.

 A: For example, it was in the countryside, ah ah, I live at the age of MAO Zedong era, it was very hard at that time. Yes, yes, that substance is very good, and you will get bored. Yes, that's different now. Since I have been in the countryside since I was 65 years ago, then the government has taken care of the old man. At first, it is 50 yuan, 50 yuan a month after 55 yuan, slowly lift, slowly lift, until now there are more than 200 points.
 Q: Every month, right?
 A: Yes
Q: every month you although you this number in the outside this situation is that proportion is very small, right? But you have so much money so much old people calculate that amount is very powerful. Then you quite understand the government.
 A: Well, yes.
 Q: Do they give you, for example, the usual care is if the community pays you if the government gives you some other benefits. Ask you how well you are. That means to care about you or to check for you. You're not having anything else.

 Answer: it is a family like me, if I also had to apply at that time, is my daughter my son-in-law she is also a civil servant.

 She works in the internal department. Well, if I were ordinary and I wouldn't want that much, I would apply to the disabled government, but I was just getting right, I didn't do that. Well, even a country is not very easy either. You see, the international situation is not good, and this country costs a lot of money.
 A lot of money, if you otherwise, if the international situation is good, we can improve a little, but no way, the world is like this.
 Ask: ah, big ye, you are what work do? I think you understand the government that way,
Answer: I farm by land, farm by oneself.
 Q: Yeah, what did you don't do when you came here in' 97?

 Answer: After doing a small business, take grandchildren.
 Q: Then you just said that you just said that they are still good. But do you feel that from your own point of view, do you as an old person, do you think they can do a little better? To make you even happier? Is there any advice from here?

 A: That's not so demanding. Ok, Ok, thank you for working hard

Q: How old are you aged now?
 A: 90 years old.
 Q: Are you a local person?
 A: My hometown is in Chaoshan. I came to Shenzhen 10 years ago, and now I live in Dongguan. He had three sons and a daughter, and the sons did business, so they moved over with him.
 Q: You look so healthy.
 A: Yes, I always get sick,
Q: But you look very energetic. What are the main diseases?
 A: This heart disease. Heart at first the body is still very good, 73 years old that year car accident, originally I was often often have exercise, this his foot was broken, just stay at home for a year. He started having a bad heart at the end of age 74. Well, the heart is not good. I keep taking that heart medicine. Well. Just eat about four or five years, four or five years later began that stomach is bad, gastric ulcer, slowly chronic gastritis erosion. After this heart also this to this foot influence, began the heart is not good. The heart is not good to take medicine for a long time, the stomach is bad.be not allowed. Now you can't even take that cerebrovascular, nor is the arteriosclerosis.
 Ask: oh, this way, that this is really multiple serious diseases, that you these diseases will usually affect your life a lot, is not you usually affect your own life, or mood ah, family relations, old body health can body body is not healthy trouble. Well, then you think you now the whole of your own life, because these diseases will be your life is generally satisfied?
 A: Generally speaking, it is still ok, right?
 Q: that you and the children their family relationship, and neighbor relationship these all very good?
 A: Generally,
Q: Not so much, right? How you have your children. How many children are there?
 A: Three boys and a girl.
 Q: Oh, four children. Are you a local person or a nonlocal person?
 Answer: I chaoshan come over, how long did you come over? I started working out in Shenzhen when I was 50.
 Q: I came here in 1997. I have been here for a long time. There are still more than ten days, more than 20 years away.
 Q: Well. Oh, if you come here, will you come here for your own business, or.
 A: My son did a little business.
 Q: So you came here with your son, so you came along with you?
 A: Well.
 Q: Oh, so, that's like most of the family population is here,
A: Yes, son, what daughter-in-law, grandchildren, and these are all here.
 Q: What do you think is the happiest thing in your life now is what you are most satisfied with. What are you most satisfied with in your whole state of life?
 Answer: satisfaction is this has children to have a family, have grandson. That grandson even graduated from college and came out to work.

 Q: Generally speaking, I am quite satisfied with my own feelings and my life. Still satisfied? Or not how satisfied?
 A: Satisfied.
 Q: Do you think you are living with your children or not right now?
 A: Live together.
 Q: That means that you think you live now and you say you live with your children, right?
 A: Yes.
 Q: If you live together, do you think it is quite different to live together with the young people? Or do you prefer to live alone or prefer to live with your children to answer: ah. No, no, then you are all the young people are very filial piety.

 Q: Oh, that's good, because many young people are not used to living with the old people. Generally speaking, you feel very comfortable living with them, don't they?
 Answer: That is still very filial piety.
 Q: Are you usually sick, they will accompany you to see a doctor. Will come with you, won't you? For example, what you usually take medicine, they will help you to pay attention to ah or how
A: Yes, yes.
 Q: Ok, that means you think you mean you are generally satisfied, but do you really think about it? Well, from the community, or the government, or from the community or other aspects, you can make your life a little better and happier. Do you have any good advice? What can we do? From the community, ah, or from this medical staff, ah, or from the government, ah, what do you think we can do better. It can improve your satisfaction, improve your satisfaction, improve your life happiness, then you are happier.

 Answer: that generally basically in that community now, that service also generally still can ah.
 Q: Generally speaking, do you think this kind of service in their community is ok in all aspects. Does the government have any care or special care for your elderly people?
 A: Yes, yes, yes, yes,
Q: What are the benefits.

 A: For example, it was in the countryside, ah ah, I live at the age of MAO Zedong era, it was very hard at that time. Yes, yes, that substance is very good, and you will get bored. Yes, that's different now. Since I have been in the countryside since I was 65 years ago, then the government has taken care of the old man. At first, it is 50 yuan, 50 yuan a month after 55 yuan, slowly lift, slowly lift, until now there are more than 200 points.
 Q: Every month, right?
 A: Yes
Q: every month you although you this number in the outside this situation is that proportion is very small, right? But you have so much money so much old people calculate that amount is very powerful. Then you quite understand the government.
 A: Well, yes.
 Q: Do they give you, for example, the usual care is if the community pays you if the government gives you some other benefits. Ask you how well you are. That means to care about you or to check for you. You're not having anything else.

 Answer: it is a family like me, if I also had to apply at that time, is my daughter my son-in-law she is also a civil servant.

 She works in the internal department. Well, if I were ordinary and I wouldn't want that much, I would apply to the disabled government, but I was just getting right, I didn't do that. Well, even a country is not very easy either. You see, the international situation is not good, and this country costs a lot of money.
 A lot of money, if you otherwise, if the international situation is good, we can improve a little, but no way, the world is like this.
 Ask: ah, big ye, you are what work do? I think you understand the government that way,
Answer: I farm by land, farm by oneself.
 Q: Yeah, what did you don't do when you came here in' 97?

 Answer: After doing a small business, take grandchildren.
 Q: Then you just said that you just said that they are still good. But do you feel that from your own point of view, do you as an old person, do you think they can do a little better? To make you even happier? Is there any advice from here?

 A: That's not so demanding. Ok, Ok, thank you for working hard

Q: How old are you aged now?
 A: 81 years old.
 Q: Are you a local person?
 Answer: My hometown is in Chaoshan. I visited Shenzhen 6 years ago, and now I live in Dongguan. He had three sons and a daughter, and the sons did business, so they moved over with him.
 Q: You look so healthy.
 A: Yes, I always get sick,
Q: But you look very energetic. What are the main diseases?
 A: This heart disease. Heart at first the body is still very good, 69 years old that year car accident ah, originally I was often often have exercise ah, this his foot was broken, just stay at home for a year. I started having a bad heart at the end of my 70s. Well, the heart is not good. I keep taking that heart medicine. Well. Just eat about four or five years, four or five years later began that stomach is bad, gastric ulcer, slowly chronic gastritis erosion. After this heart also this to this foot influence, began the heart is not good. The heart is not good to take medicine for a long time, the stomach is bad.be not allowed. Now you can't even take that cerebrovascular, nor is the arteriosclerosis.
 Ask: oh, this way, that this is really multiple serious diseases, that you these diseases will usually affect your life a lot, is not you usually affect your own life, or mood ah, family relations, old body health can body body is not healthy trouble. Well, then you think you now the whole of your own life, because these diseases will be your life is generally satisfied?
 A: Generally speaking, it is still ok, right?
 Q: that you and the children their family relationship, and neighbor relationship these all very good?
 A: Generally,
Q: Not so much, right? How you have your children. How many children are there?
 A: Three boys and a girl.
 Q: Oh, four children. Are you a local person or a nonlocal person?
 Answer: I chaoshan come over, how long did you come over? I started working out in Shenzhen when I was 50.
 Q: I came here in 1997. I have been here for a long time. There are still more than ten days, more than 20 years away.
 Q: Well. Oh, if you come here, will you come here for your own business, or.
 A: My son did a little business.
 Q: So you came here with your son, so you came along with you?
 A: Well.
 Q: Oh, so, that's like most of the family population is here,
A: Yes, son, what daughter-in-law, grandchildren, and these are all here.
 Q: What do you think is the happiest thing in your life now is what you are most satisfied with. What are you most satisfied with in your whole state of life?
 Answer: satisfaction is this has children to have a family, have grandson. That grandson even graduated from college and came out to work.

 Q: Generally speaking, I am quite satisfied with my own feelings and my life. Still satisfied? Or not how satisfied?
 A: Satisfied.
 Q: Do you think you are living with your children or not right now?
 A: Live together.
 Q: That means that you think you live now and you say you live with your children, right?
 A: Yes.
 Q: If you live together, do you think it is quite different to live together with the young people? Or do you prefer to live alone or prefer to live with your children to answer: ah. No, no, then you are all the young people are very filial piety.

 Q: Oh, that's good, because many young people are not used to living with the old people. Generally speaking, you feel very comfortable living with them, don't they?
 Answer: That is still very filial piety.
 Q: Are you usually sick, they will accompany you to see a doctor. Will come with you, won't you? For example, what you usually take medicine, they will help you to pay attention to ah or how
A: Yes, yes.
 Q: Ok, that means you think you mean you are generally satisfied, but do you really think about it? Well, from the community, or the government, or from the community or other aspects, you can make your life a little better and happier. Do you have any good advice? What can we do? From the community, ah, or from this medical staff, ah, or from the government, ah, what do you think we can do better. It can improve your satisfaction, improve your satisfaction, improve your life happiness, then you are happier.

 Answer: that generally basically in that community now, that service also generally still can ah.
 Q: Generally speaking, do you think this kind of service in their community is ok in all aspects. Does the government have any care or special care for your elderly people?
 A: Yes, yes, yes, yes,
Q: What are the benefits.

 A: For example, it was in the countryside, ah ah, I live at the age of MAO Zedong era, it was very hard at that time. Yes, yes, that substance is very good, and you will get bored. Yes, that's different now. Since I have been in the countryside since I was 65 years ago, then the government has taken care of the old man. At first, it is 50 yuan, 50 yuan a month after 55 yuan, slowly lift, slowly lift, until now there are more than 200 points.
 Q: Every month, right?
 A: Yes
Q: every month you although you this number in the outside this situation is that proportion is very small, right? But you have so much money so much old people calculate that amount is very powerful. Then you quite understand the government.
 A: Well, yes.
 Q: Do they give you, for example, the usual care is if the community pays you if the government gives you some other benefits. Ask you how well you are. That means to care about you or to check for you. You're not having anything else.

 Answer: it is a family like me, if I also had to apply at that time, is my daughter my son-in-law she is also a civil servant.

 She works in the internal department. Well, if I were ordinary and I wouldn't want that much, I would apply to the disabled government, but I was just getting right, I didn't do that. Well, even a country is not very easy either. You see, the international situation is not good, and this country costs a lot of money.
 A lot of money, if you otherwise, if the international situation is good, we can improve a little, but no way, the world is like this.
 Ask: ah, big ye, you are what work do? I think you understand the government that way,
Answer: I farm by land, farm by oneself.
 Q: Yeah, what did you don't do when you came here in' 97?

 Answer: After doing a small business, take grandchildren.
 Q: Then you just said that you just said that they are still good. But do you feel that from your own point of view, do you as an old person, do you think they can do a little better? To make you even happier? Is there any advice from here?

 A: That's not so demanding. Ok, Ok, thank you for working hard

Q: How old are you aged now?
 A: 82 years old.
 Q: Are you a local person?
 Answer: My hometown is in Chaoshan. I visited Shenzhen 7 years ago, and now I live in Dongguan. He had three sons and a daughter, and the sons did business, so they moved over with him.
 Q: You look so healthy.
 A: Yes, I always get sick,
Q: But you look very energetic. What are the main diseases?
 A: This heart disease. Heart at first the body is still very good, 70 years old that year car accident, originally I was often often have exercise, this his foot broke, just stay at home for a year. I started having a bad heart at the end of age 71. Well, the heart is not good. I keep taking that heart medicine. Well. Just eat about four or five years, four or five years later began that stomach is bad, gastric ulcer, slowly chronic gastritis erosion. After this heart also this to this foot influence, began the heart is not good. The heart is not good to take medicine for a long time, the stomach is bad.be not allowed. Now you can't even take that cerebrovascular, nor is the arteriosclerosis.
 Ask: oh, this way, that this is really multiple serious diseases, that you these diseases will usually affect your life a lot, is not you usually affect your own life, or mood ah, family relations, old body health can body body is not healthy trouble. Well, then you think you now the whole of your own life, because these diseases will be your life is generally satisfied?
 A: Generally speaking, it is still ok, right?
 Q: that you and the children their family relationship, and neighbor relationship these all very good?
 A: Generally,
Q: Not so much, right? How you have your children. How many children are there?
 A: Three boys and a girl.
 Q: Oh, four children. Are you a local person or a nonlocal person?
 Answer: I chaoshan come over, how long did you come over? I started working out in Shenzhen when I was 50.
 Q: I came here in 1997. I have been here for a long time. There are still more than ten days, more than 20 years away.
 Q: Well. Oh, if you come here, will you come here for your own business, or.
 A: My son did a little business.
 Q: So you came here with your son, so you came along with you?
 A: Well.
 Q: Oh, so, that's like most of the family population is here,
A: Yes, son, what daughter-in-law, grandchildren, and these are all here.
 Q: What do you think is the happiest thing in your life now is what you are most satisfied with. What are you most satisfied with in your whole state of life?
 Answer: satisfaction is this has children to have a family, have grandson. That grandson even graduated from college and came out to work.

 Q: Generally speaking, I am quite satisfied with my own feelings and my life. Still satisfied? Or not how satisfied?
 A: Satisfied.
 Q: Do you think you are living with your children or not right now?
 A: Live together.
 Q: That means that you think you live now and you say you live with your children, right?
 A: Yes.
 Q: If you live together, do you think it is quite different to live together with the young people? Or do you prefer to live alone or prefer to live with your children to answer: ah. No, no, then you are all the young people are very filial piety.

 Q: Oh, that's good, because many young people are not used to living with the old people. Generally speaking, you feel very comfortable living with them, don't they?
 Answer: That is still very filial piety.
 Q: Are you usually sick, they will accompany you to see a doctor. Will come with you, won't you? For example, what you usually take medicine, they will help you to pay attention to ah or how
A: Yes, yes.
 Q: Ok, that means you think you mean you are generally satisfied, but do you really think about it? Well, from the community, or the government, or from the community or other aspects, you can make your life a little better and happier. Do you have any good advice? What can we do? From the community, ah, or from this medical staff, ah, or from the government, ah, what do you think we can do better. It can improve your satisfaction, improve your satisfaction, improve your life happiness, then you are happier.

 Answer: that generally basically in that community now, that service also generally still can ah.
 Q: Generally speaking, do you think this kind of service in their community is ok in all aspects. Does the government have any care or special care for your elderly people?
 A: Yes, yes, yes, yes,
Q: What are the benefits.

 A: For example, it was in the countryside, ah ah, I live at the age of MAO Zedong era, it was very hard at that time. Yes, yes, that substance is very good, and you will get bored. Yes, that's different now. Since I have been in the countryside since I was 65 years ago, then the government has taken care of the old man. At first, it is 50 yuan, 50 yuan a month after 55 yuan, slowly lift, slowly lift, until now there are more than 200 points.
 Q: Every month, right?
 A: Yes
Q: every month you although you this number in the outside this situation is that proportion is very small, right? But you have so much money so much old people calculate that amount is very powerful. Then you quite understand the government.
 A: Well, yes.
 Q: Do they give you, for example, the usual care is if the community pays you if the government gives you some other benefits. Ask you how well you are. That means to care about you or to check for you. You're not having anything else.

 Answer: it is a family like me, if I also had to apply at that time, is my daughter my son-in-law she is also a civil servant.

 She works in the internal department. Well, if I were ordinary and I wouldn't want that much, I would apply to the disabled government, but I was just getting right, I didn't do that. Well, even a country is not very easy either. You see, the international situation is not good, and this country costs a lot of money.
 A lot of money, if you otherwise, if the international situation is good, we can improve a little, but no way, the world is like this.
 Ask: ah, big ye, you are what work do? I think you understand the government that way,
Answer: I farm by land, farm by oneself.
 Q: Yeah, what did you don't do when you came here in' 97?

 Answer: After doing a small business, take grandchildren.
 Q: Then you just said that you just said that they are still good. But do you feel that from your own point of view, do you as an old person, do you think they can do a little better? To make you even happier? Is there any advice from here?

 A: That's not so demanding. Ok, Ok, thank you for working hard

Q: How old are you aged now?
 A: 83 years old.
 Q: Are you a local person?
 A: My hometown is in Chaoshan. I visited Shenzhen 8 years ago, and now I live in Dongguan. He had three sons and a daughter, and the sons did business, so they moved over with him.
 Q: You look so healthy.
 A: Yes, I always get sick,
Q: But you look very energetic. What are the main diseases?
 A: This heart disease. Heart at first the body is still very good, 71 years old that year car accident ah, originally I was often often have exercise ah, this his foot broke, just stay at home for a year. He started having a bad heart at the end of age 72. Well, the heart is not good. I keep taking that heart medicine. Well. Just eat about four or five years, four or five years later began that stomach is bad, gastric ulcer, slowly chronic gastritis erosion. After this heart also this to this foot influence, began the heart is not good. The heart is not good to take medicine for a long time, the stomach is bad.be not allowed. Now you can't even take that cerebrovascular, nor is the arteriosclerosis.
 Ask: oh, this way, that this is really multiple serious diseases, that you these diseases will usually affect your life a lot, is not you usually affect your own life, or mood ah, family relations, old body health can body body is not healthy trouble. Well, then you think you now the whole of your own life, because these diseases will be your life is generally satisfied?
 A: Generally speaking, it is still ok, right?
 Q: that you and the children their family relationship, and neighbor relationship these all very good?
 A: Generally,
Q: Not so much, right? How you have your children. How many children are there?
 A: Three boys and a girl.
 Q: Oh, four children. Are you a local person or a nonlocal person?
 Answer: I chaoshan come over, how long did you come over? I started working out in Shenzhen when I was 50.
 Q: I came here in 1997. I have been here for a long time. There are still more than ten days, more than 20 years away.
 Q: Well. Oh, if you come here, will you come here for your own business, or.
 A: My son did a little business.
 Q: So you came here with your son, so you came along with you?
 A: Well.
 Q: Oh, so, that's like most of the family population is here,
A: Yes, son, what daughter-in-law, grandchildren, and these are all here.
 Q: What do you think is the happiest thing in your life now is what you are most satisfied with. What are you most satisfied with in your whole state of life?
 Answer: satisfaction is this has children to have a family, have grandson. That grandson even graduated from college and came out to work.

 Q: Generally speaking, I am quite satisfied with my own feelings and my life. Still satisfied? Or not how satisfied?
 A: Satisfied.
 Q: Do you think you are living with your children or not right now?
 A: Live together.
 Q: That means that you think you live now and you say you live with your children, right?
 A: Yes.
 Q: If you live together, do you think it is quite different to live together with the young people? Or do you prefer to live alone or prefer to live with your children to answer: ah. No, no, then you are all the young people are very filial piety.

 Q: Oh, that's good, because many young people are not used to living with the old people. Generally speaking, you feel very comfortable living with them, don't they?
 Answer: That is still very filial piety.
 Q: Are you usually sick, they will accompany you to see a doctor. Will come with you, won't you? For example, what you usually take medicine, they will help you to pay attention to ah or how
A: Yes, yes.
 Q: Ok, that means you think you mean you are generally satisfied, but do you really think about it? Well, from the community, or the government, or from the community or other aspects, you can make your life a little better and happier. Do you have any good advice? What can we do? From the community, ah, or from this medical staff, ah, or from the government, ah, what do you think we can do better. It can improve your satisfaction, improve your satisfaction, improve your life happiness, then you are happier.

 Answer: that generally basically in that community now, that service also generally still can ah.
 Q: Generally speaking, do you think this kind of service in their community is ok in all aspects. Does the government have any care or special care for your elderly people?
 A: Yes, yes, yes, yes,
Q: What are the benefits.

 A: For example, it was in the countryside, ah ah, I live at the age of MAO Zedong era, it was very hard at that time. Yes, yes, that substance is very good, and you will get bored. Yes, that's different now. Since I have been in the countryside since I was 65 years ago, then the government has taken care of the old man. At first, it is 50 yuan, 50 yuan a month after 55 yuan, slowly lift, slowly lift, until now there are more than 200 points.
 Q: Every month, right?
 A: Yes
Q: every month you although you this number in the outside this situation is that proportion is very small, right? But you have so much money so much old people calculate that amount is very powerful. Then you quite understand the government.
 A: Well, yes.
 Q: Do they give you, for example, the usual care is if the community pays you if the government gives you some other benefits. Ask you how well you are. That means to care about you or to check for you. You're not having anything else.

 Answer: it is a family like me, if I also had to apply at that time, is my daughter my son-in-law she is also a civil servant.

 She works in the internal department. Well, if I were ordinary and I wouldn't want that much, I would apply to the disabled government, but I was just getting right, I didn't do that. Well, even a country is not very easy either. You see, the international situation is not good, and this country costs a lot of money.
 A lot of money, if you otherwise, if the international situation is good, we can improve a little, but no way, the world is like this.
 Ask: ah, big ye, you are what work do? I think you understand the government that way,
Answer: I farm by land, farm by oneself.
 Q: Yeah, what did you don't do when you came here in' 97?

 Answer: After doing a small business, take grandchildren.
 Q: Then you just said that you just said that they are still good. But do you feel that from your own point of view, do you as an old person, do you think they can do a little better? To make you even happier? Is there any advice from here?

 A: That's not so demanding. Ok, Ok, thank you for working hard

Q: How old are you aged now?
 A: 84 years old.
 Q: Are you a local person?
 A: My hometown is in Chaoshan. I visited Shenzhen 9 years ago, and now I live in Dongguan. He had three sons and a daughter, and the sons did business, so they moved over with him.
 Q: You look so healthy.
 A: Yes, I always get sick,
Q: But you look very energetic. What are the main diseases?
 A: This heart disease. Heart at first the body is very good, 72 years old that year car accident, originally I was often often have exercise, this his foot broken, just stay at home for a year. He started having a bad heart at the end of age 73. Well, the heart is not good. I keep taking that heart medicine. Well. Just eat about four or five years, four or five years later began that stomach is bad, gastric ulcer, slowly chronic gastritis erosion. After this heart also this to this foot influence, began the heart is not good. The heart is not good to take medicine for a long time, the stomach is bad.be not allowed. Now you can't even take that cerebrovascular, nor is the arteriosclerosis.
 Ask: oh, this way, that this is really multiple serious diseases, that you these diseases will usually affect your life a lot, is not you usually affect your own life, or mood ah, family relations, old body health can body body is not healthy trouble. Well, then you think you now the whole of your own life, because these diseases will be your life is generally satisfied?
 A: Generally speaking, it is still ok, right?
 Q: that you and the children their family relationship, and neighbor relationship these all very good?
 A: Generally,
Q: Not so much, right? How you have your children. How many children are there?
 A: Three boys and a girl.
 Q: Oh, four children. Are you a local person or a nonlocal person?
 Answer: I chaoshan come over, how long did you come over? I started working out in Shenzhen when I was 50.
 Q: I came here in 1997. I have been here for a long time. There are still more than ten days, more than 20 years away.
 Q: Well. Oh, if you come here, will you come here for your own business, or.
 A: My son did a little business.
 Q: So you came here with your son, so you came along with you?
 A: Well.
 Q: Oh, so, that's like most of the family population is here,
A: Yes, son, what daughter-in-law, grandchildren, and these are all here.
 Q: What do you think is the happiest thing in your life now is what you are most satisfied with. What are you most satisfied with in your whole state of life?
 Answer: satisfaction is this has children to have a family, have grandson. That grandson even graduated from college and came out to work.

 Q: Generally speaking, I am quite satisfied with my own feelings and my life. Still satisfied? Or not how satisfied?
 A: Satisfied.
 Q: Do you think you are living with your children or not right now?
 A: Live together.
 Q: That means that you think you live now and you say you live with your children, right?
 A: Yes.
 Q: If you live together, do you think it is quite different to live together with the young people? Or do you prefer to live alone or prefer to live with your children to answer: ah. No, no, then you are all the young people are very filial piety.

 Q: Oh, that's good, because many young people are not used to living with the old people. Generally speaking, you feel very comfortable living with them, don't they?
 Answer: That is still very filial piety.
 Q: Are you usually sick, they will accompany you to see a doctor. Will come with you, won't you? For example, what you usually take medicine, they will help you to pay attention to ah or how
A: Yes, yes.
 Q: Ok, that means you think you mean you are generally satisfied, but do you really think about it? Well, from the community, or the government, or from the community or other aspects, you can make your life a little better and happier. Do you have any good advice? What can we do? From the community, ah, or from this medical staff, ah, or from the government, ah, what do you think we can do better. It can improve your satisfaction, improve your satisfaction, improve your life happiness, then you are happier.

 Answer: that generally basically in that community now, that service also generally still can ah.
 Q: Generally speaking, do you think this kind of service in their community is ok in all aspects. Does the government have any care or special care for your elderly people?
 A: Yes, yes, yes, yes,
Q: What are the benefits.

 A: For example, it was in the countryside, ah ah, I live at the age of MAO Zedong era, it was very hard at that time. Yes, yes, that substance is very good, and you will get bored. Yes, that's different now. Since I have been in the countryside since I was 65 years ago, then the government has taken care of the old man. At first, it is 50 yuan, 50 yuan a month after 55 yuan, slowly lift, slowly lift, until now there are more than 200 points.
 Q: Every month, right?
 A: Yes
Q: every month you although you this number in the outside this situation is that proportion is very small, right? But you have so much money so much old people calculate that amount is very powerful. Then you quite understand the government.
 A: Well, yes.
 Q: Do they give you, for example, the usual care is if the community pays you if the government gives you some other benefits. Ask you how well you are. That means to care about you or to check for you. You're not having anything else.

 Answer: it is a family like me, if I also had to apply at that time, is my daughter my son-in-law she is also a civil servant.

 She works in the internal department. Well, if I were ordinary and I wouldn't want that much, I would apply to the disabled government, but I was just getting right, I didn't do that. Well, even a country is not very easy either. You see, the international situation is not good, and this country costs a lot of money.
 A lot of money, if you otherwise, if the international situation is good, we can improve a little, but no way, the world is like this.
 Ask: ah, big ye, you are what work do? I think you understand the government that way,
Answer: I farm by land, farm by oneself.
 Q: Yeah, what did you don't do when you came here in' 97?

 Answer: After doing a small business, take grandchildren.
 Q: Then you just said that you just said that they are still good. But do you feel that from your own point of view, do you as an old person, do you think they can do a little better? To make you even happier? Is there any advice from here?

 A: That's not so demanding. Ok, Ok, thank you for working hard

Q: How old are you aged now?
 A: 85 years old.
 Q: Are you a local person?
 A: My hometown is in Chaoshan. I came to Shenzhen 10 years ago, and now I live in Dongguan. He had three sons and a daughter, and the sons did business, so they moved over with him.
 Q: You look so healthy.
 A: Yes, I always get sick,
Q: But you look very energetic. What are the main diseases?
 A: This heart disease. Heart at first the body is still very good, 73 years old that year car accident, originally I was often often have exercise, this his foot was broken, just stay at home for a year. He started having a bad heart at the end of age 74. Well, the heart is not good. I keep taking that heart medicine. Well. Just eat about four or five years, four or five years later began that stomach is bad, gastric ulcer, slowly chronic gastritis erosion. After this heart also this to this foot influence, began the heart is not good. The heart is not good to take medicine for a long time, the stomach is bad.be not allowed. Now you can't even take that cerebrovascular, nor is the arteriosclerosis.
 Ask: oh, this way, that this is really multiple serious diseases, that you these diseases will usually affect your life a lot, is not you usually affect your own life, or mood ah, family relations, old body health can body body is not healthy trouble. Well, then you think you now the whole of your own life, because these diseases will be your life is generally satisfied?
 A: Generally speaking, it is still ok, right?
 Q: that you and the children their family relationship, and neighbor relationship these all very good?
 A: Generally,
Q: Not so much, right? How you have your children. How many children are there?
 A: Three boys and a girl.
 Q: Oh, four children. Are you a local person or a nonlocal person?
 Answer: I chaoshan come over, how long did you come over? I started working out in Shenzhen when I was 50.
 Q: I came here in 1997. I have been here for a long time. There are still more than ten days, more than 20 years away.
 Q: Well. Oh, if you come here, will you come here for your own business, or.
 A: My son did a little business.
 Q: So you came here with your son, so you came along with you?
 A: Well.
 Q: Oh, so, that's like most of the family population is here,
A: Yes, son, what daughter-in-law, grandchildren, and these are all here.
 Q: What do you think is the happiest thing in your life now is what you are most satisfied with. What are you most satisfied with in your whole state of life?
 Answer: satisfaction is this has children to have a family, have grandson. That grandson even graduated from college and came out to work.

 Q: Generally speaking, I am quite satisfied with my own feelings and my life. Still satisfied? Or not how satisfied?
 A: Satisfied.
 Q: Do you think you are living with your children or not right now?
 A: Live together.
 Q: That means that you think you live now and you say you live with your children, right?
 A: Yes.
 Q: If you live together, do you think it is quite different to live together with the young people? Or do you prefer to live alone or prefer to live with your children to answer: ah. No, no, then you are all the young people are very filial piety.

 Q: Oh, that's good, because many young people are not used to living with the old people. Generally speaking, you feel very comfortable living with them, don't they?
 Answer: That is still very filial piety.
 Q: Are you usually sick, they will accompany you to see a doctor. Will come with you, won't you? For example, what you usually take medicine, they will help you to pay attention to ah or how
A: Yes, yes.
 Q: Ok, that means you think you mean you are generally satisfied, but do you really think about it? Well, from the community, or the government, or from the community or other aspects, you can make your life a little better and happier. Do you have any good advice? What can we do? From the community, ah, or from this medical staff, ah, or from the government, ah, what do you think we can do better. It can improve your satisfaction, improve your satisfaction, improve your life happiness, then you are happier.

 Answer: that generally basically in that community now, that service also generally still can ah.
 Q: Generally speaking, do you think this kind of service in their community is ok in all aspects. Does the government have any care or special care for your elderly people?
 A: Yes, yes, yes, yes,
Q: What are the benefits.

 A: For example, it was in the countryside, ah ah, I live at the age of MAO Zedong era, it was very hard at that time. Yes, yes, that substance is very good, and you will get bored. Yes, that's different now. Since I have been in the countryside since I was 65 years ago, then the government has taken care of the old man. At first, it is 50 yuan, 50 yuan a month after 55 yuan, slowly lift, slowly lift, until now there are more than 200 points.
 Q: Every month, right?
 A: Yes
Q: every month you although you this number in the outside this situation is that proportion is very small, right? But you have so much money so much old people calculate that amount is very powerful. Then you quite understand the government.
 A: Well, yes.
 Q: Do they give you, for example, the usual care is if the community pays you if the government gives you some other benefits. Ask you how well you are. That means to care about you or to check for you. You're not having anything else.

 Answer: it is a family like me, if I also had to apply at that time, is my daughter my son-in-law she is also a civil servant.

 She works in the internal department. Well, if I were ordinary and I wouldn't want that much, I would apply to the disabled government, but I was just getting right, I didn't do that. Well, even a country is not very easy either. You see, the international situation is not good, and this country costs a lot of money.
 A lot of money, if you otherwise, if the international situation is good, we can improve a little, but no way, the world is like this.
 Ask: ah, big ye, you are what work do? I think you understand the government that way,
Answer: I farm by land, farm by oneself.
 Q: Yeah, what did you don't do when you came here in' 97?

 Answer: After doing a small business, take grandchildren.
 Q: Then you just said that you just said that they are still good. But do you feel that from your own point of view, do you as an old person, do you think they can do a little better? To make you even happier? Is there any advice from here?

 A: That's not so demanding. Ok, Ok, thank you for working hard

Q: How old are you aged now?
 A: 86 years old.
 Q: Are you a local person?
 Answer: My hometown is in Chaoshan. I visited Shenzhen 6 years ago, and now I live in Dongguan. He had three sons and a daughter, and the sons did business, so they moved over with him.
 Q: You look so healthy.
 A: Yes, I always get sick,
Q: But you look very energetic. What are the main diseases?
 A: This heart disease. Heart at first the body is still very good, 69 years old that year car accident ah, originally I was often often have exercise ah, this his foot was broken, just stay at home for a year. I started having a bad heart at the end of my 70s. Well, the heart is not good. I keep taking that heart medicine. Well. Just eat about four or five years, four or five years later began that stomach is bad, gastric ulcer, slowly chronic gastritis erosion. After this heart also this to this foot influence, began the heart is not good. The heart is not good to take medicine for a long time, the stomach is bad.be not allowed. Now you can't even take that cerebrovascular, nor is the arteriosclerosis.
 Ask: oh, this way, that this is really multiple serious diseases, that you these diseases will usually affect your life a lot, is not you usually affect your own life, or mood ah, family relations, old body health can body body is not healthy trouble. Well, then you think you now the whole of your own life, because these diseases will be your life is generally satisfied?
 A: Generally speaking, it is still ok, right?
 Q: that you and the children their family relationship, and neighbor relationship these all very good?
 A: Generally,
Q: Not so much, right? How you have your children. How many children are there?
 A: Three boys and a girl.
 Q: Oh, four children. Are you a local person or a nonlocal person?
 Answer: I chaoshan come over, how long did you come over? I started working out in Shenzhen when I was 50.
 Q: I came here in 1997. I have been here for a long time. There are still more than ten days, more than 20 years away.
 Q: Well. Oh, if you come here, will you come here for your own business, or.
 A: My son did a little business.
 Q: So you came here with your son, so you came along with you?
 A: Well.
 Q: Oh, so, that's like most of the family population is here,
A: Yes, son, what daughter-in-law, grandchildren, and these are all here.
 Q: What do you think is the happiest thing in your life now is what you are most satisfied with. What are you most satisfied with in your whole state of life?
 Answer: satisfaction is this has children to have a family, have grandson. That grandson even graduated from college and came out to work.

 Q: Generally speaking, I am quite satisfied with my own feelings and my life. Still satisfied? Or not how satisfied?
 A: Satisfied.
 Q: Do you think you are living with your children or not right now?
 A: Live together.
 Q: That means that you think you live now and you say you live with your children, right?
 A: Yes.
 Q: If you live together, do you think it is quite different to live together with the young people? Or do you prefer to live alone or prefer to live with your children to answer: ah. No, no, then you are all the young people are very filial piety.

 Q: Oh, that's good, because many young people are not used to living with the old people. Generally speaking, you feel very comfortable living with them, don't they?
 Answer: That is still very filial piety.
 Q: Are you usually sick, they will accompany you to see a doctor. Will come with you, won't you? For example, what you usually take medicine, they will help you to pay attention to ah or how
A: Yes, yes.
 Q: Ok, that means you think you mean you are generally satisfied, but do you really think about it? Well, from the community, or the government, or from the community or other aspects, you can make your life a little better and happier. Do you have any good advice? What can we do? From the community, ah, or from this medical staff, ah, or from the government, ah, what do you think we can do better. It can improve your satisfaction, improve your satisfaction, improve your life happiness, then you are happier.

 Answer: that generally basically in that community now, that service also generally still can ah.
 Q: Generally speaking, do you think this kind of service in their community is ok in all aspects. Does the government have any care or special care for your elderly people?
 A: Yes, yes, yes, yes,
Q: What are the benefits.

 A: For example, it was in the countryside, ah ah, I live at the age of MAO Zedong era, it was very hard at that time. Yes, yes, that substance is very good, and you will get bored. Yes, that's different now. Since I have been in the countryside since I was 65 years ago, then the government has taken care of the old man. At first, it is 50 yuan, 50 yuan a month after 55 yuan, slowly lift, slowly lift, until now there are more than 200 points.
 Q: Every month, right?
 A: Yes
Q: every month you although you this number in the outside this situation is that proportion is very small, right? But you have so much money so much old people calculate that amount is very powerful. Then you quite understand the government.
 A: Well, yes.
 Q: Do they give you, for example, the usual care is if the community pays you if the government gives you some other benefits. Ask you how well you are. That means to care about you or to check for you. You're not having anything else.

 Answer: it is a family like me, if I also had to apply at that time, is my daughter my son-in-law she is also a civil servant.

 She works in the internal department. Well, if I were ordinary and I wouldn't want that much, I would apply to the disabled government, but I was just getting right, I didn't do that. Well, even a country is not very easy either. You see, the international situation is not good, and this country costs a lot of money.
 A lot of money, if you otherwise, if the international situation is good, we can improve a little, but no way, the world is like this.
 Ask: ah, big ye, you are what work do? I think you understand the government that way,
Answer: I farm by land, farm by oneself.
 Q: Yeah, what did you don't do when you came here in' 97?

 Answer: After doing a small business, take grandchildren.
 Q: Then you just said that you just said that they are still good. But do you feel that from your own point of view, do you as an old person, do you think they can do a little better? To make you even happier? Is there any advice from here?

 A: That's not so demanding. Ok, Ok, thank you for working hard

Q: How old are you aged now?
 A: 87 years old.
 Q: Are you a local person?
 Answer: My hometown is in Chaoshan. I visited Shenzhen 7 years ago, and now I live in Dongguan. He had three sons and a daughter, and the sons did business, so they moved over with him.
 Q: You look so healthy.
 A: Yes, I always get sick,
Q: But you look very energetic. What are the main diseases?
 A: This heart disease. Heart at first the body is still very good, 70 years old that year car accident, originally I was often often have exercise, this his foot broke, just stay at home for a year. I started having a bad heart at the end of age 71. Well, the heart is not good. I keep taking that heart medicine. Well. Just eat about four or five years, four or five years later began that stomach is bad, gastric ulcer, slowly chronic gastritis erosion. After this heart also this to this foot influence, began the heart is not good. The heart is not good to take medicine for a long time, the stomach is bad.be not allowed. Now you can't even take that cerebrovascular, nor is the arteriosclerosis.
 Ask: oh, this way, that this is really multiple serious diseases, that you these diseases will usually affect your life a lot, is not you usually affect your own life, or mood ah, family relations, old body health can body body is not healthy trouble. Well, then you think you now the whole of your own life, because these diseases will be your life is generally satisfied?
 A: Generally speaking, it is still ok, right?
 Q: that you and the children their family relationship, and neighbor relationship these all very good?
 A: Generally,
Q: Not so much, right? How you have your children. How many children are there?
 A: Three boys and a girl.
 Q: Oh, four children. Are you a local person or a nonlocal person?
 Answer: I chaoshan come over, how long did you come over? I started working out in Shenzhen when I was 50.
 Q: I came here in 1997. I have been here for a long time. There are still more than ten days, more than 20 years away.
 Q: Well. Oh, if you come here, will you come here for your own business, or.
 A: My son did a little business.
 Q: So you came here with your son, so you came along with you?
 A: Well.
 Q: Oh, so, that's like most of the family population is here,
A: Yes, son, what daughter-in-law, grandchildren, and these are all here.
 Q: What do you think is the happiest thing in your life now is what you are most satisfied with. What are you most satisfied with in your whole state of life?
 Answer: satisfaction is this has children to have a family, have grandson. That grandson even graduated from college and came out to work.

 Q: Generally speaking, I am quite satisfied with my own feelings and my life. Still satisfied? Or not how satisfied?
 A: Satisfied.
 Q: Do you think you are living with your children or not right now?
 A: Live together.
 Q: That means that you think you live now and you say you live with your children, right?
 A: Yes.
 Q: If you live together, do you think it is quite different to live together with the young people? Or do you prefer to live alone or prefer to live with your children to answer: ah. No, no, then you are all the young people are very filial piety.

 Q: Oh, that's good, because many young people are not used to living with the old people. Generally speaking, you feel very comfortable living with them, don't they?
 Answer: That is still very filial piety.
 Q: Are you usually sick, they will accompany you to see a doctor. Will come with you, won't you? For example, what you usually take medicine, they will help you to pay attention to ah or how
A: Yes, yes.
 Q: Ok, that means you think you mean you are generally satisfied, but do you really think about it? Well, from the community, or the government, or from the community or other aspects, you can make your life a little better and happier. Do you have any good advice? What can we do? From the community, ah, or from this medical staff, ah, or from the government, ah, what do you think we can do better. It can improve your satisfaction, improve your satisfaction, improve your life happiness, then you are happier.

 Answer: that generally basically in that community now, that service also generally still can ah.
 Q: Generally speaking, do you think this kind of service in their community is ok in all aspects. Does the government have any care or special care for your elderly people?
 A: Yes, yes, yes, yes,
Q: What are the benefits.

 A: For example, it was in the countryside, ah ah, I live at the age of MAO Zedong era, it was very hard at that time. Yes, yes, that substance is very good, and you will get bored. Yes, that's different now. Since I have been in the countryside since I was 65 years ago, then the government has taken care of the old man. At first, it is 50 yuan, 50 yuan a month after 55 yuan, slowly lift, slowly lift, until now there are more than 200 points.
 Q: Every month, right?
 A: Yes
Q: every month you although you this number in the outside this situation is that proportion is very small, right? But you have so much money so much old people calculate that amount is very powerful. Then you quite understand the government.
 A: Well, yes.
 Q: Do they give you, for example, the usual care is if the community pays you if the government gives you some other benefits. Ask you how well you are. That means to care about you or to check for you. You're not having anything else.

 Answer: it is a family like me, if I also had to apply at that time, is my daughter my son-in-law she is also a civil servant.

 She works in the internal department. Well, if I were ordinary and I wouldn't want that much, I would apply to the disabled government, but I was just getting right, I didn't do that. Well, even a country is not very easy either. You see, the international situation is not good, and this country costs a lot of money.
 A lot of money, if you otherwise, if the international situation is good, we can improve a little, but no way, the world is like this.
 Ask: ah, big ye, you are what work do? I think you understand the government that way,
Answer: I farm by land, farm by oneself.
 Q: Yeah, what did you don't do when you came here in' 97?

 Answer: After doing a small business, take grandchildren.
 Q: Then you just said that you just said that they are still good. But do you feel that from your own point of view, do you as an old person, do you think they can do a little better? To make you even happier? Is there any advice from here?

 A: That's not so demanding. Ok, Ok, thank you for working hard

Q: How old are you aged now?
 A: 88 years old.
 Q: Are you a local person?
 A: My hometown is in Chaoshan. I visited Shenzhen 8 years ago, and now I live in Dongguan. He had three sons and a daughter, and the sons did business, so they moved over with him.
 Q: You look so healthy.
 A: Yes, I always get sick,
Q: But you look very energetic. What are the main diseases?
 A: This heart disease. Heart at first the body is still very good, 71 years old that year car accident ah, originally I was often often have exercise ah, this his foot broke, just stay at home for a year. He started having a bad heart at the end of age 72. Well, the heart is not good. I keep taking that heart medicine. Well. Just eat about four or five years, four or five years later began that stomach is bad, gastric ulcer, slowly chronic gastritis erosion. After this heart also this to this foot influence, began the heart is not good. The heart is not good to take medicine for a long time, the stomach is bad.be not allowed. Now you can't even take that cerebrovascular, nor is the arteriosclerosis.
 Ask: oh, this way, that this is really multiple serious diseases, that you these diseases will usually affect your life a lot, is not you usually affect your own life, or mood ah, family relations, old body health can body body is not healthy trouble. Well, then you think you now the whole of your own life, because these diseases will be your life is generally satisfied?
 A: Generally speaking, it is still ok, right?
 Q: that you and the children their family relationship, and neighbor relationship these all very good?
 A: Generally,
Q: Not so much, right? How you have your children. How many children are there?
 A: Three boys and a girl.
 Q: Oh, four children. Are you a local person or a nonlocal person?
 Answer: I chaoshan come over, how long did you come over? I started working out in Shenzhen when I was 50.
 Q: I came here in 1997. I have been here for a long time. There are still more than ten days, more than 20 years away.
 Q: Well. Oh, if you come here, will you come here for your own business, or.
 A: My son did a little business.
 Q: So you came here with your son, so you came along with you?
 A: Well.
 Q: Oh, so, that's like most of the family population is here,
A: Yes, son, what daughter-in-law, grandchildren, and these are all here.
 Q: What do you think is the happiest thing in your life now is what you are most satisfied with. What are you most satisfied with in your whole state of life?
 Answer: satisfaction is this has children to have a family, have grandson. That grandson even graduated from college and came out to work.

 Q: Generally speaking, I am quite satisfied with my own feelings and my life. Still satisfied? Or not how satisfied?
 A: Satisfied.
 Q: Do you think you are living with your children or not right now?
 A: Live together.
 Q: That means that you think you live now and you say you live with your children, right?
 A: Yes.
 Q: If you live together, do you think it is quite different to live together with the young people? Or do you prefer to live alone or prefer to live with your children to answer: ah. No, no, then you are all the young people are very filial piety.

 Q: Oh, that's good, because many young people are not used to living with the old people. Generally speaking, you feel very comfortable living with them, don't they?
 Answer: That is still very filial piety.
 Q: Are you usually sick, they will accompany you to see a doctor. Will come with you, won't you? For example, what you usually take medicine, they will help you to pay attention to ah or how
A: Yes, yes.
 Q: Ok, that means you think you mean you are generally satisfied, but do you really think about it? Well, from the community, or the government, or from the community or other aspects, you can make your life a little better and happier. Do you have any good advice? What can we do? From the community, ah, or from this medical staff, ah, or from the government, ah, what do you think we can do better. It can improve your satisfaction, improve your satisfaction, improve your life happiness, then you are happier.

 Answer: that generally basically in that community now, that service also generally still can ah.
 Q: Generally speaking, do you think this kind of service in their community is ok in all aspects. Does the government have any care or special care for your elderly people?
 A: Yes, yes, yes, yes,
Q: What are the benefits.

 A: For example, it was in the countryside, ah ah, I live at the age of MAO Zedong era, it was very hard at that time. Yes, yes, that substance is very good, and you will get bored. Yes, that's different now. Since I have been in the countryside since I was 65 years ago, then the government has taken care of the old man. At first, it is 50 yuan, 50 yuan a month after 55 yuan, slowly lift, slowly lift, until now there are more than 200 points.
 Q: Every month, right?
 A: Yes
Q: every month you although you this number in the outside this situation is that proportion is very small, right? But you have so much money so much old people calculate that amount is very powerful. Then you quite understand the government.
 A: Well, yes.
 Q: Do they give you, for example, the usual care is if the community pays you if the government gives you some other benefits. Ask you how well you are. That means to care about you or to check for you. You're not having anything else.

 Answer: it is a family like me, if I also had to apply at that time, is my daughter my son-in-law she is also a civil servant.

 She works in the internal department. Well, if I were ordinary and I wouldn't want that much, I would apply to the disabled government, but I was just getting right, I didn't do that. Well, even a country is not very easy either. You see, the international situation is not good, and this country costs a lot of money.
 A lot of money, if you otherwise, if the international situation is good, we can improve a little, but no way, the world is like this.
 Ask: ah, big ye, you are what work do? I think you understand the government that way,
Answer: I farm by land, farm by oneself.
 Q: Yeah, what did you don't do when you came here in' 97?

 Answer: After doing a small business, take grandchildren.
 Q: Then you just said that you just said that they are still good. But do you feel that from your own point of view, do you as an old person, do you think they can do a little better? To make you even happier? Is there any advice from here?

 A: That's not so demanding. Ok, Ok, thank you for working hard

Q: How old are you aged now?
 A: 89 years old.
 Q: Are you a local person?
 A: My hometown is in Chaoshan. I visited Shenzhen 9 years ago, and now I live in Dongguan. He had three sons and a daughter, and the sons did business, so they moved over with him.
 Q: You look so healthy.
 A: Yes, I always get sick,
Q: But you look very energetic. What are the main diseases?
 A: This heart disease. Heart at first the body is very good, 72 years old that year car accident, originally I was often often have exercise, this his foot broken, just stay at home for a year. He started having a bad heart at the end of age 73. Well, the heart is not good. I keep taking that heart medicine. Well. Just eat about four or five years, four or five years later began that stomach is bad, gastric ulcer, slowly chronic gastritis erosion. After this heart also this to this foot influence, began the heart is not good. The heart is not good to take medicine for a long time, the stomach is bad.be not allowed. Now you can't even take that cerebrovascular, nor is the arteriosclerosis.
 Ask: oh, this way, that this is really multiple serious diseases, that you these diseases will usually affect your life a lot, is not you usually affect your own life, or mood ah, family relations, old body health can body body is not healthy trouble. Well, then you think you now the whole of your own life, because these diseases will be your life is generally satisfied?
 A: Generally speaking, it is still ok, right?
 Q: that you and the children their family relationship, and neighbor relationship these all very good?
 A: Generally,
Q: Not so much, right? How you have your children. How many children are there?
 A: Three boys and a girl.
 Q: Oh, four children. Are you a local person or a nonlocal person?
 Answer: I chaoshan come over, how long did you come over? I started working out in Shenzhen when I was 50.
 Q: I came here in 1997. I have been here for a long time. There are still more than ten days, more than 20 years away.
 Q: Well. Oh, if you come here, will you come here for your own business, or.
 A: My son did a little business.
 Q: So you came here with your son, so you came along with you?
 A: Well.
 Q: Oh, so, that's like most of the family population is here,
A: Yes, son, what daughter-in-law, grandchildren, and these are all here.
 Q: What do you think is the happiest thing in your life now is what you are most satisfied with. What are you most satisfied with in your whole state of life?
 Answer: satisfaction is this has children to have a family, have grandson. That grandson even graduated from college and came out to work.

 Q: Generally speaking, I am quite satisfied with my own feelings and my life. Still satisfied? Or not how satisfied?
 A: Satisfied.
 Q: Do you think you are living with your children or not right now?
 A: Live together.
 Q: That means that you think you live now and you say you live with your children, right?
 A: Yes.
 Q: If you live together, do you think it is quite different to live together with the young people? Or do you prefer to live alone or prefer to live with your children to answer: ah. No, no, then you are all the young people are very filial piety.

 Q: Oh, that's good, because many young people are not used to living with the old people. Generally speaking, you feel very comfortable living with them, don't they?
 Answer: That is still very filial piety.
 Q: Are you usually sick, they will accompany you to see a doctor. Will come with you, won't you? For example, what you usually take medicine, they will help you to pay attention to ah or how
A: Yes, yes.
 Q: Ok, that means you think you mean you are generally satisfied, but do you really think about it? Well, from the community, or the government, or from the community or other aspects, you can make your life a little better and happier. Do you have any good advice? What can we do? From the community, ah, or from this medical staff, ah, or from the government, ah, what do you think we can do better. It can improve your satisfaction, improve your satisfaction, improve your life happiness, then you are happier.

 Answer: that generally basically in that community now, that service also generally still can ah.
 Q: Generally speaking, do you think this kind of service in their community is ok in all aspects. Does the government have any care or special care for your elderly people?
 A: Yes, yes, yes, yes,
Q: What are the benefits.

 A: For example, it was in the countryside, ah ah, I live at the age of MAO Zedong era, it was very hard at that time. Yes, yes, that substance is very good, and you will get bored. Yes, that's different now. Since I have been in the countryside since I was 65 years ago, then the government has taken care of the old man. At first, it is 50 yuan, 50 yuan a month after 55 yuan, slowly lift, slowly lift, until now there are more than 200 points.
 Q: Every month, right?
 A: Yes
Q: every month you although you this number in the outside this situation is that proportion is very small, right? But you have so much money so much old people calculate that amount is very powerful. Then you quite understand the government.
 A: Well, yes.
 Q: Do they give you, for example, the usual care is if the community pays you if the government gives you some other benefits. Ask you how well you are. That means to care about you or to check for you. You're not having anything else.

 Answer: it is a family like me, if I also had to apply at that time, is my daughter my son-in-law she is also a civil servant.

 She works in the internal department. Well, if I were ordinary and I wouldn't want that much, I would apply to the disabled government, but I was just getting right, I didn't do that. Well, even a country is not very easy either. You see, the international situation is not good, and this country costs a lot of money.
 A lot of money, if you otherwise, if the international situation is good, we can improve a little, but no way, the world is like this.
 Ask: ah, big ye, you are what work do? I think you understand the government that way,
Answer: I farm by land, farm by oneself.
 Q: Yeah, what did you don't do when you came here in' 97?

 Answer: After doing a small business, take grandchildren.
 Q: Then you just said that you just said that they are still good. But do you feel that from your own point of view, do you as an old person, do you think they can do a little better? To make you even happier? Is there any advice from here?

 A: That's not so demanding. Ok, Ok, thank you for working hard

Q: How old are you aged now?
 A: 90 years old.
 Q: Are you a local person?
 A: My hometown is in Chaoshan. I came to Shenzhen 10 years ago, and now I live in Dongguan. He had three sons and a daughter, and the sons did business, so they moved over with him.
 Q: You look so healthy.
 A: Yes, I always get sick,
Q: But you look very energetic. What are the main diseases?
 A: This heart disease. Heart at first the body is still very good, 73 years old that year car accident, originally I was often often have exercise, this his foot was broken, just stay at home for a year. He started having a bad heart at the end of age 74. Well, the heart is not good. I keep taking that heart medicine. Well. Just eat about four or five years, four or five years later began that stomach is bad, gastric ulcer, slowly chronic gastritis erosion. After this heart also this to this foot influence, began the heart is not good. The heart is not good to take medicine for a long time, the stomach is bad.be not allowed. Now you can't even take that cerebrovascular, nor is the arteriosclerosis.
 Ask: oh, this way, that this is really multiple serious diseases, that you these diseases will usually affect your life a lot, is not you usually affect your own life, or mood ah, family relations, old body health can body body is not healthy trouble. Well, then you think you now the whole of your own life, because these diseases will be your life is generally satisfied?
 A: Generally speaking, it is still ok, right?
 Q: that you and the children their family relationship, and neighbor relationship these all very good?
 A: Generally,
Q: Not so much, right? How you have your children. How many children are there?
 A: Three boys and a girl.
 Q: Oh, four children. Are you a local person or a nonlocal person?
 Answer: I chaoshan come over, how long did you come over? I started working out in Shenzhen when I was 50.
 Q: I came here in 1997. I have been here for a long time. There are still more than ten days, more than 20 years away.
 Q: Well. Oh, if you come here, will you come here for your own business, or.
 A: My son did a little business.
 Q: So you came here with your son, so you came along with you?
 A: Well.
 Q: Oh, so, that's like most of the family population is here,
A: Yes, son, what daughter-in-law, grandchildren, and these are all here.
 Q: What do you think is the happiest thing in your life now is what you are most satisfied with. What are you most satisfied with in your whole state of life?
 Answer: satisfaction is this has children to have a family, have grandson. That grandson even graduated from college and came out to work.

 Q: Generally speaking, I am quite satisfied with my own feelings and my life. Still satisfied? Or not how satisfied?
 A: Satisfied.
 Q: Do you think you are living with your children or not right now?
 A: Live together.
 Q: That means that you think you live now and you say you live with your children, right?
 A: Yes.
 Q: If you live together, do you think it is quite different to live together with the young people? Or do you prefer to live alone or prefer to live with your children to answer: ah. No, no, then you are all the young people are very filial piety.

 Q: Oh, that's good, because many young people are not used to living with the old people. Generally speaking, you feel very comfortable living with them, don't they?
 Answer: That is still very filial piety.
 Q: Are you usually sick, they will accompany you to see a doctor. Will come with you, won't you? For example, what you usually take medicine, they will help you to pay attention to ah or how
A: Yes, yes.
 Q: Ok, that means you think you mean you are generally satisfied, but do you really think about it? Well, from the community, or the government, or from the community or other aspects, you can make your life a little better and happier. Do you have any good advice? What can we do? From the community, ah, or from this medical staff, ah, or from the government, ah, what do you think we can do better. It can improve your satisfaction, improve your satisfaction, improve your life happiness, then you are happier.

 Answer: that generally basically in that community now, that service also generally still can ah.
 Q: Generally speaking, do you think this kind of service in their community is ok in all aspects. Does the government have any care or special care for your elderly people?
 A: Yes, yes, yes, yes,
Q: What are the benefits.

 A: For example, it was in the countryside, ah ah, I live at the age of MAO Zedong era, it was very hard at that time. Yes, yes, that substance is very good, and you will get bored. Yes, that's different now. Since I have been in the countryside since I was 65 years ago, then the government has taken care of the old man. At first, it is 50 yuan, 50 yuan a month after 55 yuan, slowly lift, slowly lift, until now there are more than 200 points.
 Q: Every month, right?
 A: Yes
Q: every month you although you this number in the outside this situation is that proportion is very small, right? But you have so much money so much old people calculate that amount is very powerful. Then you quite understand the government.
 A: Well, yes.
 Q: Do they give you, for example, the usual care is if the community pays you if the government gives you some other benefits. Ask you how well you are. That means to care about you or to check for you. You're not having anything else.

 Answer: it is a family like me, if I also had to apply at that time, is my daughter my son-in-law she is also a civil servant.

 She works in the internal department. Well, if I were ordinary and I wouldn't want that much, I would apply to the disabled government, but I was just getting right, I didn't do that. Well, even a country is not very easy either. You see, the international situation is not good, and this country costs a lot of money.
 A lot of money, if you otherwise, if the international situation is good, we can improve a little, but no way, the world is like this.
 Ask: ah, big ye, you are what work do? I think you understand the government that way,
Answer: I farm by land, farm by oneself.
 Q: Yeah, what did you don't do when you came here in' 97?

 Answer: After doing a small business, take grandchildren.
 Q: Then you just said that you just said that they are still good. But do you feel that from your own point of view, do you as an old person, do you think they can do a little better? To make you even happier? Is there any advice from here?

 A: That's not so demanding. Ok, Ok, thank you for working hard

Q: How old are you aged now?
 A: 81 years old.
 Q: Are you a local person?
 Answer: My hometown is in Chaoshan. I visited Shenzhen 6 years ago, and now I live in Dongguan. He had three sons and a daughter, and the sons did business, so they moved over with him.
 Q: You look so healthy.
 A: Yes, I always get sick,
Q: But you look very energetic. What are the main diseases?
 A: This heart disease. Heart at first the body is still very good, 69 years old that year car accident ah, originally I was often often have exercise ah, this his foot was broken, just stay at home for a year. I started having a bad heart at the end of my 70s. Well, the heart is not good. I keep taking that heart medicine. Well. Just eat about four or five years, four or five years later began that stomach is bad, gastric ulcer, slowly chronic gastritis erosion. After this heart also this to this foot influence, began the heart is not good. The heart is not good to take medicine for a long time, the stomach is bad.be not allowed. Now you can't even take that cerebrovascular, nor is the arteriosclerosis.
 Ask: oh, this way, that this is really multiple serious diseases, that you these diseases will usually affect your life a lot, is not you usually affect your own life, or mood ah, family relations, old body health can body body is not healthy trouble. Well, then you think you now the whole of your own life, because these diseases will be your life is generally satisfied?
 A: Generally speaking, it is still ok, right?
 Q: that you and the children their family relationship, and neighbor relationship these all very good?
 A: Generally,
Q: Not so much, right? How you have your children. How many children are there?
 A: Three boys and a girl.
 Q: Oh, four children. Are you a local person or a nonlocal person?
 Answer: I chaoshan come over, how long did you come over? I started working out in Shenzhen when I was 50.
 Q: I came here in 1997. I have been here for a long time. There are still more than ten days, more than 20 years away.
 Q: Well. Oh, if you come here, will you come here for your own business, or.
 A: My son did a little business.
 Q: So you came here with your son, so you came along with you?
 A: Well.
 Q: Oh, so, that's like most of the family population is here,
A: Yes, son, what daughter-in-law, grandchildren, and these are all here.
 Q: What do you think is the happiest thing in your life now is what you are most satisfied with. What are you most satisfied with in your whole state of life?
 Answer: satisfaction is this has children to have a family, have grandson. That grandson even graduated from college and came out to work.

 Q: Generally speaking, I am quite satisfied with my own feelings and my life. Still satisfied? Or not how satisfied?
 A: Satisfied.
 Q: Do you think you are living with your children or not right now?
 A: Live together.
 Q: That means that you think you live now and you say you live with your children, right?
 A: Yes.
 Q: If you live together, do you think it is quite different to live together with the young people? Or do you prefer to live alone or prefer to live with your children to answer: ah. No, no, then you are all the young people are very filial piety.

 Q: Oh, that's good, because many young people are not used to living with the old people. Generally speaking, you feel very comfortable living with them, don't they?
 Answer: That is still very filial piety.
 Q: Are you usually sick, they will accompany you to see a doctor. Will come with you, won't you? For example, what you usually take medicine, they will help you to pay attention to ah or how
A: Yes, yes.
 Q: Ok, that means you think you mean you are generally satisfied, but do you really think about it? Well, from the community, or the government, or from the community or other aspects, you can make your life a little better and happier. Do you have any good advice? What can we do? From the community, ah, or from this medical staff, ah, or from the government, ah, what do you think we can do better. It can improve your satisfaction, improve your satisfaction, improve your life happiness, then you are happier.

 Answer: that generally basically in that community now, that service also generally still can ah.
 Q: Generally speaking, do you think this kind of service in their community is ok in all aspects. Does the government have any care or special care for your elderly people?
 A: Yes, yes, yes, yes,
Q: What are the benefits.

 A: For example, it was in the countryside, ah ah, I live at the age of MAO Zedong era, it was very hard at that time. Yes, yes, that substance is very good, and you will get bored. Yes, that's different now. Since I have been in the countryside since I was 65 years ago, then the government has taken care of the old man. At first, it is 50 yuan, 50 yuan a month after 55 yuan, slowly lift, slowly lift, until now there are more than 200 points.
 Q: Every month, right?
 A: Yes
Q: every month you although you this number in the outside this situation is that proportion is very small, right? But you have so much money so much old people calculate that amount is very powerful. Then you quite understand the government.
 A: Well, yes.
 Q: Do they give you, for example, the usual care is if the community pays you if the government gives you some other benefits. Ask you how well you are. That means to care about you or to check for you. You're not having anything else.

 Answer: it is a family like me, if I also had to apply at that time, is my daughter my son-in-law she is also a civil servant.

 She works in the internal department. Well, if I were ordinary and I wouldn't want that much, I would apply to the disabled government, but I was just getting right, I didn't do that. Well, even a country is not very easy either. You see, the international situation is not good, and this country costs a lot of money.
 A lot of money, if you otherwise, if the international situation is good, we can improve a little, but no way, the world is like this.
 Ask: ah, big ye, you are what work do? I think you understand the government that way,
Answer: I farm by land, farm by oneself.
 Q: Yeah, what did you don't do when you came here in' 97?

 Answer: After doing a small business, take grandchildren.
 Q: Then you just said that you just said that they are still good. But do you feel that from your own point of view, do you as an old person, do you think they can do a little better? To make you even happier? Is there any advice from here?

 A: That's not so demanding. Ok, Ok, thank you for working hard

Q: How old are you aged now?
 A: 82 years old.
 Q: Are you a local person?
 Answer: My hometown is in Chaoshan. I visited Shenzhen 7 years ago, and now I live in Dongguan. He had three sons and a daughter, and the sons did business, so they moved over with him.
 Q: You look so healthy.
 A: Yes, I always get sick,
Q: But you look very energetic. What are the main diseases?
 A: This heart disease. Heart at first the body is still very good, 70 years old that year car accident, originally I was often often have exercise, this his foot broke, just stay at home for a year. I started having a bad heart at the end of age 71. Well, the heart is not good. I keep taking that heart medicine. Well. Just eat about four or five years, four or five years later began that stomach is bad, gastric ulcer, slowly chronic gastritis erosion. After this heart also this to this foot influence, began the heart is not good. The heart is not good to take medicine for a long time, the stomach is bad.be not allowed. Now you can't even take that cerebrovascular, nor is the arteriosclerosis.
 Ask: oh, this way, that this is really multiple serious diseases, that you these diseases will usually affect your life a lot, is not you usually affect your own life, or mood ah, family relations, old body health can body body is not healthy trouble. Well, then you think you now the whole of your own life, because these diseases will be your life is generally satisfied?
 A: Generally speaking, it is still ok, right?
 Q: that you and the children their family relationship, and neighbor relationship these all very good?
 A: Generally,
Q: Not so much, right? How you have your children. How many children are there?
 A: Three boys and a girl.
 Q: Oh, four children. Are you a local person or a nonlocal person?
 Answer: I chaoshan come over, how long did you come over? I started working out in Shenzhen when I was 50.
 Q: I came here in 1997. I have been here for a long time. There are still more than ten days, more than 20 years away.
 Q: Well. Oh, if you come here, will you come here for your own business, or.
 A: My son did a little business.
 Q: So you came here with your son, so you came along with you?
 A: Well.
 Q: Oh, so, that's like most of the family population is here,
A: Yes, son, what daughter-in-law, grandchildren, and these are all here.
 Q: What do you think is the happiest thing in your life now is what you are most satisfied with. What are you most satisfied with in your whole state of life?
 Answer: satisfaction is this has children to have a family, have grandson. That grandson even graduated from college and came out to work.

 Q: Generally speaking, I am quite satisfied with my own feelings and my life. Still satisfied? Or not how satisfied?
 A: Satisfied.
 Q: Do you think you are living with your children or not right now?
 A: Live together.
 Q: That means that you think you live now and you say you live with your children, right?
 A: Yes.
 Q: If you live together, do you think it is quite different to live together with the young people? Or do you prefer to live alone or prefer to live with your children to answer: ah. No, no, then you are all the young people are very filial piety.

 Q: Oh, that's good, because many young people are not used to living with the old people. Generally speaking, you feel very comfortable living with them, don't they?
 Answer: That is still very filial piety.
 Q: Are you usually sick, they will accompany you to see a doctor. Will come with you, won't you? For example, what you usually take medicine, they will help you to pay attention to ah or how
A: Yes, yes.
 Q: Ok, that means you think you mean you are generally satisfied, but do you really think about it? Well, from the community, or the government, or from the community or other aspects, you can make your life a little better and happier. Do you have any good advice? What can we do? From the community, ah, or from this medical staff, ah, or from the government, ah, what do you think we can do better. It can improve your satisfaction, improve your satisfaction, improve your life happiness, then you are happier.

 Answer: that generally basically in that community now, that service also generally still can ah.
 Q: Generally speaking, do you think this kind of service in their community is ok in all aspects. Does the government have any care or special care for your elderly people?
 A: Yes, yes, yes, yes,
Q: What are the benefits.

 A: For example, it was in the countryside, ah ah, I live at the age of MAO Zedong era, it was very hard at that time. Yes, yes, that substance is very good, and you will get bored. Yes, that's different now. Since I have been in the countryside since I was 65 years ago, then the government has taken care of the old man. At first, it is 50 yuan, 50 yuan a month after 55 yuan, slowly lift, slowly lift, until now there are more than 200 points.
 Q: Every month, right?
 A: Yes
Q: every month you although you this number in the outside this situation is that proportion is very small, right? But you have so much money so much old people calculate that amount is very powerful. Then you quite understand the government.
 A: Well, yes.
 Q: Do they give you, for example, the usual care is if the community pays you if the government gives you some other benefits. Ask you how well you are. That means to care about you or to check for you. You're not having anything else.

 Answer: it is a family like me, if I also had to apply at that time, is my daughter my son-in-law she is also a civil servant.

 She works in the internal department. Well, if I were ordinary and I wouldn't want that much, I would apply to the disabled government, but I was just getting right, I didn't do that. Well, even a country is not very easy either. You see, the international situation is not good, and this country costs a lot of money.
 A lot of money, if you otherwise, if the international situation is good, we can improve a little, but no way, the world is like this.
 Ask: ah, big ye, you are what work do? I think you understand the government that way,
Answer: I farm by land, farm by oneself.
 Q: Yeah, what did you don't do when you came here in' 97?

 Answer: After doing a small business, take grandchildren.
 Q: Then you just said that you just said that they are still good. But do you feel that from your own point of view, do you as an old person, do you think they can do a little better? To make you even happier? Is there any advice from here?

 A: That's not so demanding. Ok, Ok, thank you for working hard

Q: How old are you aged now?
 A: 83 years old.
 Q: Are you a local person?
 A: My hometown is in Chaoshan. I visited Shenzhen 8 years ago, and now I live in Dongguan. He had three sons and a daughter, and the sons did business, so they moved over with him.
 Q: You look so healthy.
 A: Yes, I always get sick,
Q: But you look very energetic. What are the main diseases?
 A: This heart disease. Heart at first the body is still very good, 71 years old that year car accident ah, originally I was often often have exercise ah, this his foot broke, just stay at home for a year. He started having a bad heart at the end of age 72. Well, the heart is not good. I keep taking that heart medicine. Well. Just eat about four or five years, four or five years later began that stomach is bad, gastric ulcer, slowly chronic gastritis erosion. After this heart also this to this foot influence, began the heart is not good. The heart is not good to take medicine for a long time, the stomach is bad.be not allowed. Now you can't even take that cerebrovascular, nor is the arteriosclerosis.
 Ask: oh, this way, that this is really multiple serious diseases, that you these diseases will usually affect your life a lot, is not you usually affect your own life, or mood ah, family relations, old body health can body body is not healthy trouble. Well, then you think you now the whole of your own life, because these diseases will be your life is generally satisfied?
 A: Generally speaking, it is still ok, right?
 Q: that you and the children their family relationship, and neighbor relationship these all very good?
 A: Generally,
Q: Not so much, right? How you have your children. How many children are there?
 A: Three boys and a girl.
 Q: Oh, four children. Are you a local person or a nonlocal person?
 Answer: I chaoshan come over, how long did you come over? I started working out in Shenzhen when I was 50.
 Q: I came here in 1997. I have been here for a long time. There are still more than ten days, more than 20 years away.
 Q: Well. Oh, if you come here, will you come here for your own business, or.
 A: My son did a little business.
 Q: So you came here with your son, so you came along with you?
 A: Well.
 Q: Oh, so, that's like most of the family population is here,
A: Yes, son, what daughter-in-law, grandchildren, and these are all here.
 Q: What do you think is the happiest thing in your life now is what you are most satisfied with. What are you most satisfied with in your whole state of life?
 Answer: satisfaction is this has children to have a family, have grandson. That grandson even graduated from college and came out to work.

 Q: Generally speaking, I am quite satisfied with my own feelings and my life. Still satisfied? Or not how satisfied?
 A: Satisfied.
 Q: Do you think you are living with your children or not right now?
 A: Live together.
 Q: That means that you think you live now and you say you live with your children, right?
 A: Yes.
 Q: If you live together, do you think it is quite different to live together with the young people? Or do you prefer to live alone or prefer to live with your children to answer: ah. No, no, then you are all the young people are very filial piety.

 Q: Oh, that's good, because many young people are not used to living with the old people. Generally speaking, you feel very comfortable living with them, don't they?
 Answer: That is still very filial piety.
 Q: Are you usually sick, they will accompany you to see a doctor. Will come with you, won't you? For example, what you usually take medicine, they will help you to pay attention to ah or how
A: Yes, yes.
 Q: Ok, that means you think you mean you are generally satisfied, but do you really think about it? Well, from the community, or the government, or from the community or other aspects, you can make your life a little better and happier. Do you have any good advice? What can we do? From the community, ah, or from this medical staff, ah, or from the government, ah, what do you think we can do better. It can improve your satisfaction, improve your satisfaction, improve your life happiness, then you are happier.

 Answer: that generally basically in that community now, that service also generally still can ah.
 Q: Generally speaking, do you think this kind of service in their community is ok in all aspects. Does the government have any care or special care for your elderly people?
 A: Yes, yes, yes, yes,
Q: What are the benefits.

 A: For example, it was in the countryside, ah ah, I live at the age of MAO Zedong era, it was very hard at that time. Yes, yes, that substance is very good, and you will get bored. Yes, that's different now. Since I have been in the countryside since I was 65 years ago, then the government has taken care of the old man. At first, it is 50 yuan, 50 yuan a month after 55 yuan, slowly lift, slowly lift, until now there are more than 200 points.
 Q: Every month, right?
 A: Yes
Q: every month you although you this number in the outside this situation is that proportion is very small, right? But you have so much money so much old people calculate that amount is very powerful. Then you quite understand the government.
 A: Well, yes.
 Q: Do they give you, for example, the usual care is if the community pays you if the government gives you some other benefits. Ask you how well you are. That means to care about you or to check for you. You're not having anything else.

 Answer: it is a family like me, if I also had to apply at that time, is my daughter my son-in-law she is also a civil servant.

 She works in the internal department. Well, if I were ordinary and I wouldn't want that much, I would apply to the disabled government, but I was just getting right, I didn't do that. Well, even a country is not very easy either. You see, the international situation is not good, and this country costs a lot of money.
 A lot of money, if you otherwise, if the international situation is good, we can improve a little, but no way, the world is like this.
 Ask: ah, big ye, you are what work do? I think you understand the government that way,
Answer: I farm by land, farm by oneself.
 Q: Yeah, what did you don't do when you came here in' 97?

 Answer: After doing a small business, take grandchildren.
 Q: Then you just said that you just said that they are still good. But do you feel that from your own point of view, do you as an old person, do you think they can do a little better? To make you even happier? Is there any advice from here?

 A: That's not so demanding. Ok, Ok, thank you for working hard

Q: How old are you aged now?
 A: 84 years old.
 Q: Are you a local person?
 A: My hometown is in Chaoshan. I visited Shenzhen 9 years ago, and now I live in Dongguan. He had three sons and a daughter, and the sons did business, so they moved over with him.
 Q: You look so healthy.
 A: Yes, I always get sick,
Q: But you look very energetic. What are the main diseases?
 A: This heart disease. Heart at first the body is very good, 72 years old that year car accident, originally I was often often have exercise, this his foot broken, just stay at home for a year. He started having a bad heart at the end of age 73. Well, the heart is not good. I keep taking that heart medicine. Well. Just eat about four or five years, four or five years later began that stomach is bad, gastric ulcer, slowly chronic gastritis erosion. After this heart also this to this foot influence, began the heart is not good. The heart is not good to take medicine for a long time, the stomach is bad.be not allowed. Now you can't even take that cerebrovascular, nor is the arteriosclerosis.
 Ask: oh, this way, that this is really multiple serious diseases, that you these diseases will usually affect your life a lot, is not you usually affect your own life, or mood ah, family relations, old body health can body body is not healthy trouble. Well, then you think you now the whole of your own life, because these diseases will be your life is generally satisfied?
 A: Generally speaking, it is still ok, right?
 Q: that you and the children their family relationship, and neighbor relationship these all very good?
 A: Generally,
Q: Not so much, right? How you have your children. How many children are there?
 A: Three boys and a girl.
 Q: Oh, four children. Are you a local person or a nonlocal person?
 Answer: I chaoshan come over, how long did you come over? I started working out in Shenzhen when I was 50.
 Q: I came here in 1997. I have been here for a long time. There are still more than ten days, more than 20 years away.
 Q: Well. Oh, if you come here, will you come here for your own business, or.
 A: My son did a little business.
 Q: So you came here with your son, so you came along with you?
 A: Well.
 Q: Oh, so, that's like most of the family population is here,
A: Yes, son, what daughter-in-law, grandchildren, and these are all here.
 Q: What do you think is the happiest thing in your life now is what you are most satisfied with. What are you most satisfied with in your whole state of life?
 Answer: satisfaction is this has children to have a family, have grandson. That grandson even graduated from college and came out to work.

 Q: Generally speaking, I am quite satisfied with my own feelings and my life. Still satisfied? Or not how satisfied?
 A: Satisfied.
 Q: Do you think you are living with your children or not right now?
 A: Live together.
 Q: That means that you think you live now and you say you live with your children, right?
 A: Yes.
 Q: If you live together, do you think it is quite different to live together with the young people? Or do you prefer to live alone or prefer to live with your children to answer: ah. No, no, then you are all the young people are very filial piety.

 Q: Oh, that's good, because many young people are not used to living with the old people. Generally speaking, you feel very comfortable living with them, don't they?
 Answer: That is still very filial piety.
 Q: Are you usually sick, they will accompany you to see a doctor. Will come with you, won't you? For example, what you usually take medicine, they will help you to pay attention to ah or how
A: Yes, yes.
 Q: Ok, that means you think you mean you are generally satisfied, but do you really think about it? Well, from the community, or the government, or from the community or other aspects, you can make your life a little better and happier. Do you have any good advice? What can we do? From the community, ah, or from this medical staff, ah, or from the government, ah, what do you think we can do better. It can improve your satisfaction, improve your satisfaction, improve your life happiness, then you are happier.

 Answer: that generally basically in that community now, that service also generally still can ah.
 Q: Generally speaking, do you think this kind of service in their community is ok in all aspects. Does the government have any care or special care for your elderly people?
 A: Yes, yes, yes, yes,
Q: What are the benefits.

 A: For example, it was in the countryside, ah ah, I live at the age of MAO Zedong era, it was very hard at that time. Yes, yes, that substance is very good, and you will get bored. Yes, that's different now. Since I have been in the countryside since I was 65 years ago, then the government has taken care of the old man. At first, it is 50 yuan, 50 yuan a month after 55 yuan, slowly lift, slowly lift, until now there are more than 200 points.
 Q: Every month, right?
 A: Yes
Q: every month you although you this number in the outside this situation is that proportion is very small, right? But you have so much money so much old people calculate that amount is very powerful. Then you quite understand the government.
 A: Well, yes.
 Q: Do they give you, for example, the usual care is if the community pays you if the government gives you some other benefits. Ask you how well you are. That means to care about you or to check for you. You're not having anything else.

 Answer: it is a family like me, if I also had to apply at that time, is my daughter my son-in-law she is also a civil servant.

 She works in the internal department. Well, if I were ordinary and I wouldn't want that much, I would apply to the disabled government, but I was just getting right, I didn't do that. Well, even a country is not very easy either. You see, the international situation is not good, and this country costs a lot of money.
 A lot of money, if you otherwise, if the international situation is good, we can improve a little, but no way, the world is like this.
 Ask: ah, big ye, you are what work do? I think you understand the government that way,
Answer: I farm by land, farm by oneself.
 Q: Yeah, what did you don't do when you came here in' 97?

 Answer: After doing a small business, take grandchildren.
 Q: Then you just said that you just said that they are still good. But do you feel that from your own point of view, do you as an old person, do you think they can do a little better? To make you even happier? Is there any advice from here?

 A: That's not so demanding. Ok, Ok, thank you for working hard

Q: How old are you aged now?
 A: 85 years old.
 Q: Are you a local person?
 A: My hometown is in Chaoshan. I came to Shenzhen 10 years ago, and now I live in Dongguan. He had three sons and a daughter, and the sons did business, so they moved over with him.
 Q: You look so healthy.
 A: Yes, I always get sick,
Q: But you look very energetic. What are the main diseases?
 A: This heart disease. Heart at first the body is still very good, 73 years old that year car accident, originally I was often often have exercise, this his foot was broken, just stay at home for a year. He started having a bad heart at the end of age 74. Well, the heart is not good. I keep taking that heart medicine. Well. Just eat about four or five years, four or five years later began that stomach is bad, gastric ulcer, slowly chronic gastritis erosion. After this heart also this to this foot influence, began the heart is not good. The heart is not good to take medicine for a long time, the stomach is bad.be not allowed. Now you can't even take that cerebrovascular, nor is the arteriosclerosis.
 Ask: oh, this way, that this is really multiple serious diseases, that you these diseases will usually affect your life a lot, is not you usually affect your own life, or mood ah, family relations, old body health can body body is not healthy trouble. Well, then you think you now the whole of your own life, because these diseases will be your life is generally satisfied?
 A: Generally speaking, it is still ok, right?
 Q: that you and the children their family relationship, and neighbor relationship these all very good?
 A: Generally,
Q: Not so much, right? How you have your children. How many children are there?
 A: Three boys and a girl.
 Q: Oh, four children. Are you a local person or a nonlocal person?
 Answer: I chaoshan come over, how long did you come over? I started working out in Shenzhen when I was 50.
 Q: I came here in 1997. I have been here for a long time. There are still more than ten days, more than 20 years away.
 Q: Well. Oh, if you come here, will you come here for your own business, or.
 A: My son did a little business.
 Q: So you came here with your son, so you came along with you?
 A: Well.
 Q: Oh, so, that's like most of the family population is here,
A: Yes, son, what daughter-in-law, grandchildren, and these are all here.
 Q: What do you think is the happiest thing in your life now is what you are most satisfied with. What are you most satisfied with in your whole state of life?
 Answer: satisfaction is this has children to have a family, have grandson. That grandson even graduated from college and came out to work.

 Q: Generally speaking, I am quite satisfied with my own feelings and my life. Still satisfied? Or not how satisfied?
 A: Satisfied.
 Q: Do you think you are living with your children or not right now?
 A: Live together.
 Q: That means that you think you live now and you say you live with your children, right?
 A: Yes.
 Q: If you live together, do you think it is quite different to live together with the young people? Or do you prefer to live alone or prefer to live with your children to answer: ah. No, no, then you are all the young people are very filial piety.

 Q: Oh, that's good, because many young people are not used to living with the old people. Generally speaking, you feel very comfortable living with them, don't they?
 Answer: That is still very filial piety.
 Q: Are you usually sick, they will accompany you to see a doctor. Will come with you, won't you? For example, what you usually take medicine, they will help you to pay attention to ah or how
A: Yes, yes.
 Q: Ok, that means you think you mean you are generally satisfied, but do you really think about it? Well, from the community, or the government, or from the community or other aspects, you can make your life a little better and happier. Do you have any good advice? What can we do? From the community, ah, or from this medical staff, ah, or from the government, ah, what do you think we can do better. It can improve your satisfaction, improve your satisfaction, improve your life happiness, then you are happier.

 Answer: that generally basically in that community now, that service also generally still can ah.
 Q: Generally speaking, do you think this kind of service in their community is ok in all aspects. Does the government have any care or special care for your elderly people?
 A: Yes, yes, yes, yes,
Q: What are the benefits.

 A: For example, it was in the countryside, ah ah, I live at the age of MAO Zedong era, it was very hard at that time. Yes, yes, that substance is very good, and you will get bored. Yes, that's different now. Since I have been in the countryside since I was 65 years ago, then the government has taken care of the old man. At first, it is 50 yuan, 50 yuan a month after 55 yuan, slowly lift, slowly lift, until now there are more than 200 points.
 Q: Every month, right?
 A: Yes
Q: every month you although you this number in the outside this situation is that proportion is very small, right? But you have so much money so much old people calculate that amount is very powerful. Then you quite understand the government.
 A: Well, yes.
 Q: Do they give you, for example, the usual care is if the community pays you if the government gives you some other benefits. Ask you how well you are. That means to care about you or to check for you. You're not having anything else.

 Answer: it is a family like me, if I also had to apply at that time, is my daughter my son-in-law she is also a civil servant.

 She works in the internal department. Well, if I were ordinary and I wouldn't want that much, I would apply to the disabled government, but I was just getting right, I didn't do that. Well, even a country is not very easy either. You see, the international situation is not good, and this country costs a lot of money.
 A lot of money, if you otherwise, if the international situation is good, we can improve a little, but no way, the world is like this.
 Ask: ah, big ye, you are what work do? I think you understand the government that way,
Answer: I farm by land, farm by oneself.
 Q: Yeah, what did you don't do when you came here in' 97?

 Answer: After doing a small business, take grandchildren.
 Q: Then you just said that you just said that they are still good. But do you feel that from your own point of view, do you as an old person, do you think they can do a little better? To make you even happier? Is there any advice from here?

 A: That's not so demanding. Ok, Ok, thank you for working hard

Q: How old are you aged now?
 A: 86 years old.
 Q: Are you a local person?
 Answer: My hometown is in Chaoshan. I visited Shenzhen 6 years ago, and now I live in Dongguan. He had three sons and a daughter, and the sons did business, so they moved over with him.
 Q: You look so healthy.
 A: Yes, I always get sick,
Q: But you look very energetic. What are the main diseases?
 A: This heart disease. Heart at first the body is still very good, 69 years old that year car accident ah, originally I was often often have exercise ah, this his foot was broken, just stay at home for a year. I started having a bad heart at the end of my 70s. Well, the heart is not good. I keep taking that heart medicine. Well. Just eat about four or five years, four or five years later began that stomach is bad, gastric ulcer, slowly chronic gastritis erosion. After this heart also this to this foot influence, began the heart is not good. The heart is not good to take medicine for a long time, the stomach is bad.be not allowed. Now you can't even take that cerebrovascular, nor is the arteriosclerosis.
 Ask: oh, this way, that this is really multiple serious diseases, that you these diseases will usually affect your life a lot, is not you usually affect your own life, or mood ah, family relations, old body health can body body is not healthy trouble. Well, then you think you now the whole of your own life, because these diseases will be your life is generally satisfied?
 A: Generally speaking, it is still ok, right?
 Q: that you and the children their family relationship, and neighbor relationship these all very good?
 A: Generally,
Q: Not so much, right? How you have your children. How many children are there?
 A: Three boys and a girl.
 Q: Oh, four children. Are you a local person or a nonlocal person?
 Answer: I chaoshan come over, how long did you come over? I started working out in Shenzhen when I was 50.
 Q: I came here in 1997. I have been here for a long time. There are still more than ten days, more than 20 years away.
 Q: Well. Oh, if you come here, will you come here for your own business, or.
 A: My son did a little business.
 Q: So you came here with your son, so you came along with you?
 A: Well.
 Q: Oh, so, that's like most of the family population is here,
A: Yes, son, what daughter-in-law, grandchildren, and these are all here.
 Q: What do you think is the happiest thing in your life now is what you are most satisfied with. What are you most satisfied with in your whole state of life?
 Answer: satisfaction is this has children to have a family, have grandson. That grandson even graduated from college and came out to work.

 Q: Generally speaking, I am quite satisfied with my own feelings and my life. Still satisfied? Or not how satisfied?
 A: Satisfied.
 Q: Do you think you are living with your children or not right now?
 A: Live together.
 Q: That means that you think you live now and you say you live with your children, right?
 A: Yes.
 Q: If you live together, do you think it is quite different to live together with the young people? Or do you prefer to live alone or prefer to live with your children to answer: ah. No, no, then you are all the young people are very filial piety.

 Q: Oh, that's good, because many young people are not used to living with the old people. Generally speaking, you feel very comfortable living with them, don't they?
 Answer: That is still very filial piety.
 Q: Are you usually sick, they will accompany you to see a doctor. Will come with you, won't you? For example, what you usually take medicine, they will help you to pay attention to ah or how
A: Yes, yes.
 Q: Ok, that means you think you mean you are generally satisfied, but do you really think about it? Well, from the community, or the government, or from the community or other aspects, you can make your life a little better and happier. Do you have any good advice? What can we do? From the community, ah, or from this medical staff, ah, or from the government, ah, what do you think we can do better. It can improve your satisfaction, improve your satisfaction, improve your life happiness, then you are happier.

 Answer: that generally basically in that community now, that service also generally still can ah.
 Q: Generally speaking, do you think this kind of service in their community is ok in all aspects. Does the government have any care or special care for your elderly people?
 A: Yes, yes, yes, yes,
Q: What are the benefits.

 A: For example, it was in the countryside, ah ah, I live at the age of MAO Zedong era, it was very hard at that time. Yes, yes, that substance is very good, and you will get bored. Yes, that's different now. Since I have been in the countryside since I was 65 years ago, then the government has taken care of the old man. At first, it is 50 yuan, 50 yuan a month after 55 yuan, slowly lift, slowly lift, until now there are more than 200 points.
 Q: Every month, right?
 A: Yes
Q: every month you although you this number in the outside this situation is that proportion is very small, right? But you have so much money so much old people calculate that amount is very powerful. Then you quite understand the government.
 A: Well, yes.
 Q: Do they give you, for example, the usual care is if the community pays you if the government gives you some other benefits. Ask you how well you are. That means to care about you or to check for you. You're not having anything else.

 Answer: it is a family like me, if I also had to apply at that time, is my daughter my son-in-law she is also a civil servant.

 She works in the internal department. Well, if I were ordinary and I wouldn't want that much, I would apply to the disabled government, but I was just getting right, I didn't do that. Well, even a country is not very easy either. You see, the international situation is not good, and this country costs a lot of money.
 A lot of money, if you otherwise, if the international situation is good, we can improve a little, but no way, the world is like this.
 Ask: ah, big ye, you are what work do? I think you understand the government that way,
Answer: I farm by land, farm by oneself.
 Q: Yeah, what did you don't do when you came here in' 97?

 Answer: After doing a small business, take grandchildren.
 Q: Then you just said that you just said that they are still good. But do you feel that from your own point of view, do you as an old person, do you think they can do a little better? To make you even happier? Is there any advice from here?

 A: That's not so demanding. Ok, Ok, thank you for working hard

Q: How old are you aged now?
 A: 87 years old.
 Q: Are you a local person?
 Answer: My hometown is in Chaoshan. I visited Shenzhen 7 years ago, and now I live in Dongguan. He had three sons and a daughter, and the sons did business, so they moved over with him.
 Q: You look so healthy.
 A: Yes, I always get sick,
Q: But you look very energetic. What are the main diseases?
 A: This heart disease. Heart at first the body is still very good, 70 years old that year car accident, originally I was often often have exercise, this his foot broke, just stay at home for a year. I started having a bad heart at the end of age 71. Well, the heart is not good. I keep taking that heart medicine. Well. Just eat about four or five years, four or five years later began that stomach is bad, gastric ulcer, slowly chronic gastritis erosion. After this heart also this to this foot influence, began the heart is not good. The heart is not good to take medicine for a long time, the stomach is bad.be not allowed. Now you can't even take that cerebrovascular, nor is the arteriosclerosis.
 Ask: oh, this way, that this is really multiple serious diseases, that you these diseases will usually affect your life a lot, is not you usually affect your own life, or mood ah, family relations, old body health can body body is not healthy trouble. Well, then you think you now the whole of your own life, because these diseases will be your life is generally satisfied?
 A: Generally speaking, it is still ok, right?
 Q: that you and the children their family relationship, and neighbor relationship these all very good?
 A: Generally,
Q: Not so much, right? How you have your children. How many children are there?
 A: Three boys and a girl.
 Q: Oh, four children. Are you a local person or a nonlocal person?
 Answer: I chaoshan come over, how long did you come over? I started working out in Shenzhen when I was 50.
 Q: I came here in 1997. I have been here for a long time. There are still more than ten days, more than 20 years away.
 Q: Well. Oh, if you come here, will you come here for your own business, or.
 A: My son did a little business.
 Q: So you came here with your son, so you came along with you?
 A: Well.
 Q: Oh, so, that's like most of the family population is here,
A: Yes, son, what daughter-in-law, grandchildren, and these are all here.
 Q: What do you think is the happiest thing in your life now is what you are most satisfied with. What are you most satisfied with in your whole state of life?
 Answer: satisfaction is this has children to have a family, have grandson. That grandson even graduated from college and came out to work.

 Q: Generally speaking, I am quite satisfied with my own feelings and my life. Still satisfied? Or not how satisfied?
 A: Satisfied.
 Q: Do you think you are living with your children or not right now?
 A: Live together.
 Q: That means that you think you live now and you say you live with your children, right?
 A: Yes.
 Q: If you live together, do you think it is quite different to live together with the young people? Or do you prefer to live alone or prefer to live with your children to answer: ah. No, no, then you are all the young people are very filial piety.

 Q: Oh, that's good, because many young people are not used to living with the old people. Generally speaking, you feel very comfortable living with them, don't they?
 Answer: That is still very filial piety.
 Q: Are you usually sick, they will accompany you to see a doctor. Will come with you, won't you? For example, what you usually take medicine, they will help you to pay attention to ah or how
A: Yes, yes.
 Q: Ok, that means you think you mean you are generally satisfied, but do you really think about it? Well, from the community, or the government, or from the community or other aspects, you can make your life a little better and happier. Do you have any good advice? What can we do? From the community, ah, or from this medical staff, ah, or from the government, ah, what do you think we can do better. It can improve your satisfaction, improve your satisfaction, improve your life happiness, then you are happier.

 Answer: that generally basically in that community now, that service also generally still can ah.
 Q: Generally speaking, do you think this kind of service in their community is ok in all aspects. Does the government have any care or special care for your elderly people?
 A: Yes, yes, yes, yes,
Q: What are the benefits.

 A: For example, it was in the countryside, ah ah, I live at the age of MAO Zedong era, it was very hard at that time. Yes, yes, that substance is very good, and you will get bored. Yes, that's different now. Since I have been in the countryside since I was 65 years ago, then the government has taken care of the old man. At first, it is 50 yuan, 50 yuan a month after 55 yuan, slowly lift, slowly lift, until now there are more than 200 points.
 Q: Every month, right?
 A: Yes
Q: every month you although you this number in the outside this situation is that proportion is very small, right? But you have so much money so much old people calculate that amount is very powerful. Then you quite understand the government.
 A: Well, yes.
 Q: Do they give you, for example, the usual care is if the community pays you if the government gives you some other benefits. Ask you how well you are. That means to care about you or to check for you. You're not having anything else.

 Answer: it is a family like me, if I also had to apply at that time, is my daughter my son-in-law she is also a civil servant.

 She works in the internal department. Well, if I were ordinary and I wouldn't want that much, I would apply to the disabled government, but I was just getting right, I didn't do that. Well, even a country is not very easy either. You see, the international situation is not good, and this country costs a lot of money.
 A lot of money, if you otherwise, if the international situation is good, we can improve a little, but no way, the world is like this.
 Ask: ah, big ye, you are what work do? I think you understand the government that way,
Answer: I farm by land, farm by oneself.
 Q: Yeah, what did you don't do when you came here in' 97?

 Answer: After doing a small business, take grandchildren.
 Q: Then you just said that you just said that they are still good. But do you feel that from your own point of view, do you as an old person, do you think they can do a little better? To make you even happier? Is there any advice from here?

 A: That's not so demanding. Ok, Ok, thank you for working hard

Q: How old are you aged now?
 A: 88 years old.
 Q: Are you a local person?
 A: My hometown is in Chaoshan. I visited Shenzhen 8 years ago, and now I live in Dongguan. He had three sons and a daughter, and the sons did business, so they moved over with him.
 Q: You look so healthy.
 A: Yes, I always get sick,
Q: But you look very energetic. What are the main diseases?
 A: This heart disease. Heart at first the body is still very good, 71 years old that year car accident ah, originally I was often often have exercise ah, this his foot broke, just stay at home for a year. He started having a bad heart at the end of age 72. Well, the heart is not good. I keep taking that heart medicine. Well. Just eat about four or five years, four or five years later began that stomach is bad, gastric ulcer, slowly chronic gastritis erosion. After this heart also this to this foot influence, began the heart is not good. The heart is not good to take medicine for a long time, the stomach is bad.be not allowed. Now you can't even take that cerebrovascular, nor is the arteriosclerosis.
 Ask: oh, this way, that this is really multiple serious diseases, that you these diseases will usually affect your life a lot, is not you usually affect your own life, or mood ah, family relations, old body health can body body is not healthy trouble. Well, then you think you now the whole of your own life, because these diseases will be your life is generally satisfied?
 A: Generally speaking, it is still ok, right?
 Q: that you and the children their family relationship, and neighbor relationship these all very good?
 A: Generally,
Q: Not so much, right? How you have your children. How many children are there?
 A: Three boys and a girl.
 Q: Oh, four children. Are you a local person or a nonlocal person?
 Answer: I chaoshan come over, how long did you come over? I started working out in Shenzhen when I was 50.
 Q: I came here in 1997. I have been here for a long time. There are still more than ten days, more than 20 years away.
 Q: Well. Oh, if you come here, will you come here for your own business, or.
 A: My son did a little business.
 Q: So you came here with your son, so you came along with you?
 A: Well.
 Q: Oh, so, that's like most of the family population is here,
A: Yes, son, what daughter-in-law, grandchildren, and these are all here.
 Q: What do you think is the happiest thing in your life now is what you are most satisfied with. What are you most satisfied with in your whole state of life?
 Answer: satisfaction is this has children to have a family, have grandson. That grandson even graduated from college and came out to work.

 Q: Generally speaking, I am quite satisfied with my own feelings and my life. Still satisfied? Or not how satisfied?
 A: Satisfied.
 Q: Do you think you are living with your children or not right now?
 A: Live together.
 Q: That means that you think you live now and you say you live with your children, right?
 A: Yes.
 Q: If you live together, do you think it is quite different to live together with the young people? Or do you prefer to live alone or prefer to live with your children to answer: ah. No, no, then you are all the young people are very filial piety.

 Q: Oh, that's good, because many young people are not used to living with the old people. Generally speaking, you feel very comfortable living with them, don't they?
 Answer: That is still very filial piety.
 Q: Are you usually sick, they will accompany you to see a doctor. Will come with you, won't you? For example, what you usually take medicine, they will help you to pay attention to ah or how
A: Yes, yes.
 Q: Ok, that means you think you mean you are generally satisfied, but do you really think about it? Well, from the community, or the government, or from the community or other aspects, you can make your life a little better and happier. Do you have any good advice? What can we do? From the community, ah, or from this medical staff, ah, or from the government, ah, what do you think we can do better. It can improve your satisfaction, improve your satisfaction, improve your life happiness, then you are happier.

 Answer: that generally basically in that community now, that service also generally still can ah.
 Q: Generally speaking, do you think this kind of service in their community is ok in all aspects. Does the government have any care or special care for your elderly people?
 A: Yes, yes, yes, yes,
Q: What are the benefits.

 A: For example, it was in the countryside, ah ah, I live at the age of MAO Zedong era, it was very hard at that time. Yes, yes, that substance is very good, and you will get bored. Yes, that's different now. Since I have been in the countryside since I was 65 years ago, then the government has taken care of the old man. At first, it is 50 yuan, 50 yuan a month after 55 yuan, slowly lift, slowly lift, until now there are more than 200 points.
 Q: Every month, right?
 A: Yes
Q: every month you although you this number in the outside this situation is that proportion is very small, right? But you have so much money so much old people calculate that amount is very powerful. Then you quite understand the government.
 A: Well, yes.
 Q: Do they give you, for example, the usual care is if the community pays you if the government gives you some other benefits. Ask you how well you are. That means to care about you or to check for you. You're not having anything else.

 Answer: it is a family like me, if I also had to apply at that time, is my daughter my son-in-law she is also a civil servant.

 She works in the internal department. Well, if I were ordinary and I wouldn't want that much, I would apply to the disabled government, but I was just getting right, I didn't do that. Well, even a country is not very easy either. You see, the international situation is not good, and this country costs a lot of money.
 A lot of money, if you otherwise, if the international situation is good, we can improve a little, but no way, the world is like this.
 Ask: ah, big ye, you are what work do? I think you understand the government that way,
Answer: I farm by land, farm by oneself.
 Q: Yeah, what did you don't do when you came here in' 97?

 Answer: After doing a small business, take grandchildren.
 Q: Then you just said that you just said that they are still good. But do you feel that from your own point of view, do you as an old person, do you think they can do a little better? To make you even happier? Is there any advice from here?

 A: That's not so demanding. Ok, Ok, thank you for working hard

Q: How old are you aged now?
 A: 89 years old.
 Q: Are you a local person?
 A: My hometown is in Chaoshan. I visited Shenzhen 9 years ago, and now I live in Dongguan. He had three sons and a daughter, and the sons did business, so they moved over with him.
 Q: You look so healthy.
 A: Yes, I always get sick,
Q: But you look very energetic. What are the main diseases?
 A: This heart disease. Heart at first the body is very good, 72 years old that year car accident, originally I was often often have exercise, this his foot broken, just stay at home for a year. He started having a bad heart at the end of age 73. Well, the heart is not good. I keep taking that heart medicine. Well. Just eat about four or five years, four or five years later began that stomach is bad, gastric ulcer, slowly chronic gastritis erosion. After this heart also this to this foot influence, began the heart is not good. The heart is not good to take medicine for a long time, the stomach is bad.be not allowed. Now you can't even take that cerebrovascular, nor is the arteriosclerosis.
 Ask: oh, this way, that this is really multiple serious diseases, that you these diseases will usually affect your life a lot, is not you usually affect your own life, or mood ah, family relations, old body health can body body is not healthy trouble. Well, then you think you now the whole of your own life, because these diseases will be your life is generally satisfied?
 A: Generally speaking, it is still ok, right?
 Q: that you and the children their family relationship, and neighbor relationship these all very good?
 A: Generally,
Q: Not so much, right? How you have your children. How many children are there?
 A: Three boys and a girl.
 Q: Oh, four children. Are you a local person or a nonlocal person?
 Answer: I chaoshan come over, how long did you come over? I started working out in Shenzhen when I was 50.
 Q: I came here in 1997. I have been here for a long time. There are still more than ten days, more than 20 years away.
 Q: Well. Oh, if you come here, will you come here for your own business, or.
 A: My son did a little business.
 Q: So you came here with your son, so you came along with you?
 A: Well.
 Q: Oh, so, that's like most of the family population is here,
A: Yes, son, what daughter-in-law, grandchildren, and these are all here.
 Q: What do you think is the happiest thing in your life now is what you are most satisfied with. What are you most satisfied with in your whole state of life?
 Answer: satisfaction is this has children to have a family, have grandson. That grandson even graduated from college and came out to work.

 Q: Generally speaking, I am quite satisfied with my own feelings and my life. Still satisfied? Or not how satisfied?
 A: Satisfied.
 Q: Do you think you are living with your children or not right now?
 A: Live together.
 Q: That means that you think you live now and you say you live with your children, right?
 A: Yes.
 Q: If you live together, do you think it is quite different to live together with the young people? Or do you prefer to live alone or prefer to live with your children to answer: ah. No, no, then you are all the young people are very filial piety.

 Q: Oh, that's good, because many young people are not used to living with the old people. Generally speaking, you feel very comfortable living with them, don't they?
 Answer: That is still very filial piety.
 Q: Are you usually sick, they will accompany you to see a doctor. Will come with you, won't you? For example, what you usually take medicine, they will help you to pay attention to ah or how
A: Yes, yes.
 Q: Ok, that means you think you mean you are generally satisfied, but do you really think about it? Well, from the community, or the government, or from the community or other aspects, you can make your life a little better and happier. Do you have any good advice? What can we do? From the community, ah, or from this medical staff, ah, or from the government, ah, what do you think we can do better. It can improve your satisfaction, improve your satisfaction, improve your life happiness, then you are happier.

 Answer: that generally basically in that community now, that service also generally still can ah.
 Q: Generally speaking, do you think this kind of service in their community is ok in all aspects. Does the government have any care or special care for your elderly people?
 A: Yes, yes, yes, yes,
Q: What are the benefits.

 A: For example, it was in the countryside, ah ah, I live at the age of MAO Zedong era, it was very hard at that time. Yes, yes, that substance is very good, and you will get bored. Yes, that's different now. Since I have been in the countryside since I was 65 years ago, then the government has taken care of the old man. At first, it is 50 yuan, 50 yuan a month after 55 yuan, slowly lift, slowly lift, until now there are more than 200 points.
 Q: Every month, right?
 A: Yes
Q: every month you although you this number in the outside this situation is that proportion is very small, right? But you have so much money so much old people calculate that amount is very powerful. Then you quite understand the government.
 A: Well, yes.
 Q: Do they give you, for example, the usual care is if the community pays you if the government gives you some other benefits. Ask you how well you are. That means to care about you or to check for you. You're not having anything else.

 Answer: it is a family like me, if I also had to apply at that time, is my daughter my son-in-law she is also a civil servant.

 She works in the internal department. Well, if I were ordinary and I wouldn't want that much, I would apply to the disabled government, but I was just getting right, I didn't do that. Well, even a country is not very easy either. You see, the international situation is not good, and this country costs a lot of money.
 A lot of money, if you otherwise, if the international situation is good, we can improve a little, but no way, the world is like this.
 Ask: ah, big ye, you are what work do? I think you understand the government that way,
Answer: I farm by land, farm by oneself.
 Q: Yeah, what did you don't do when you came here in' 97?

 Answer: After doing a small business, take grandchildren.
 Q: Then you just said that you just said that they are still good. But do you feel that from your own point of view, do you as an old person, do you think they can do a little better? To make you even happier? Is there any advice from here?

 A: That's not so demanding. Ok, Ok, thank you for working hard

Q: How old are you aged now?
 A: 90 years old.
 Q: Are you a local person?
 A: My hometown is in Chaoshan. I came to Shenzhen 10 years ago, and now I live in Dongguan. He had three sons and a daughter, and the sons did business, so they moved over with him.
 Q: You look so healthy.
 A: Yes, I always get sick,
Q: But you look very energetic. What are the main diseases?
 A: This heart disease. Heart at first the body is still very good, 73 years old that year car accident, originally I was often often have exercise, this his foot was broken, just stay at home for a year. He started having a bad heart at the end of age 74. Well, the heart is not good. I keep taking that heart medicine. Well. Just eat about four or five years, four or five years later began that stomach is bad, gastric ulcer, slowly chronic gastritis erosion. After this heart also this to this foot influence, began the heart is not good. The heart is not good to take medicine for a long time, the stomach is bad.be not allowed. Now you can't even take that cerebrovascular, nor is the arteriosclerosis.
 Ask: oh, this way, that this is really multiple serious diseases, that you these diseases will usually affect your life a lot, is not you usually affect your own life, or mood ah, family relations, old body health can body body is not healthy trouble. Well, then you think you now the whole of your own life, because these diseases will be your life is generally satisfied?
 A: Generally speaking, it is still ok, right?
 Q: that you and the children their family relationship, and neighbor relationship these all very good?
 A: Generally,
Q: Not so much, right? How you have your children. How many children are there?
 A: Three boys and a girl.
 Q: Oh, four children. Are you a local person or a nonlocal person?
 Answer: I chaoshan come over, how long did you come over? I started working out in Shenzhen when I was 50.
 Q: I came here in 1997. I have been here for a long time. There are still more than ten days, more than 20 years away.
 Q: Well. Oh, if you come here, will you come here for your own business, or.
 A: My son did a little business.
 Q: So you came here with your son, so you came along with you?
 A: Well.
 Q: Oh, so, that's like most of the family population is here,
A: Yes, son, what daughter-in-law, grandchildren, and these are all here.
 Q: What do you think is the happiest thing in your life now is what you are most satisfied with. What are you most satisfied with in your whole state of life?
 Answer: satisfaction is this has children to have a family, have grandson. That grandson even graduated from college and came out to work.

 Q: Generally speaking, I am quite satisfied with my own feelings and my life. Still satisfied? Or not how satisfied?
 A: Satisfied.
 Q: Do you think you are living with your children or not right now?
 A: Live together.
 Q: That means that you think you live now and you say you live with your children, right?
 A: Yes.
 Q: If you live together, do you think it is quite different to live together with the young people? Or do you prefer to live alone or prefer to live with your children to answer: ah. No, no, then you are all the young people are very filial piety.

 Q: Oh, that's good, because many young people are not used to living with the old people. Generally speaking, you feel very comfortable living with them, don't they?
 Answer: That is still very filial piety.
 Q: Are you usually sick, they will accompany you to see a doctor. Will come with you, won't you? For example, what you usually take medicine, they will help you to pay attention to ah or how
A: Yes, yes.
 Q: Ok, that means you think you mean you are generally satisfied, but do you really think about it? Well, from the community, or the government, or from the community or other aspects, you can make your life a little better and happier. Do you have any good advice? What can we do? From the community, ah, or from this medical staff, ah, or from the government, ah, what do you think we can do better. It can improve your satisfaction, improve your satisfaction, improve your life happiness, then you are happier.

 Answer: that generally basically in that community now, that service also generally still can ah.
 Q: Generally speaking, do you think this kind of service in their community is ok in all aspects. Does the government have any care or special care for your elderly people?
 A: Yes, yes, yes, yes,
Q: What are the benefits.

 A: For example, it was in the countryside, ah ah, I live at the age of MAO Zedong era, it was very hard at that time. Yes, yes, that substance is very good, and you will get bored. Yes, that's different now. Since I have been in the countryside since I was 65 years ago, then the government has taken care of the old man. At first, it is 50 yuan, 50 yuan a month after 55 yuan, slowly lift, slowly lift, until now there are more than 200 points.
 Q: Every month, right?
 A: Yes
Q: every month you although you this number in the outside this situation is that proportion is very small, right? But you have so much money so much old people calculate that amount is very powerful. Then you quite understand the government.
 A: Well, yes.
 Q: Do they give you, for example, the usual care is if the community pays you if the government gives you some other benefits. Ask you how well you are. That means to care about you or to check for you. You're not having anything else.

 Answer: it is a family like me, if I also had to apply at that time, is my daughter my son-in-law she is also a civil servant.

 She works in the internal department. Well, if I were ordinary and I wouldn't want that much, I would apply to the disabled government, but I was just getting right, I didn't do that. Well, even a country is not very easy either. You see, the international situation is not good, and this country costs a lot of money.
 A lot of money, if you otherwise, if the international situation is good, we can improve a little, but no way, the world is like this.
 Ask: ah, big ye, you are what work do? I think you understand the government that way,
Answer: I farm by land, farm by oneself.
 Q: Yeah, what did you don't do when you came here in' 97?

 Answer: After doing a small business, take grandchildren.
 Q: Then you just said that you just said that they are still good. But do you feel that from your own point of view, do you as an old person, do you think they can do a little better? To make you even happier? Is there any advice from here?

 A: That's not so demanding. Ok, Ok, thank you for working hard

Q: How old are you aged now?
 A: 81 years old.
 Q: Are you a local person?
 Answer: My hometown is in Chaoshan. I visited Shenzhen 6 years ago, and now I live in Dongguan. He had three sons and a daughter, and the sons did business, so they moved over with him.
 Q: You look so healthy.
 A: Yes, I always get sick,
Q: But you look very energetic. What are the main diseases?
 A: This heart disease. Heart at first the body is still very good, 69 years old that year car accident ah, originally I was often often have exercise ah, this his foot was broken, just stay at home for a year. I started having a bad heart at the end of my 70s. Well, the heart is not good. I keep taking that heart medicine. Well. Just eat about four or five years, four or five years later began that stomach is bad, gastric ulcer, slowly chronic gastritis erosion. After this heart also this to this foot influence, began the heart is not good. The heart is not good to take medicine for a long time, the stomach is bad.be not allowed. Now you can't even take that cerebrovascular, nor is the arteriosclerosis.
 Ask: oh, this way, that this is really multiple serious diseases, that you these diseases will usually affect your life a lot, is not you usually affect your own life, or mood ah, family relations, old body health can body body is not healthy trouble. Well, then you think you now the whole of your own life, because these diseases will be your life is generally satisfied?
 A: Generally speaking, it is still ok, right?
 Q: that you and the children their family relationship, and neighbor relationship these all very good?
 A: Generally,
Q: Not so much, right? How you have your children. How many children are there?
 A: Three boys and a girl.
 Q: Oh, four children. Are you a local person or a nonlocal person?
 Answer: I chaoshan come over, how long did you come over? I started working out in Shenzhen when I was 50.
 Q: I came here in 1997. I have been here for a long time. There are still more than ten days, more than 20 years away.
 Q: Well. Oh, if you come here, will you come here for your own business, or.
 A: My son did a little business.
 Q: So you came here with your son, so you came along with you?
 A: Well.
 Q: Oh, so, that's like most of the family population is here,
A: Yes, son, what daughter-in-law, grandchildren, and these are all here.
 Q: What do you think is the happiest thing in your life now is what you are most satisfied with. What are you most satisfied with in your whole state of life?
 Answer: satisfaction is this has children to have a family, have grandson. That grandson even graduated from college and came out to work.

 Q: Generally speaking, I am quite satisfied with my own feelings and my life. Still satisfied? Or not how satisfied?
 A: Satisfied.
 Q: Do you think you are living with your children or not right now?
 A: Live together.
 Q: That means that you think you live now and you say you live with your children, right?
 A: Yes.
 Q: If you live together, do you think it is quite different to live together with the young people? Or do you prefer to live alone or prefer to live with your children to answer: ah. No, no, then you are all the young people are very filial piety.

 Q: Oh, that's good, because many young people are not used to living with the old people. Generally speaking, you feel very comfortable living with them, don't they?
 Answer: That is still very filial piety.
 Q: Are you usually sick, they will accompany you to see a doctor. Will come with you, won't you? For example, what you usually take medicine, they will help you to pay attention to ah or how
A: Yes, yes.
 Q: Ok, that means you think you mean you are generally satisfied, but do you really think about it? Well, from the community, or the government, or from the community or other aspects, you can make your life a little better and happier. Do you have any good advice? What can we do? From the community, ah, or from this medical staff, ah, or from the government, ah, what do you think we can do better. It can improve your satisfaction, improve your satisfaction, improve your life happiness, then you are happier.

 Answer: that generally basically in that community now, that service also generally still can ah.
 Q: Generally speaking, do you think this kind of service in their community is ok in all aspects. Does the government have any care or special care for your elderly people?
 A: Yes, yes, yes, yes,
Q: What are the benefits.

 A: For example, it was in the countryside, ah ah, I live at the age of MAO Zedong era, it was very hard at that time. Yes, yes, that substance is very good, and you will get bored. Yes, that's different now. Since I have been in the countryside since I was 65 years ago, then the government has taken care of the old man. At first, it is 50 yuan, 50 yuan a month after 55 yuan, slowly lift, slowly lift, until now there are more than 200 points.
 Q: Every month, right?
 A: Yes
Q: every month you although you this number in the outside this situation is that proportion is very small, right? But you have so much money so much old people calculate that amount is very powerful. Then you quite understand the government.
 A: Well, yes.
 Q: Do they give you, for example, the usual care is if the community pays you if the government gives you some other benefits. Ask you how well you are. That means to care about you or to check for you. You're not having anything else.

 Answer: it is a family like me, if I also had to apply at that time, is my daughter my son-in-law she is also a civil servant.

 She works in the internal department. Well, if I were ordinary and I wouldn't want that much, I would apply to the disabled government, but I was just getting right, I didn't do that. Well, even a country is not very easy either. You see, the international situation is not good, and this country costs a lot of money.
 A lot of money, if you otherwise, if the international situation is good, we can improve a little, but no way, the world is like this.
 Ask: ah, big ye, you are what work do? I think you understand the government that way,
Answer: I farm by land, farm by oneself.
 Q: Yeah, what did you don't do when you came here in' 97?

 Answer: After doing a small business, take grandchildren.
 Q: Then you just said that you just said that they are still good. But do you feel that from your own point of view, do you as an old person, do you think they can do a little better? To make you even happier? Is there any advice from here?

 A: That's not so demanding. Ok, Ok, thank you for working hard

Q: How old are you aged now?
 A: 82 years old.
 Q: Are you a local person?
 Answer: My hometown is in Chaoshan. I visited Shenzhen 7 years ago, and now I live in Dongguan. He had three sons and a daughter, and the sons did business, so they moved over with him.
 Q: You look so healthy.
 A: Yes, I always get sick,
Q: But you look very energetic. What are the main diseases?
 A: This heart disease. Heart at first the body is still very good, 70 years old that year car accident, originally I was often often have exercise, this his foot broke, just stay at home for a year. I started having a bad heart at the end of age 71. Well, the heart is not good. I keep taking that heart medicine. Well. Just eat about four or five years, four or five years later began that stomach is bad, gastric ulcer, slowly chronic gastritis erosion. After this heart also this to this foot influence, began the heart is not good. The heart is not good to take medicine for a long time, the stomach is bad.be not allowed. Now you can't even take that cerebrovascular, nor is the arteriosclerosis.
 Ask: oh, this way, that this is really multiple serious diseases, that you these diseases will usually affect your life a lot, is not you usually affect your own life, or mood ah, family relations, old body health can body body is not healthy trouble. Well, then you think you now the whole of your own life, because these diseases will be your life is generally satisfied?
 A: Generally speaking, it is still ok, right?
 Q: that you and the children their family relationship, and neighbor relationship these all very good?
 A: Generally,
Q: Not so much, right? How you have your children. How many children are there?
 A: Three boys and a girl.
 Q: Oh, four children. Are you a local person or a nonlocal person?
 Answer: I chaoshan come over, how long did you come over? I started working out in Shenzhen when I was 50.
 Q: I came here in 1997. I have been here for a long time. There are still more than ten days, more than 20 years away.
 Q: Well. Oh, if you come here, will you come here for your own business, or.
 A: My son did a little business.
 Q: So you came here with your son, so you came along with you?
 A: Well.
 Q: Oh, so, that's like most of the family population is here,
A: Yes, son, what daughter-in-law, grandchildren, and these are all here.
 Q: What do you think is the happiest thing in your life now is what you are most satisfied with. What are you most satisfied with in your whole state of life?
 Answer: satisfaction is this has children to have a family, have grandson. That grandson even graduated from college and came out to work.

 Q: Generally speaking, I am quite satisfied with my own feelings and my life. Still satisfied? Or not how satisfied?
 A: Satisfied.
 Q: Do you think you are living with your children or not right now?
 A: Live together.
 Q: That means that you think you live now and you say you live with your children, right?
 A: Yes.
 Q: If you live together, do you think it is quite different to live together with the young people? Or do you prefer to live alone or prefer to live with your children to answer: ah. No, no, then you are all the young people are very filial piety.

 Q: Oh, that's good, because many young people are not used to living with the old people. Generally speaking, you feel very comfortable living with them, don't they?
 Answer: That is still very filial piety.
 Q: Are you usually sick, they will accompany you to see a doctor. Will come with you, won't you? For example, what you usually take medicine, they will help you to pay attention to ah or how
A: Yes, yes.
 Q: Ok, that means you think you mean you are generally satisfied, but do you really think about it? Well, from the community, or the government, or from the community or other aspects, you can make your life a little better and happier. Do you have any good advice? What can we do? From the community, ah, or from this medical staff, ah, or from the government, ah, what do you think we can do better. It can improve your satisfaction, improve your satisfaction, improve your life happiness, then you are happier.

 Answer: that generally basically in that community now, that service also generally still can ah.
 Q: Generally speaking, do you think this kind of service in their community is ok in all aspects. Does the government have any care or special care for your elderly people?
 A: Yes, yes, yes, yes,
Q: What are the benefits.

 A: For example, it was in the countryside, ah ah, I live at the age of MAO Zedong era, it was very hard at that time. Yes, yes, that substance is very good, and you will get bored. Yes, that's different now. Since I have been in the countryside since I was 65 years ago, then the government has taken care of the old man. At first, it is 50 yuan, 50 yuan a month after 55 yuan, slowly lift, slowly lift, until now there are more than 200 points.
 Q: Every month, right?
 A: Yes
Q: every month you although you this number in the outside this situation is that proportion is very small, right? But you have so much money so much old people calculate that amount is very powerful. Then you quite understand the government.
 A: Well, yes.
 Q: Do they give you, for example, the usual care is if the community pays you if the government gives you some other benefits. Ask you how well you are. That means to care about you or to check for you. You're not having anything else.

 Answer: it is a family like me, if I also had to apply at that time, is my daughter my son-in-law she is also a civil servant.

 She works in the internal department. Well, if I were ordinary and I wouldn't want that much, I would apply to the disabled government, but I was just getting right, I didn't do that. Well, even a country is not very easy either. You see, the international situation is not good, and this country costs a lot of money.
 A lot of money, if you otherwise, if the international situation is good, we can improve a little, but no way, the world is like this.
 Ask: ah, big ye, you are what work do? I think you understand the government that way,
Answer: I farm by land, farm by oneself.
 Q: Yeah, what did you don't do when you came here in' 97?

 Answer: After doing a small business, take grandchildren.
 Q: Then you just said that you just said that they are still good. But do you feel that from your own point of view, do you as an old person, do you think they can do a little better? To make you even happier? Is there any advice from here?

 A: That's not so demanding. Ok, Ok, thank you for working hard

Q: How old are you aged now?
 A: 83 years old.
 Q: Are you a local person?
 A: My hometown is in Chaoshan. I visited Shenzhen 8 years ago, and now I live in Dongguan. He had three sons and a daughter, and the sons did business, so they moved over with him.
 Q: You look so healthy.
 A: Yes, I always get sick,
Q: But you look very energetic. What are the main diseases?
 A: This heart disease. Heart at first the body is still very good, 71 years old that year car accident ah, originally I was often often have exercise ah, this his foot broke, just stay at home for a year. He started having a bad heart at the end of age 72. Well, the heart is not good. I keep taking that heart medicine. Well. Just eat about four or five years, four or five years later began that stomach is bad, gastric ulcer, slowly chronic gastritis erosion. After this heart also this to this foot influence, began the heart is not good. The heart is not good to take medicine for a long time, the stomach is bad.be not allowed. Now you can't even take that cerebrovascular, nor is the arteriosclerosis.
 Ask: oh, this way, that this is really multiple serious diseases, that you these diseases will usually affect your life a lot, is not you usually affect your own life, or mood ah, family relations, old body health can body body is not healthy trouble. Well, then you think you now the whole of your own life, because these diseases will be your life is generally satisfied?
 A: Generally speaking, it is still ok, right?
 Q: that you and the children their family relationship, and neighbor relationship these all very good?
 A: Generally,
Q: Not so much, right? How you have your children. How many children are there?
 A: Three boys and a girl.
 Q: Oh, four children. Are you a local person or a nonlocal person?
 Answer: I chaoshan come over, how long did you come over? I started working out in Shenzhen when I was 50.
 Q: I came here in 1997. I have been here for a long time. There are still more than ten days, more than 20 years away.
 Q: Well. Oh, if you come here, will you come here for your own business, or.
 A: My son did a little business.
 Q: So you came here with your son, so you came along with you?
 A: Well.
 Q: Oh, so, that's like most of the family population is here,
A: Yes, son, what daughter-in-law, grandchildren, and these are all here.
 Q: What do you think is the happiest thing in your life now is what you are most satisfied with. What are you most satisfied with in your whole state of life?
 Answer: satisfaction is this has children to have a family, have grandson. That grandson even graduated from college and came out to work.

 Q: Generally speaking, I am quite satisfied with my own feelings and my life. Still satisfied? Or not how satisfied?
 A: Satisfied.
 Q: Do you think you are living with your children or not right now?
 A: Live together.
 Q: That means that you think you live now and you say you live with your children, right?
 A: Yes.
 Q: If you live together, do you think it is quite different to live together with the young people? Or do you prefer to live alone or prefer to live with your children to answer: ah. No, no, then you are all the young people are very filial piety.

 Q: Oh, that's good, because many young people are not used to living with the old people. Generally speaking, you feel very comfortable living with them, don't they?
 Answer: That is still very filial piety.
 Q: Are you usually sick, they will accompany you to see a doctor. Will come with you, won't you? For example, what you usually take medicine, they will help you to pay attention to ah or how
A: Yes, yes.
 Q: Ok, that means you think you mean you are generally satisfied, but do you really think about it? Well, from the community, or the government, or from the community or other aspects, you can make your life a little better and happier. Do you have any good advice? What can we do? From the community, ah, or from this medical staff, ah, or from the government, ah, what do you think we can do better. It can improve your satisfaction, improve your satisfaction, improve your life happiness, then you are happier.

 Answer: that generally basically in that community now, that service also generally still can ah.
 Q: Generally speaking, do you think this kind of service in their community is ok in all aspects. Does the government have any care or special care for your elderly people?
 A: Yes, yes, yes, yes,
Q: What are the benefits.

 A: For example, it was in the countryside, ah ah, I live at the age of MAO Zedong era, it was very hard at that time. Yes, yes, that substance is very good, and you will get bored. Yes, that's different now. Since I have been in the countryside since I was 65 years ago, then the government has taken care of the old man. At first, it is 50 yuan, 50 yuan a month after 55 yuan, slowly lift, slowly lift, until now there are more than 200 points.
 Q: Every month, right?
 A: Yes
Q: every month you although you this number in the outside this situation is that proportion is very small, right? But you have so much money so much old people calculate that amount is very powerful. Then you quite understand the government.
 A: Well, yes.
 Q: Do they give you, for example, the usual care is if the community pays you if the government gives you some other benefits. Ask you how well you are. That means to care about you or to check for you. You're not having anything else.

 Answer: it is a family like me, if I also had to apply at that time, is my daughter my son-in-law she is also a civil servant.

 She works in the internal department. Well, if I were ordinary and I wouldn't want that much, I would apply to the disabled government, but I was just getting right, I didn't do that. Well, even a country is not very easy either. You see, the international situation is not good, and this country costs a lot of money.
 A lot of money, if you otherwise, if the international situation is good, we can improve a little, but no way, the world is like this.
 Ask: ah, big ye, you are what work do? I think you understand the government that way,
Answer: I farm by land, farm by oneself.
 Q: Yeah, what did you don't do when you came here in' 97?

 Answer: After doing a small business, take grandchildren.
 Q: Then you just said that you just said that they are still good. But do you feel that from your own point of view, do you as an old person, do you think they can do a little better? To make you even happier? Is there any advice from here?

 A: That's not so demanding. Ok, Ok, thank you for working hard

Q: How old are you aged now?
 A: 84 years old.
 Q: Are you a local person?
 A: My hometown is in Chaoshan. I visited Shenzhen 9 years ago, and now I live in Dongguan. He had three sons and a daughter, and the sons did business, so they moved over with him.
 Q: You look so healthy.
 A: Yes, I always get sick,
Q: But you look very energetic. What are the main diseases?
 A: This heart disease. Heart at first the body is very good, 72 years old that year car accident, originally I was often often have exercise, this his foot broken, just stay at home for a year. He started having a bad heart at the end of age 73. Well, the heart is not good. I keep taking that heart medicine. Well. Just eat about four or five years, four or five years later began that stomach is bad, gastric ulcer, slowly chronic gastritis erosion. After this heart also this to this foot influence, began the heart is not good. The heart is not good to take medicine for a long time, the stomach is bad.be not allowed. Now you can't even take that cerebrovascular, nor is the arteriosclerosis.
 Ask: oh, this way, that this is really multiple serious diseases, that you these diseases will usually affect your life a lot, is not you usually affect your own life, or mood ah, family relations, old body health can body body is not healthy trouble. Well, then you think you now the whole of your own life, because these diseases will be your life is generally satisfied?
 A: Generally speaking, it is still ok, right?
 Q: that you and the children their family relationship, and neighbor relationship these all very good?
 A: Generally,
Q: Not so much, right? How you have your children. How many children are there?
 A: Three boys and a girl.
 Q: Oh, four children. Are you a local person or a nonlocal person?
 Answer: I chaoshan come over, how long did you come over? I started working out in Shenzhen when I was 50.
 Q: I came here in 1997. I have been here for a long time. There are still more than ten days, more than 20 years away.
 Q: Well. Oh, if you come here, will you come here for your own business, or.
 A: My son did a little business.
 Q: So you came here with your son, so you came along with you?
 A: Well.
 Q: Oh, so, that's like most of the family population is here,
A: Yes, son, what daughter-in-law, grandchildren, and these are all here.
 Q: What do you think is the happiest thing in your life now is what you are most satisfied with. What are you most satisfied with in your whole state of life?
 Answer: satisfaction is this has children to have a family, have grandson. That grandson even graduated from college and came out to work.

 Q: Generally speaking, I am quite satisfied with my own feelings and my life. Still satisfied? Or not how satisfied?
 A: Satisfied.
 Q: Do you think you are living with your children or not right now?
 A: Live together.
 Q: That means that you think you live now and you say you live with your children, right?
 A: Yes.
 Q: If you live together, do you think it is quite different to live together with the young people? Or do you prefer to live alone or prefer to live with your children to answer: ah. No, no, then you are all the young people are very filial piety.

 Q: Oh, that's good, because many young people are not used to living with the old people. Generally speaking, you feel very comfortable living with them, don't they?
 Answer: That is still very filial piety.
 Q: Are you usually sick, they will accompany you to see a doctor. Will come with you, won't you? For example, what you usually take medicine, they will help you to pay attention to ah or how
A: Yes, yes.
 Q: Ok, that means you think you mean you are generally satisfied, but do you really think about it? Well, from the community, or the government, or from the community or other aspects, you can make your life a little better and happier. Do you have any good advice? What can we do? From the community, ah, or from this medical staff, ah, or from the government, ah, what do you think we can do better. It can improve your satisfaction, improve your satisfaction, improve your life happiness, then you are happier.

 Answer: that generally basically in that community now, that service also generally still can ah.
 Q: Generally speaking, do you think this kind of service in their community is ok in all aspects. Does the government have any care or special care for your elderly people?
 A: Yes, yes, yes, yes,
Q: What are the benefits.

 A: For example, it was in the countryside, ah ah, I live at the age of MAO Zedong era, it was very hard at that time. Yes, yes, that substance is very good, and you will get bored. Yes, that's different now. Since I have been in the countryside since I was 65 years ago, then the government has taken care of the old man. At first, it is 50 yuan, 50 yuan a month after 55 yuan, slowly lift, slowly lift, until now there are more than 200 points.
 Q: Every month, right?
 A: Yes
Q: every month you although you this number in the outside this situation is that proportion is very small, right? But you have so much money so much old people calculate that amount is very powerful. Then you quite understand the government.
 A: Well, yes.
 Q: Do they give you, for example, the usual care is if the community pays you if the government gives you some other benefits. Ask you how well you are. That means to care about you or to check for you. You're not having anything else.

 Answer: it is a family like me, if I also had to apply at that time, is my daughter my son-in-law she is also a civil servant.

 She works in the internal department. Well, if I were ordinary and I wouldn't want that much, I would apply to the disabled government, but I was just getting right, I didn't do that. Well, even a country is not very easy either. You see, the international situation is not good, and this country costs a lot of money.
 A lot of money, if you otherwise, if the international situation is good, we can improve a little, but no way, the world is like this.
 Ask: ah, big ye, you are what work do? I think you understand the government that way,
Answer: I farm by land, farm by oneself.
 Q: Yeah, what did you don't do when you came here in' 97?

 Answer: After doing a small business, take grandchildren.
 Q: Then you just said that you just said that they are still good. But do you feel that from your own point of view, do you as an old person, do you think they can do a little better? To make you even happier? Is there any advice from here?

 A: That's not so demanding. Ok, Ok, thank you for working hard

Q: How old are you aged now?
 A: 85 years old.
 Q: Are you a local person?
 A: My hometown is in Chaoshan. I came to Shenzhen 10 years ago, and now I live in Dongguan. He had three sons and a daughter, and the sons did business, so they moved over with him.
 Q: You look so healthy.
 A: Yes, I always get sick,
Q: But you look very energetic. What are the main diseases?
 A: This heart disease. Heart at first the body is still very good, 73 years old that year car accident, originally I was often often have exercise, this his foot was broken, just stay at home for a year. He started having a bad heart at the end of age 74. Well, the heart is not good. I keep taking that heart medicine. Well. Just eat about four or five years, four or five years later began that stomach is bad, gastric ulcer, slowly chronic gastritis erosion. After this heart also this to this foot influence, began the heart is not good. The heart is not good to take medicine for a long time, the stomach is bad.be not allowed. Now you can't even take that cerebrovascular, nor is the arteriosclerosis.
 Ask: oh, this way, that this is really multiple serious diseases, that you these diseases will usually affect your life a lot, is not you usually affect your own life, or mood ah, family relations, old body health can body body is not healthy trouble. Well, then you think you now the whole of your own life, because these diseases will be your life is generally satisfied?
 A: Generally speaking, it is still ok, right?
 Q: that you and the children their family relationship, and neighbor relationship these all very good?
 A: Generally,
Q: Not so much, right? How you have your children. How many children are there?
 A: Three boys and a girl.
 Q: Oh, four children. Are you a local person or a nonlocal person?
 Answer: I chaoshan come over, how long did you come over? I started working out in Shenzhen when I was 50.
 Q: I came here in 1997. I have been here for a long time. There are still more than ten days, more than 20 years away.
 Q: Well. Oh, if you come here, will you come here for your own business, or.
 A: My son did a little business.
 Q: So you came here with your son, so you came along with you?
 A: Well.
 Q: Oh, so, that's like most of the family population is here,
A: Yes, son, what daughter-in-law, grandchildren, and these are all here.
 Q: What do you think is the happiest thing in your life now is what you are most satisfied with. What are you most satisfied with in your whole state of life?
 Answer: satisfaction is this has children to have a family, have grandson. That grandson even graduated from college and came out to work.

 Q: Generally speaking, I am quite satisfied with my own feelings and my life. Still satisfied? Or not how satisfied?
 A: Satisfied.
 Q: Do you think you are living with your children or not right now?
 A: Live together.
 Q: That means that you think you live now and you say you live with your children, right?
 A: Yes.
 Q: If you live together, do you think it is quite different to live together with the young people? Or do you prefer to live alone or prefer to live with your children to answer: ah. No, no, then you are all the young people are very filial piety.

 Q: Oh, that's good, because many young people are not used to living with the old people. Generally speaking, you feel very comfortable living with them, don't they?
 Answer: That is still very filial piety.
 Q: Are you usually sick, they will accompany you to see a doctor. Will come with you, won't you? For example, what you usually take medicine, they will help you to pay attention to ah or how
A: Yes, yes.
 Q: Ok, that means you think you mean you are generally satisfied, but do you really think about it? Well, from the community, or the government, or from the community or other aspects, you can make your life a little better and happier. Do you have any good advice? What can we do? From the community, ah, or from this medical staff, ah, or from the government, ah, what do you think we can do better. It can improve your satisfaction, improve your satisfaction, improve your life happiness, then you are happier.

 Answer: that generally basically in that community now, that service also generally still can ah.
 Q: Generally speaking, do you think this kind of service in their community is ok in all aspects. Does the government have any care or special care for your elderly people?
 A: Yes, yes, yes, yes,
Q: What are the benefits.

 A: For example, it was in the countryside, ah ah, I live at the age of MAO Zedong era, it was very hard at that time. Yes, yes, that substance is very good, and you will get bored. Yes, that's different now. Since I have been in the countryside since I was 65 years ago, then the government has taken care of the old man. At first, it is 50 yuan, 50 yuan a month after 55 yuan, slowly lift, slowly lift, until now there are more than 200 points.
 Q: Every month, right?
 A: Yes
Q: every month you although you this number in the outside this situation is that proportion is very small, right? But you have so much money so much old people calculate that amount is very powerful. Then you quite understand the government.
 A: Well, yes.
 Q: Do they give you, for example, the usual care is if the community pays you if the government gives you some other benefits. Ask you how well you are. That means to care about you or to check for you. You're not having anything else.

 Answer: it is a family like me, if I also had to apply at that time, is my daughter my son-in-law she is also a civil servant.

 She works in the internal department. Well, if I were ordinary and I wouldn't want that much, I would apply to the disabled government, but I was just getting right, I didn't do that. Well, even a country is not very easy either. You see, the international situation is not good, and this country costs a lot of money.
 A lot of money, if you otherwise, if the international situation is good, we can improve a little, but no way, the world is like this.
 Ask: ah, big ye, you are what work do? I think you understand the government that way,
Answer: I farm by land, farm by oneself.
 Q: Yeah, what did you don't do when you came here in' 97?

 Answer: After doing a small business, take grandchildren.
 Q: Then you just said that you just said that they are still good. But do you feel that from your own point of view, do you as an old person, do you think they can do a little better? To make you even happier? Is there any advice from here?

 A: That's not so demanding. Ok, Ok, thank you for working hard

Q: How old are you aged now?
 A: 86 years old.
 Q: Are you a local person?
 Answer: My hometown is in Chaoshan. I visited Shenzhen 6 years ago, and now I live in Dongguan. He had three sons and a daughter, and the sons did business, so they moved over with him.
 Q: You look so healthy.
 A: Yes, I always get sick,
Q: But you look very energetic. What are the main diseases?
 A: This heart disease. Heart at first the body is still very good, 69 years old that year car accident ah, originally I was often often have exercise ah, this his foot was broken, just stay at home for a year. I started having a bad heart at the end of my 70s. Well, the heart is not good. I keep taking that heart medicine. Well. Just eat about four or five years, four or five years later began that stomach is bad, gastric ulcer, slowly chronic gastritis erosion. After this heart also this to this foot influence, began the heart is not good. The heart is not good to take medicine for a long time, the stomach is bad.be not allowed. Now you can't even take that cerebrovascular, nor is the arteriosclerosis.
 Ask: oh, this way, that this is really multiple serious diseases, that you these diseases will usually affect your life a lot, is not you usually affect your own life, or mood ah, family relations, old body health can body body is not healthy trouble. Well, then you think you now the whole of your own life, because these diseases will be your life is generally satisfied?
 A: Generally speaking, it is still ok, right?
 Q: that you and the children their family relationship, and neighbor relationship these all very good?
 A: Generally,
Q: Not so much, right? How you have your children. How many children are there?
 A: Three boys and a girl.
 Q: Oh, four children. Are you a local person or a nonlocal person?
 Answer: I chaoshan come over, how long did you come over? I started working out in Shenzhen when I was 50.
 Q: I came here in 1997. I have been here for a long time. There are still more than ten days, more than 20 years away.
 Q: Well. Oh, if you come here, will you come here for your own business, or.
 A: My son did a little business.
 Q: So you came here with your son, so you came along with you?
 A: Well.
 Q: Oh, so, that's like most of the family population is here,
A: Yes, son, what daughter-in-law, grandchildren, and these are all here.
 Q: What do you think is the happiest thing in your life now is what you are most satisfied with. What are you most satisfied with in your whole state of life?
 Answer: satisfaction is this has children to have a family, have grandson. That grandson even graduated from college and came out to work.

 Q: Generally speaking, I am quite satisfied with my own feelings and my life. Still satisfied? Or not how satisfied?
 A: Satisfied.
 Q: Do you think you are living with your children or not right now?
 A: Live together.
 Q: That means that you think you live now and you say you live with your children, right?
 A: Yes.
 Q: If you live together, do you think it is quite different to live together with the young people? Or do you prefer to live alone or prefer to live with your children to answer: ah. No, no, then you are all the young people are very filial piety.

 Q: Oh, that's good, because many young people are not used to living with the old people. Generally speaking, you feel very comfortable living with them, don't they?
 Answer: That is still very filial piety.
 Q: Are you usually sick, they will accompany you to see a doctor. Will come with you, won't you? For example, what you usually take medicine, they will help you to pay attention to ah or how
A: Yes, yes.
 Q: Ok, that means you think you mean you are generally satisfied, but do you really think about it? Well, from the community, or the government, or from the community or other aspects, you can make your life a little better and happier. Do you have any good advice? What can we do? From the community, ah, or from this medical staff, ah, or from the government, ah, what do you think we can do better. It can improve your satisfaction, improve your satisfaction, improve your life happiness, then you are happier.

 Answer: that generally basically in that community now, that service also generally still can ah.
 Q: Generally speaking, do you think this kind of service in their community is ok in all aspects. Does the government have any care or special care for your elderly people?
 A: Yes, yes, yes, yes,
Q: What are the benefits.

 A: For example, it was in the countryside, ah ah, I live at the age of MAO Zedong era, it was very hard at that time. Yes, yes, that substance is very good, and you will get bored. Yes, that's different now. Since I have been in the countryside since I was 65 years ago, then the government has taken care of the old man. At first, it is 50 yuan, 50 yuan a month after 55 yuan, slowly lift, slowly lift, until now there are more than 200 points.
 Q: Every month, right?
 A: Yes
Q: every month you although you this number in the outside this situation is that proportion is very small, right? But you have so much money so much old people calculate that amount is very powerful. Then you quite understand the government.
 A: Well, yes.
 Q: Do they give you, for example, the usual care is if the community pays you if the government gives you some other benefits. Ask you how well you are. That means to care about you or to check for you. You're not having anything else.

 Answer: it is a family like me, if I also had to apply at that time, is my daughter my son-in-law she is also a civil servant.

 She works in the internal department. Well, if I were ordinary and I wouldn't want that much, I would apply to the disabled government, but I was just getting right, I didn't do that. Well, even a country is not very easy either. You see, the international situation is not good, and this country costs a lot of money.
 A lot of money, if you otherwise, if the international situation is good, we can improve a little, but no way, the world is like this.
 Ask: ah, big ye, you are what work do? I think you understand the government that way,
Answer: I farm by land, farm by oneself.
 Q: Yeah, what did you don't do when you came here in' 97?

 Answer: After doing a small business, take grandchildren.
 Q: Then you just said that you just said that they are still good. But do you feel that from your own point of view, do you as an old person, do you think they can do a little better? To make you even happier? Is there any advice from here?

 A: That's not so demanding. Ok, Ok, thank you for working hard

Q: How old are you aged now?
 A: 87 years old.
 Q: Are you a local person?
 Answer: My hometown is in Chaoshan. I visited Shenzhen 7 years ago, and now I live in Dongguan. He had three sons and a daughter, and the sons did business, so they moved over with him.
 Q: You look so healthy.
 A: Yes, I always get sick,
Q: But you look very energetic. What are the main diseases?
 A: This heart disease. Heart at first the body is still very good, 70 years old that year car accident, originally I was often often have exercise, this his foot broke, just stay at home for a year. I started having a bad heart at the end of age 71. Well, the heart is not good. I keep taking that heart medicine. Well. Just eat about four or five years, four or five years later began that stomach is bad, gastric ulcer, slowly chronic gastritis erosion. After this heart also this to this foot influence, began the heart is not good. The heart is not good to take medicine for a long time, the stomach is bad.be not allowed. Now you can't even take that cerebrovascular, nor is the arteriosclerosis.
 Ask: oh, this way, that this is really multiple serious diseases, that you these diseases will usually affect your life a lot, is not you usually affect your own life, or mood ah, family relations, old body health can body body is not healthy trouble. Well, then you think you now the whole of your own life, because these diseases will be your life is generally satisfied?
 A: Generally speaking, it is still ok, right?
 Q: that you and the children their family relationship, and neighbor relationship these all very good?
 A: Generally,
Q: Not so much, right? How you have your children. How many children are there?
 A: Three boys and a girl.
 Q: Oh, four children. Are you a local person or a nonlocal person?
 Answer: I chaoshan come over, how long did you come over? I started working out in Shenzhen when I was 50.
 Q: I came here in 1997. I have been here for a long time. There are still more than ten days, more than 20 years away.
 Q: Well. Oh, if you come here, will you come here for your own business, or.
 A: My son did a little business.
 Q: So you came here with your son, so you came along with you?
 A: Well.
 Q: Oh, so, that's like most of the family population is here,
A: Yes, son, what daughter-in-law, grandchildren, and these are all here.
 Q: What do you think is the happiest thing in your life now is what you are most satisfied with. What are you most satisfied with in your whole state of life?
 Answer: satisfaction is this has children to have a family, have grandson. That grandson even graduated from college and came out to work.

 Q: Generally speaking, I am quite satisfied with my own feelings and my life. Still satisfied? Or not how satisfied?
 A: Satisfied.
 Q: Do you think you are living with your children or not right now?
 A: Live together.
 Q: That means that you think you live now and you say you live with your children, right?
 A: Yes.
 Q: If you live together, do you think it is quite different to live together with the young people? Or do you prefer to live alone or prefer to live with your children to answer: ah. No, no, then you are all the young people are very filial piety.

 Q: Oh, that's good, because many young people are not used to living with the old people. Generally speaking, you feel very comfortable living with them, don't they?
 Answer: That is still very filial piety.
 Q: Are you usually sick, they will accompany you to see a doctor. Will come with you, won't you? For example, what you usually take medicine, they will help you to pay attention to ah or how
A: Yes, yes.
 Q: Ok, that means you think you mean you are generally satisfied, but do you really think about it? Well, from the community, or the government, or from the community or other aspects, you can make your life a little better and happier. Do you have any good advice? What can we do? From the community, ah, or from this medical staff, ah, or from the government, ah, what do you think we can do better. It can improve your satisfaction, improve your satisfaction, improve your life happiness, then you are happier.

 Answer: that generally basically in that community now, that service also generally still can ah.
 Q: Generally speaking, do you think this kind of service in their community is ok in all aspects. Does the government have any care or special care for your elderly people?
 A: Yes, yes, yes, yes,
Q: What are the benefits.

 A: For example, it was in the countryside, ah ah, I live at the age of MAO Zedong era, it was very hard at that time. Yes, yes, that substance is very good, and you will get bored. Yes, that's different now. Since I have been in the countryside since I was 65 years ago, then the government has taken care of the old man. At first, it is 50 yuan, 50 yuan a month after 55 yuan, slowly lift, slowly lift, until now there are more than 200 points.
 Q: Every month, right?
 A: Yes
Q: every month you although you this number in the outside this situation is that proportion is very small, right? But you have so much money so much old people calculate that amount is very powerful. Then you quite understand the government.
 A: Well, yes.
 Q: Do they give you, for example, the usual care is if the community pays you if the government gives you some other benefits. Ask you how well you are. That means to care about you or to check for you. You're not having anything else.

 Answer: it is a family like me, if I also had to apply at that time, is my daughter my son-in-law she is also a civil servant.

 She works in the internal department. Well, if I were ordinary and I wouldn't want that much, I would apply to the disabled government, but I was just getting right, I didn't do that. Well, even a country is not very easy either. You see, the international situation is not good, and this country costs a lot of money.
 A lot of money, if you otherwise, if the international situation is good, we can improve a little, but no way, the world is like this.
 Ask: ah, big ye, you are what work do? I think you understand the government that way,
Answer: I farm by land, farm by oneself.
 Q: Yeah, what did you don't do when you came here in' 97?

 Answer: After doing a small business, take grandchildren.
 Q: Then you just said that you just said that they are still good. But do you feel that from your own point of view, do you as an old person, do you think they can do a little better? To make you even happier? Is there any advice from here?

 A: That's not so demanding. Ok, Ok, thank you for working hard

Q: How old are you aged now?
 A: 88 years old.
 Q: Are you a local person?
 A: My hometown is in Chaoshan. I visited Shenzhen 8 years ago, and now I live in Dongguan. He had three sons and a daughter, and the sons did business, so they moved over with him.
 Q: You look so healthy.
 A: Yes, I always get sick,
Q: But you look very energetic. What are the main diseases?
 A: This heart disease. Heart at first the body is still very good, 71 years old that year car accident ah, originally I was often often have exercise ah, this his foot broke, just stay at home for a year. He started having a bad heart at the end of age 72. Well, the heart is not good. I keep taking that heart medicine. Well. Just eat about four or five years, four or five years later began that stomach is bad, gastric ulcer, slowly chronic gastritis erosion. After this heart also this to this foot influence, began the heart is not good. The heart is not good to take medicine for a long time, the stomach is bad.be not allowed. Now you can't even take that cerebrovascular, nor is the arteriosclerosis.
 Ask: oh, this way, that this is really multiple serious diseases, that you these diseases will usually affect your life a lot, is not you usually affect your own life, or mood ah, family relations, old body health can body body is not healthy trouble. Well, then you think you now the whole of your own life, because these diseases will be your life is generally satisfied?
 A: Generally speaking, it is still ok, right?
 Q: that you and the children their family relationship, and neighbor relationship these all very good?
 A: Generally,
Q: Not so much, right? How you have your children. How many children are there?
 A: Three boys and a girl.
 Q: Oh, four children. Are you a local person or a nonlocal person?
 Answer: I chaoshan come over, how long did you come over? I started working out in Shenzhen when I was 50.
 Q: I came here in 1997. I have been here for a long time. There are still more than ten days, more than 20 years away.
 Q: Well. Oh, if you come here, will you come here for your own business, or.
 A: My son did a little business.
 Q: So you came here with your son, so you came along with you?
 A: Well.
 Q: Oh, so, that's like most of the family population is here,
A: Yes, son, what daughter-in-law, grandchildren, and these are all here.
 Q: What do you think is the happiest thing in your life now is what you are most satisfied with. What are you most satisfied with in your whole state of life?
 Answer: satisfaction is this has children to have a family, have grandson. That grandson even graduated from college and came out to work.

 Q: Generally speaking, I am quite satisfied with my own feelings and my life. Still satisfied? Or not how satisfied?
 A: Satisfied.
 Q: Do you think you are living with your children or not right now?
 A: Live together.
 Q: That means that you think you live now and you say you live with your children, right?
 A: Yes.
 Q: If you live together, do you think it is quite different to live together with the young people? Or do you prefer to live alone or prefer to live with your children to answer: ah. No, no, then you are all the young people are very filial piety.

 Q: Oh, that's good, because many young people are not used to living with the old people. Generally speaking, you feel very comfortable living with them, don't they?
 Answer: That is still very filial piety.
 Q: Are you usually sick, they will accompany you to see a doctor. Will come with you, won't you? For example, what you usually take medicine, they will help you to pay attention to ah or how
A: Yes, yes.
 Q: Ok, that means you think you mean you are generally satisfied, but do you really think about it? Well, from the community, or the government, or from the community or other aspects, you can make your life a little better and happier. Do you have any good advice? What can we do? From the community, ah, or from this medical staff, ah, or from the government, ah, what do you think we can do better. It can improve your satisfaction, improve your satisfaction, improve your life happiness, then you are happier.

 Answer: that generally basically in that community now, that service also generally still can ah.
 Q: Generally speaking, do you think this kind of service in their community is ok in all aspects. Does the government have any care or special care for your elderly people?
 A: Yes, yes, yes, yes,
Q: What are the benefits.

 A: For example, it was in the countryside, ah ah, I live at the age of MAO Zedong era, it was very hard at that time. Yes, yes, that substance is very good, and you will get bored. Yes, that's different now. Since I have been in the countryside since I was 65 years ago, then the government has taken care of the old man. At first, it is 50 yuan, 50 yuan a month after 55 yuan, slowly lift, slowly lift, until now there are more than 200 points.
 Q: Every month, right?
 A: Yes
Q: every month you although you this number in the outside this situation is that proportion is very small, right? But you have so much money so much old people calculate that amount is very powerful. Then you quite understand the government.
 A: Well, yes.
 Q: Do they give you, for example, the usual care is if the community pays you if the government gives you some other benefits. Ask you how well you are. That means to care about you or to check for you. You're not having anything else.

 Answer: it is a family like me, if I also had to apply at that time, is my daughter my son-in-law she is also a civil servant.

 She works in the internal department. Well, if I were ordinary and I wouldn't want that much, I would apply to the disabled government, but I was just getting right, I didn't do that. Well, even a country is not very easy either. You see, the international situation is not good, and this country costs a lot of money.
 A lot of money, if you otherwise, if the international situation is good, we can improve a little, but no way, the world is like this.
 Ask: ah, big ye, you are what work do? I think you understand the government that way,
Answer: I farm by land, farm by oneself.
 Q: Yeah, what did you don't do when you came here in' 97?

 Answer: After doing a small business, take grandchildren.
 Q: Then you just said that you just said that they are still good. But do you feel that from your own point of view, do you as an old person, do you think they can do a little better? To make you even happier? Is there any advice from here?

 A: That's not so demanding. Ok, Ok, thank you for working hard

Q: How old are you aged now?
 A: 89 years old.
 Q: Are you a local person?
 A: My hometown is in Chaoshan. I visited Shenzhen 9 years ago, and now I live in Dongguan. He had three sons and a daughter, and the sons did business, so they moved over with him.
 Q: You look so healthy.
 A: Yes, I always get sick,
Q: But you look very energetic. What are the main diseases?
 A: This heart disease. Heart at first the body is very good, 72 years old that year car accident, originally I was often often have exercise, this his foot broken, just stay at home for a year. He started having a bad heart at the end of age 73. Well, the heart is not good. I keep taking that heart medicine. Well. Just eat about four or five years, four or five years later began that stomach is bad, gastric ulcer, slowly chronic gastritis erosion. After this heart also this to this foot influence, began the heart is not good. The heart is not good to take medicine for a long time, the stomach is bad.be not allowed. Now you can't even take that cerebrovascular, nor is the arteriosclerosis.
 Ask: oh, this way, that this is really multiple serious diseases, that you these diseases will usually affect your life a lot, is not you usually affect your own life, or mood ah, family relations, old body health can body body is not healthy trouble. Well, then you think you now the whole of your own life, because these diseases will be your life is generally satisfied?
 A: Generally speaking, it is still ok, right?
 Q: that you and the children their family relationship, and neighbor relationship these all very good?
 A: Generally,
Q: Not so much, right? How you have your children. How many children are there?
 A: Three boys and a girl.
 Q: Oh, four children. Are you a local person or a nonlocal person?
 Answer: I chaoshan come over, how long did you come over? I started working out in Shenzhen when I was 50.
 Q: I came here in 1997. I have been here for a long time. There are still more than ten days, more than 20 years away.
 Q: Well. Oh, if you come here, will you come here for your own business, or.
 A: My son did a little business.
 Q: So you came here with your son, so you came along with you?
 A: Well.
 Q: Oh, so, that's like most of the family population is here,
A: Yes, son, what daughter-in-law, grandchildren, and these are all here.
 Q: What do you think is the happiest thing in your life now is what you are most satisfied with. What are you most satisfied with in your whole state of life?
 Answer: satisfaction is this has children to have a family, have grandson. That grandson even graduated from college and came out to work.

 Q: Generally speaking, I am quite satisfied with my own feelings and my life. Still satisfied? Or not how satisfied?
 A: Satisfied.
 Q: Do you think you are living with your children or not right now?
 A: Live together.
 Q: That means that you think you live now and you say you live with your children, right?
 A: Yes.
 Q: If you live together, do you think it is quite different to live together with the young people? Or do you prefer to live alone or prefer to live with your children to answer: ah. No, no, then you are all the young people are very filial piety.

 Q: Oh, that's good, because many young people are not used to living with the old people. Generally speaking, you feel very comfortable living with them, don't they?
 Answer: That is still very filial piety.
 Q: Are you usually sick, they will accompany you to see a doctor. Will come with you, won't you? For example, what you usually take medicine, they will help you to pay attention to ah or how
A: Yes, yes.
 Q: Ok, that means you think you mean you are generally satisfied, but do you really think about it? Well, from the community, or the government, or from the community or other aspects, you can make your life a little better and happier. Do you have any good advice? What can we do? From the community, ah, or from this medical staff, ah, or from the government, ah, what do you think we can do better. It can improve your satisfaction, improve your satisfaction, improve your life happiness, then you are happier.

 Answer: that generally basically in that community now, that service also generally still can ah.
 Q: Generally speaking, do you think this kind of service in their community is ok in all aspects. Does the government have any care or special care for your elderly people?
 A: Yes, yes, yes, yes,
Q: What are the benefits.

 A: For example, it was in the countryside, ah ah, I live at the age of MAO Zedong era, it was very hard at that time. Yes, yes, that substance is very good, and you will get bored. Yes, that's different now. Since I have been in the countryside since I was 65 years ago, then the government has taken care of the old man. At first, it is 50 yuan, 50 yuan a month after 55 yuan, slowly lift, slowly lift, until now there are more than 200 points.
 Q: Every month, right?
 A: Yes
Q: every month you although you this number in the outside this situation is that proportion is very small, right? But you have so much money so much old people calculate that amount is very powerful. Then you quite understand the government.
 A: Well, yes.
 Q: Do they give you, for example, the usual care is if the community pays you if the government gives you some other benefits. Ask you how well you are. That means to care about you or to check for you. You're not having anything else.

 Answer: it is a family like me, if I also had to apply at that time, is my daughter my son-in-law she is also a civil servant.

 She works in the internal department. Well, if I were ordinary and I wouldn't want that much, I would apply to the disabled government, but I was just getting right, I didn't do that. Well, even a country is not very easy either. You see, the international situation is not good, and this country costs a lot of money.
 A lot of money, if you otherwise, if the international situation is good, we can improve a little, but no way, the world is like this.
 Ask: ah, big ye, you are what work do? I think you understand the government that way,
Answer: I farm by land, farm by oneself.
 Q: Yeah, what did you don't do when you came here in' 97?

 Answer: After doing a small business, take grandchildren.
 Q: Then you just said that you just said that they are still good. But do you feel that from your own point of view, do you as an old person, do you think they can do a little better? To make you even happier? Is there any advice from here?

 A: That's not so demanding. Ok, Ok, thank you for working hard

Q: How old are you aged now?
 A: 90 years old.
 Q: Are you a local person?
 A: My hometown is in Chaoshan. I came to Shenzhen 10 years ago, and now I live in Dongguan. He had three sons and a daughter, and the sons did business, so they moved over with him.
 Q: You look so healthy.
 A: Yes, I always get sick,
Q: But you look very energetic. What are the main diseases?
 A: This heart disease. Heart at first the body is still very good, 73 years old that year car accident, originally I was often often have exercise, this his foot was broken, just stay at home for a year. He started having a bad heart at the end of age 74. Well, the heart is not good. I keep taking that heart medicine. Well. Just eat about four or five years, four or five years later began that stomach is bad, gastric ulcer, slowly chronic gastritis erosion. After this heart also this to this foot influence, began the heart is not good. The heart is not good to take medicine for a long time, the stomach is bad.be not allowed. Now you can't even take that cerebrovascular, nor is the arteriosclerosis.
 Ask: oh, this way, that this is really multiple serious diseases, that you these diseases will usually affect your life a lot, is not you usually affect your own life, or mood ah, family relations, old body health can body body is not healthy trouble. Well, then you think you now the whole of your own life, because these diseases will be your life is generally satisfied?
 A: Generally speaking, it is still ok, right?
 Q: that you and the children their family relationship, and neighbor relationship these all very good?
 A: Generally,
Q: Not so much, right? How you have your children. How many children are there?
 A: Three boys and a girl.
 Q: Oh, four children. Are you a local person or a nonlocal person?
 Answer: I chaoshan come over, how long did you come over? I started working out in Shenzhen when I was 50.
 Q: I came here in 1997. I have been here for a long time. There are still more than ten days, more than 20 years away.
 Q: Well. Oh, if you come here, will you come here for your own business, or.
 A: My son did a little business.
 Q: So you came here with your son, so you came along with you?
 A: Well.
 Q: Oh, so, that's like most of the family population is here,
A: Yes, son, what daughter-in-law, grandchildren, and these are all here.
 Q: What do you think is the happiest thing in your life now is what you are most satisfied with. What are you most satisfied with in your whole state of life?
 Answer: satisfaction is this has children to have a family, have grandson. That grandson even graduated from college and came out to work.

 Q: Generally speaking, I am quite satisfied with my own feelings and my life. Still satisfied? Or not how satisfied?
 A: Satisfied.
 Q: Do you think you are living with your children or not right now?
 A: Live together.
 Q: That means that you think you live now and you say you live with your children, right?
 A: Yes.
 Q: If you live together, do you think it is quite different to live together with the young people? Or do you prefer to live alone or prefer to live with your children to answer: ah. No, no, then you are all the young people are very filial piety.

 Q: Oh, that's good, because many young people are not used to living with the old people. Generally speaking, you feel very comfortable living with them, don't they?
 Answer: That is still very filial piety.
 Q: Are you usually sick, they will accompany you to see a doctor. Will come with you, won't you? For example, what you usually take medicine, they will help you to pay attention to ah or how
A: Yes, yes.
 Q: Ok, that means you think you mean you are generally satisfied, but do you really think about it? Well, from the community, or the government, or from the community or other aspects, you can make your life a little better and happier. Do you have any good advice? What can we do? From the community, ah, or from this medical staff, ah, or from the government, ah, what do you think we can do better. It can improve your satisfaction, improve your satisfaction, improve your life happiness, then you are happier.

 Answer: that generally basically in that community now, that service also generally still can ah.
 Q: Generally speaking, do you think this kind of service in their community is ok in all aspects. Does the government have any care or special care for your elderly people?
 A: Yes, yes, yes, yes,
Q: What are the benefits.

 A: For example, it was in the countryside, ah ah, I live at the age of MAO Zedong era, it was very hard at that time. Yes, yes, that substance is very good, and you will get bored. Yes, that's different now. Since I have been in the countryside since I was 65 years ago, then the government has taken care of the old man. At first, it is 50 yuan, 50 yuan a month after 55 yuan, slowly lift, slowly lift, until now there are more than 200 points.
 Q: Every month, right?
 A: Yes
Q: every month you although you this number in the outside this situation is that proportion is very small, right? But you have so much money so much old people calculate that amount is very powerful. Then you quite understand the government.
 A: Well, yes.
 Q: Do they give you, for example, the usual care is if the community pays you if the government gives you some other benefits. Ask you how well you are. That means to care about you or to check for you. You're not having anything else.

 Answer: it is a family like me, if I also had to apply at that time, is my daughter my son-in-law she is also a civil servant.

 She works in the internal department. Well, if I were ordinary and I wouldn't want that much, I would apply to the disabled government, but I was just getting right, I didn't do that. Well, even a country is not very easy either. You see, the international situation is not good, and this country costs a lot of money.
 A lot of money, if you otherwise, if the international situation is good, we can improve a little, but no way, the world is like this.
 Ask: ah, big ye, you are what work do? I think you understand the government that way,
Answer: I farm by land, farm by oneself.
 Q: Yeah, what did you don't do when you came here in' 97?

 Answer: After doing a small business, take grandchildren.
 Q: Then you just said that you just said that they are still good. But do you feel that from your own point of view, do you as an old person, do you think they can do a little better? To make you even happier? Is there any advice from here?

 A: That's not so demanding. Ok, Ok, thank you for working hard

Q: How old are you aged now?
 A: 81 years old.
 Q: Are you a local person?
 Answer: My hometown is in Chaoshan. I visited Shenzhen 6 years ago, and now I live in Dongguan. He had three sons and a daughter, and the sons did business, so they moved over with him.
 Q: You look so healthy.
 A: Yes, I always get sick,
Q: But you look very energetic. What are the main diseases?
 A: This heart disease. Heart at first the body is still very good, 69 years old that year car accident ah, originally I was often often have exercise ah, this his foot was broken, just stay at home for a year. I started having a bad heart at the end of my 70s. Well, the heart is not good. I keep taking that heart medicine. Well. Just eat about four or five years, four or five years later began that stomach is bad, gastric ulcer, slowly chronic gastritis erosion. After this heart also this to this foot influence, began the heart is not good. The heart is not good to take medicine for a long time, the stomach is bad.be not allowed. Now you can't even take that cerebrovascular, nor is the arteriosclerosis.
 Ask: oh, this way, that this is really multiple serious diseases, that you these diseases will usually affect your life a lot, is not you usually affect your own life, or mood ah, family relations, old body health can body body is not healthy trouble. Well, then you think you now the whole of your own life, because these diseases will be your life is generally satisfied?
 A: Generally speaking, it is still ok, right?
 Q: that you and the children their family relationship, and neighbor relationship these all very good?
 A: Generally,
Q: Not so much, right? How you have your children. How many children are there?
 A: Three boys and a girl.
 Q: Oh, four children. Are you a local person or a nonlocal person?
 Answer: I chaoshan come over, how long did you come over? I started working out in Shenzhen when I was 50.
 Q: I came here in 1997. I have been here for a long time. There are still more than ten days, more than 20 years away.
 Q: Well. Oh, if you come here, will you come here for your own business, or.
 A: My son did a little business.
 Q: So you came here with your son, so you came along with you?
 A: Well.
 Q: Oh, so, that's like most of the family population is here,
A: Yes, son, what daughter-in-law, grandchildren, and these are all here.
 Q: What do you think is the happiest thing in your life now is what you are most satisfied with. What are you most satisfied with in your whole state of life?
 Answer: satisfaction is this has children to have a family, have grandson. That grandson even graduated from college and came out to work.

 Q: Generally speaking, I am quite satisfied with my own feelings and my life. Still satisfied? Or not how satisfied?
 A: Satisfied.
 Q: Do you think you are living with your children or not right now?
 A: Live together.
 Q: That means that you think you live now and you say you live with your children, right?
 A: Yes.
 Q: If you live together, do you think it is quite different to live together with the young people? Or do you prefer to live alone or prefer to live with your children to answer: ah. No, no, then you are all the young people are very filial piety.

 Q: Oh, that's good, because many young people are not used to living with the old people. Generally speaking, you feel very comfortable living with them, don't they?
 Answer: That is still very filial piety.
 Q: Are you usually sick, they will accompany you to see a doctor. Will come with you, won't you? For example, what you usually take medicine, they will help you to pay attention to ah or how
A: Yes, yes.
 Q: Ok, that means you think you mean you are generally satisfied, but do you really think about it? Well, from the community, or the government, or from the community or other aspects, you can make your life a little better and happier. Do you have any good advice? What can we do? From the community, ah, or from this medical staff, ah, or from the government, ah, what do you think we can do better. It can improve your satisfaction, improve your satisfaction, improve your life happiness, then you are happier.

 Answer: that generally basically in that community now, that service also generally still can ah.
 Q: Generally speaking, do you think this kind of service in their community is ok in all aspects. Does the government have any care or special care for your elderly people?
 A: Yes, yes, yes, yes,
Q: What are the benefits.

 A: For example, it was in the countryside, ah ah, I live at the age of MAO Zedong era, it was very hard at that time. Yes, yes, that substance is very good, and you will get bored. Yes, that's different now. Since I have been in the countryside since I was 65 years ago, then the government has taken care of the old man. At first, it is 50 yuan, 50 yuan a month after 55 yuan, slowly lift, slowly lift, until now there are more than 200 points.
 Q: Every month, right?
 A: Yes
Q: every month you although you this number in the outside this situation is that proportion is very small, right? But you have so much money so much old people calculate that amount is very powerful. Then you quite understand the government.
 A: Well, yes.
 Q: Do they give you, for example, the usual care is if the community pays you if the government gives you some other benefits. Ask you how well you are. That means to care about you or to check for you. You're not having anything else.

 Answer: it is a family like me, if I also had to apply at that time, is my daughter my son-in-law she is also a civil servant.

 She works in the internal department. Well, if I were ordinary and I wouldn't want that much, I would apply to the disabled government, but I was just getting right, I didn't do that. Well, even a country is not very easy either. You see, the international situation is not good, and this country costs a lot of money.
 A lot of money, if you otherwise, if the international situation is good, we can improve a little, but no way, the world is like this.
 Ask: ah, big ye, you are what work do? I think you understand the government that way,
Answer: I farm by land, farm by oneself.
 Q: Yeah, what did you don't do when you came here in' 97?

 Answer: After doing a small business, take grandchildren.
 Q: Then you just said that you just said that they are still good. But do you feel that from your own point of view, do you as an old person, do you think they can do a little better? To make you even happier? Is there any advice from here?

 A: That's not so demanding. Ok, Ok, thank you for working hard

Q: How old are you aged now?
 A: 82 years old.
 Q: Are you a local person?
 Answer: My hometown is in Chaoshan. I visited Shenzhen 7 years ago, and now I live in Dongguan. He had three sons and a daughter, and the sons did business, so they moved over with him.
 Q: You look so healthy.
 A: Yes, I always get sick,
Q: But you look very energetic. What are the main diseases?
 A: This heart disease. Heart at first the body is still very good, 70 years old that year car accident, originally I was often often have exercise, this his foot broke, just stay at home for a year. I started having a bad heart at the end of age 71. Well, the heart is not good. I keep taking that heart medicine. Well. Just eat about four or five years, four or five years later began that stomach is bad, gastric ulcer, slowly chronic gastritis erosion. After this heart also this to this foot influence, began the heart is not good. The heart is not good to take medicine for a long time, the stomach is bad.be not allowed. Now you can't even take that cerebrovascular, nor is the arteriosclerosis.
 Ask: oh, this way, that this is really multiple serious diseases, that you these diseases will usually affect your life a lot, is not you usually affect your own life, or mood ah, family relations, old body health can body body is not healthy trouble. Well, then you think you now the whole of your own life, because these diseases will be your life is generally satisfied?
 A: Generally speaking, it is still ok, right?
 Q: that you and the children their family relationship, and neighbor relationship these all very good?
 A: Generally,
Q: Not so much, right? How you have your children. How many children are there?
 A: Three boys and a girl.
 Q: Oh, four children. Are you a local person or a nonlocal person?
 Answer: I chaoshan come over, how long did you come over? I started working out in Shenzhen when I was 50.
 Q: I came here in 1997. I have been here for a long time. There are still more than ten days, more than 20 years away.
 Q: Well. Oh, if you come here, will you come here for your own business, or.
 A: My son did a little business.
 Q: So you came here with your son, so you came along with you?
 A: Well.
 Q: Oh, so, that's like most of the family population is here,
A: Yes, son, what daughter-in-law, grandchildren, and these are all here.
 Q: What do you think is the happiest thing in your life now is what you are most satisfied with. What are you most satisfied with in your whole state of life?
 Answer: satisfaction is this has children to have a family, have grandson. That grandson even graduated from college and came out to work.

 Q: Generally speaking, I am quite satisfied with my own feelings and my life. Still satisfied? Or not how satisfied?
 A: Satisfied.
 Q: Do you think you are living with your children or not right now?
 A: Live together.
 Q: That means that you think you live now and you say you live with your children, right?
 A: Yes.
 Q: If you live together, do you think it is quite different to live together with the young people? Or do you prefer to live alone or prefer to live with your children to answer: ah. No, no, then you are all the young people are very filial piety.

 Q: Oh, that's good, because many young people are not used to living with the old people. Generally speaking, you feel very comfortable living with them, don't they?
 Answer: That is still very filial piety.
 Q: Are you usually sick, they will accompany you to see a doctor. Will come with you, won't you? For example, what you usually take medicine, they will help you to pay attention to ah or how
A: Yes, yes.
 Q: Ok, that means you think you mean you are generally satisfied, but do you really think about it? Well, from the community, or the government, or from the community or other aspects, you can make your life a little better and happier. Do you have any good advice? What can we do? From the community, ah, or from this medical staff, ah, or from the government, ah, what do you think we can do better. It can improve your satisfaction, improve your satisfaction, improve your life happiness, then you are happier.

 Answer: that generally basically in that community now, that service also generally still can ah.
 Q: Generally speaking, do you think this kind of service in their community is ok in all aspects. Does the government have any care or special care for your elderly people?
 A: Yes, yes, yes, yes,
Q: What are the benefits.

 A: For example, it was in the countryside, ah ah, I live at the age of MAO Zedong era, it was very hard at that time. Yes, yes, that substance is very good, and you will get bored. Yes, that's different now. Since I have been in the countryside since I was 65 years ago, then the government has taken care of the old man. At first, it is 50 yuan, 50 yuan a month after 55 yuan, slowly lift, slowly lift, until now there are more than 200 points.
 Q: Every month, right?
 A: Yes
Q: every month you although you this number in the outside this situation is that proportion is very small, right? But you have so much money so much old people calculate that amount is very powerful. Then you quite understand the government.
 A: Well, yes.
 Q: Do they give you, for example, the usual care is if the community pays you if the government gives you some other benefits. Ask you how well you are. That means to care about you or to check for you. You're not having anything else.

 Answer: it is a family like me, if I also had to apply at that time, is my daughter my son-in-law she is also a civil servant.

 She works in the internal department. Well, if I were ordinary and I wouldn't want that much, I would apply to the disabled government, but I was just getting right, I didn't do that. Well, even a country is not very easy either. You see, the international situation is not good, and this country costs a lot of money.
 A lot of money, if you otherwise, if the international situation is good, we can improve a little, but no way, the world is like this.
 Ask: ah, big ye, you are what work do? I think you understand the government that way,
Answer: I farm by land, farm by oneself.
 Q: Yeah, what did you don't do when you came here in' 97?

 Answer: After doing a small business, take grandchildren.
 Q: Then you just said that you just said that they are still good. But do you feel that from your own point of view, do you as an old person, do you think they can do a little better? To make you even happier? Is there any advice from here?

 A: That's not so demanding. Ok, Ok, thank you for working hard

Q: How old are you aged now?
 A: 83 years old.
 Q: Are you a local person?
 A: My hometown is in Chaoshan. I visited Shenzhen 8 years ago, and now I live in Dongguan. He had three sons and a daughter, and the sons did business, so they moved over with him.
 Q: You look so healthy.
 A: Yes, I always get sick,
Q: But you look very energetic. What are the main diseases?
 A: This heart disease. Heart at first the body is still very good, 71 years old that year car accident ah, originally I was often often have exercise ah, this his foot broke, just stay at home for a year. He started having a bad heart at the end of age 72. Well, the heart is not good. I keep taking that heart medicine. Well. Just eat about four or five years, four or five years later began that stomach is bad, gastric ulcer, slowly chronic gastritis erosion. After this heart also this to this foot influence, began the heart is not good. The heart is not good to take medicine for a long time, the stomach is bad.be not allowed. Now you can't even take that cerebrovascular, nor is the arteriosclerosis.
 Ask: oh, this way, that this is really multiple serious diseases, that you these diseases will usually affect your life a lot, is not you usually affect your own life, or mood ah, family relations, old body health can body body is not healthy trouble. Well, then you think you now the whole of your own life, because these diseases will be your life is generally satisfied?
 A: Generally speaking, it is still ok, right?
 Q: that you and the children their family relationship, and neighbor relationship these all very good?
 A: Generally,
Q: Not so much, right? How you have your children. How many children are there?
 A: Three boys and a girl.
 Q: Oh, four children. Are you a local person or a nonlocal person?
 Answer: I chaoshan come over, how long did you come over? I started working out in Shenzhen when I was 50.
 Q: I came here in 1997. I have been here for a long time. There are still more than ten days, more than 20 years away.
 Q: Well. Oh, if you come here, will you come here for your own business, or.
 A: My son did a little business.
 Q: So you came here with your son, so you came along with you?
 A: Well.
 Q: Oh, so, that's like most of the family population is here,
A: Yes, son, what daughter-in-law, grandchildren, and these are all here.
 Q: What do you think is the happiest thing in your life now is what you are most satisfied with. What are you most satisfied with in your whole state of life?
 Answer: satisfaction is this has children to have a family, have grandson. That grandson even graduated from college and came out to work.

 Q: Generally speaking, I am quite satisfied with my own feelings and my life. Still satisfied? Or not how satisfied?
 A: Satisfied.
 Q: Do you think you are living with your children or not right now?
 A: Live together.
 Q: That means that you think you live now and you say you live with your children, right?
 A: Yes.
 Q: If you live together, do you think it is quite different to live together with the young people? Or do you prefer to live alone or prefer to live with your children to answer: ah. No, no, then you are all the young people are very filial piety.

 Q: Oh, that's good, because many young people are not used to living with the old people. Generally speaking, you feel very comfortable living with them, don't they?
 Answer: That is still very filial piety.
 Q: Are you usually sick, they will accompany you to see a doctor. Will come with you, won't you? For example, what you usually take medicine, they will help you to pay attention to ah or how
A: Yes, yes.
 Q: Ok, that means you think you mean you are generally satisfied, but do you really think about it? Well, from the community, or the government, or from the community or other aspects, you can make your life a little better and happier. Do you have any good advice? What can we do? From the community, ah, or from this medical staff, ah, or from the government, ah, what do you think we can do better. It can improve your satisfaction, improve your satisfaction, improve your life happiness, then you are happier.

 Answer: that generally basically in that community now, that service also generally still can ah.
 Q: Generally speaking, do you think this kind of service in their community is ok in all aspects. Does the government have any care or special care for your elderly people?
 A: Yes, yes, yes, yes,
Q: What are the benefits.

 A: For example, it was in the countryside, ah ah, I live at the age of MAO Zedong era, it was very hard at that time. Yes, yes, that substance is very good, and you will get bored. Yes, that's different now. Since I have been in the countryside since I was 65 years ago, then the government has taken care of the old man. At first, it is 50 yuan, 50 yuan a month after 55 yuan, slowly lift, slowly lift, until now there are more than 200 points.
 Q: Every month, right?
 A: Yes
Q: every month you although you this number in the outside this situation is that proportion is very small, right? But you have so much money so much old people calculate that amount is very powerful. Then you quite understand the government.
 A: Well, yes.
 Q: Do they give you, for example, the usual care is if the community pays you if the government gives you some other benefits. Ask you how well you are. That means to care about you or to check for you. You're not having anything else.

 Answer: it is a family like me, if I also had to apply at that time, is my daughter my son-in-law she is also a civil servant.

 She works in the internal department. Well, if I were ordinary and I wouldn't want that much, I would apply to the disabled government, but I was just getting right, I didn't do that. Well, even a country is not very easy either. You see, the international situation is not good, and this country costs a lot of money.
 A lot of money, if you otherwise, if the international situation is good, we can improve a little, but no way, the world is like this.
 Ask: ah, big ye, you are what work do? I think you understand the government that way,
Answer: I farm by land, farm by oneself.
 Q: Yeah, what did you don't do when you came here in' 97?

 Answer: After doing a small business, take grandchildren.
 Q: Then you just said that you just said that they are still good. But do you feel that from your own point of view, do you as an old person, do you think they can do a little better? To make you even happier? Is there any advice from here?

 A: That's not so demanding. Ok, Ok, thank you for working hard

Q: How old are you aged now?
 A: 84 years old.
 Q: Are you a local person?
 A: My hometown is in Chaoshan. I visited Shenzhen 9 years ago, and now I live in Dongguan. He had three sons and a daughter, and the sons did business, so they moved over with him.
 Q: You look so healthy.
 A: Yes, I always get sick,
Q: But you look very energetic. What are the main diseases?
 A: This heart disease. Heart at first the body is very good, 72 years old that year car accident, originally I was often often have exercise, this his foot broken, just stay at home for a year. He started having a bad heart at the end of age 73. Well, the heart is not good. I keep taking that heart medicine. Well. Just eat about four or five years, four or five years later began that stomach is bad, gastric ulcer, slowly chronic gastritis erosion. After this heart also this to this foot influence, began the heart is not good. The heart is not good to take medicine for a long time, the stomach is bad.be not allowed. Now you can't even take that cerebrovascular, nor is the arteriosclerosis.
 Ask: oh, this way, that this is really multiple serious diseases, that you these diseases will usually affect your life a lot, is not you usually affect your own life, or mood ah, family relations, old body health can body body is not healthy trouble. Well, then you think you now the whole of your own life, because these diseases will be your life is generally satisfied?
 A: Generally speaking, it is still ok, right?
 Q: that you and the children their family relationship, and neighbor relationship these all very good?
 A: Generally,
Q: Not so much, right? How you have your children. How many children are there?
 A: Three boys and a girl.
 Q: Oh, four children. Are you a local person or a nonlocal person?
 Answer: I chaoshan come over, how long did you come over? I started working out in Shenzhen when I was 50.
 Q: I came here in 1997. I have been here for a long time. There are still more than ten days, more than 20 years away.
 Q: Well. Oh, if you come here, will you come here for your own business, or.
 A: My son did a little business.
 Q: So you came here with your son, so you came along with you?
 A: Well.
 Q: Oh, so, that's like most of the family population is here,
A: Yes, son, what daughter-in-law, grandchildren, and these are all here.
 Q: What do you think is the happiest thing in your life now is what you are most satisfied with. What are you most satisfied with in your whole state of life?
 Answer: satisfaction is this has children to have a family, have grandson. That grandson even graduated from college and came out to work.

 Q: Generally speaking, I am quite satisfied with my own feelings and my life. Still satisfied? Or not how satisfied?
 A: Satisfied.
 Q: Do you think you are living with your children or not right now?
 A: Live together.
 Q: That means that you think you live now and you say you live with your children, right?
 A: Yes.
 Q: If you live together, do you think it is quite different to live together with the young people? Or do you prefer to live alone or prefer to live with your children to answer: ah. No, no, then you are all the young people are very filial piety.

 Q: Oh, that's good, because many young people are not used to living with the old people. Generally speaking, you feel very comfortable living with them, don't they?
 Answer: That is still very filial piety.
 Q: Are you usually sick, they will accompany you to see a doctor. Will come with you, won't you? For example, what you usually take medicine, they will help you to pay attention to ah or how
A: Yes, yes.
 Q: Ok, that means you think you mean you are generally satisfied, but do you really think about it? Well, from the community, or the government, or from the community or other aspects, you can make your life a little better and happier. Do you have any good advice? What can we do? From the community, ah, or from this medical staff, ah, or from the government, ah, what do you think we can do better. It can improve your satisfaction, improve your satisfaction, improve your life happiness, then you are happier.

 Answer: that generally basically in that community now, that service also generally still can ah.
 Q: Generally speaking, do you think this kind of service in their community is ok in all aspects. Does the government have any care or special care for your elderly people?
 A: Yes, yes, yes, yes,
Q: What are the benefits.

 A: For example, it was in the countryside, ah ah, I live at the age of MAO Zedong era, it was very hard at that time. Yes, yes, that substance is very good, and you will get bored. Yes, that's different now. Since I have been in the countryside since I was 65 years ago, then the government has taken care of the old man. At first, it is 50 yuan, 50 yuan a month after 55 yuan, slowly lift, slowly lift, until now there are more than 200 points.
 Q: Every month, right?
 A: Yes
Q: every month you although you this number in the outside this situation is that proportion is very small, right? But you have so much money so much old people calculate that amount is very powerful. Then you quite understand the government.
 A: Well, yes.
 Q: Do they give you, for example, the usual care is if the community pays you if the government gives you some other benefits. Ask you how well you are. That means to care about you or to check for you. You're not having anything else.

 Answer: it is a family like me, if I also had to apply at that time, is my daughter my son-in-law she is also a civil servant.

 She works in the internal department. Well, if I were ordinary and I wouldn't want that much, I would apply to the disabled government, but I was just getting right, I didn't do that. Well, even a country is not very easy either. You see, the international situation is not good, and this country costs a lot of money.
 A lot of money, if you otherwise, if the international situation is good, we can improve a little, but no way, the world is like this.
 Ask: ah, big ye, you are what work do? I think you understand the government that way,
Answer: I farm by land, farm by oneself.
 Q: Yeah, what did you don't do when you came here in' 97?

 Answer: After doing a small business, take grandchildren.
 Q: Then you just said that you just said that they are still good. But do you feel that from your own point of view, do you as an old person, do you think they can do a little better? To make you even happier? Is there any advice from here?

 A: That's not so demanding. Ok, Ok, thank you for working hard

Q: How old are you aged now?
 A: 85 years old.
 Q: Are you a local person?
 A: My hometown is in Chaoshan. I came to Shenzhen 10 years ago, and now I live in Dongguan. He had three sons and a daughter, and the sons did business, so they moved over with him.
 Q: You look so healthy.
 A: Yes, I always get sick,
Q: But you look very energetic. What are the main diseases?
 A: This heart disease. Heart at first the body is still very good, 73 years old that year car accident, originally I was often often have exercise, this his foot was broken, just stay at home for a year. He started having a bad heart at the end of age 74. Well, the heart is not good. I keep taking that heart medicine. Well. Just eat about four or five years, four or five years later began that stomach is bad, gastric ulcer, slowly chronic gastritis erosion. After this heart also this to this foot influence, began the heart is not good. The heart is not good to take medicine for a long time, the stomach is bad.be not allowed. Now you can't even take that cerebrovascular, nor is the arteriosclerosis.
 Ask: oh, this way, that this is really multiple serious diseases, that you these diseases will usually affect your life a lot, is not you usually affect your own life, or mood ah, family relations, old body health can body body is not healthy trouble. Well, then you think you now the whole of your own life, because these diseases will be your life is generally satisfied?
 A: Generally speaking, it is still ok, right?
 Q: that you and the children their family relationship, and neighbor relationship these all very good?
 A: Generally,
Q: Not so much, right? How you have your children. How many children are there?
 A: Three boys and a girl.
 Q: Oh, four children. Are you a local person or a nonlocal person?
 Answer: I chaoshan come over, how long did you come over? I started working out in Shenzhen when I was 50.
 Q: I came here in 1997. I have been here for a long time. There are still more than ten days, more than 20 years away.
 Q: Well. Oh, if you come here, will you come here for your own business, or.
 A: My son did a little business.
 Q: So you came here with your son, so you came along with you?
 A: Well.
 Q: Oh, so, that's like most of the family population is here,
A: Yes, son, what daughter-in-law, grandchildren, and these are all here.
 Q: What do you think is the happiest thing in your life now is what you are most satisfied with. What are you most satisfied with in your whole state of life?
 Answer: satisfaction is this has children to have a family, have grandson. That grandson even graduated from college and came out to work.

 Q: Generally speaking, I am quite satisfied with my own feelings and my life. Still satisfied? Or not how satisfied?
 A: Satisfied.
 Q: Do you think you are living with your children or not right now?
 A: Live together.
 Q: That means that you think you live now and you say you live with your children, right?
 A: Yes.
 Q: If you live together, do you think it is quite different to live together with the young people? Or do you prefer to live alone or prefer to live with your children to answer: ah. No, no, then you are all the young people are very filial piety.

 Q: Oh, that's good, because many young people are not used to living with the old people. Generally speaking, you feel very comfortable living with them, don't they?
 Answer: That is still very filial piety.
 Q: Are you usually sick, they will accompany you to see a doctor. Will come with you, won't you? For example, what you usually take medicine, they will help you to pay attention to ah or how
A: Yes, yes.
 Q: Ok, that means you think you mean you are generally satisfied, but do you really think about it? Well, from the community, or the government, or from the community or other aspects, you can make your life a little better and happier. Do you have any good advice? What can we do? From the community, ah, or from this medical staff, ah, or from the government, ah, what do you think we can do better. It can improve your satisfaction, improve your satisfaction, improve your life happiness, then you are happier.

 Answer: that generally basically in that community now, that service also generally still can ah.
 Q: Generally speaking, do you think this kind of service in their community is ok in all aspects. Does the government have any care or special care for your elderly people?
 A: Yes, yes, yes, yes,
Q: What are the benefits.

 A: For example, it was in the countryside, ah ah, I live at the age of MAO Zedong era, it was very hard at that time. Yes, yes, that substance is very good, and you will get bored. Yes, that's different now. Since I have been in the countryside since I was 65 years ago, then the government has taken care of the old man. At first, it is 50 yuan, 50 yuan a month after 55 yuan, slowly lift, slowly lift, until now there are more than 200 points.
 Q: Every month, right?
 A: Yes
Q: every month you although you this number in the outside this situation is that proportion is very small, right? But you have so much money so much old people calculate that amount is very powerful. Then you quite understand the government.
 A: Well, yes.
 Q: Do they give you, for example, the usual care is if the community pays you if the government gives you some other benefits. Ask you how well you are. That means to care about you or to check for you. You're not having anything else.

 Answer: it is a family like me, if I also had to apply at that time, is my daughter my son-in-law she is also a civil servant.

 She works in the internal department. Well, if I were ordinary and I wouldn't want that much, I would apply to the disabled government, but I was just getting right, I didn't do that. Well, even a country is not very easy either. You see, the international situation is not good, and this country costs a lot of money.
 A lot of money, if you otherwise, if the international situation is good, we can improve a little, but no way, the world is like this.
 Ask: ah, big ye, you are what work do? I think you understand the government that way,
Answer: I farm by land, farm by oneself.
 Q: Yeah, what did you don't do when you came here in' 97?

 Answer: After doing a small business, take grandchildren.
 Q: Then you just said that you just said that they are still good. But do you feel that from your own point of view, do you as an old person, do you think they can do a little better? To make you even happier? Is there any advice from here?

 A: That's not so demanding. Ok, Ok, thank you for working hard

Q: How old are you aged now?
 A: 86 years old.
 Q: Are you a local person?
 Answer: My hometown is in Chaoshan. I visited Shenzhen 6 years ago, and now I live in Dongguan. He had three sons and a daughter, and the sons did business, so they moved over with him.
 Q: You look so healthy.
 A: Yes, I always get sick,
Q: But you look very energetic. What are the main diseases?
 A: This heart disease. Heart at first the body is still very good, 69 years old that year car accident ah, originally I was often often have exercise ah, this his foot was broken, just stay at home for a year. I started having a bad heart at the end of my 70s. Well, the heart is not good. I keep taking that heart medicine. Well. Just eat about four or five years, four or five years later began that stomach is bad, gastric ulcer, slowly chronic gastritis erosion. After this heart also this to this foot influence, began the heart is not good. The heart is not good to take medicine for a long time, the stomach is bad.be not allowed. Now you can't even take that cerebrovascular, nor is the arteriosclerosis.
 Ask: oh, this way, that this is really multiple serious diseases, that you these diseases will usually affect your life a lot, is not you usually affect your own life, or mood ah, family relations, old body health can body body is not healthy trouble. Well, then you think you now the whole of your own life, because these diseases will be your life is generally satisfied?
 A: Generally speaking, it is still ok, right?
 Q: that you and the children their family relationship, and neighbor relationship these all very good?
 A: Generally,
Q: Not so much, right? How you have your children. How many children are there?
 A: Three boys and a girl.
 Q: Oh, four children. Are you a local person or a nonlocal person?
 Answer: I chaoshan come over, how long did you come over? I started working out in Shenzhen when I was 50.
 Q: I came here in 1997. I have been here for a long time. There are still more than ten days, more than 20 years away.
 Q: Well. Oh, if you come here, will you come here for your own business, or.
 A: My son did a little business.
 Q: So you came here with your son, so you came along with you?
 A: Well.
 Q: Oh, so, that's like most of the family population is here,
A: Yes, son, what daughter-in-law, grandchildren, and these are all here.
 Q: What do you think is the happiest thing in your life now is what you are most satisfied with. What are you most satisfied with in your whole state of life?
 Answer: satisfaction is this has children to have a family, have grandson. That grandson even graduated from college and came out to work.

 Q: Generally speaking, I am quite satisfied with my own feelings and my life. Still satisfied? Or not how satisfied?
 A: Satisfied.
 Q: Do you think you are living with your children or not right now?
 A: Live together.
 Q: That means that you think you live now and you say you live with your children, right?
 A: Yes.
 Q: If you live together, do you think it is quite different to live together with the young people? Or do you prefer to live alone or prefer to live with your children to answer: ah. No, no, then you are all the young people are very filial piety.

 Q: Oh, that's good, because many young people are not used to living with the old people. Generally speaking, you feel very comfortable living with them, don't they?
 Answer: That is still very filial piety.
 Q: Are you usually sick, they will accompany you to see a doctor. Will come with you, won't you? For example, what you usually take medicine, they will help you to pay attention to ah or how
A: Yes, yes.
 Q: Ok, that means you think you mean you are generally satisfied, but do you really think about it? Well, from the community, or the government, or from the community or other aspects, you can make your life a little better and happier. Do you have any good advice? What can we do? From the community, ah, or from this medical staff, ah, or from the government, ah, what do you think we can do better. It can improve your satisfaction, improve your satisfaction, improve your life happiness, then you are happier.

 Answer: that generally basically in that community now, that service also generally still can ah.
 Q: Generally speaking, do you think this kind of service in their community is ok in all aspects. Does the government have any care or special care for your elderly people?
 A: Yes, yes, yes, yes,
Q: What are the benefits.

 A: For example, it was in the countryside, ah ah, I live at the age of MAO Zedong era, it was very hard at that time. Yes, yes, that substance is very good, and you will get bored. Yes, that's different now. Since I have been in the countryside since I was 65 years ago, then the government has taken care of the old man. At first, it is 50 yuan, 50 yuan a month after 55 yuan, slowly lift, slowly lift, until now there are more than 200 points.
 Q: Every month, right?
 A: Yes
Q: every month you although you this number in the outside this situation is that proportion is very small, right? But you have so much money so much old people calculate that amount is very powerful. Then you quite understand the government.
 A: Well, yes.
 Q: Do they give you, for example, the usual care is if the community pays you if the government gives you some other benefits. Ask you how well you are. That means to care about you or to check for you. You're not having anything else.

 Answer: it is a family like me, if I also had to apply at that time, is my daughter my son-in-law she is also a civil servant.

 She works in the internal department. Well, if I were ordinary and I wouldn't want that much, I would apply to the disabled government, but I was just getting right, I didn't do that. Well, even a country is not very easy either. You see, the international situation is not good, and this country costs a lot of money.
 A lot of money, if you otherwise, if the international situation is good, we can improve a little, but no way, the world is like this.
 Ask: ah, big ye, you are what work do? I think you understand the government that way,
Answer: I farm by land, farm by oneself.
 Q: Yeah, what did you don't do when you came here in' 97?

 Answer: After doing a small business, take grandchildren.
 Q: Then you just said that you just said that they are still good. But do you feel that from your own point of view, do you as an old person, do you think they can do a little better? To make you even happier? Is there any advice from here?

 A: That's not so demanding. Ok, Ok, thank you for working hard

Q: How old are you aged now?
 A: 87 years old.
 Q: Are you a local person?
 Answer: My hometown is in Chaoshan. I visited Shenzhen 7 years ago, and now I live in Dongguan. He had three sons and a daughter, and the sons did business, so they moved over with him.
 Q: You look so healthy.
 A: Yes, I always get sick,
Q: But you look very energetic. What are the main diseases?
 A: This heart disease. Heart at first the body is still very good, 70 years old that year car accident, originally I was often often have exercise, this his foot broke, just stay at home for a year. I started having a bad heart at the end of age 71. Well, the heart is not good. I keep taking that heart medicine. Well. Just eat about four or five years, four or five years later began that stomach is bad, gastric ulcer, slowly chronic gastritis erosion. After this heart also this to this foot influence, began the heart is not good. The heart is not good to take medicine for a long time, the stomach is bad.be not allowed. Now you can't even take that cerebrovascular, nor is the arteriosclerosis.
 Ask: oh, this way, that this is really multiple serious diseases, that you these diseases will usually affect your life a lot, is not you usually affect your own life, or mood ah, family relations, old body health can body body is not healthy trouble. Well, then you think you now the whole of your own life, because these diseases will be your life is generally satisfied?
 A: Generally speaking, it is still ok, right?
 Q: that you and the children their family relationship, and neighbor relationship these all very good?
 A: Generally,
Q: Not so much, right? How you have your children. How many children are there?
 A: Three boys and a girl.
 Q: Oh, four children. Are you a local person or a nonlocal person?
 Answer: I chaoshan come over, how long did you come over? I started working out in Shenzhen when I was 50.
 Q: I came here in 1997. I have been here for a long time. There are still more than ten days, more than 20 years away.
 Q: Well. Oh, if you come here, will you come here for your own business, or.
 A: My son did a little business.
 Q: So you came here with your son, so you came along with you?
 A: Well.
 Q: Oh, so, that's like most of the family population is here,
A: Yes, son, what daughter-in-law, grandchildren, and these are all here.
 Q: What do you think is the happiest thing in your life now is what you are most satisfied with. What are you most satisfied with in your whole state of life?
 Answer: satisfaction is this has children to have a family, have grandson. That grandson even graduated from college and came out to work.

 Q: Generally speaking, I am quite satisfied with my own feelings and my life. Still satisfied? Or not how satisfied?
 A: Satisfied.
 Q: Do you think you are living with your children or not right now?
 A: Live together.
 Q: That means that you think you live now and you say you live with your children, right?
 A: Yes.
 Q: If you live together, do you think it is quite different to live together with the young people? Or do you prefer to live alone or prefer to live with your children to answer: ah. No, no, then you are all the young people are very filial piety.

 Q: Oh, that's good, because many young people are not used to living with the old people. Generally speaking, you feel very comfortable living with them, don't they?
 Answer: That is still very filial piety.
 Q: Are you usually sick, they will accompany you to see a doctor. Will come with you, won't you? For example, what you usually take medicine, they will help you to pay attention to ah or how
A: Yes, yes.
 Q: Ok, that means you think you mean you are generally satisfied, but do you really think about it? Well, from the community, or the government, or from the community or other aspects, you can make your life a little better and happier. Do you have any good advice? What can we do? From the community, ah, or from this medical staff, ah, or from the government, ah, what do you think we can do better. It can improve your satisfaction, improve your satisfaction, improve your life happiness, then you are happier.

 Answer: that generally basically in that community now, that service also generally still can ah.
 Q: Generally speaking, do you think this kind of service in their community is ok in all aspects. Does the government have any care or special care for your elderly people?
 A: Yes, yes, yes, yes,
Q: What are the benefits.

 A: For example, it was in the countryside, ah ah, I live at the age of MAO Zedong era, it was very hard at that time. Yes, yes, that substance is very good, and you will get bored. Yes, that's different now. Since I have been in the countryside since I was 65 years ago, then the government has taken care of the old man. At first, it is 50 yuan, 50 yuan a month after 55 yuan, slowly lift, slowly lift, until now there are more than 200 points.
 Q: Every month, right?
 A: Yes
Q: every month you although you this number in the outside this situation is that proportion is very small, right? But you have so much money so much old people calculate that amount is very powerful. Then you quite understand the government.
 A: Well, yes.
 Q: Do they give you, for example, the usual care is if the community pays you if the government gives you some other benefits. Ask you how well you are. That means to care about you or to check for you. You're not having anything else.

 Answer: it is a family like me, if I also had to apply at that time, is my daughter my son-in-law she is also a civil servant.

 She works in the internal department. Well, if I were ordinary and I wouldn't want that much, I would apply to the disabled government, but I was just getting right, I didn't do that. Well, even a country is not very easy either. You see, the international situation is not good, and this country costs a lot of money.
 A lot of money, if you otherwise, if the international situation is good, we can improve a little, but no way, the world is like this.
 Ask: ah, big ye, you are what work do? I think you understand the government that way,
Answer: I farm by land, farm by oneself.
 Q: Yeah, what did you don't do when you came here in' 97?

 Answer: After doing a small business, take grandchildren.
 Q: Then you just said that you just said that they are still good. But do you feel that from your own point of view, do you as an old person, do you think they can do a little better? To make you even happier? Is there any advice from here?

 A: That's not so demanding. Ok, Ok, thank you for working hard

Q: How old are you aged now?
 A: 88 years old.
 Q: Are you a local person?
 A: My hometown is in Chaoshan. I visited Shenzhen 8 years ago, and now I live in Dongguan. He had three sons and a daughter, and the sons did business, so they moved over with him.
 Q: You look so healthy.
 A: Yes, I always get sick,
Q: But you look very energetic. What are the main diseases?
 A: This heart disease. Heart at first the body is still very good, 71 years old that year car accident ah, originally I was often often have exercise ah, this his foot broke, just stay at home for a year. He started having a bad heart at the end of age 72. Well, the heart is not good. I keep taking that heart medicine. Well. Just eat about four or five years, four or five years later began that stomach is bad, gastric ulcer, slowly chronic gastritis erosion. After this heart also this to this foot influence, began the heart is not good. The heart is not good to take medicine for a long time, the stomach is bad.be not allowed. Now you can't even take that cerebrovascular, nor is the arteriosclerosis.
 Ask: oh, this way, that this is really multiple serious diseases, that you these diseases will usually affect your life a lot, is not you usually affect your own life, or mood ah, family relations, old body health can body body is not healthy trouble. Well, then you think you now the whole of your own life, because these diseases will be your life is generally satisfied?
 A: Generally speaking, it is still ok, right?
 Q: that you and the children their family relationship, and neighbor relationship these all very good?
 A: Generally,
Q: Not so much, right? How you have your children. How many children are there?
 A: Three boys and a girl.
 Q: Oh, four children. Are you a local person or a nonlocal person?
 Answer: I chaoshan come over, how long did you come over? I started working out in Shenzhen when I was 50.
 Q: I came here in 1997. I have been here for a long time. There are still more than ten days, more than 20 years away.
 Q: Well. Oh, if you come here, will you come here for your own business, or.
 A: My son did a little business.
 Q: So you came here with your son, so you came along with you?
 A: Well.
 Q: Oh, so, that's like most of the family population is here,
A: Yes, son, what daughter-in-law, grandchildren, and these are all here.
 Q: What do you think is the happiest thing in your life now is what you are most satisfied with. What are you most satisfied with in your whole state of life?
 Answer: satisfaction is this has children to have a family, have grandson. That grandson even graduated from college and came out to work.

 Q: Generally speaking, I am quite satisfied with my own feelings and my life. Still satisfied? Or not how satisfied?
 A: Satisfied.
 Q: Do you think you are living with your children or not right now?
 A: Live together.
 Q: That means that you think you live now and you say you live with your children, right?
 A: Yes.
 Q: If you live together, do you think it is quite different to live together with the young people? Or do you prefer to live alone or prefer to live with your children to answer: ah. No, no, then you are all the young people are very filial piety.

 Q: Oh, that's good, because many young people are not used to living with the old people. Generally speaking, you feel very comfortable living with them, don't they?
 Answer: That is still very filial piety.
 Q: Are you usually sick, they will accompany you to see a doctor. Will come with you, won't you? For example, what you usually take medicine, they will help you to pay attention to ah or how
A: Yes, yes.
 Q: Ok, that means you think you mean you are generally satisfied, but do you really think about it? Well, from the community, or the government, or from the community or other aspects, you can make your life a little better and happier. Do you have any good advice? What can we do? From the community, ah, or from this medical staff, ah, or from the government, ah, what do you think we can do better. It can improve your satisfaction, improve your satisfaction, improve your life happiness, then you are happier.

 Answer: that generally basically in that community now, that service also generally still can ah.
 Q: Generally speaking, do you think this kind of service in their community is ok in all aspects. Does the government have any care or special care for your elderly people?
 A: Yes, yes, yes, yes,
Q: What are the benefits.

 A: For example, it was in the countryside, ah ah, I live at the age of MAO Zedong era, it was very hard at that time. Yes, yes, that substance is very good, and you will get bored. Yes, that's different now. Since I have been in the countryside since I was 65 years ago, then the government has taken care of the old man. At first, it is 50 yuan, 50 yuan a month after 55 yuan, slowly lift, slowly lift, until now there are more than 200 points.
 Q: Every month, right?
 A: Yes
Q: every month you although you this number in the outside this situation is that proportion is very small, right? But you have so much money so much old people calculate that amount is very powerful. Then you quite understand the government.
 A: Well, yes.
 Q: Do they give you, for example, the usual care is if the community pays you if the government gives you some other benefits. Ask you how well you are. That means to care about you or to check for you. You're not having anything else.

 Answer: it is a family like me, if I also had to apply at that time, is my daughter my son-in-law she is also a civil servant.

 She works in the internal department. Well, if I were ordinary and I wouldn't want that much, I would apply to the disabled government, but I was just getting right, I didn't do that. Well, even a country is not very easy either. You see, the international situation is not good, and this country costs a lot of money.
 A lot of money, if you otherwise, if the international situation is good, we can improve a little, but no way, the world is like this.
 Ask: ah, big ye, you are what work do? I think you understand the government that way,
Answer: I farm by land, farm by oneself.
 Q: Yeah, what did you don't do when you came here in' 97?

 Answer: After doing a small business, take grandchildren.
 Q: Then you just said that you just said that they are still good. But do you feel that from your own point of view, do you as an old person, do you think they can do a little better? To make you even happier? Is there any advice from here?

 A: That's not so demanding. Ok, Ok, thank you for working hard

Q: How old are you aged now?
 A: 89 years old.
 Q: Are you a local person?
 A: My hometown is in Chaoshan. I visited Shenzhen 9 years ago, and now I live in Dongguan. He had three sons and a daughter, and the sons did business, so they moved over with him.
 Q: You look so healthy.
 A: Yes, I always get sick,
Q: But you look very energetic. What are the main diseases?
 A: This heart disease. Heart at first the body is very good, 72 years old that year car accident, originally I was often often have exercise, this his foot broken, just stay at home for a year. He started having a bad heart at the end of age 73. Well, the heart is not good. I keep taking that heart medicine. Well. Just eat about four or five years, four or five years later began that stomach is bad, gastric ulcer, slowly chronic gastritis erosion. After this heart also this to this foot influence, began the heart is not good. The heart is not good to take medicine for a long time, the stomach is bad.be not allowed. Now you can't even take that cerebrovascular, nor is the arteriosclerosis.
 Ask: oh, this way, that this is really multiple serious diseases, that you these diseases will usually affect your life a lot, is not you usually affect your own life, or mood ah, family relations, old body health can body body is not healthy trouble. Well, then you think you now the whole of your own life, because these diseases will be your life is generally satisfied?
 A: Generally speaking, it is still ok, right?
 Q: that you and the children their family relationship, and neighbor relationship these all very good?
 A: Generally,
Q: Not so much, right? How you have your children. How many children are there?
 A: Three boys and a girl.
 Q: Oh, four children. Are you a local person or a nonlocal person?
 Answer: I chaoshan come over, how long did you come over? I started working out in Shenzhen when I was 50.
 Q: I came here in 1997. I have been here for a long time. There are still more than ten days, more than 20 years away.
 Q: Well. Oh, if you come here, will you come here for your own business, or.
 A: My son did a little business.
 Q: So you came here with your son, so you came along with you?
 A: Well.
 Q: Oh, so, that's like most of the family population is here,
A: Yes, son, what daughter-in-law, grandchildren, and these are all here.
 Q: What do you think is the happiest thing in your life now is what you are most satisfied with. What are you most satisfied with in your whole state of life?
 Answer: satisfaction is this has children to have a family, have grandson. That grandson even graduated from college and came out to work.

 Q: Generally speaking, I am quite satisfied with my own feelings and my life. Still satisfied? Or not how satisfied?
 A: Satisfied.
 Q: Do you think you are living with your children or not right now?
 A: Live together.
 Q: That means that you think you live now and you say you live with your children, right?
 A: Yes.
 Q: If you live together, do you think it is quite different to live together with the young people? Or do you prefer to live alone or prefer to live with your children to answer: ah. No, no, then you are all the young people are very filial piety.

 Q: Oh, that's good, because many young people are not used to living with the old people. Generally speaking, you feel very comfortable living with them, don't they?
 Answer: That is still very filial piety.
 Q: Are you usually sick, they will accompany you to see a doctor. Will come with you, won't you? For example, what you usually take medicine, they will help you to pay attention to ah or how
A: Yes, yes.
 Q: Ok, that means you think you mean you are generally satisfied, but do you really think about it? Well, from the community, or the government, or from the community or other aspects, you can make your life a little better and happier. Do you have any good advice? What can we do? From the community, ah, or from this medical staff, ah, or from the government, ah, what do you think we can do better. It can improve your satisfaction, improve your satisfaction, improve your life happiness, then you are happier.

 Answer: that generally basically in that community now, that service also generally still can ah.
 Q: Generally speaking, do you think this kind of service in their community is ok in all aspects. Does the government have any care or special care for your elderly people?
 A: Yes, yes, yes, yes,
Q: What are the benefits.

 A: For example, it was in the countryside, ah ah, I live at the age of MAO Zedong era, it was very hard at that time. Yes, yes, that substance is very good, and you will get bored. Yes, that's different now. Since I have been in the countryside since I was 65 years ago, then the government has taken care of the old man. At first, it is 50 yuan, 50 yuan a month after 55 yuan, slowly lift, slowly lift, until now there are more than 200 points.
 Q: Every month, right?
 A: Yes
Q: every month you although you this number in the outside this situation is that proportion is very small, right? But you have so much money so much old people calculate that amount is very powerful. Then you quite understand the government.
 A: Well, yes.
 Q: Do they give you, for example, the usual care is if the community pays you if the government gives you some other benefits. Ask you how well you are. That means to care about you or to check for you. You're not having anything else.

 Answer: it is a family like me, if I also had to apply at that time, is my daughter my son-in-law she is also a civil servant.

 She works in the internal department. Well, if I were ordinary and I wouldn't want that much, I would apply to the disabled government, but I was just getting right, I didn't do that. Well, even a country is not very easy either. You see, the international situation is not good, and this country costs a lot of money.
 A lot of money, if you otherwise, if the international situation is good, we can improve a little, but no way, the world is like this.
 Ask: ah, big ye, you are what work do? I think you understand the government that way,
Answer: I farm by land, farm by oneself.
 Q: Yeah, what did you don't do when you came here in' 97?

 Answer: After doing a small business, take grandchildren.
 Q: Then you just said that you just said that they are still good. But do you feel that from your own point of view, do you as an old person, do you think they can do a little better? To make you even happier? Is there any advice from here?

 A: That's not so demanding. Ok, Ok, thank you for working hard

Q: How old are you aged now?
 A: 90 years old.
 Q: Are you a local person?
 A: My hometown is in Chaoshan. I came to Shenzhen 10 years ago, and now I live in Dongguan. He had three sons and a daughter, and the sons did business, so they moved over with him.
 Q: You look so healthy.
 A: Yes, I always get sick,
Q: But you look very energetic. What are the main diseases?
 A: This heart disease. Heart at first the body is still very good, 73 years old that year car accident, originally I was often often have exercise, this his foot was broken, just stay at home for a year. He started having a bad heart at the end of age 74. Well, the heart is not good. I keep taking that heart medicine. Well. Just eat about four or five years, four or five years later began that stomach is bad, gastric ulcer, slowly chronic gastritis erosion. After this heart also this to this foot influence, began the heart is not good. The heart is not good to take medicine for a long time, the stomach is bad.be not allowed. Now you can't even take that cerebrovascular, nor is the arteriosclerosis.
 Ask: oh, this way, that this is really multiple serious diseases, that you these diseases will usually affect your life a lot, is not you usually affect your own life, or mood ah, family relations, old body health can body body is not healthy trouble. Well, then you think you now the whole of your own life, because these diseases will be your life is generally satisfied?
 A: Generally speaking, it is still ok, right?
 Q: that you and the children their family relationship, and neighbor relationship these all very good?
 A: Generally,
Q: Not so much, right? How you have your children. How many children are there?
 A: Three boys and a girl.
 Q: Oh, four children. Are you a local person or a nonlocal person?
 Answer: I chaoshan come over, how long did you come over? I started working out in Shenzhen when I was 50.
 Q: I came here in 1997. I have been here for a long time. There are still more than ten days, more than 20 years away.
 Q: Well. Oh, if you come here, will you come here for your own business, or.
 A: My son did a little business.
 Q: So you came here with your son, so you came along with you?
 A: Well.
 Q: Oh, so, that's like most of the family population is here,
A: Yes, son, what daughter-in-law, grandchildren, and these are all here.
 Q: What do you think is the happiest thing in your life now is what you are most satisfied with. What are you most satisfied with in your whole state of life?
 Answer: satisfaction is this has children to have a family, have grandson. That grandson even graduated from college and came out to work.

 Q: Generally speaking, I am quite satisfied with my own feelings and my life. Still satisfied? Or not how satisfied?
 A: Satisfied.
 Q: Do you think you are living with your children or not right now?
 A: Live together.
 Q: That means that you think you live now and you say you live with your children, right?
 A: Yes.
 Q: If you live together, do you think it is quite different to live together with the young people? Or do you prefer to live alone or prefer to live with your children to answer: ah. No, no, then you are all the young people are very filial piety.

 Q: Oh, that's good, because many young people are not used to living with the old people. Generally speaking, you feel very comfortable living with them, don't they?
 Answer: That is still very filial piety.
 Q: Are you usually sick, they will accompany you to see a doctor. Will come with you, won't you? For example, what you usually take medicine, they will help you to pay attention to ah or how
A: Yes, yes.
 Q: Ok, that means you think you mean you are generally satisfied, but do you really think about it? Well, from the community, or the government, or from the community or other aspects, you can make your life a little better and happier. Do you have any good advice? What can we do? From the community, ah, or from this medical staff, ah, or from the government, ah, what do you think we can do better. It can improve your satisfaction, improve your satisfaction, improve your life happiness, then you are happier.

 Answer: that generally basically in that community now, that service also generally still can ah.
 Q: Generally speaking, do you think this kind of service in their community is ok in all aspects. Does the government have any care or special care for your elderly people?
 A: Yes, yes, yes, yes,
Q: What are the benefits.

 A: For example, it was in the countryside, ah ah, I live at the age of MAO Zedong era, it was very hard at that time. Yes, yes, that substance is very good, and you will get bored. Yes, that's different now. Since I have been in the countryside since I was 65 years ago, then the government has taken care of the old man. At first, it is 50 yuan, 50 yuan a month after 55 yuan, slowly lift, slowly lift, until now there are more than 200 points.
 Q: Every month, right?
 A: Yes
Q: every month you although you this number in the outside this situation is that proportion is very small, right? But you have so much money so much old people calculate that amount is very powerful. Then you quite understand the government.
 A: Well, yes.
 Q: Do they give you, for example, the usual care is if the community pays you if the government gives you some other benefits. Ask you how well you are. That means to care about you or to check for you. You're not having anything else.

 Answer: it is a family like me, if I also had to apply at that time, is my daughter my son-in-law she is also a civil servant.

 She works in the internal department. Well, if I were ordinary and I wouldn't want that much, I would apply to the disabled government, but I was just getting right, I didn't do that. Well, even a country is not very easy either. You see, the international situation is not good, and this country costs a lot of money.
 A lot of money, if you otherwise, if the international situation is good, we can improve a little, but no way, the world is like this.
 Ask: ah, big ye, you are what work do? I think you understand the government that way,
Answer: I farm by land, farm by oneself.
 Q: Yeah, what did you don't do when you came here in' 97?

 Answer: After doing a small business, take grandchildren.
 Q: Then you just said that you just said that they are still good. But do you feel that from your own point of view, do you as an old person, do you think they can do a little better? To make you even happier? Is there any advice from here?

 A: That's not so demanding. Ok, Ok, thank you for working hard
